# Low Molecular Weight Dextran Sulfate Mitigates Acute Kidney Injury Induced by Renal Ischemia/Reperfusion: Implication of JNK/p38 MAPK and Caspase-3

**DOI:** 10.3390/antiox15101238

**Published:** 2026-09-25

**Authors:** Nadrah S. Alamri, Ahlam M. Alhusaini, Wedad S. Sarawi, Hala A. Attia, Iman H. Hasan, Alia O. Alshabanah, Ragad A. Alharajeen, Ahmed A. Altuwaijri, Faris A. Almutairi, Hanaa M. Altuwaijri, Dalal A. Alkhelb, Sahar S. Alotaibi, Abdullah Y. Almubarak, Yasser A. Alshawakir

**Affiliations:** 1 Department of Pharmacology and Toxicology, College of Pharmacy, King Saud University, P.O. Box 2457, Riyadh 11451, Saudi Arabia; 446201192@student.ksu.edu.sa (N.S.A.); wsarawi@ksu.edu.sa (W.S.S.); hsalem@ksu.edu.sa (H.A.A.); ihasan@ksu.edu.sa (I.H.H.); 443200204@student.ksu.edu.sa (A.O.A.); 443202689@student.ksu.edu.sa (R.A.A.); aaltuwaijri@ksu.edu.sa (A.A.A.); faralmutairi@ksu.edu.sa (F.A.A.); dalkhelb@ksu.edu.sa (D.A.A.); 445205548@student.ksu.edu.sa (S.S.A.); 2 Department of Pharmaceutical Chemistry, College of Pharmacy, King Saud University, P.O. Box 2457, Riyadh 11451, Saudi Arabia; haltuwajri@ksu.edu.sa; 3 Experimental Surgery and Animal Lab, Prince Naif for Health Research Center (PNHRC), College of Medicine, King Saud University, P.O. Box 2925, Riyadh 11451, Saudi Arabia; aalmubarak@ksu.edu.sa (A.Y.A.); yalshawakir@ksu.edu.sa (Y.A.A.)

**Keywords:** renal ischemia/reperfusion injury, low molecular weight-dextran sulfate, oxidative stress, inflammation, apoptosis

## Abstract

Renal ischemia/reperfusion injury (RI/RI) is one of the major causes of acute kidney injury (AKI). Low molecular weight-dextran sulphate (LMW-DS) is a bioactive polysaccharide with anti-inflammatory and cytoprotective properties. This study investigated the nephroprotective potential of LMW-DS against the RI/RI. RI/RI rat model underwent surgery that involved a complete bilateral clamping of renal hila for 30 min, followed by unclamping for 30 min. LMW-DS (5 mg/kg, s.c.) was injected 5 min before unclamping. After 24 h, kidneys were collected for biochemical, histopathological, and immunohistochemical examinations. RI/RI dramatically induced renal deterioration, as reflected by marked elevation in the levels of renal oxidative stress, tumor necrosis factor-α, interleukin-6, and toll-like receptor-4, indicating lipid peroxidation and inflammatory response. Furthermore, RI/RI caused upregulation of kidney injury molecule-1 and neutrophil gelatinase-associated lipocalin. Additionally, morphological alterations and collagen deposition were also detected in kidney tissues. The immunohistochemical analysis revealed a strong positive immunoreaction for p38 mitogen-activated protein kinase, c-Jun N-terminal kinase, and caspase-3. Treatment with LMW-DS produced a pronounced amelioration in the altered parameters induced by RI/RI. In conclusion, LMW-DS exerts nephroprotective effects against RI/RI by restoring redox balance, suppressing inflammatory signaling, JNK/p38 MAPK, and caspase-3, highlighting its potential as a therapeutic strategy for renal I/R-induced AKI.

## 1. Introduction

Renal ischemia/reperfusion injury (RI/RI) is one of the most common causes of acute kidney injury (AKI), and it is a leading factor for renal transplantation failure. Due to their high metabolic demand, renal tubular epithelial cells are particularly vulnerable to ischemia. Reduced blood flow results in oxygen deprivation, forcing cells to rely on anaerobic metabolism, which leads to lactate accumulation and acidosis. Ischemia also disrupts ion homeostasis, causing intracellular sodium and calcium overload [1]. Elevated intracellular calcium levels promote the opening of mitochondrial permeability transition pores, aggravating reactive oxygen species (ROS) production and leading to cell death [2]. Upon reperfusion, the sudden reintroduction of oxygen elevates ROS production by activating the NADPH oxidase enzyme, which is the main source of superoxide radicals [3]. Excessive ROS leads to an oxidative burst that overwhelms the cellular antioxidants, such as reduced glutathione (GSH) and superoxide dismutase (SOD), which are known to be involved in the endogenous antioxidant system by neutralizing ROS and maintaining redox homeostasis [4]. ROS attack polyunsaturated fatty acids in cell membranes, leading to cellular damage and the formation of lipid peroxidation byproduct Malondialdehyde (MDA) [4].

Inflammation plays a central, complex, and critical pathological role in RI/RI, where the transition from ischemia to reperfusion can convert the initial injury into sustained damage, leading to AKI [5]. Moreover, the disruption of endothelial monolayers and glycocalyx caused by ischemia leads to an increase in vascular permeability, allowing harmful substances and cells to penetrate kidney tissues [6]. Ischemic damage triggers the release of damage-associated molecular patterns (DAMPs), leading to Toll-like receptor 4 (TLR-4)/Nuclear factor kappa B (NF-κB) activation and an increase in the production of pro-inflammatory cytokines, including Tumor necrosis factor-alpha (TNF-α) and Interleukin-6 (IL-6) [7,8]. These mediators exacerbate tubular injury, leukocyte infiltration, and cell death, while sustained IL-6/STAT3 signaling contributes to fibrosis and maladaptive renal repair [9,10].

In addition, apoptosis is a major mechanism of tubular cell loss in RI/RI. Ischemia-induced ATP depletion, oxidative stress, and calcium overload disrupt mitochondrial integrity, leading to cytochrome c release and activation of the caspase cascade, particularly caspase-3 [11]. In parallel, inflammatory cytokines such as TNF-α activate extrinsic apoptotic signaling through death receptors [12]. During reperfusion, excessive ROS generation and calcium accumulation further aggravate mitochondrial dysfunction and promote the expression of kidney injury molecule-1 (KIM-1), a sensitive marker of tubular injury. KIM-1 has been linked to MAPK signaling pathways, including extracellular signal-regulated kinase (ERK) and c-Jun N-terminal kinase (JNK), which regulate cellular stress responses and contribute to renal injury progression [13].

These studies have improved our understanding of the pathophysiological mechanisms of RI/RI and identified critical pathways involved in renal injury. However, RI/RI is still a challenging condition because no efficient and safe medication has been clinically proven for preventing or treating this state. Thus, there is a need to explore an agent with a clear safety profile and clinical value. Low-molecular-weight dextran sulfate (LMW-DS) is a sulfated negatively charged polysaccharide with an average molecular weight of approximately 5 kilodaltons. It possesses anti-inflammatory, anticoagulant, and neuroprotective properties [14,15]. Previous studies have shown that LMW-DS can inhibit complement activation, suppress TLR/NF-κB-mediated inflammatory signaling, reduce leukocyte adhesion and infiltration, and attenuate oxidative stress by restoring antioxidant defenses and reducing lipid peroxidation [16,17,18]. Moreover, LMW-DS has demonstrated anti-apoptotic effects through preservation of mitochondrial integrity, inhibition of caspase activation, and modulation of cell survival pathways [19]. Despite these promising biological activities, the potential nephroprotective role of LMW-DS in RI/RI has not been fully investigated. This study aims to investigate the nephroprotective effect of LMW-DS against the RI/RI rat model, focusing on its antioxidant, anti-inflammatory, and anti-apoptotic effects.

## 2. Materials and Methods

### 2.1. Materials

LMW-DS was obtained from TdB Labs (Ultuna, Uppsala, Sweden, Cat#DB005) and prepared by dissolving it in normal saline to reach the final concentration of 2.33 mg/mL (0.43 mL/200 g rat). For the surgical procedure, Ketamine and xylazine chloride were purchased from Alfasan, (Woerden, The Netherlands), and Povidone Iodine 10% *w*/*v* was obtained from (Riyadh Pharma, Riyadh, Riyadh Province, Saudi Arabia). Sevoflurane Inhalation was sourced from (Baxter Healthcare Corporation, Deerfield, IL, USA). Urea, uric acid, and creatinine colorimetric kits were purchased from (Human, Wiesbaden, Germany). SOD activity, GSH Content, and MDA Content assay kits were obtained from (Solarbio, Beijing, China). Moreover, the TLR-4, TNF-α, IL-6, KIM-1, and neutrophil gelatinase-associated lipocalin (NGAL) enzyme-linked immunosorbent assay (ELISA) kits were purchased from (Solarbio, Beijing, China). Primary antibodies used in this study included monoclonal P38 MAPK (Cell Signaling Technology, Danvers, MA, USA), monoclonal JNK1/JNK2 (Invitrogen, Carlsbad, CA, USA), and polyclonal Caspase-3 (Abcam, Cambridge, UK). VECASTAIN Elite ABC Kit and DAB Substrate Kit were bought from (Vector Laboratories, Newark, CA, USA).

### 2.2. Animals

Thirty-two healthy male Wistar albino rats weighing 200–250 g were obtained from the Experimental Surgery and Animal Laboratory, Prince Naif Center for Health Research, King Saud University. The sample size was calculated according to “resource equation” method [20].

The rats were randomly divided into 4 groups (*n* = 8) and housed under standard conditions, including temperature, 12-h light/dark cycle, and humidity, with free access to food and water. They were kept for at least two weeks before starting the experiment to adapt to the laboratory environment. All experiments were carried out in accordance with the recommendations of the Research Ethics Committee and the Institutional Animal Care and Use Committee (IACUC). The research protocol was approved by the Scientific Research Ethics Committee, King Saud University (KSU-SE-25-16) on (5 March 2025), and reported in accordance with ARRIVE guidelines [21]. In addition, we performed the experiment, investigated the outcomes, and analyzed the data blindly to eliminate bias.

### 2.3. Method

#### 2.3.1. Induction of RI/RI

The animals were anesthetized and maintained under anesthesia using ketamine/xylazine (100/20 mg/kg, i.p.), and the body temperature was kept at 37 °C ± 0.5 using a heating pad until the completion of the surgery. Rats were positioned prone, and their limbs were secured with tape to achieve full extension. Then, the flank area was shaved with an electric shaver. Next, the skin was disinfected using povidone-iodine solution. Two vertical flank incisions were made using surgical scissors to sequentially penetrate the skin, fascia, and muscle. The kidneys were swept laterally outward and separated from the surrounding retroperitoneal fat using a cotton swab to expose the renal hilum. Clamping of the renal hilum was performed using medical forceps for 30 min, followed by unclamping to reintroduce blood to the kidneys for 30 min (to ensure successful reperfusion) [22] (Figure 1). The kidneys were covered with gauze soaked in normal saline to avoid dryness. After the specified periods of clamping and unclamping, the kidneys were returned inside the body, and the wounds were stitched. Fusidic acid cream was applied over the wounds to protect rats from post-operative infections. No post-operative analgesic drug was given to the animals.

#### 2.3.2. LMW-DS Administration and Sample Collection

- In sham rats (**groups 1 and 2**), surgery was done without clamping the renal hilum. Thirty min later, rats in group 1 or 2 were subcutaneously injected with either normal saline or LMW-DS (5 mg/kg) [18], respectively.
- In the RI/RI rats (**group 3**), the kidneys’ hila were completely clamped for 30 min, followed by unclamping for 30 min. Normal saline was injected subcutaneously into the dorsal cervical region 5 min before unclamping, and then the kidneys were returned to their place after ensuring reperfusion.
- In the RI/RI+LMW-DS pre-reperfusion rats (**group 4**), the kidneys’ hila were completely clamped for 30 min, followed by unclamping for 30 min. LMW-DS was injected subcutaneously into the dorsal cervical region 5 min before unclamping, and then the kidneys were returned to their place after ensuring reperfusion. LMW-DS has a rapid onset of action; because it is a strongly negative charged polysaccharide, allowing rapid diffusion to circulatory system through tissue fluid after subcutaneous injection [18].

After 24 h of surgery, the rats were anesthetized by sevoflurane inhalation. The blood was collected via cardiac puncture, allowed to coagulate for 30 min, and then centrifuged for 30 min at 3000 rpm to obtain serum, which was kept in a −80 °C freezer. The rats were euthanized by cervical dislocation to ensure complete death. Kidneys were then rapidly harvested, and parts of the kidney tissues from each rat were frozen in liquid nitrogen and stored at −80 °C (Figure 2). The other parts were fixed in a buffered formalin solution (10%) for subsequent histopathological and immunohistochemical (IHC) examinations. Tissue homogenate was obtained by mixing 1 g of kidney tissue with 5 mL of normal saline, followed by homogenization using a tissue homogenizer. Then the mixture was centrifuged for 30 min at 3000 rpm, and the supernatant was collected and kept in a −80 °C freezer for further biochemical analyses.

#### 2.3.3. Assessment of Kidney Function

Kidney function was assessed by the determination of urea (Cat# 10505), uric acid (Cat# 10690), and creatinine (Cat# 10052) in serum. These assays were performed according to the manufacturer’s instructions, and the concentrations of the kidney function markers were quantified spectrophotometrically.

#### 2.3.4. Assessment of Oxidative Stress

The levels of SOD (Cat# BC0175), GSH (Cat# BC1175), and MDA (Cat# BC0025) were assessed colorimetrically in tissue homogenate and their levels were normalized to the initial wet tissue mass according to the manufacturer’s instructions.

#### 2.3.5. Determination of Inflammation and Renal Injury

The levels of TLR-4 (Cat# SEKR-0184), TNF-α (Cat# SEKR-0009), IL-6 (Cat# SEKR-0005), KIM-1 (Cat# SEKR-0145), and NGAL (Cat# SEKR-0028) in tissue homogenates were determined using ELISA kits (Solarbio, Beijing, China). The ELISA concentrations were determined from assay-specific standard curves. Concentrations of the analytes were determined from assay-specific standard curves and reported as concentrations in the homogenate supernatant.

#### 2.3.6. Histopathological and Immunohistochemical Assessment

The specimens were extracted and kept in 10% formalin overnight at room temperature. Then they were trimmed with a scalpel into a size compatible with standard labeled cassettes in preparation for histological analysis. The sections were subsequently stained with hematoxylin and eosin (H&E) for general histopathological evaluation and with Masson’s trichrome stain for collagen deposition assessment. Kidney damage was examined blindly by an experienced histologist, and kidney sections were applied to histopathological scoring system and achieved through evaluation of 3 fields (magnification ×400) per section for each rat (8 rats/group). The scoring was focused on the following parameters: glomerular atrophy, tubular degeneration, interstitial inflammation, congestion or hemorrhage, collagen, and cast formation according to the following scale: 0 = non, 1 = Minimal damage (<10% of tissue affected), 2 = Mild damage (10–25%), 3 = Moderate damage (26–50%), 4 = Severe damage (51–75%), 5 = Very severe damage (>75%) [23].

The expressions of p38 MAPK, JNK, and caspase-3 were detected in kidney sections using immunohistochemical staining. Paraffin-embedded kidney sections were deparaffinized and rehydrated using graded ethanol. Sections were subjected to heat-induced antigen retrieval using EDTA buffer (pH = 9). After blocking, the sections were incubated overnight at 4 °C with primary antibodies against p38 MAPK (Cat# 9212), JNK1/JNK2 (Cat# 279Q38), and caspase-3 (Cat# ab4051) at a dilution of 1:50. Following PBS washes, sections were incubated with appropriate secondary antibodies (anti-rabbit IgG, Cat# 31820; anti-mouse IgG, Cat# 31181). Immunoreactivity was visualized using the avidin-biotin complex (ABC, Cat# PK-6100) method with 3,3′-diaminobenzidine (DAB, Cat# SK-4100) as a chromogen. Subsequently, the sections were counterstained with hematoxylin, dehydrated, and finally mounted using DePeX mounting medium. Immunoreactivity was evaluated by examining the stained tissue sections under an Optika light microscope. The quantitative analysis of Masson’s trichrome stain and IHC was performed by measuring the percentage of optical density (%OD) and stain intensity by evaluating 3 fields (magnification ×400) per section for each rat (8 rats/group), focusing on the renal medulla, including glomeruli and tubules, using Fiji software, x86-64 (Dresden, Germany) [24] with the threshold set to 0–255.

#### 2.3.7. Statistical Analysis

All results were presented as mean ± standard error of the mean (SEM). Multiple comparisons among all groups were performed using One-way ANOVA, followed by Tukey post hoc test using GraphPad Prism^®^ software, 10. 6.1 (San Diego, CA, USA). Statistical significance was defined as *p* ≤ 0.05.

## 3. Results

### 3.1. Biochemical Studies

#### 3.1.1. Effect of LMW-DS on Kidney Function in RI/RI Rat Model

Figure 3A–C illustrate the impact of LMW-DS treatment on serum renal function markers. RI/RI significantly elevated urea (*p* < 0.0001), uric acid (*p* < 0.0001), and creatinine (*p* < 0.001) levels compared with sham-operated animals. Treatment with LMW-DS prior to reperfusion markedly reduced the levels of urea (*p* < 0.0001), uric acid (*p* < 0.001), and creatinine (*p* < 0.0001) compared to the RI/RI group, reflecting preservation of renal function.

#### 3.1.2. Effect of LMW-DS on Oxidative Stress in RI/RI Rat Model

RI/RI significantly reduced the antioxidant defenses, as evidenced by a significant reduction in GSH level and SOD activity (*p* < 0.0001), with markedly increased MDA levels (*p* < 0.0001) compared with the sham groups (Figure 4A–C). Administration of LMW-DS prior to reperfusion significantly restored GSH levels (*p* < 0.0001) and SOD activity (*p* < 0.01), and reduced MDA levels (*p* < 0.0001) relative to the RI/RI group (Figure 4A–C), indicating attenuation of oxidative stress.

#### 3.1.3. Effect of LMW-DS on Inflammatory Response in RI/RI Rat Model

The influence of LMW-DS treatment on inflammatory biomarkers in rats subjected to RI/RI is presented in Figure 5A–C. RI/RI significantly increased the levels of the pro-inflammatory cytokines TNF-α, IL-6, and TLR-4 (*p* < 0.0001) compared with the sham groups, indicating activation of the inflammatory response. Administration of LMW-DS before reperfusion significantly reduced the expression levels of TNF-α, IL-6, and TLR-4 (*p* < 0.0001) relative to the RI/RI group, with no significant differences observed between the sham groups (Figure 5A–C).

#### 3.1.4. Effect of LMW-DS on Renal Injury Biomarkers Following RI/RI

As shown in Figure 6A,B, RI/RI significantly elevated KIM-1 (*p* < 0.001) and NGAL (*p* < 0.05) levels compared with sham groups, indicating substantial renal tubular injury. Treatment with LMW-DS attenuated the elevated levels of KIM-1 (*p* < 0.01) and NGAL (*p* < 0.05) relative to the RI/RI group, suggesting a protective effect against RI/RI-induced renal damage (Figure 6A,B).

### 3.2. Histopathological Assessment

As illustrated in Figure 7, Kidney sections stained with H&E showed histopathological alterations induced by RI/RI. Control groups exhibited normal renal architecture with intact glomeruli and renal tubules (Figure 7A,B). In contrast, the RI/RI group showed marked histopathological alterations, including vascular congestion, hemorrhage, inflammatory cell infiltration, tubular and cellular degenerations, and glomerular shrinkage (Figure 7C). LMW-DS attenuated the pathological changes and preserved partial renal tissue architecture, supporting a protective effect against RI/RI-induced renal injury (Figure 7D).

Moreover, the analysis of Masson’s trichrome-stained tissues indicated collagenous deposition alterations among the different experimental groups (Figure 8). RI/RI group showed extensive collagen accumulations accompanied by vascular congestion (Figure 8C) compared to minimal collagen deposition in control groups (Figure 8A,B). LMW-DS reduced collagen deposition (Figure 8D). The quantitative analysis of Masson’s trichrome-stained tissues revealed a significant elevation of the percentage of optical density (OD%) in RI/RI group compared to control group (*p* < 0.0001) while using LMW-DS markedly reduced the accumulated collagen induced by RI/RI (*p* < 0.0001) (Figure 8E).

The scoring of morphological alterations of renal tissues among all groups, including glomerular atrophy, tubular degeneration, interstitial inflammation, hemorrhage, collagen, and casts is expressed in Table 1. Sham and LMW-DS groups exhibited normal renal architecture with zero score in all measured parameters. However, kidney sections from the RI/RI group demonstrated pronounced pathological alterations and an increase in renal damage severity which were reflected by the significant increase in all measured parameters (*p* < 0.0001) compared with the sham group. In the RI/RI+LMW-DS group, the kidney sections displayed a significant reduction in glomerular atrophy (*p* < 0.05), tubular degeneration (*p* < 0.01), interstitial inflammation (*p* < 0.01), hemorrhage (*p* < 0.01), collagen deposition (*p* < 0.05), and cast formation (*p* < 0.05) in comparison with the RI/RI group, indicating a partial improvement in the morphological alterations induced by RI/RI.

### 3.3. Immunohistochemical Analysis

#### 3.3.1. Effect of LMW-DS on Renal p38 MAPK Expression in RI/RI in Rats

The immunohistochemical analysis of p38 MAPK in kidney tissues revealed a normal histological architecture with low immunoreactivity in both control groups (Figure 9A,B). In contrast, robust p38 MAPK upregulation was observed in the RI/RI group (Figure 9C). The administration of LMW-DS in the RI/RI group was associated with attenuation of p38 MAPK immunoreactivity in comparison with the RI/RI group (Figure 9D). As illustrated in Figure 9E, RI/RI group exhibited a significant elevation of the p38 MAPK immunoreactivity compared to sham group (*p* < 0.0001); however, the administration of LMW-DS significantly reduced p38 MAPK expression in the kidney sections (*p* < 0.0001).

#### 3.3.2. Effect of LMW-DS on Renal JNK (JNK1/JNK2) Expression in RI/RI in Rats

A mild expression of JNK within renal tubular epithelial cells and glomerular cells was shown in control groups (Figure 10A,B). In contrast, the RI/RI group exhibits a strong positive JNK immunoreactivity (Figure 10C). On the other hand, RI/RI rats treated with LMW-DS showed moderate JNK immunoreactivity within tubular and glomerular structures compared with the RI/RI group (Figure 10D). Figure 10E presented a marked increase in the JNK expression in kidney samples of the RI/RI group in comparison with control group (*p* < 0.0001); in contrast the group treated with LMW-DS exhibited a prominent reduction in the JNK expression compared to the diseased group (*p* < 0.0001).

#### 3.3.3. Effect of LMW-DS on Renal Caspase-3 Expression in RI/RI Rats

Immunohistochemical analysis of caspase-3 expression in kidney tissues revealed distinct differences among the four experimental groups (Figure 11). Sham groups showed minimal basal caspase-3 and mild pro-apoptotic immunoreactivity (Figure 11A,B). Kidney sections obtained from the RI/RI group manifested a pronounced upregulation of caspase-3 with a widespread and strong immunoreactivity localized in the tubular epithelium (Figure 11C). RI/RI rats that were treated with LMW-DS exhibited a moderate degree of caspase-3 expression, with immunopositivity signals distributed in both glomerular and tubular compartments (Figure 11D). As shown in Figure 11E, the RI/RI group exhibited a pronounced elevation in caspase-3 expression compared to the control group (*p* < 0.0001), whereas the administration of LMW-DS caused significant reduction in the expression of caspase-3 when compared to the untreated group (*p* < 0.0001).

## 4. Discussion

The kidney is a vital organ that helps maintain systemic homeostasis [25]. It maintains homeostasis by regulating fluid and electrolyte balance and eliminating xenobiotics and metabolic waste products, such as urea, uric acid, and creatinine [26]. Although the kidneys represent a relatively small fraction of the body, they exhibit high oxygen demand and require ATP to sustain active transport and mitochondrial phosphorylation, thereby maintaining systemic homeostasis. This demand shows that any disruption in oxygen supply, caused by toxins, chronic diseases, or even ischemia, will lead to energy depletion and cellular injury [27]. RI/RI is a pathological condition disrupting the kidney’s normal physiology, causing AKI and renal transplantation failure. It is initiated by the sudden reintroduction of blood after ischemia, amplifying tissue damage by inducing oxidative stress, inflammation, and apoptosis responses [28]. Lack of oxygen can initiate a redox imbalance, damaged tubular epithelial cells by promoting excessive production of pro-inflammatory cytokines [7]. Moreover, mitochondrial dysfunction, tubular damage, inflammatory cascades, and the imbalance between pro-apoptotic and anti-apoptotic proteins trigger apoptosis cascades [29,30]. These events, if unresolved, can lead to progression toward chronic kidney disease (CKD) [31].

In the present study, rats underwent RI/RI and exhibited a significant elevation in serum levels of urea, uric acid, and creatinine, reflecting initial impairment in renal function. The accumulation of non-protein nitrogen compounds, such as urea and creatinine, indicates impairment in the renal clearance capacity [32]. Elevated uric acid levels may contribute to oxidative stress and endothelial dysfunction, indicating renal injury [33]. The increased uric acid in the RI/RI group could be explained by enhanced purine degradation, driven by increased nucleotide degradation and oxidative stress [34]. A recent study in a rat model showed that elevations in creatinine and blood urea nitrogen levels were clearly observed in the ischemic group and were linked to tubular injury and loss of renal functional integrity [35]. Our findings suggested a nephroprotective effect of LMW-DS against RI/RI, as LMW-DS restored levels of urea, uric acid, and creatinine, supporting its potential role in the recovery of renal function. The observed attenuation of urea, uric acid, and creatinine levels may be attributed to its ability to improve renal microcirculation through its anticoagulant and antioxidant effects [16,18].

Furthermore, the current study revealed that rats with RI/RI exhibited a failure of redox homeostasis, evidenced by depletion of the antioxidants GSH and SOD, alongside the elevation of MDA levels. Our findings are in line with studies indicating that the reperfusion phase triggers ROS overproduction and initiates a cascade of lipid peroxidation and endothelial cell damage [36]. Particularly, mitochondrial dysfunction plays a crucial role in ROS overproduction, creating a cycle of cellular injury [37]. In addition, we showed that LMW-DS significantly attenuated oxidative stress by restoring endogenous antioxidant levels and decreasing lipid peroxidation end-product (MDA). Consistent with our findings, a previous study showed that LMW-DS significantly reduced oxidative and nitrosative stress markers and improved mitochondrial function in patients with amyotrophic lateral sclerosis [15]. Similarly, another study reported that LMW-DS restored mitochondrial energy metabolism and attenuated oxidative damage in a rat model of severe traumatic brain injury [18]. Notably, the current findings extend these observations to renal tissues, proving that LMW-DS restored redox balance by enhancing endogenous antioxidant levels and limiting lipid peroxide production.

Additionally, inflammation has a major role in the current RI/RI rat model. The present study demonstrated a significant increase in the levels of TNF-α, IL-6, and TLR-4 following RI/RI, indicating upregulation of the pro-inflammatory response. Ischemic damage triggers the release of DAMPs, leading to the activation of the TLR-4/NF-κB signaling pathway, which further promotes the production of pro-inflammatory cytokines, including TNF-α and IL-6, from the damaged renal tubular cells, enhancing inflammation [7,8]. Our findings also revealed that LMW-DS significantly attenuates the levels of TNF-α, IL-6, and TLR-4, indicating its role as an anti-inflammatory agent. Our results are consistent with a study that reported the anti-inflammatory effect of LMW-DS on human dendritic cells by inhibiting the upregulation of the pro-inflammatory mediators IL-1, IL-6, TNF-α, and TLR-4. The downregulation of TLR-4 results in attenuation of NF-κB, which mediates the expression of IL-6 and TNF-α [17].

To investigate the relationship between renal inflammation and tissue injury, we examined the roles of NGAL, KIM-1, P38 MAPK, and JNK in kidney damage induced by renal I/R. RI/RI significantly increased the expression of NGAL, KIM-1, P38 MAPK, and JNK compared to the sham groups, indicating severe tubular injury accompanied by upregulation of inflammatory signaling pathways. NGAL is an inflammatory biomarker produced by various renal cell types, particularly distal tubular epithelial cells, indicating tubular injury [38]. In addition, KIM-1 is a transmembrane glycoprotein that is minimally expressed in healthy kidneys but markedly elevated following ischemic proximal tubular injury, supporting its role as a sensitive biomarker of AKI [39]. Our findings are consistent with experimental and clinical studies that have shown significant elevations in urinary and serum NGAL and KIM-1 following AKI [40,41]. Another study identified NGAL and KIM-1 as the most sensitive biomarkers for the early detection of AKI, demonstrating their rapid elevation and superiority over conventional renal function markers for early recognition [42]. The less pronounced changes in tissue NGAL levels compared with KIM-1 may reflect the transient nature of NGAL expression. NGAL rises rapidly following ischemic injury and is most commonly detected in urine and serum, whereas KIM-1 remains elevated during the later stages of tubular injury and repair [43]. Therefore, measurement in renal tissue homogenates may have favored the detection of KIM-1-related changes over those of NGAL.

In line with our results, previous studies have demonstrated activation of MAPK signaling following renal injury [44]. Likewise, accumulating evidence indicates that p38 MAPK and JNK signaling are involved in the pathogenesis of AKI. A recent study reported that RI/RI significantly activated both the p38 MAPK and JNK signaling pathways, contributing to renal tubular injury [45]. Moreover, it has been proven that ischemia/reperfusion-induced AKI activates the p38 MAPK/ezrin signaling pathway, thereby promoting proximal tubular injury, which further supports the role of p38 MAPK in the pathogenesis of RI/RI [46]. In addition, the detrimental role of JNK in RI/RI has been previously confirmed, promoting proximal tubular inflammation, cell death, and the development of acute renal failure. Pharmacogenetic deletion of JNK1 attenuated tubular injury and improved renal function, highlighting the pivotal role of JNK signaling in AKI [47]. These findings support the involvement of MAPK signaling in RI/RI.

As far as we know, this is the first study that investigates the modulatory effect of LMW-DS on the expression of NGAL, KIM-1, p38 MAPK, and JNK. In the present study, LMW-DS attenuated the upregulation of inflammatory signaling and reduced the expression of p38 MAPK and JNK, which was associated with reduced levels of renal injury biomarkers, including KIM-1 and NGAL. Since JNK signaling can promote apoptotic and fibrogenic responses [48], the elevation in the immunoreaction of JNK induced by RI/RI functions as a marker of injury-associated stress signaling. The concomitant improvement in renal injury with reduced inflammation, NGAL, and KIM-1 following treatment with LMW-DS suggests that modulation of p38 MAPK/JNK signaling may be associated with the nephroprotective effects of LMW-DS. This work did not employ specific pathway inhibitors or gene knockdown; thus, these changes in p38 MAPK/JNK reflected a significant correlation rather than a definitive causal sequence. Additional functional experiments are necessary to determine whether modulation of MAPK/JNK signaling is directly responsible for the protective effects of LMW-DS.

Our data indicated strong caspase-3 immunoreactivity following RI/RI, reflecting apoptosis-associated responses. Caspase-3 is an important component of apoptotic signaling and has been implicated in tubular cell death and subsequent renal fibrosis following ischemia/reperfusion injury [49]. ATP depletion and calcium overload in the presence of tubular damage will result in disturbing the balance between pro-apoptotic and anti-apoptotic proteins, causing apoptosis [29]. Our findings confirmed the attenuation of caspase-3 expression could be involved in the protective effect of LMW-DS against RI/RI. This reducing effect together with the improvement in renal histopathology and biochemical indices of injury, is consistent with a reduction in apoptosis-associated responses. This effect may be attributed to LMW-DS’s potential to modulate inflammatory and oxidative pathways and limit cellular injury. Interestingly, the effect of LMW-DS on the expression of caspase-3 has not been previously assessed in the literature.

Histopathological examination demonstrated significant renal injury following RI/RI, which was characterized by interstitial inflammation, tubular degeneration, and glomerular shrinkage. On the other hand, the RI/RI+LMW-DS group showed an amelioration of the histopathological alterations compared to the RI/RI group. This improvement was confirmed by reduction in vascular congestion and hemorrhage, preservation of tubular architecture, and partial restoration of glomerular morphology. Our observations are consistent with the documented structural alterations associated with RI/RI [29,50]. These studies demonstrated that RI/RI caused tubular epithelial damage and inflammation, which progressed to fibrosis. Likewise, Masson’s trichrome staining revealed increased collagen deposition in the RI/RI group, indicating early extracellular matrix accumulation and fibrotic changes following ischemic injury [51]. These alterations may be driven by profibrotic mediators such as MCP-1 and TGF-β1 [52]. Notably, LMW-DS significantly reduced collagen deposition, supporting its potential antifibrotic effect, and aligned with a previous study, which showed that modulation of MCP-1 and TGF-β1 pathways reduced inflammation-driven fibrosis [52].

Taken together, the results of this study revealed the nephroprotective activity of LMW-DS against RI/RI. LMW-DS showed antioxidant activity by increasing the GSH level and SOD activity while reducing the MDA level. Additionally, LMW-DS successfully reduced the expression levels of TNF-α, IL-6, TLR-4, KIM-1, NGAL, JNK/p38 MAPK, and caspase-3. Lastly, the histopathological data revealed a reduction in the morphological alterations induced by RI/R after LMW-DS administration, providing evidence for the nephroprotective effects of LMW-DS in the RI/RI rat model.

## 5. Conclusions

AKI occurs after ischemia/reperfusion and results from an active process involving oxidative stress, inflammation, apoptosis-associated responses, and modulation in MAPK/JNK-associated signaling. Oxidative stress appears to be an essential factor that induces inflammation, which in turn induces apoptotic responses during RI/RI. Due to the complexity of ischemia/reperfusion-induced acute renal failure, further experiments are needed to enhance our understanding of its pathophysiological mechanisms and the treatment interventions that could be used to mitigate its consequences.

### Study Limitations

The current study provided strong evidence supporting the use of a single injection of LMW-DS as a protective drug to partially overcome the pathological alterations induced by RI/RI. Nevertheless, the present findings have several limitations, including the short evaluation period and the use of a single-dose treatment protocol. Therefore, future studies should investigate the long-term effect of LMW-DS on renal function. Likewise, different doses and treatment schedules should be evaluated to establish the optimal therapeutic regimen. Furthermore, we only used male rats to avoid any variation induced by the estrous cycle of female rats. Additionally, clinical studies are warranted to confirm the translational potential and therapeutic benefits of LMW-DS in kidney transplant patients.

An important limitation of the present study is that the molecular signaling analyses were based on immunohistochemical assessment of total p38 MAPK, JNK, and caspase-3. Total protein immunoreactivity does not directly establish kinase activation or caspase-3 activation. Furthermore, no pharmacological or genetic pathway manipulation was undertaken to establish a causal role for MAPK/JNK signaling in the nephroprotective effect of LMW-DS. Therefore, the present findings should be interpreted as evidence of an association between attenuation of these signaling-associated markers and renal protection rather than definitive evidence of a causal MAPK/JNK/caspase-3 mechanism. Future studies should incorporate p-p38/p38 and p-JNK/JNK measurements, cleaved caspase-3, complementary apoptotic markers (such as TUNEL assay, Bax/Bcl-2), and direct pathway manipulation to establish the mechanistic contribution of these pathways more definitively.

## Figures and Tables

**Figure 1:**
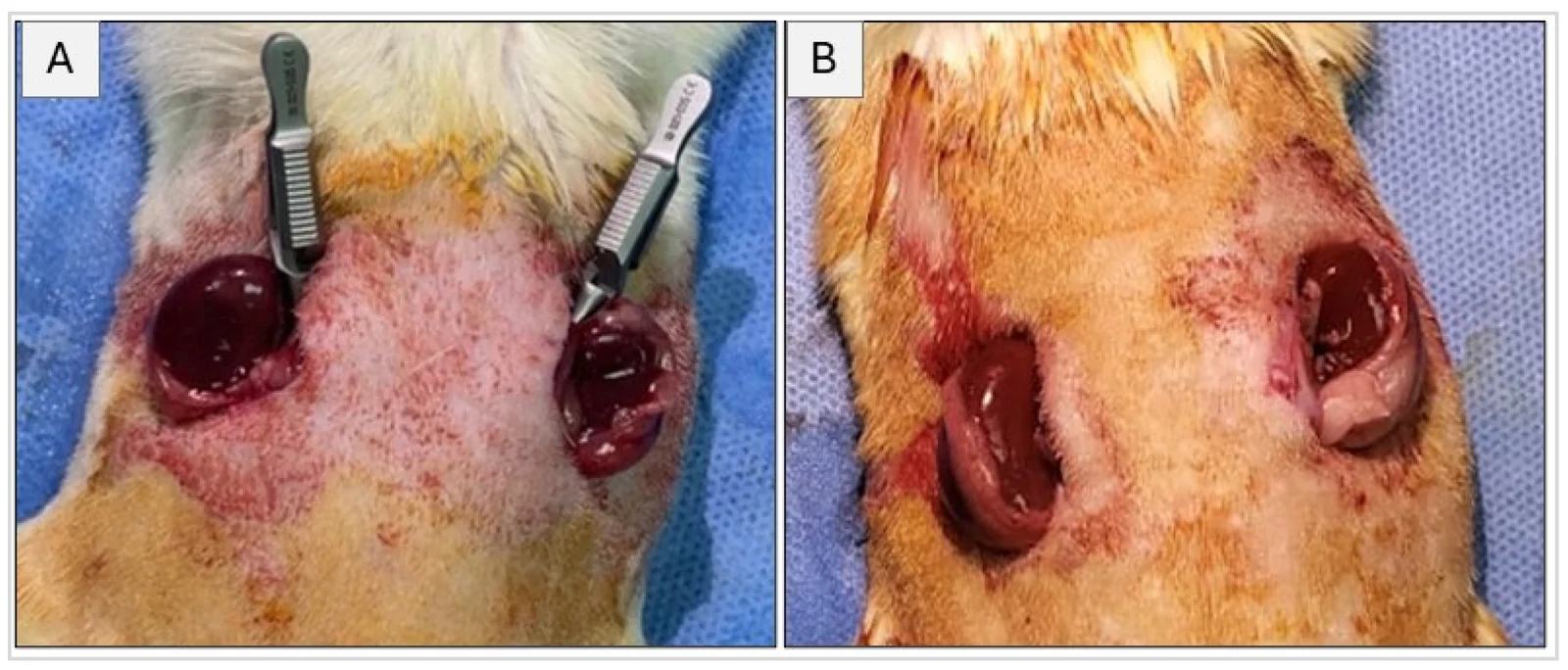
(**A**) Renal hilum clamping (Ischemic phase), (**B**) unclamping (Reperfusion phase).

**Figure 2:**
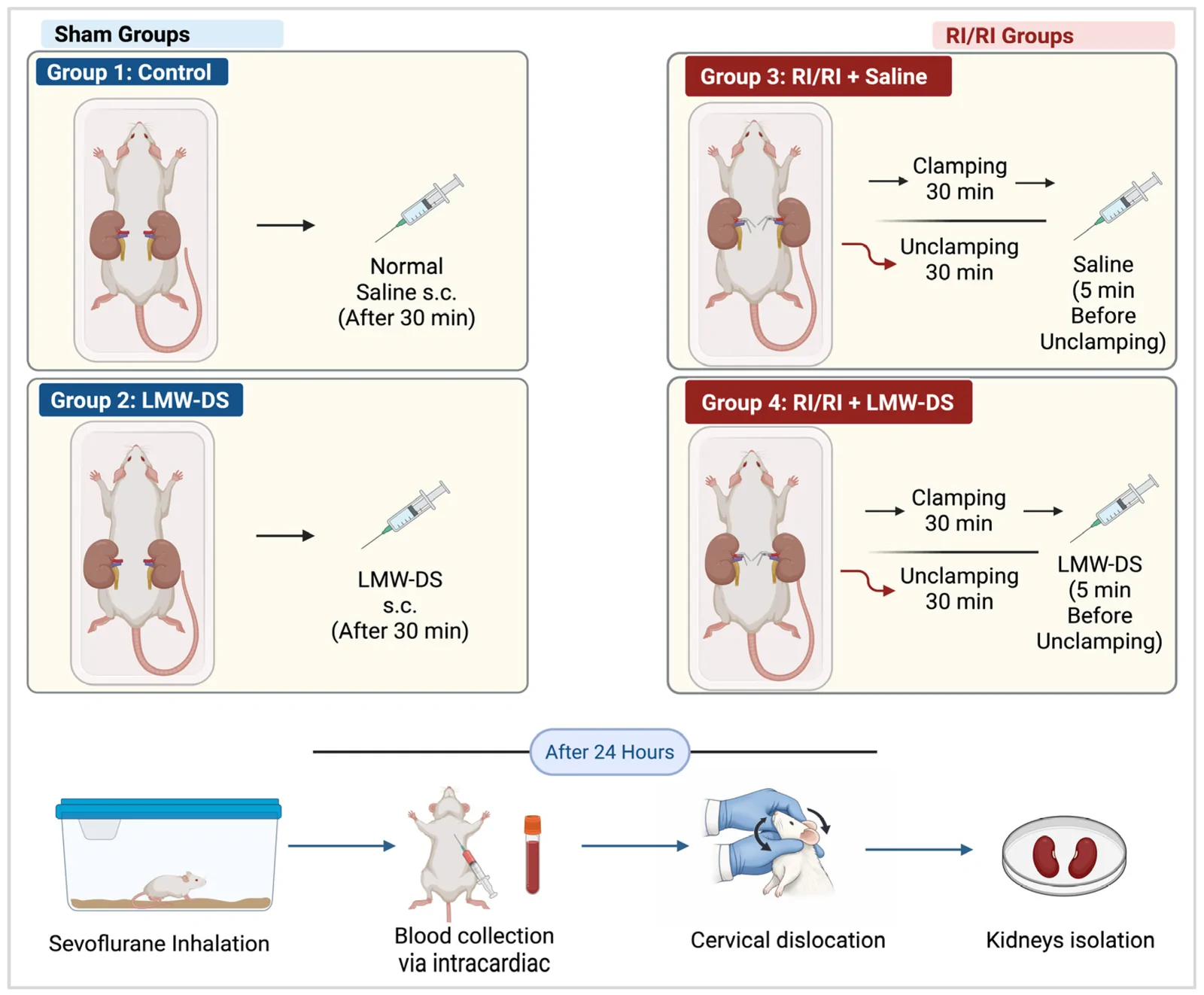
Drug Administration and sample collection (created by BioRender).

**Figure 3:**
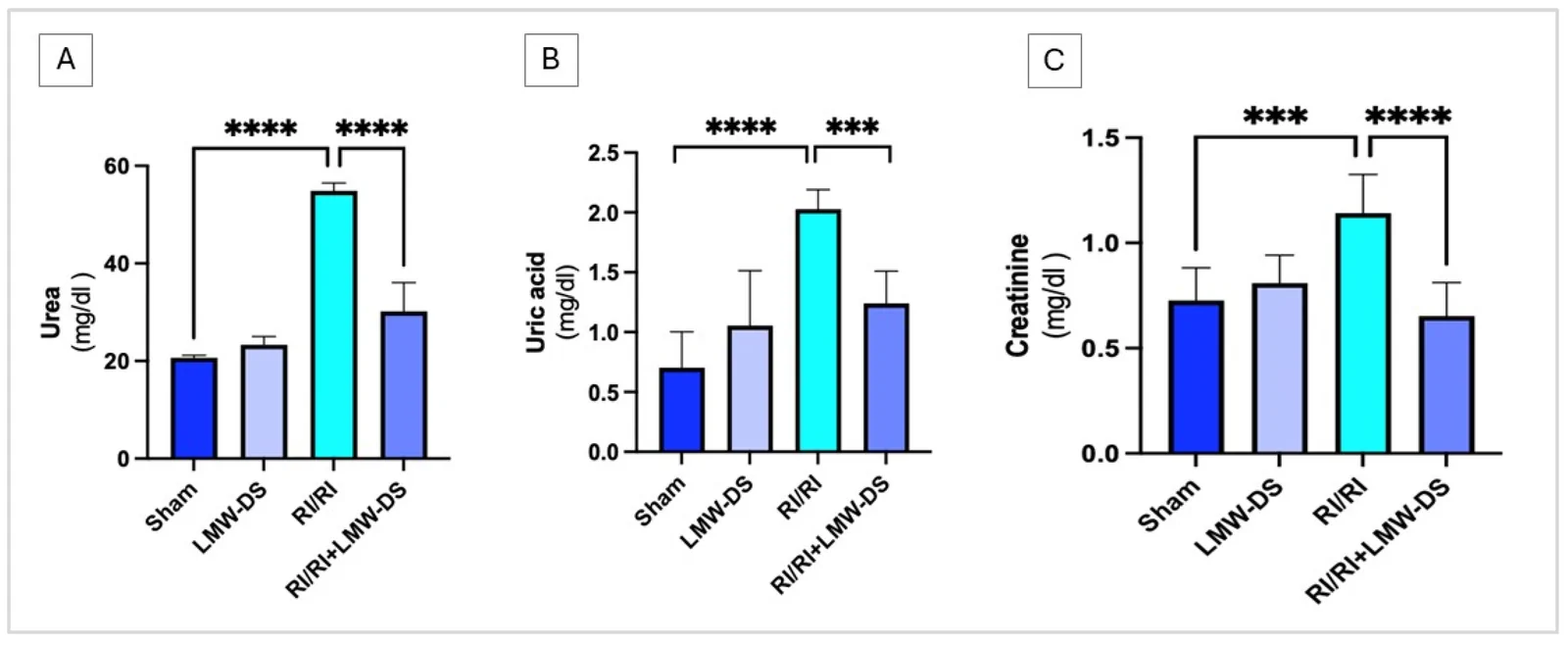
**Effect of LMW-DS on the serum levels of renal function parameters following RI/RI.** (**A**) Urea, (**B**) Uric acid, and (**C**) Creatinine. Data are presented as mean ± SEM (*n* = 8 per group). Statistical analysis was performed using one-way ANOVA followed by Tukey’s post hoc test. *** *p* < 0.001, **** *p* < 0.0001.

**Figure 4:**
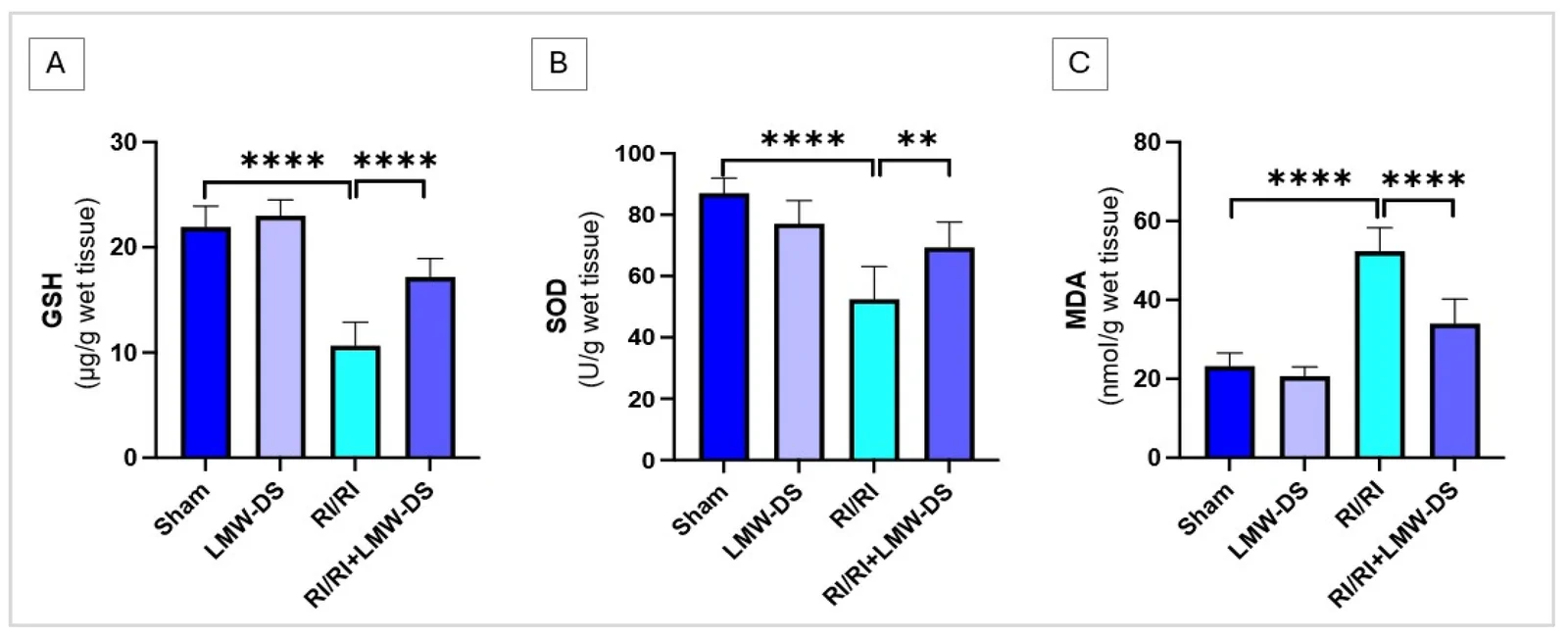
**Effect of LMW-DS on the levels of oxidative stress biomarkers in kidney tissues following RI/RI**. (**A**) Glutathione (GSH), (**B**) Superoxide dismutase (SOD), and (**C**) malondialdehyde (MDA). Data are presented as mean ± SEM (*n* = 8). Statistical analysis was performed using one-way ANOVA followed by Tukey’s post hoc test. ** *p* < 0.01, **** *p* < 0.0001.

**Figure 5:**
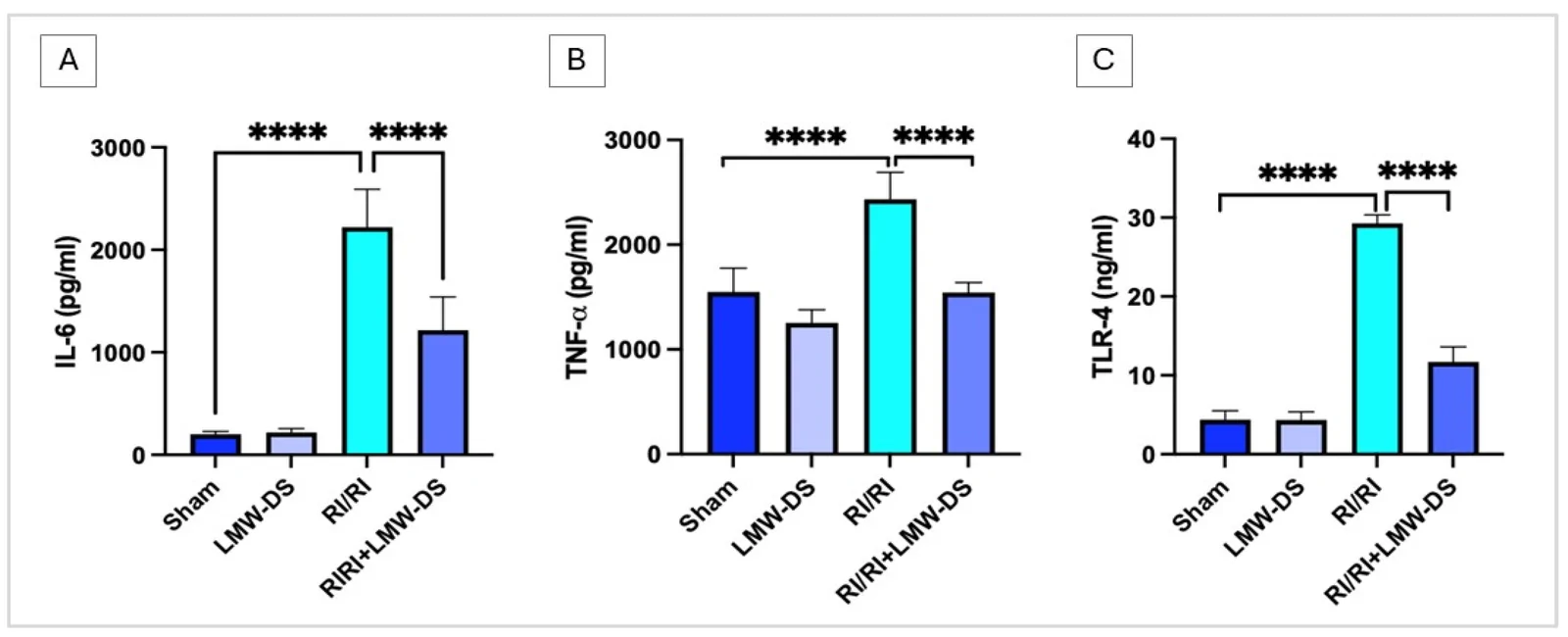
**Effect of LMW-DS on the levels of inflammatory biomarkers in kidney tissues following RI/RI.** (**A**) Interleukin-6 (IL-6), (**B**) Tumor necrosis factor-alpha (TNF-α), and (**C**) Toll-like receptor-4 (TLR-4). Data are presented as mean ± SEM (*n* = 8). Statistical analysis was performed using one-way ANOVA followed by Tukey’s post hoc test. **** *p* < 0.0001.

**Figure 6:**
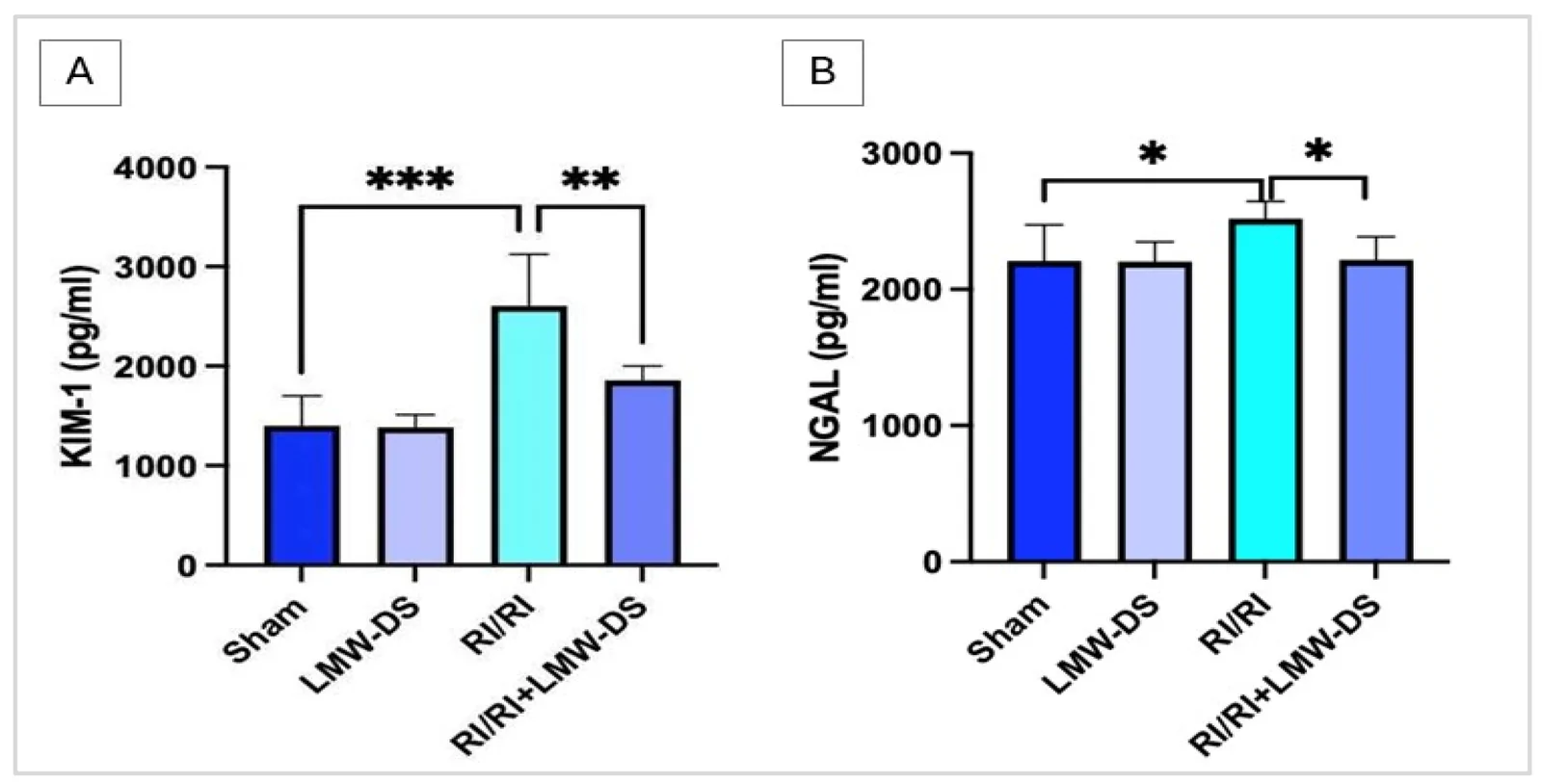
**Effect of LMW-DS on the levels of renal injury biomarkers in kidney tissues following RI/RI.** (**A**) Kidney injury molecule-1 (KIM-1) and (**B**) Neutrophil gelatinase-associated lipocalin (NGAL). Data are presented as mean ± SEM (*n* = 8). Statistical analysis was performed using one-way ANOVA followed by Tukey’s post hoc test. *** *p* < 0.001, ** *p* < 0.01, * *p* < 0.05.

**Figure 7:**
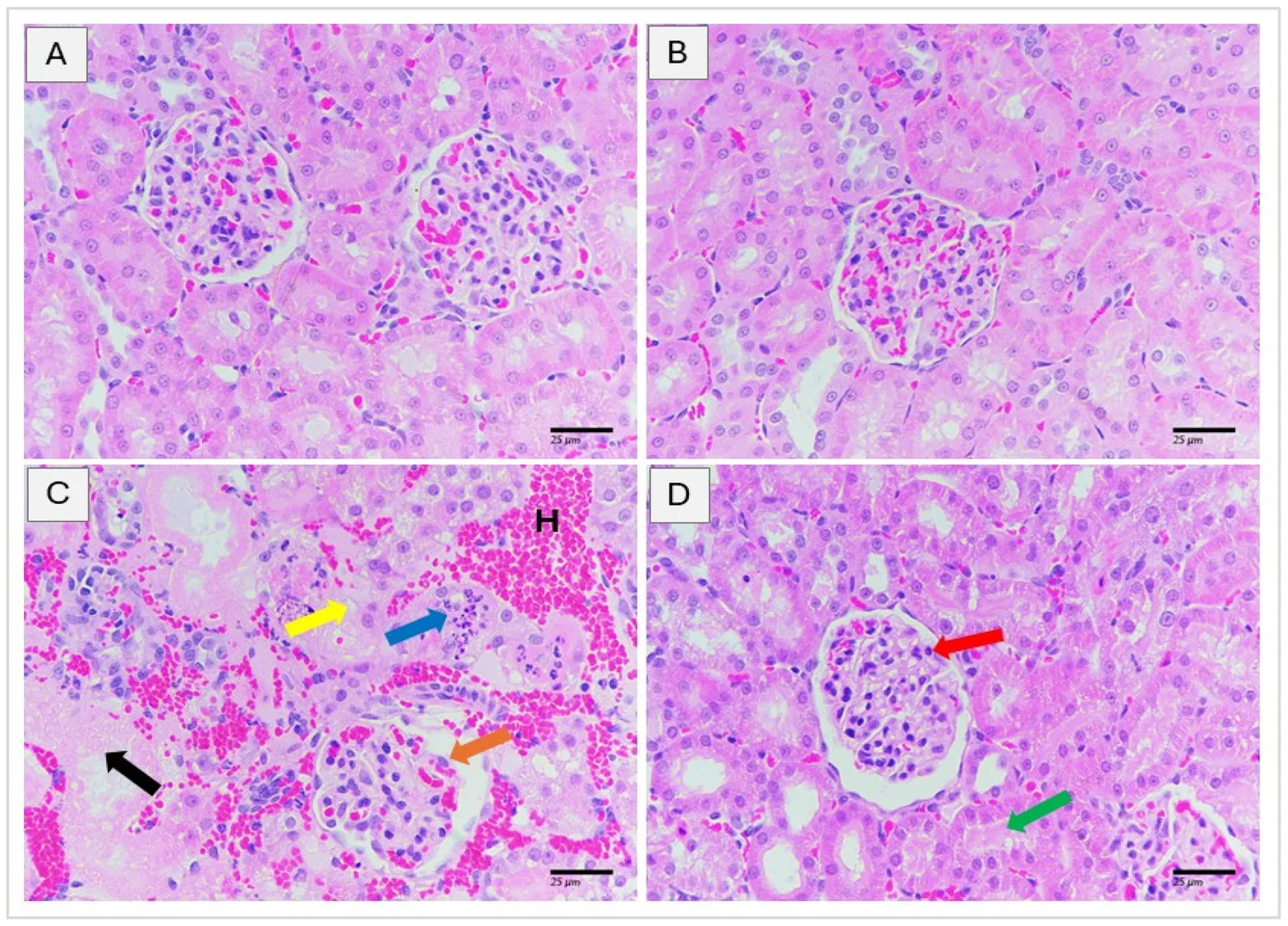
**Photomicrographs of representative kidney sections stained with H&E at 400× magnification.** (**A**) **Sham group** exhibits normal renal architecture with intact glomeruli and well-organized renal tubule. (**B**) Kidney section from **LMW-DS group** shows normal renal structure with no pathological alterations, (**C**) **RI/RI group** representative section displays severe pathological changes, congestion with haemorrhage (H), tubular degeneration (black arrow), interstitial inflammation (blue arrow), cellular degeneration (yellow arrow), and minimized glomeruli (orange arrow). (**D**) **RI/RI+LMW-DS group** shows marked improvement, with a tubular cast (green arrow) and a glomerulus with foam cells (red arrow). Scale bar = 25 μm.

**Figure 8:**
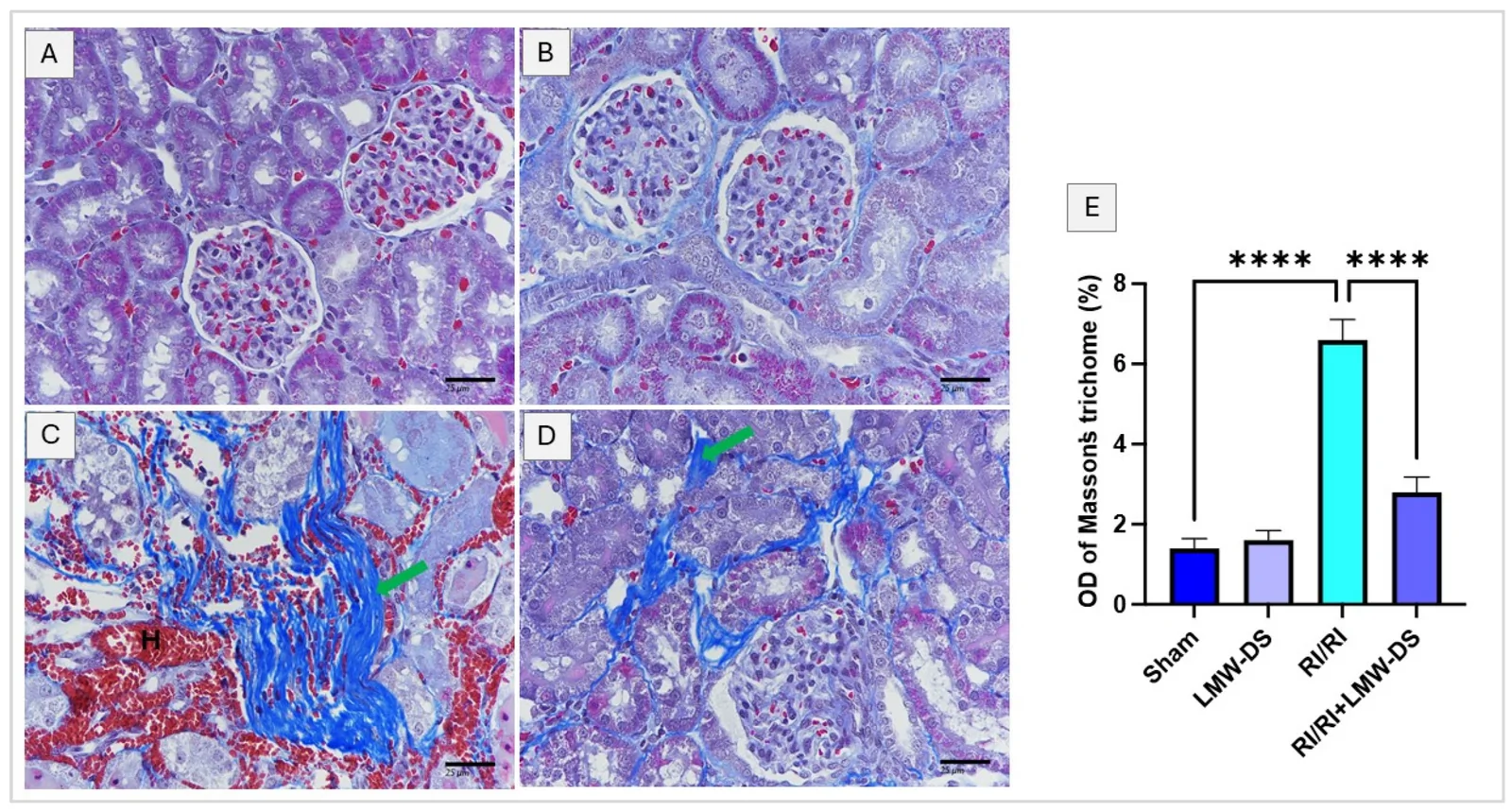
**Photomicrographs of representative kidney sections stained with Masson’s trichrome at 400× magnification.** Representative sections from both (**A**) **Sham and** (**B**) **LMW-DS groups** show low levels of collagen deposition. (**C**) **RI/RI group** section displays severe renal lesions including marked accumulation of collagen (green arrow) and vascular congestion (H). (**D**) **RI/RI+LMW-DS group** section shows a marked reduction in collagen deposition (green arrow). Scale bar = 25 μm. (**E**) Quantitative analysis of Masson’s trichrome staining in kidney. Optical density percentage (OD%) of Masson’s trichrome staining, indicating the relative collagen deposition intensity. Data are presented as mean ± SEM (*n* = 8). Statistical analysis was performed using one-way ANOVA followed by Tukey’s post hoc test. **** *p* < 0.0001.

**Figure 9:**
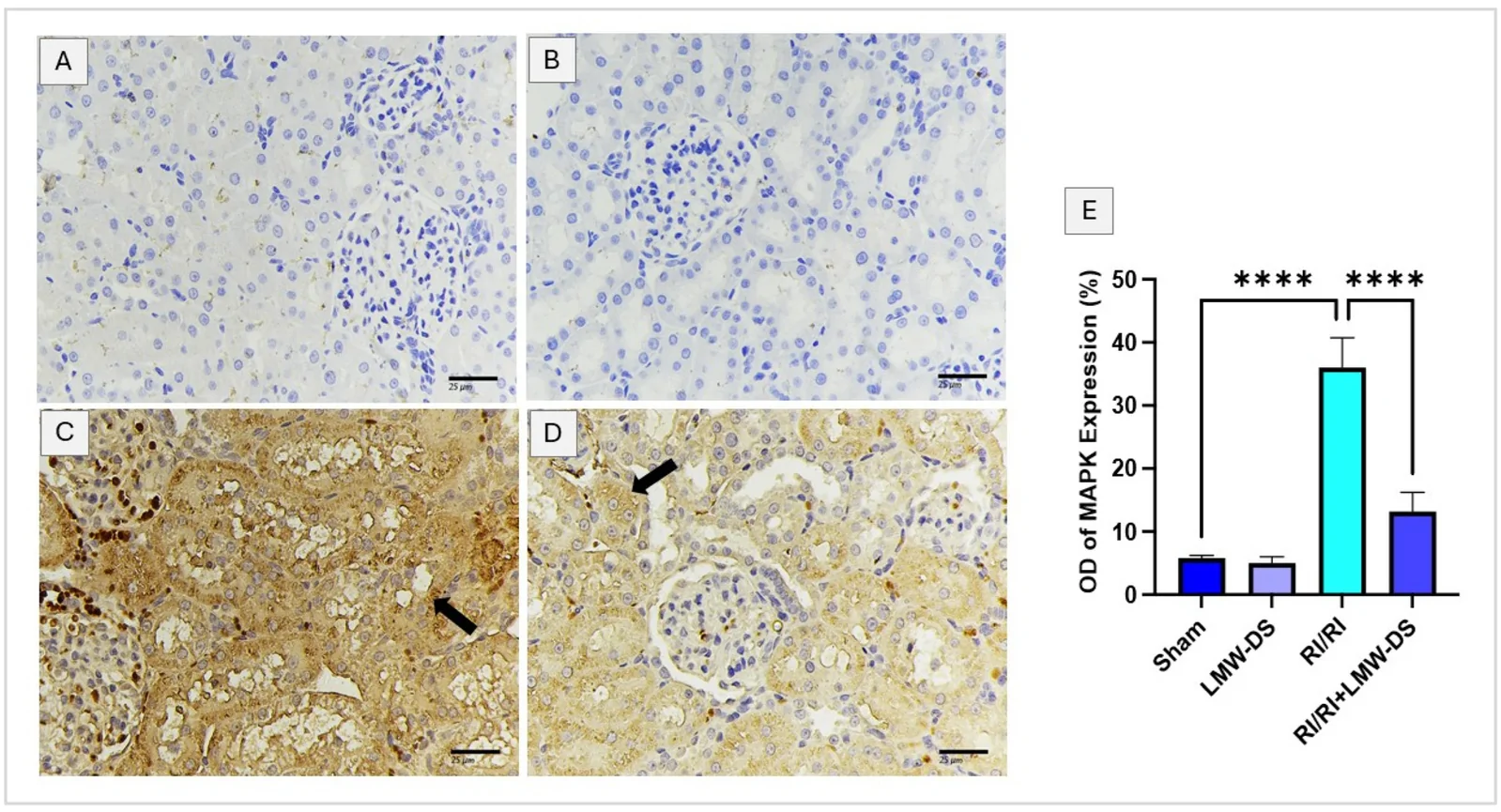
**Immunohistochemical evaluation of p38 Mitogen-Activated Protein Kinase (p38 MAPK) expression in renal tissues at 400× magnification.** Representative sections from both (**A**) **Sham and** (**B**) **LMW-DS groups** show normal renal histological architecture with very low p38MAPK immunoreactivity. (**C**) A representative section from **RI/RI group** exhibits strong immune reaction for p38 MAPK, characterized by intense brown cytoplasmic staining in renal tubular epithelial cells with evident degenerative alterations (arrow). (**D**) Kidney section from **RI/RI+LMW-DS group** displays a downregulation of p38 MAPK expression in renal tubules, with scattered positive immunostaining within tubular epithelial cells (arrow). Scale bar = 25 μm. (**E**) Quantitative analysis of P38 MAPK immunohistochemical expression in renal tissue. Relative optical density (OD %) of p38 MAPK expression in renal tissue, indicating the intensity of immunohistochemical staining. Data are presented as mean ± SEM (*n* = 8). Statistical analysis was performed using one-way ANOVA followed by Tukey’s post hoc test. **** *p* < 0.0001.

**Figure 10:**
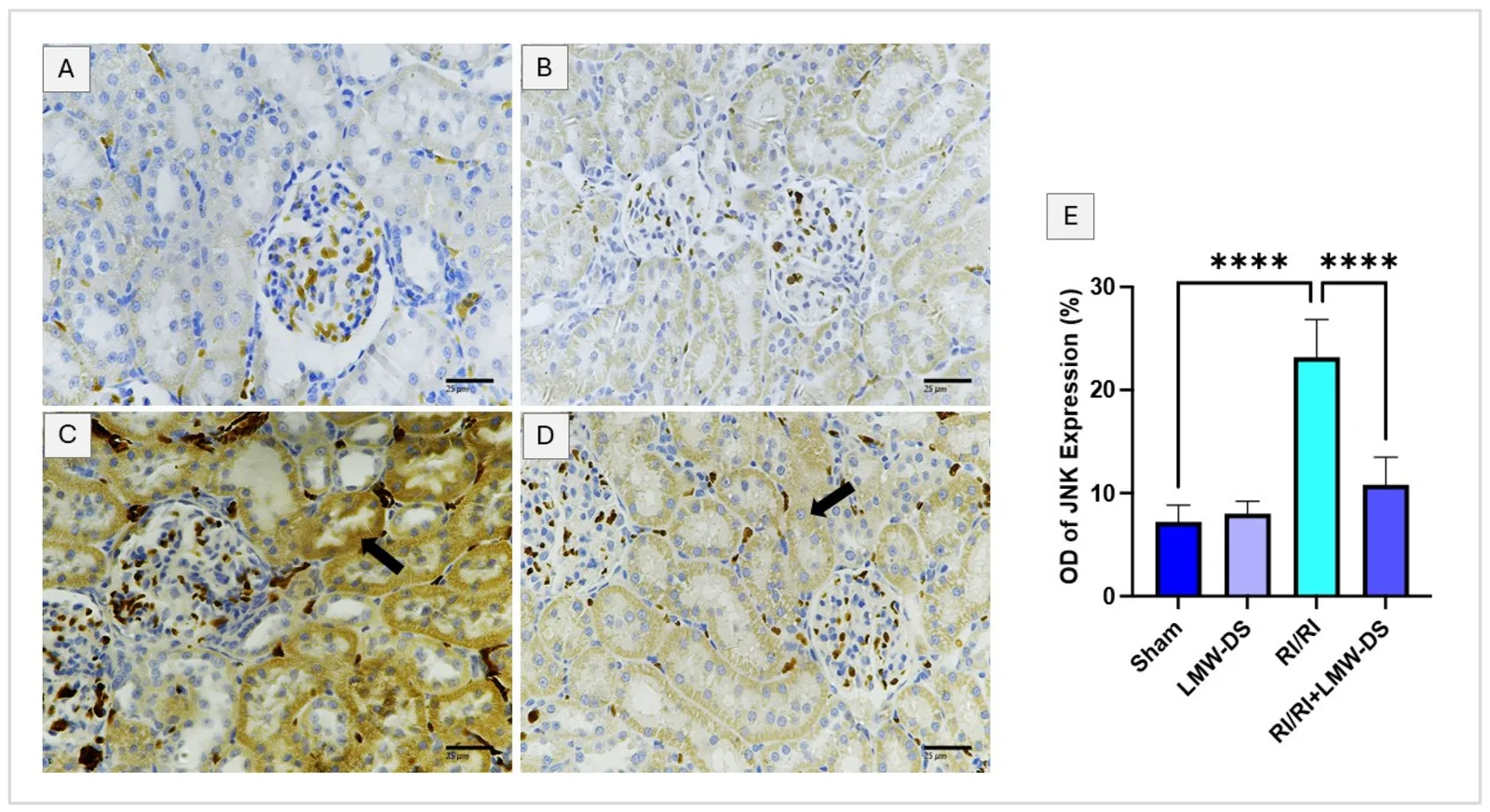
**Immunohistochemical detection of c-Jun N-terminal kinase (JNK) expression in renal tissues at 400× magnification.** Representative sections from both (**A**) **Sham and** (**B**) **LMW-DS groups** show normal renal histological architecture with weak JNK immunoreactivity. (**C**) A representative section from **RI/RI group** exhibits strong and widespread JNK immune response with intense brown cytoplasmic localization in renal tubular epithelial cells (arrow) and increased glomerular reactivity. (**D**) Kidney section from **RI/RI+LMW-DS group** displays a moderate JNK expression in renal tubular (arrow) and glomerular cells. Scale bar = 25 μm. (**E**) Quantitative analysis of JNK immunohistochemical expression in renal tissue. Relative optical density (OD %) of JNK expression in renal tissue, indicating the intensity of immunohistochemical staining. Data are presented as mean ± SEM (*n* = 8). Statistical analysis was performed using one-way ANOVA followed by Tukey’s post hoc test. **** *p* < 0.0001.

**Figure 11:**
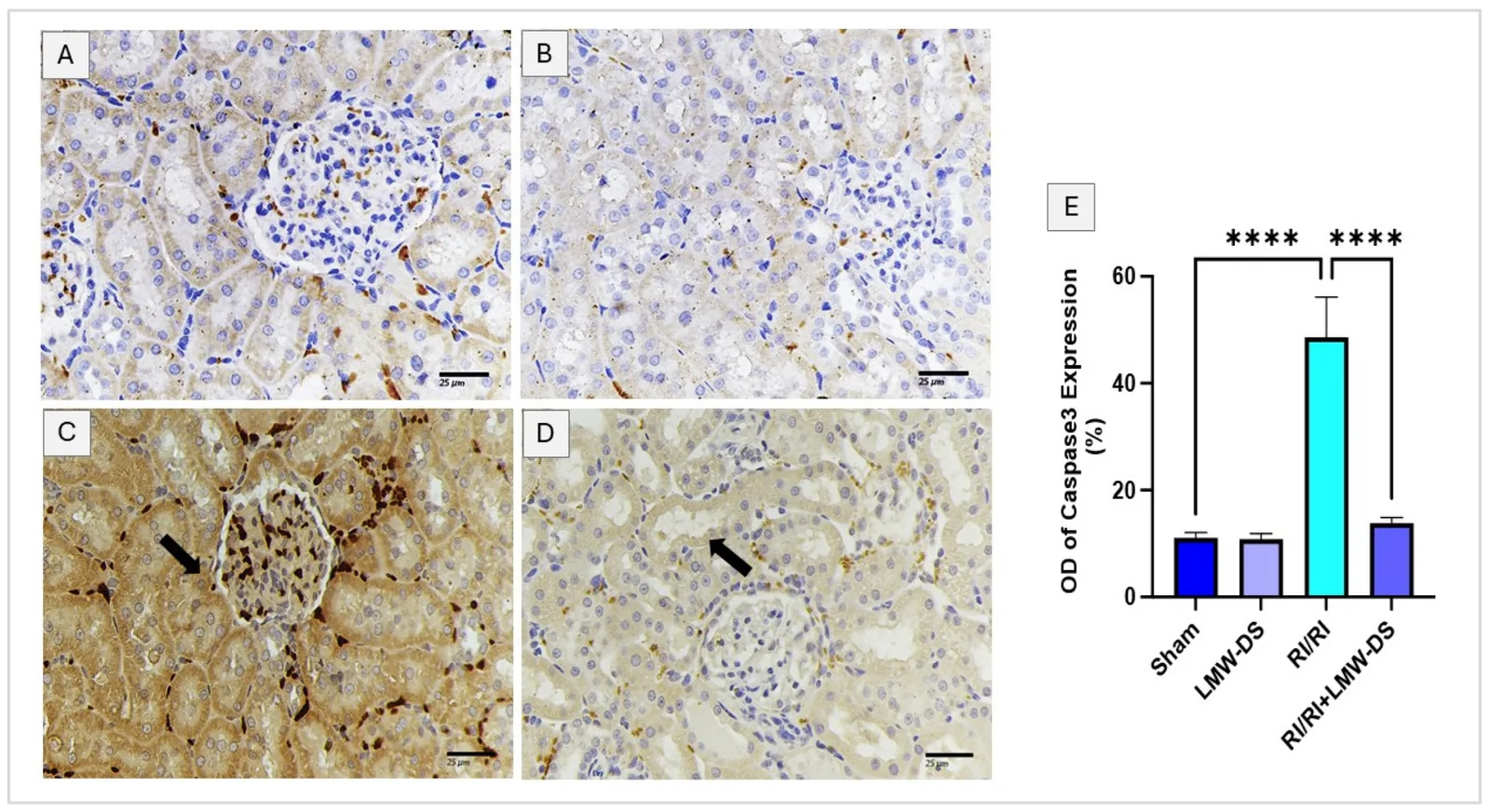
**Immunohistochemical detection of caspase-3 expression in renal tissues at 400× magnification.** Representative sections from both (**A**) **Sham and** (**B**) **LMW-DS groups** show normal renal histological architecture with minimal caspase-3 immunoreactivity. (**C**) A representative section from **RI/RI group** exhibits a strong and widespread caspase-3 immune response (arrow). (**D**) Kidney section from **RI/RI+LMW-DS group** displays a downregulation of caspase-3 expression in renal tubular (arrow) and glomerular cells. Scale bar = 25 μm. (**E**) Quantitative analysis of caspase-3 immunohistochemical expression in renal tissue. Relative optical density (OD %) of caspase-3 expression in renal tissue, indicating the intensity of immunohistochemical staining. Data are presented as mean ± SEM (*n* = 8). Statistical analysis was performed using one-way ANOVA followed by Tukey’s post hoc test. **** *p* < 0.0001.

**Table 1:** **Kidney injury scoring of morphological alterations among all experimental groups. Scoring scale:** 0 = non, 1 = Minimal damage (<10% of tissue affected), 2 = Mild damage (10–25%), 3 = Moderate damage (26–50%), 4 = Severe damage (51–75%), 5 = Very severe damage (>75%).

Groups	Glomerular Atrophy	Tubular Degeneration	Interstitial Inflammation	Hemorrhage	Collagen Deposition	Casts
**Sham**	**0.0 ± 0.0**	**0.0 ± 0.0**	**0.0 ± 0.0**	**0.0 ± 0.0**	**0.0 ± 0.0**	**0.0 ± 0.0**
**LMW-DS**	**0.0 ± 0.0**	**0.0 ± 0.0**	**0.0 ± 0.0**	**0.0 ± 0.0**	**0.0 ± 0.0**	**0.0 ± 0.0**
**RI/RI**	2.71 ± 0.09 ****	3.67 ± 0.1 ****	3.7 ± 0.09 ****	4.7 ± 0.08 ****	3.60 ± 0.1 ****	3.2 ± 0.09 ****
**RI/RI+LMW-DS**	**2.25 ± 0.09 ^#^**	**2.3 ± 0.1 ^##^**	**2.0 ± 0.11 ^##^**	**1.2 ± 0.1 ^##^**	**2.9 ± 0.1 ^#^**	**2.9 ± 0.1 ^#^**

Data are presented as mean ± SEM (3 fields/section/rat; 24 fields/group). Statistical analysis was performed using an unpaired *t*-test with Welch’s correction; equal standard deviations were not assumed, with statistical significance considered between sham group vs. RI/RI group (**** *p* < 0.0001) and RI/RI group vs. RI/RI+LMW-DS group (^#^ *p* < 0.05, ^##^ *p* < 0.01).

## Data Availability

The original contributions presented in this study are included in the article and Supplementary Materials. The raw data supporting the conclusions of this article will be made available by the authors on request.

## Supplementary Materials

The following supporting information can be downloaded at: https://www.mdpi.com/article/10.3390/antiox15101238/s1.
