# Supplementary material for: Low Molecular Weight Dextran Sulfate Mitigates Acute Kidney Injury Induced by Renal Ischemia/Reperfusion: Implication of JNK/p38 MAPK and Caspase-3
Source: Antioxidants (Basel). 2026 Sep 25;15(10):1238. doi: 10.3390/antiox15101238 (PMC13700585; doi:10.3390/antiox15101238)
Supplement: Supplementary file 1 [file antioxidants-15-01238-s001.zip › antioxidants-4484771-supplementary.pdf]

Supplementary Materials

Chemical Name	Catalogue/REF No.	Source
LMW-DS (5KD) Batch no. 21205	Cat#DB005	Ultuna, Sweden
Caspase-3 Polyclonal Antibody, anti-rabbit IgG RRID: AB_304243	Cat# ab4051	Abcam, United Kingdom
P38 MAPK Polyclonal Antibody, anti-rabbit IgG RRDI: AB_330713	Cat# 9212	Cell signaling technology, USA
JNK1/JNK2 Monoclonal Antibody, anti-mouse, clone 279Q38, RRID: AB_2536335	Cat# AHO1362	Invitrogen, USA
Anti-mouse IgG (H+L) Secondary Antibody RRID: AB_228364	REF: 31181	Invitrogen, USA
Goat anti-rabbit IgG (H+L) Secondary Antibody RRDI: AB_228340	REF:31820	Invitrogen, USA
Uric Acid Liquicolor	REF:10690	Human, Germany
Urea Liquicolor	REF: 10505	Human, Germany
Creatinine Liquicolor	REF: 10052	Human, Germany
SOD Activity Assay Kit	Cat# BC0175	Solarbio, China
GSH Content Assay Kit	Cat# BC1175	Solarbio, China
MDA Content Assay Kit	Cat# BC0025	Solarbio, China
Rat TLR4 ELISA Kit	Cat# SEKR-0184	Solarbio, China
Rat NGAL/Lipocalin-2 ELISA Kit	Cat# SEKR-0028	Solarbio, China
Rat TIM-1/KIM-1/HAVCR ELISA Kit	Cat# SEKR-0145	Solarbio, China
Rat IL-6 ELISA Kit	Cat# SEKR-0005	Solarbio, China
Rat TNF-α ELISA Kit	Cat# SEKR-0009	Solarbio, China

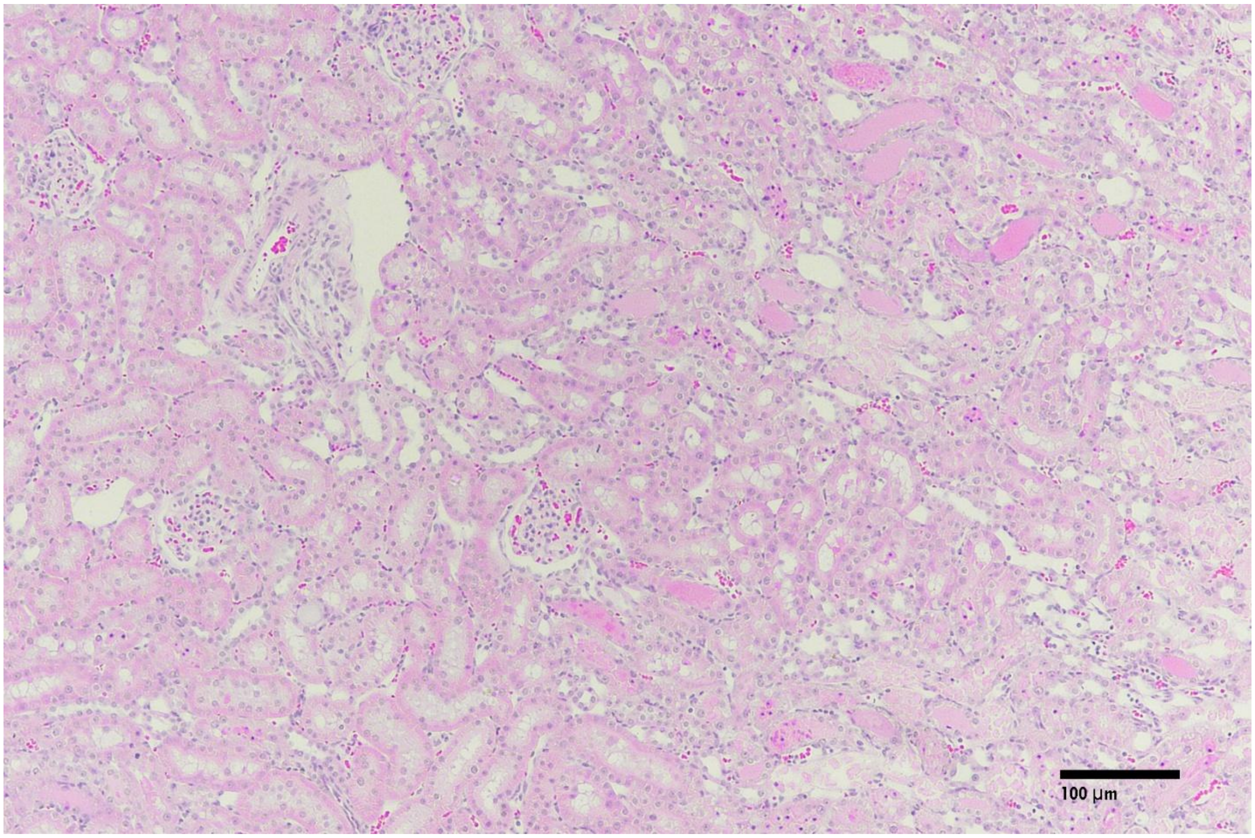
100 µm

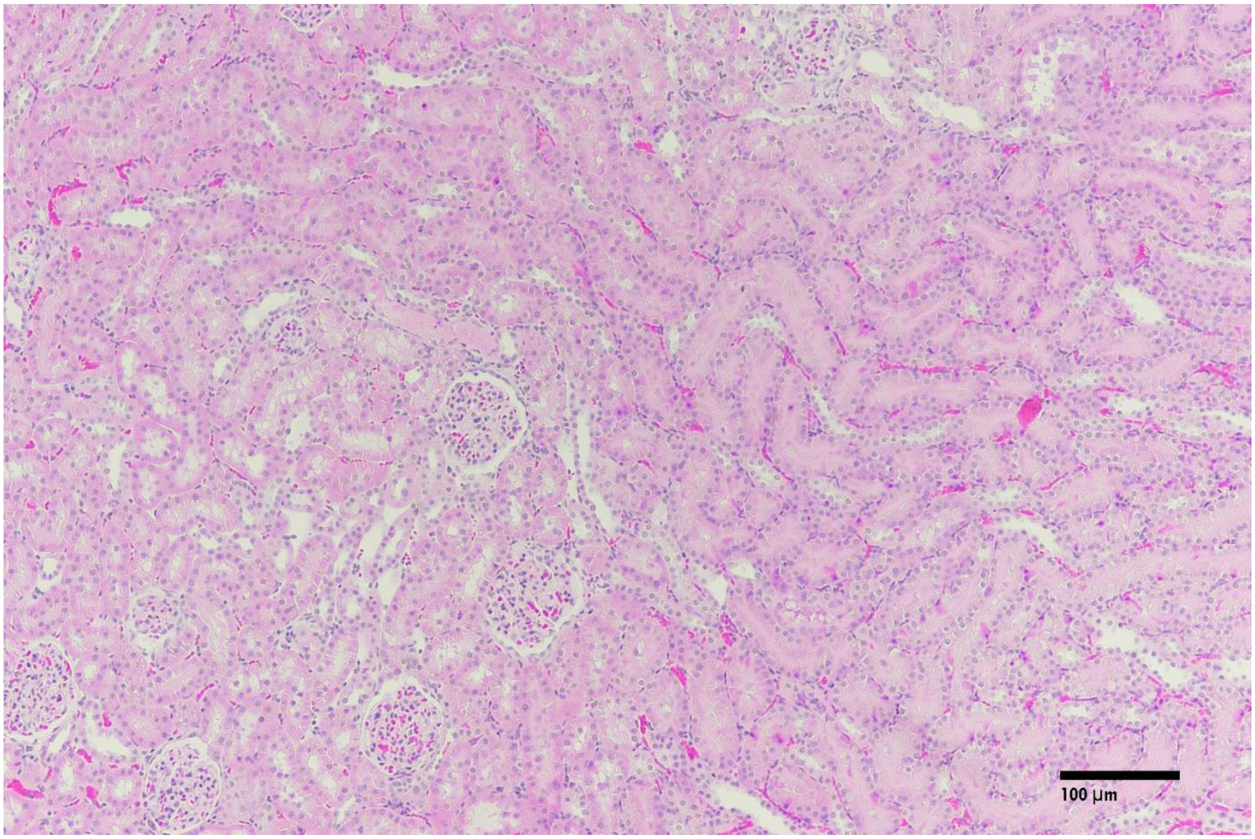
100 µm

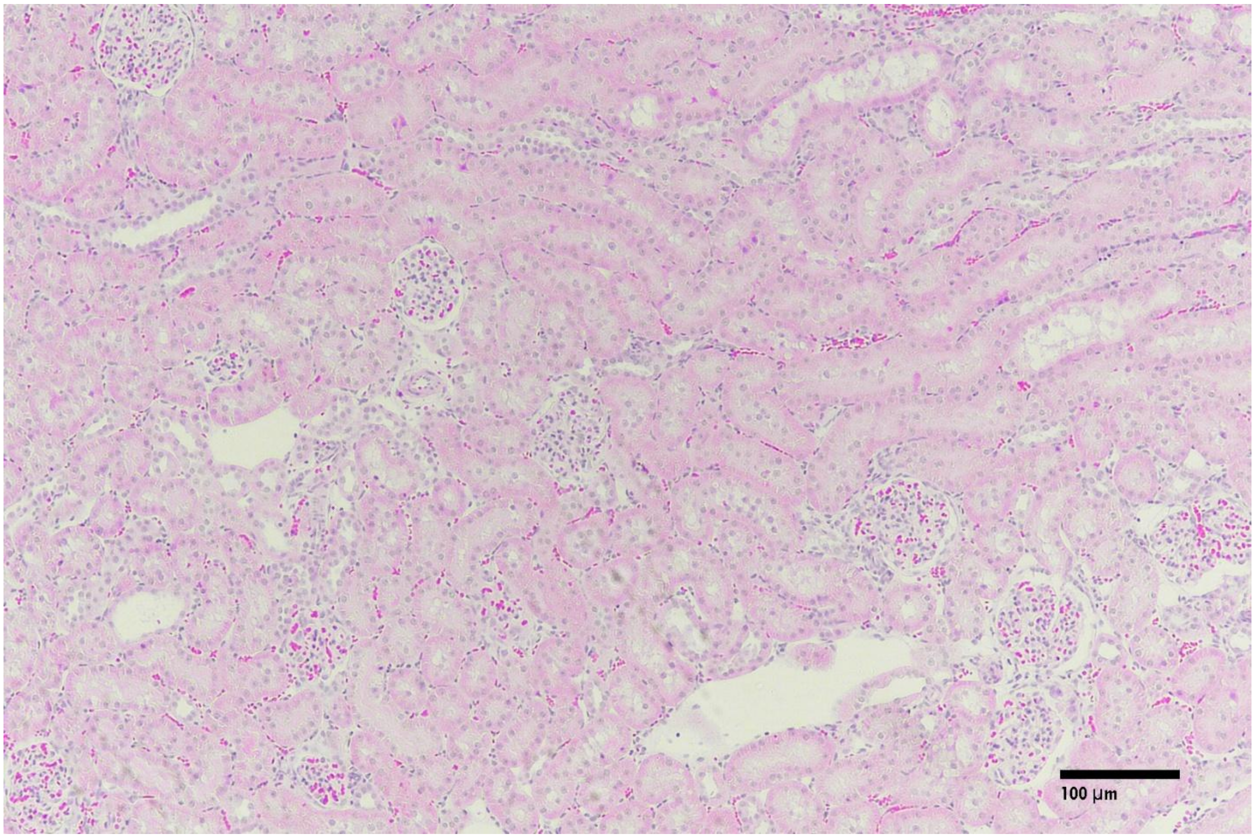
100 µm

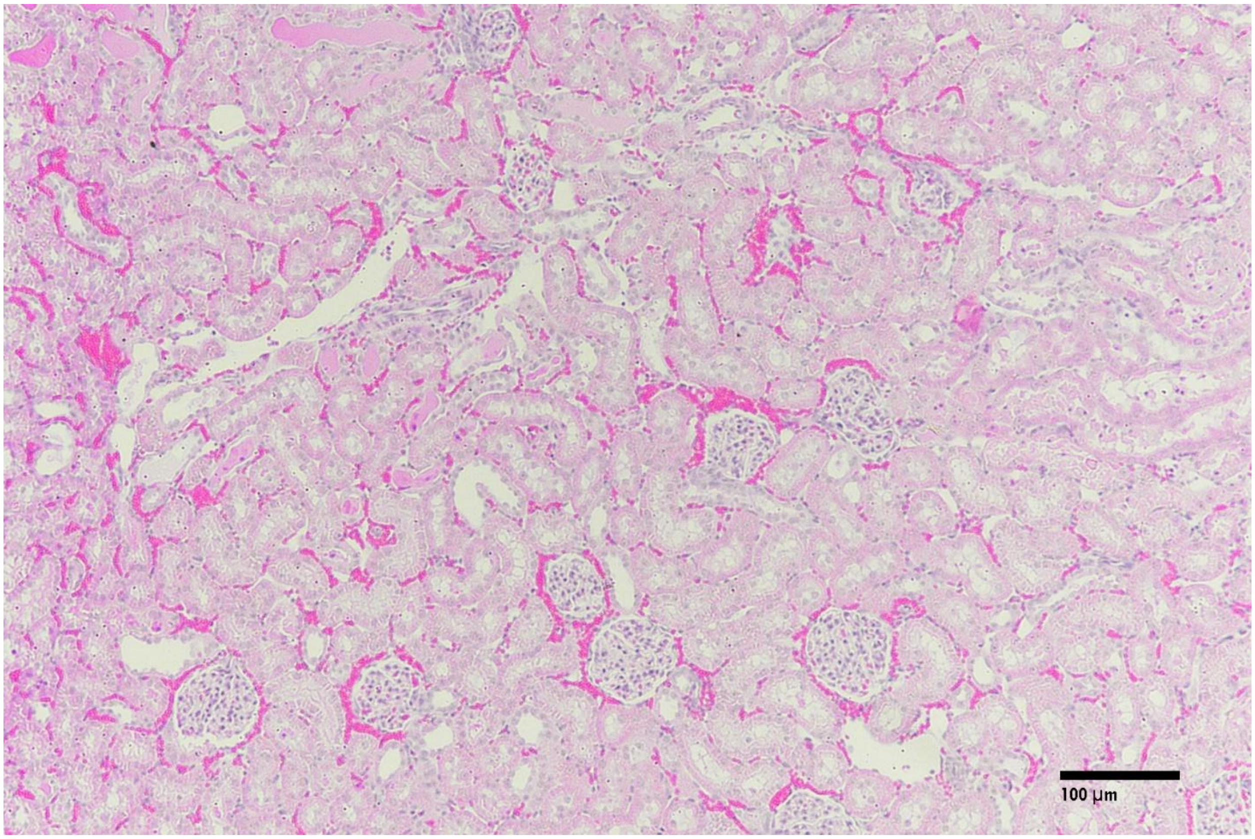
100 µm

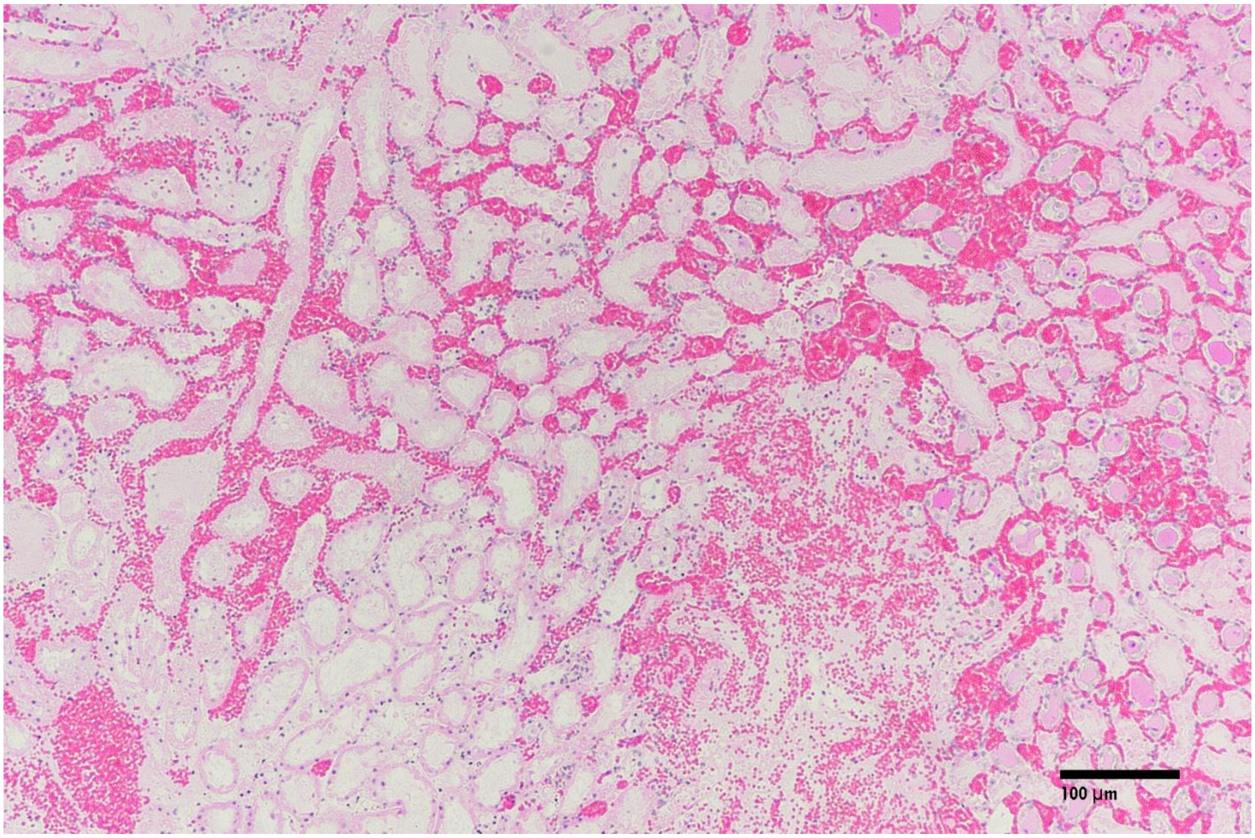
100 µm

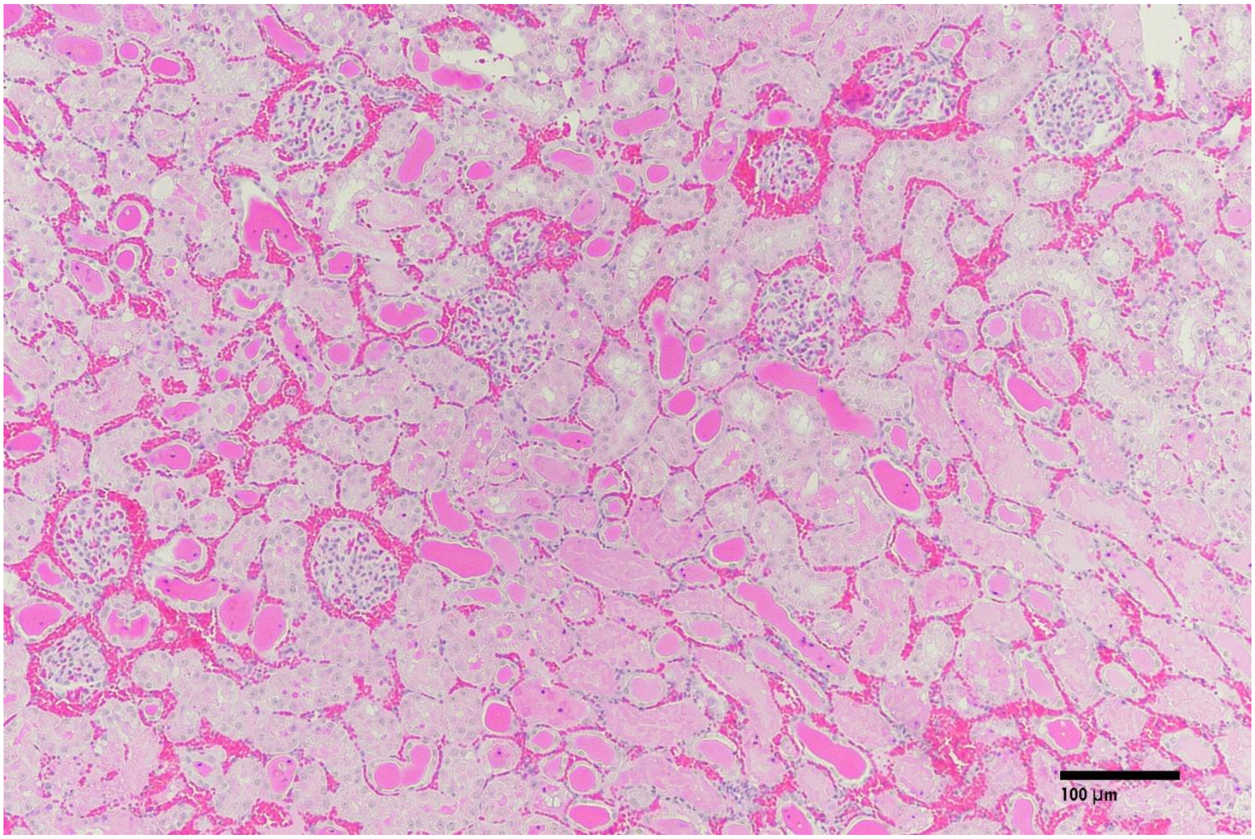
100 µm

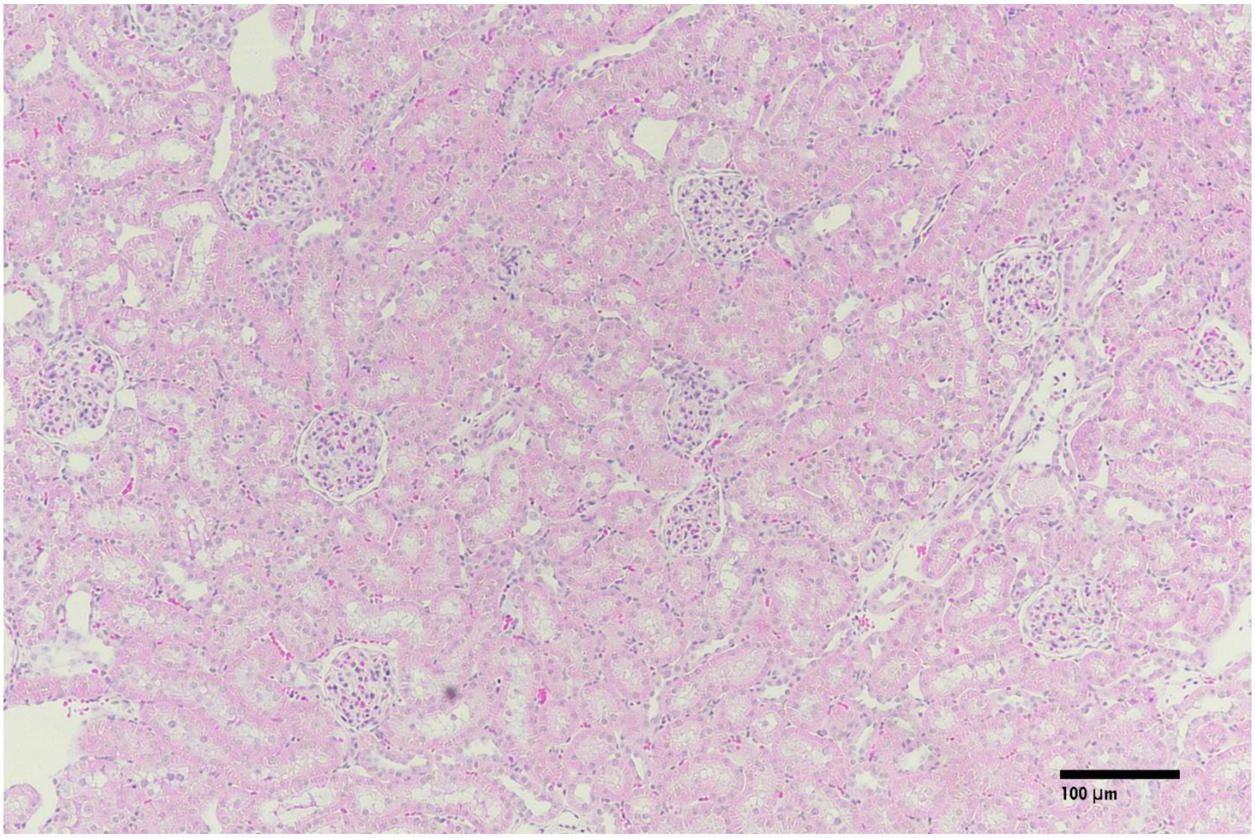
100 µm

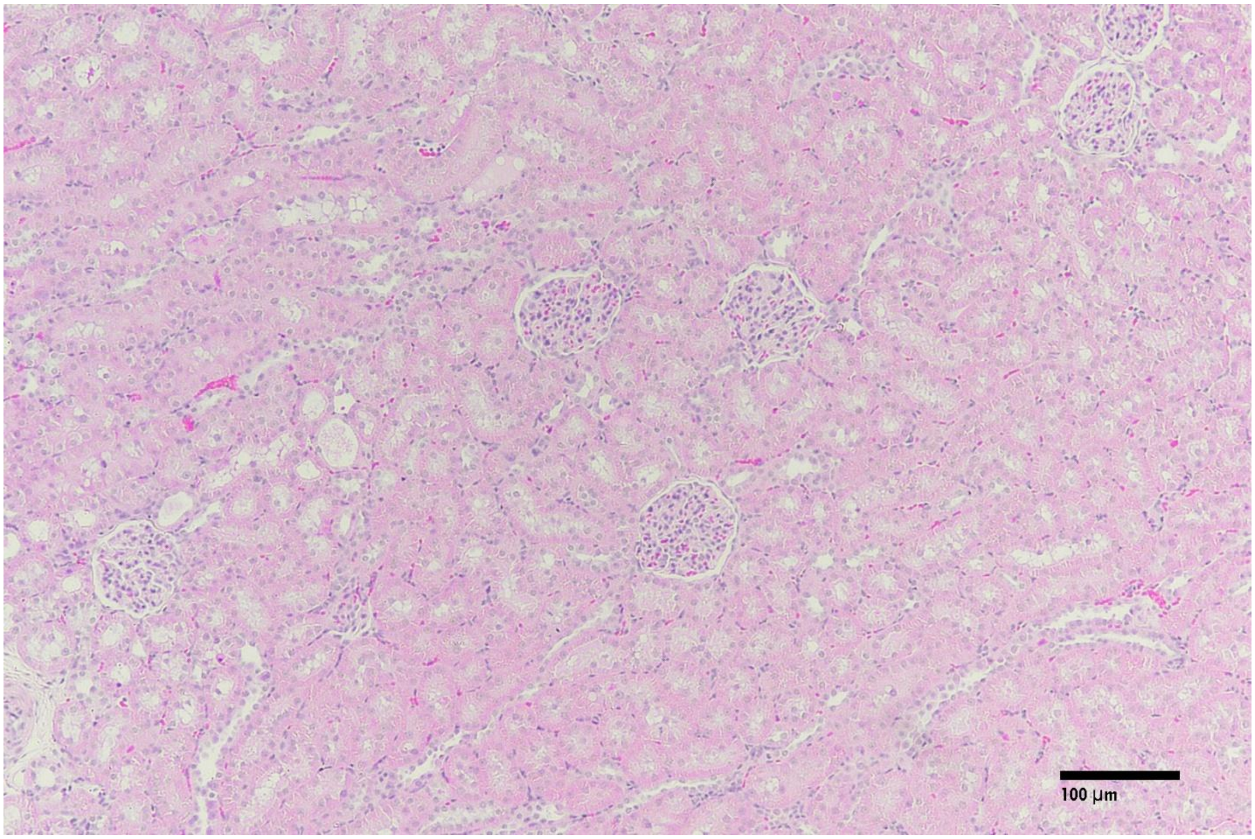
100 µm

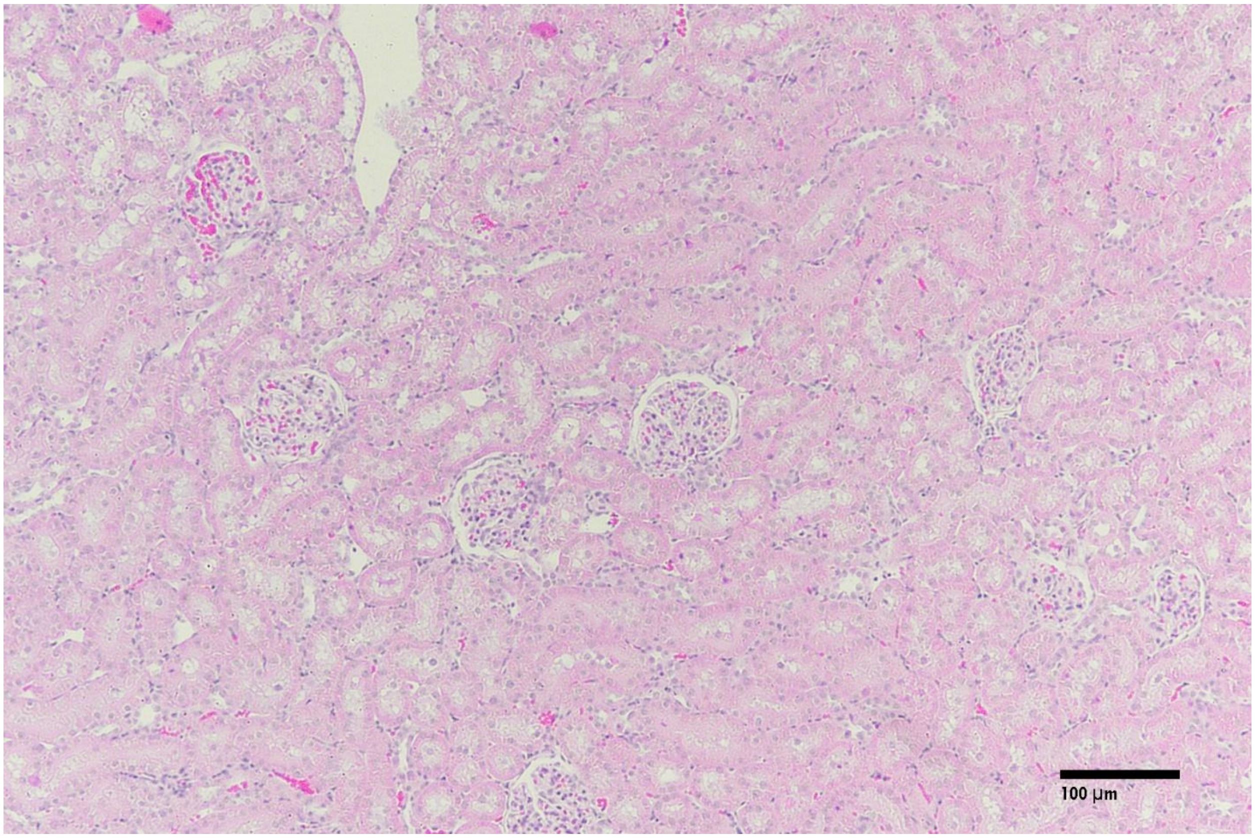
100 µm

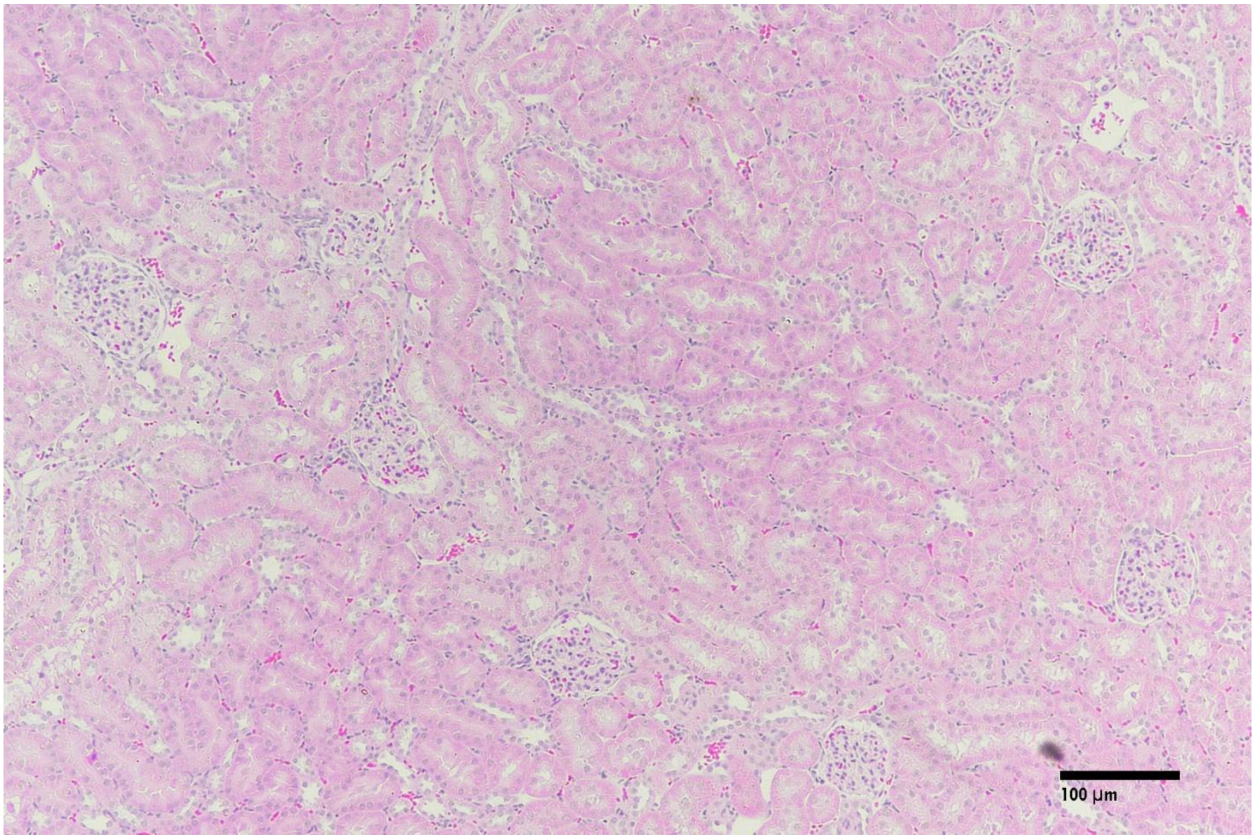
100 µm

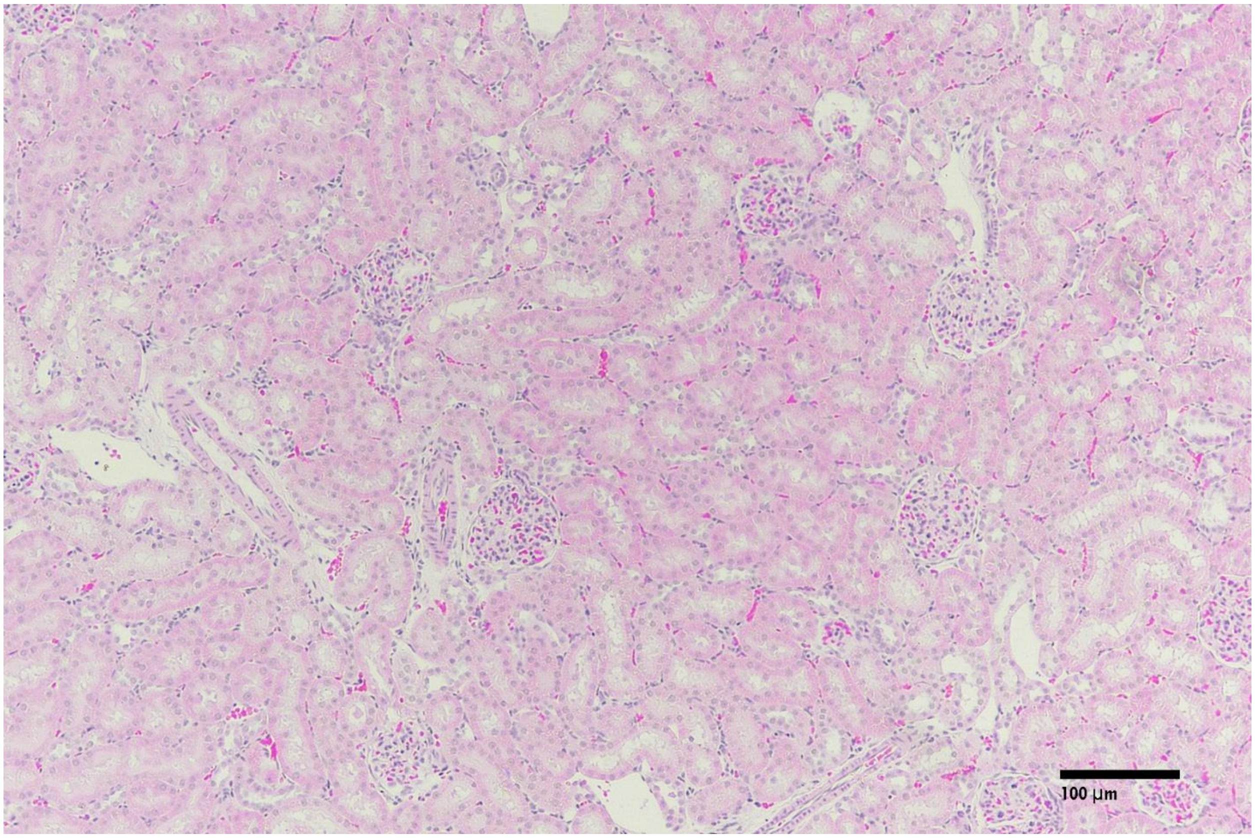
100 µm

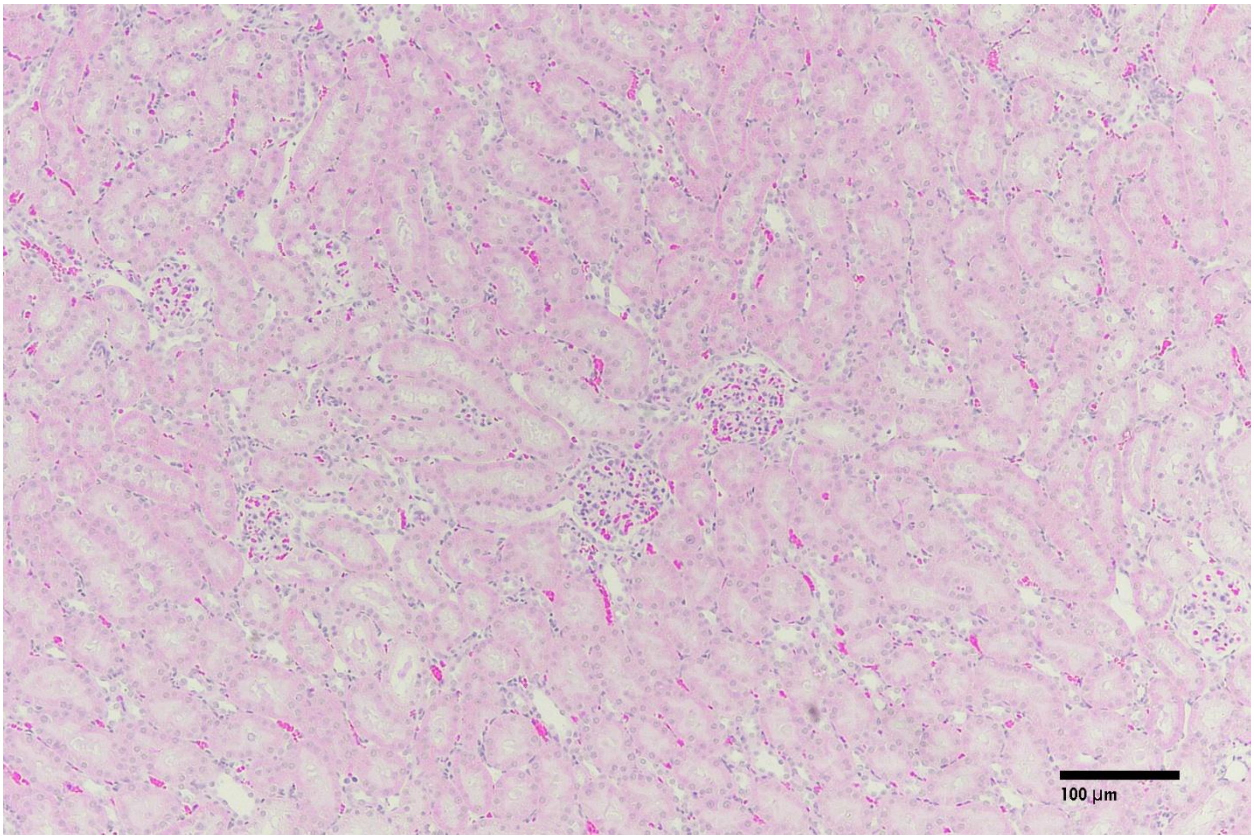
100 µm

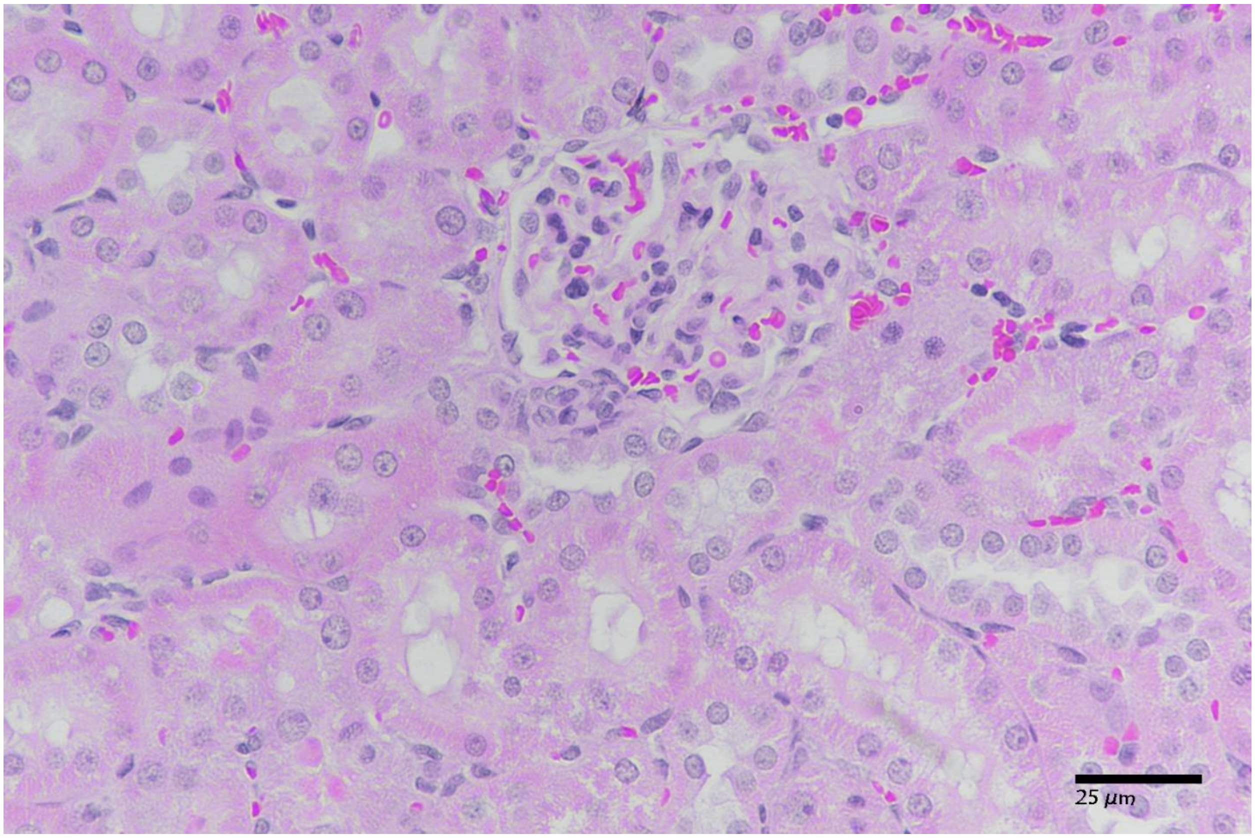
25 μm

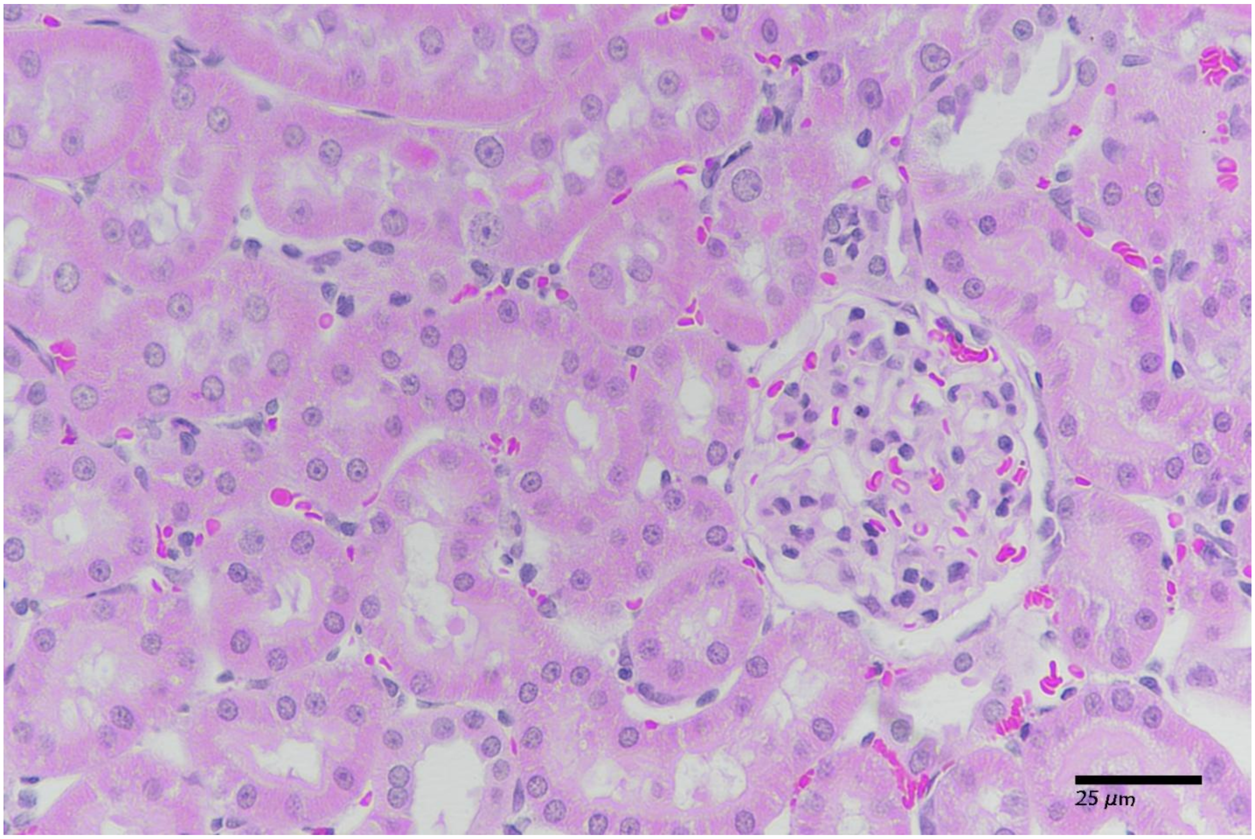
25 μm

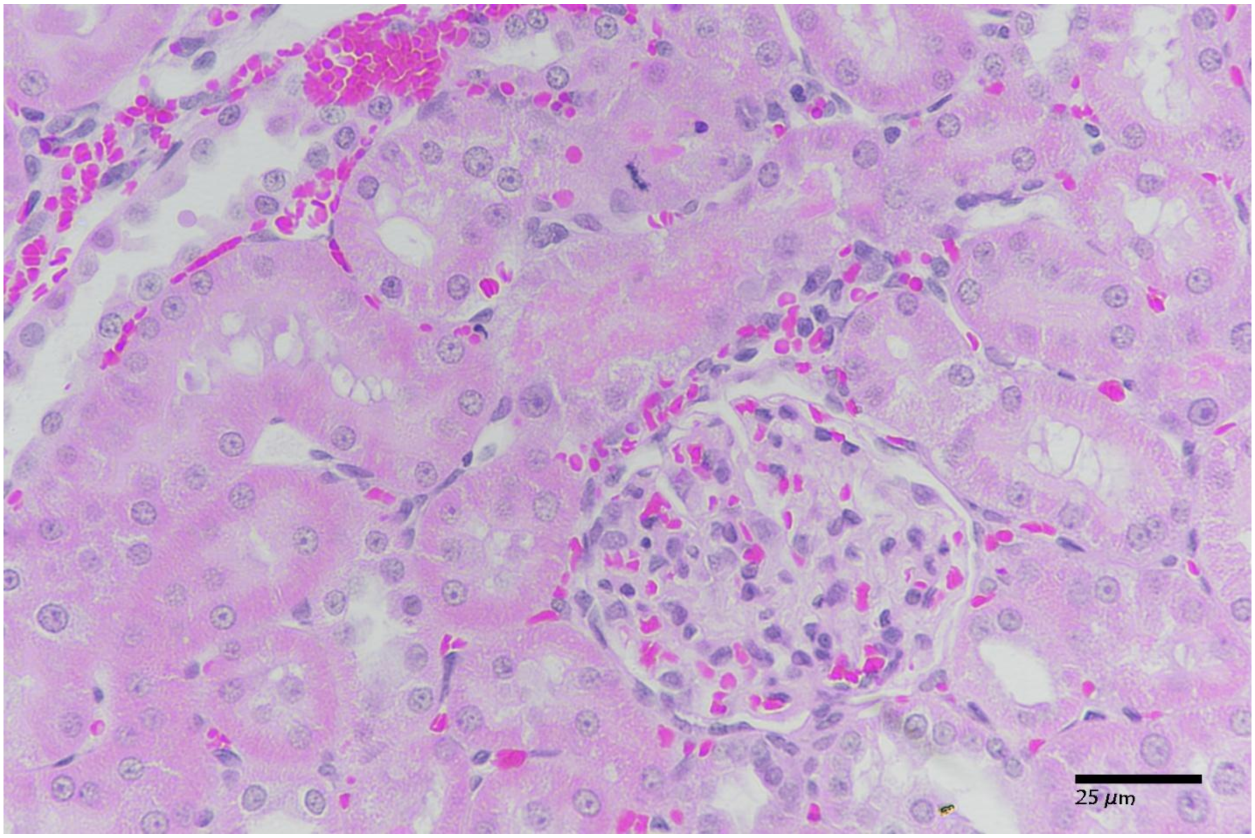
25 μm

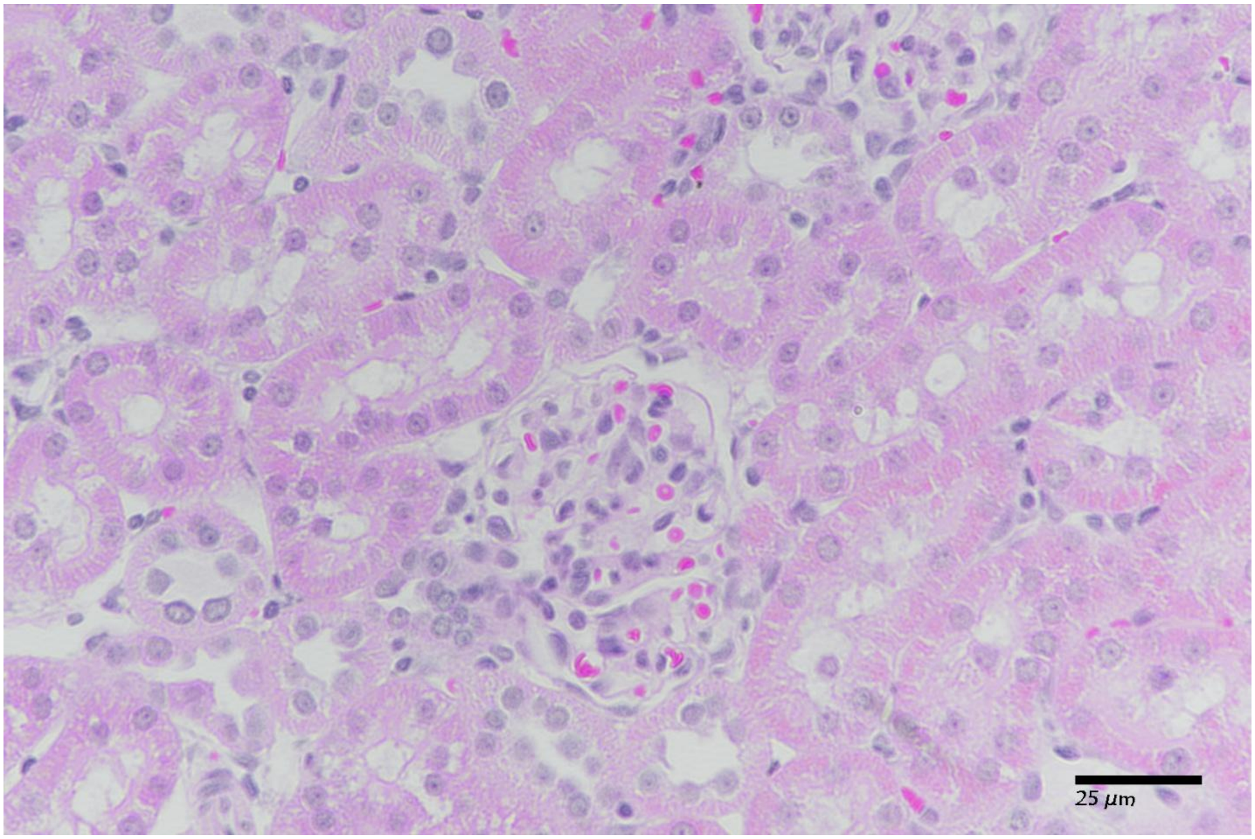
25 μm

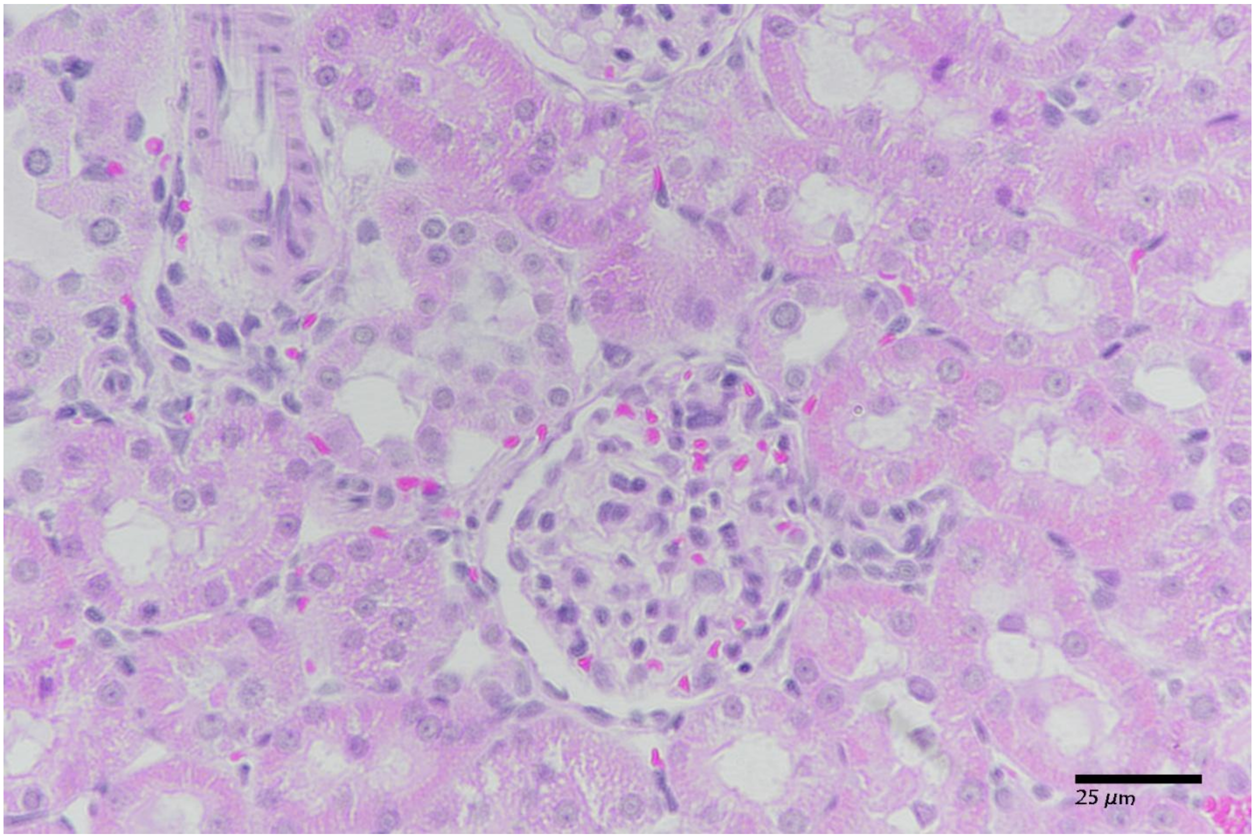
25 μm

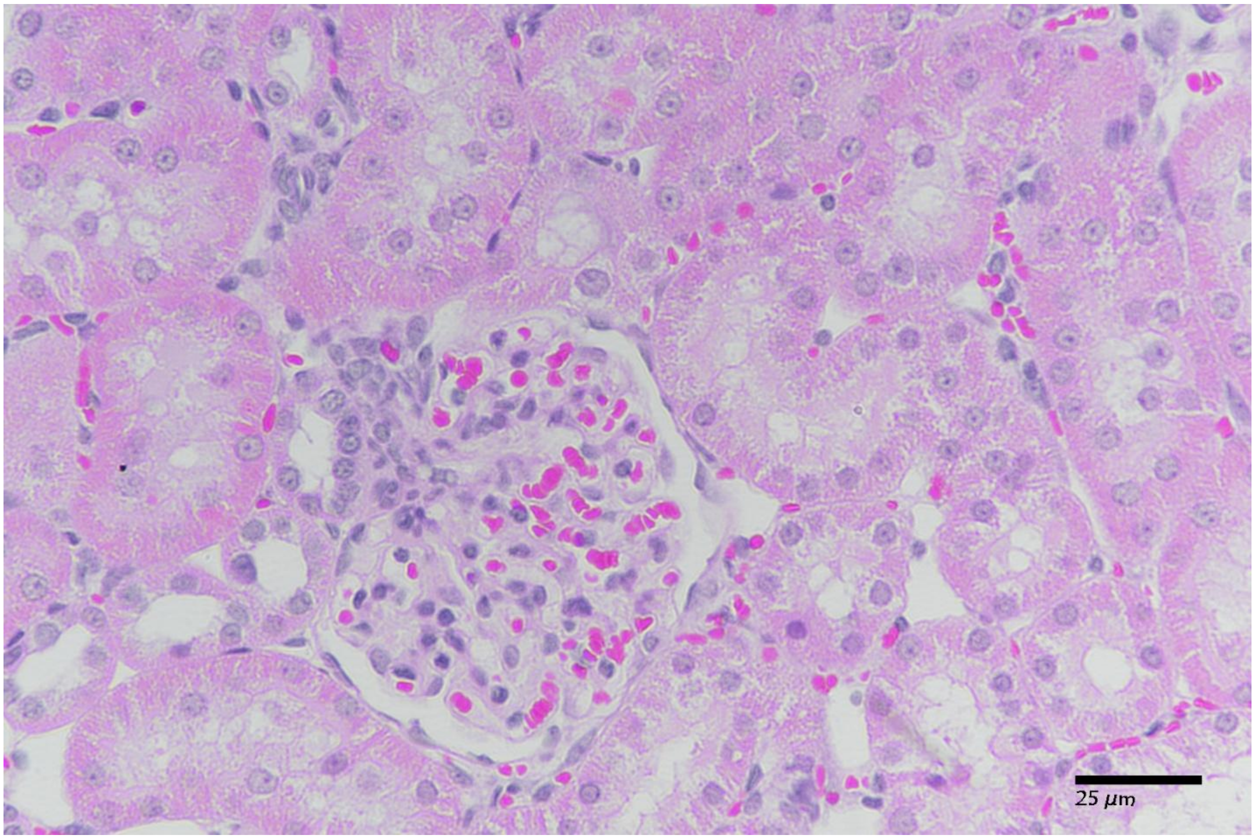
25 μm

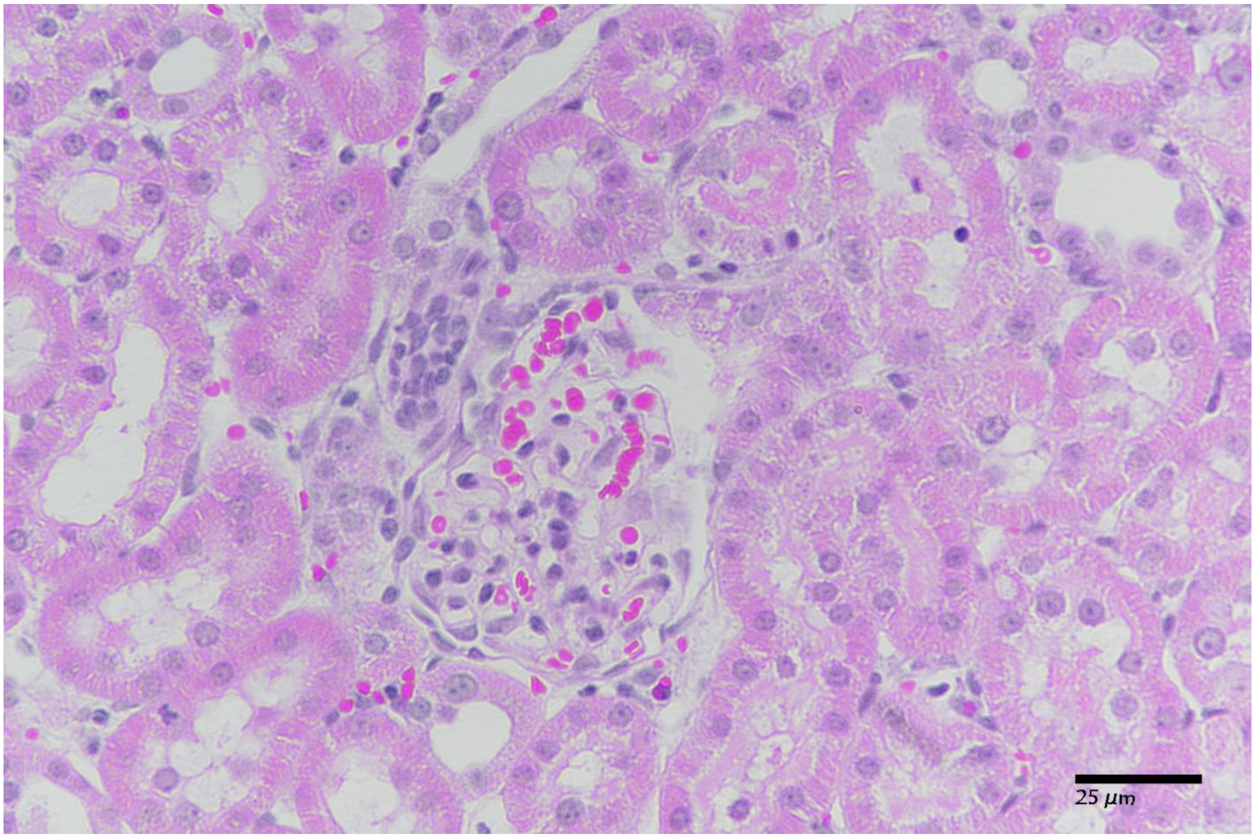
25 μm

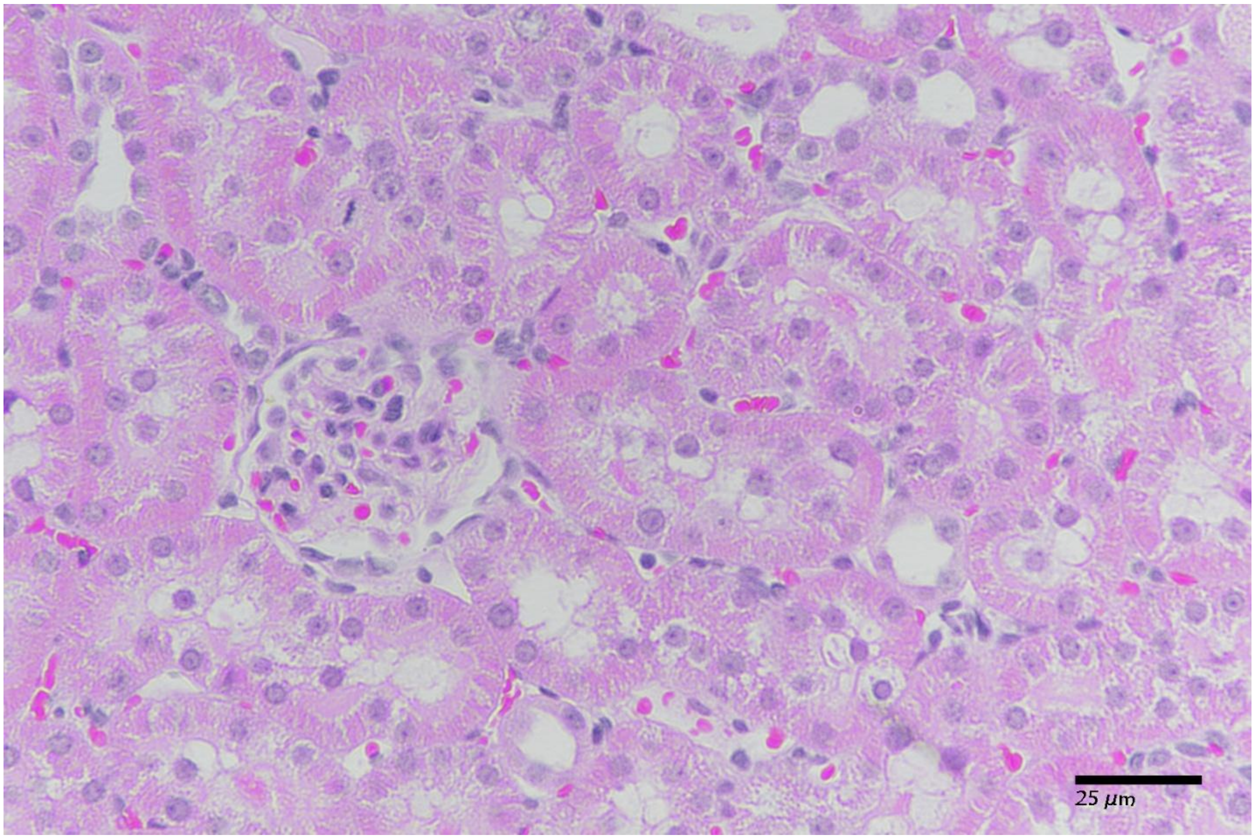
25 μm

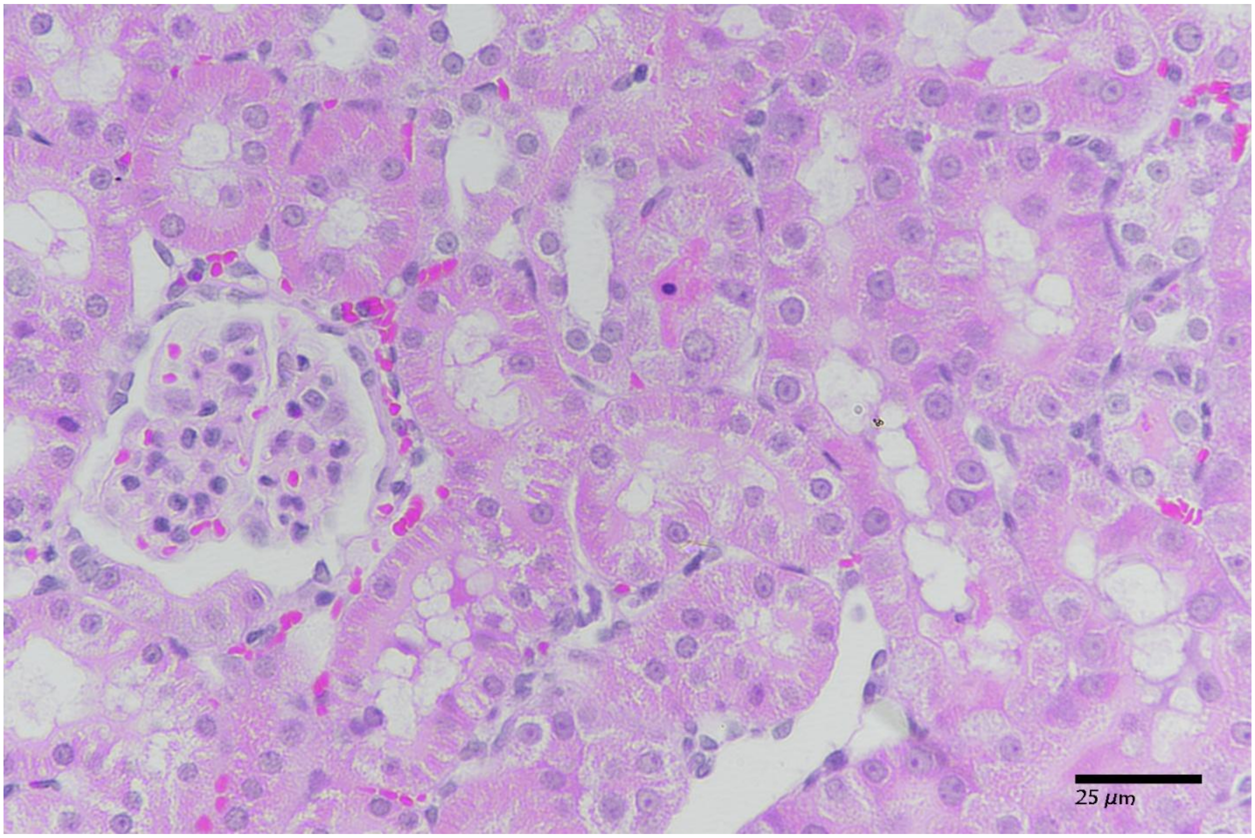
25 μm

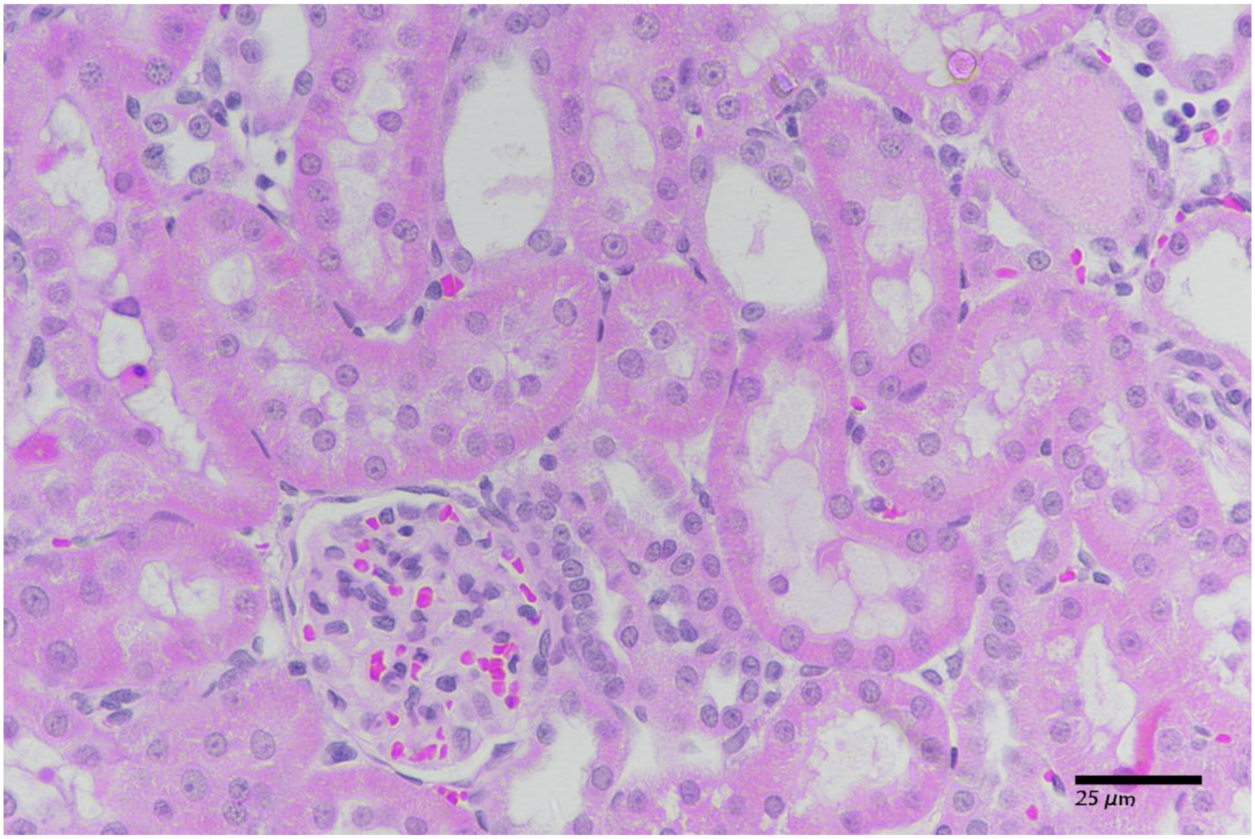
25 μm

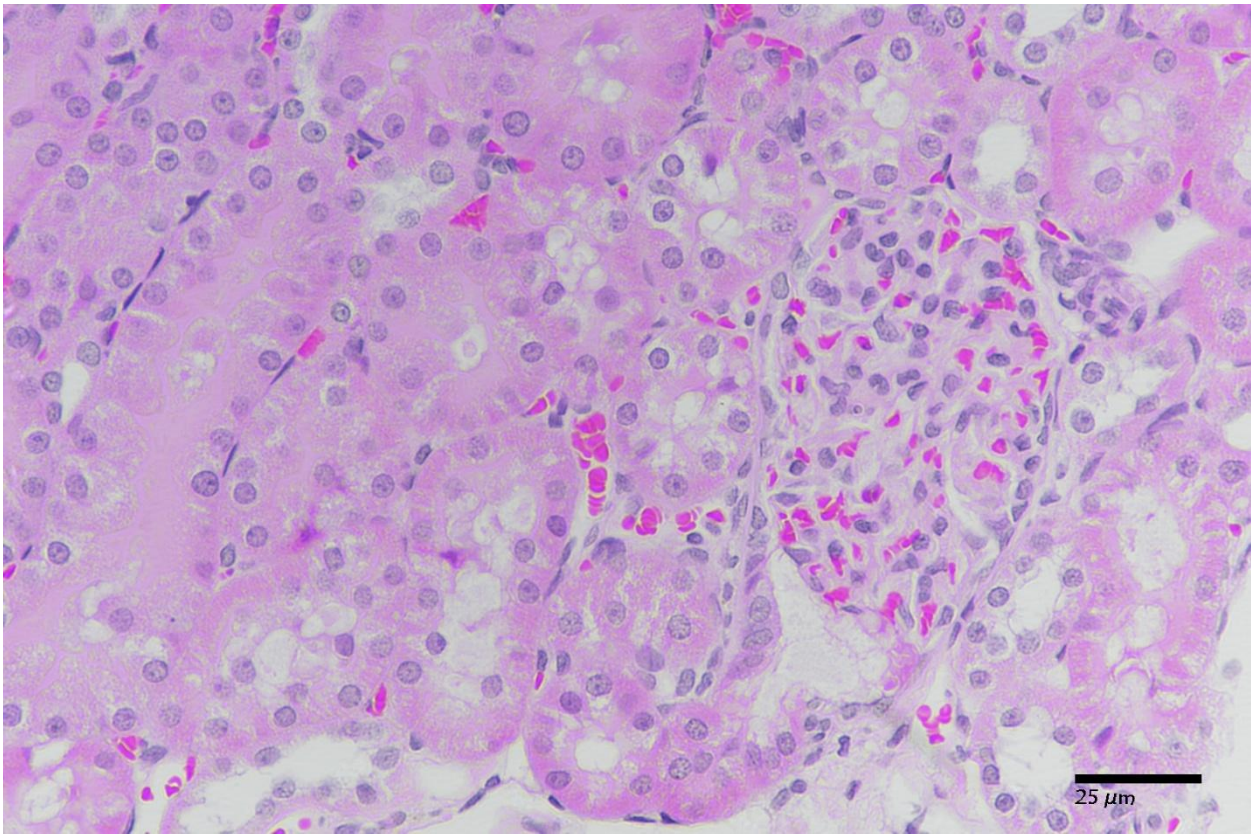
25 μm

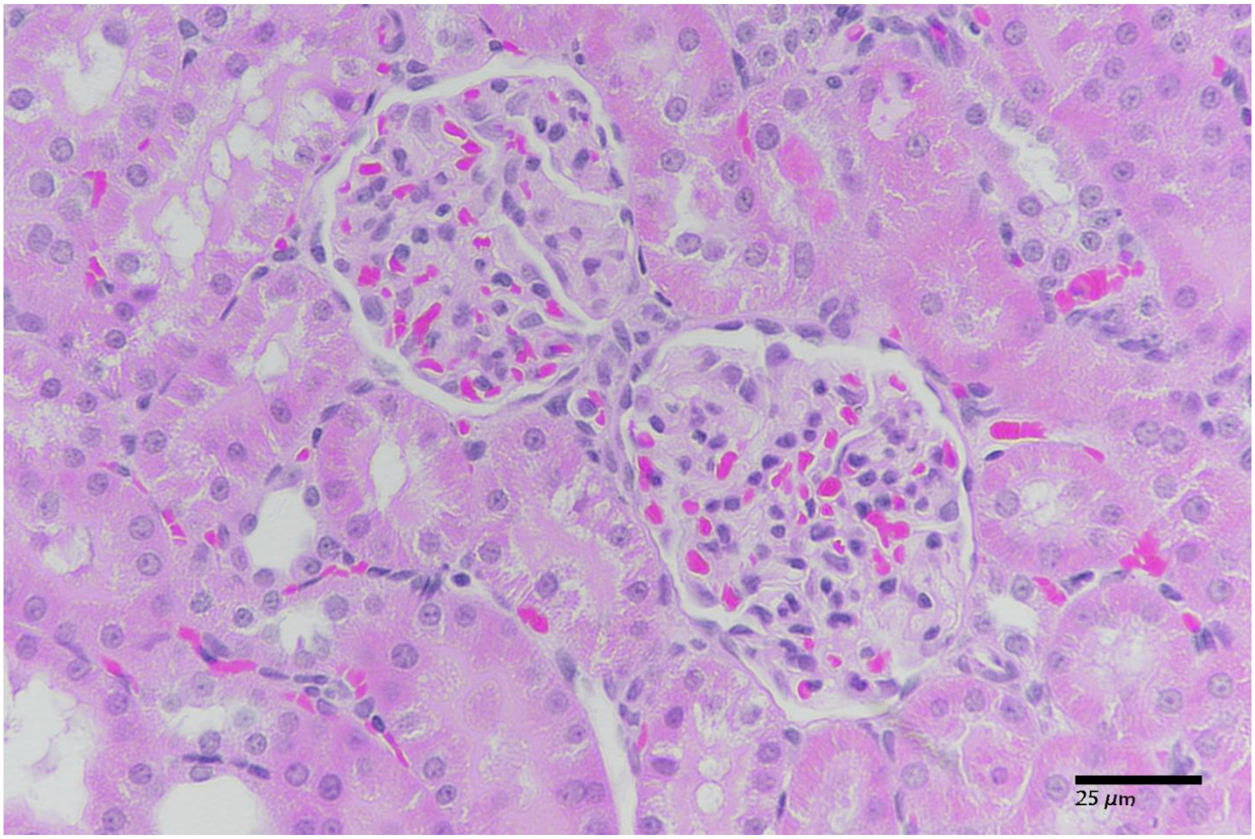
25 μm

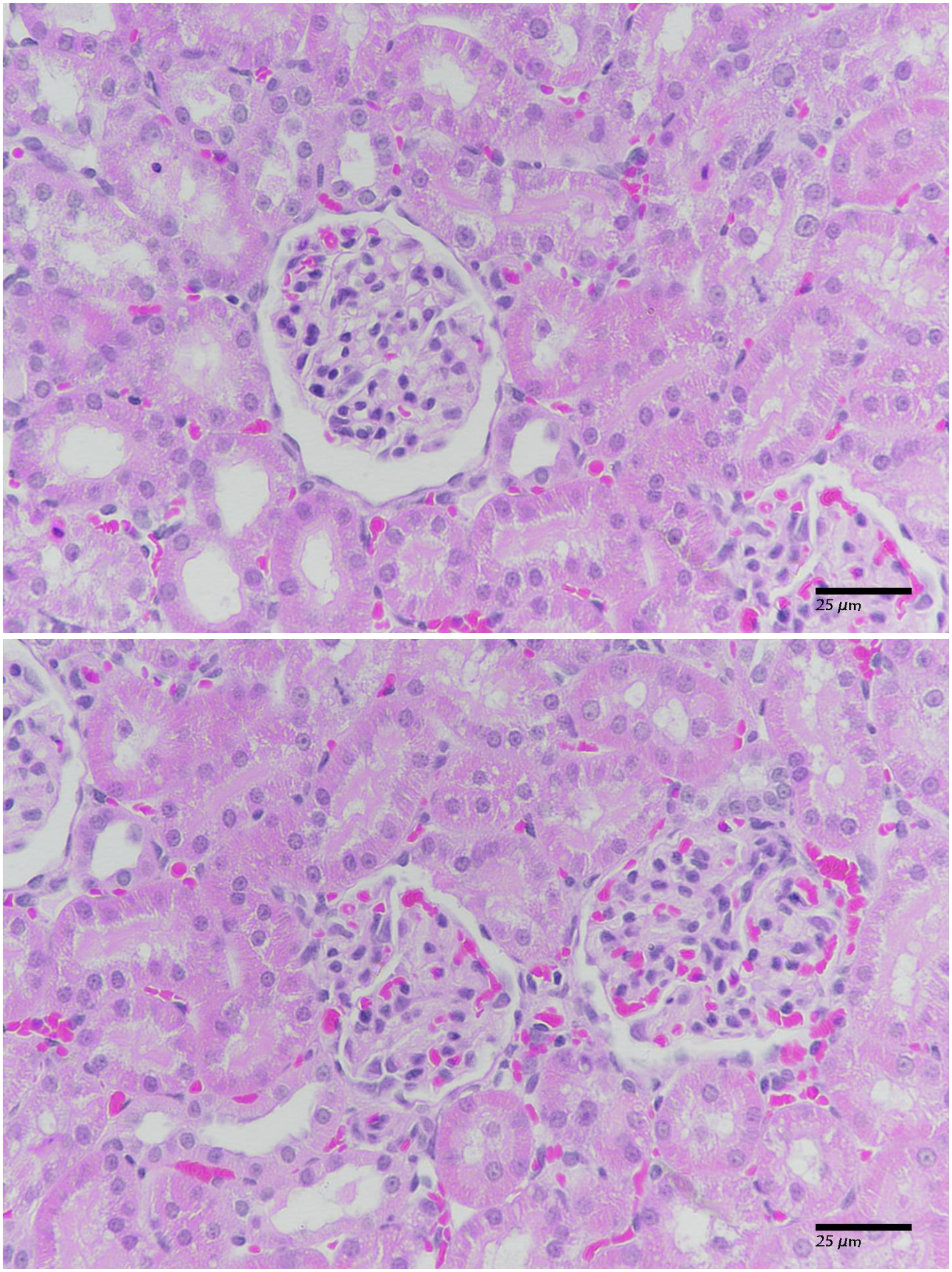
25 μm
25 μm

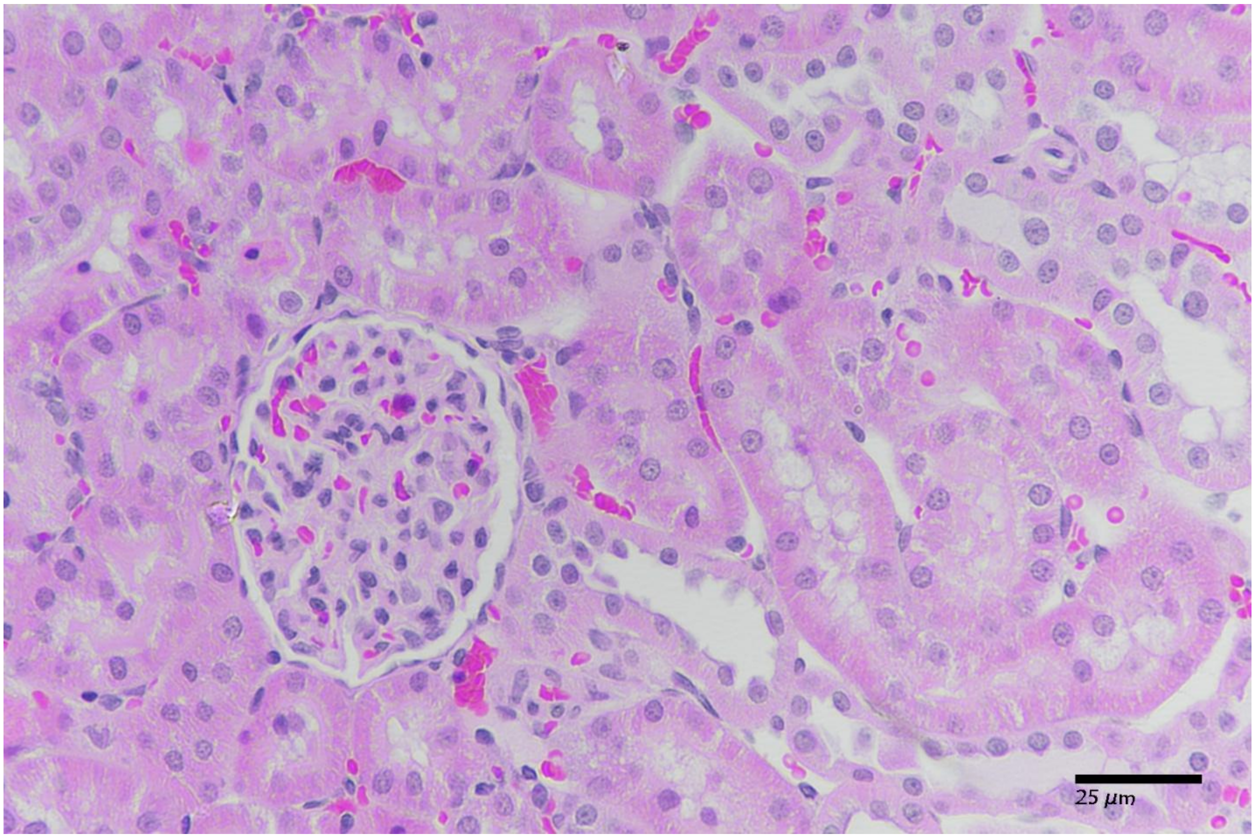
25 μm

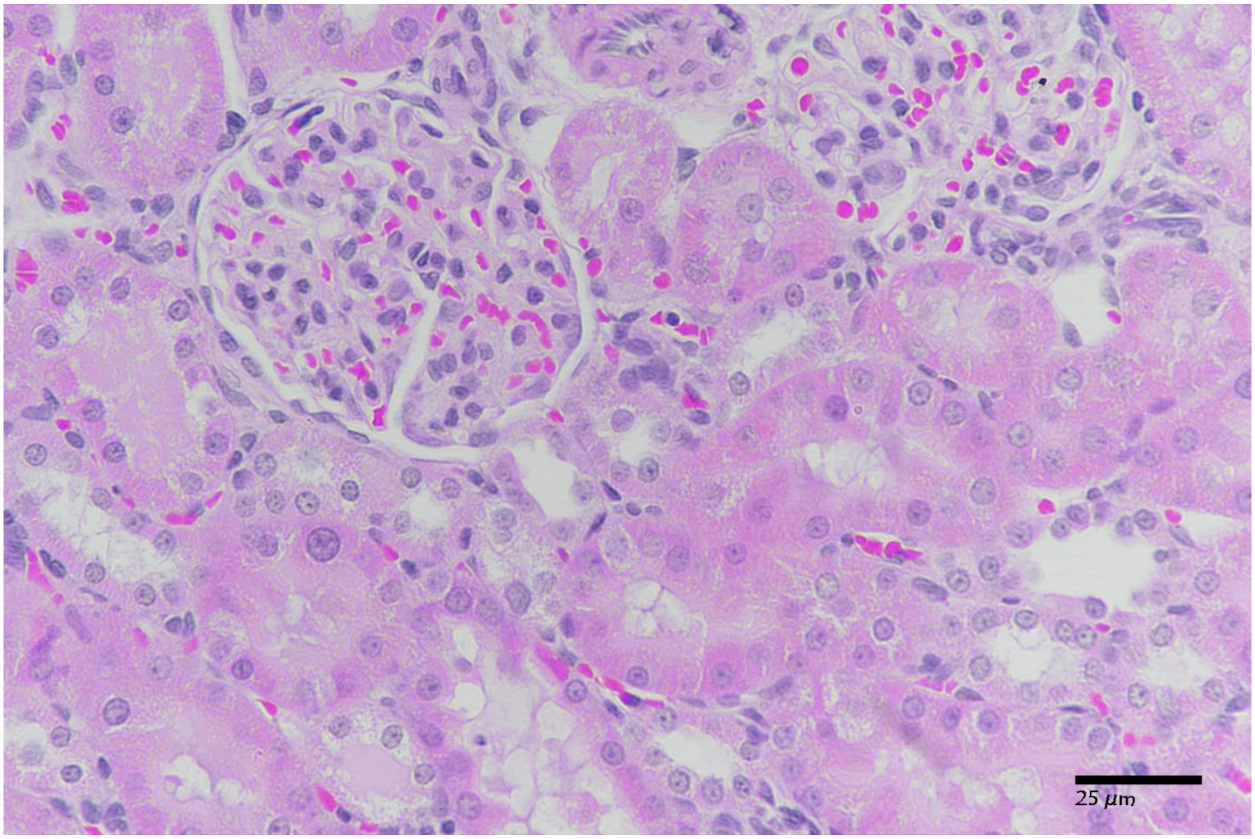
25 μm

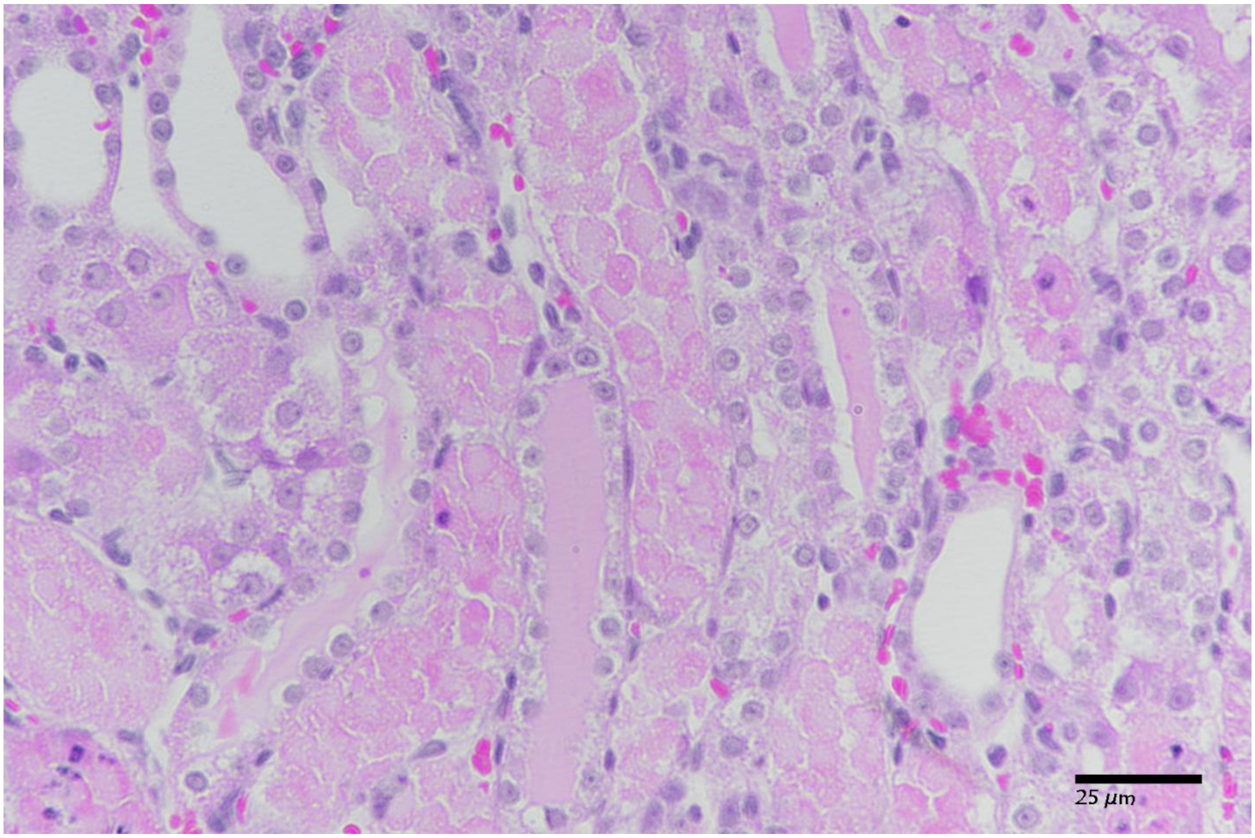
25 μm

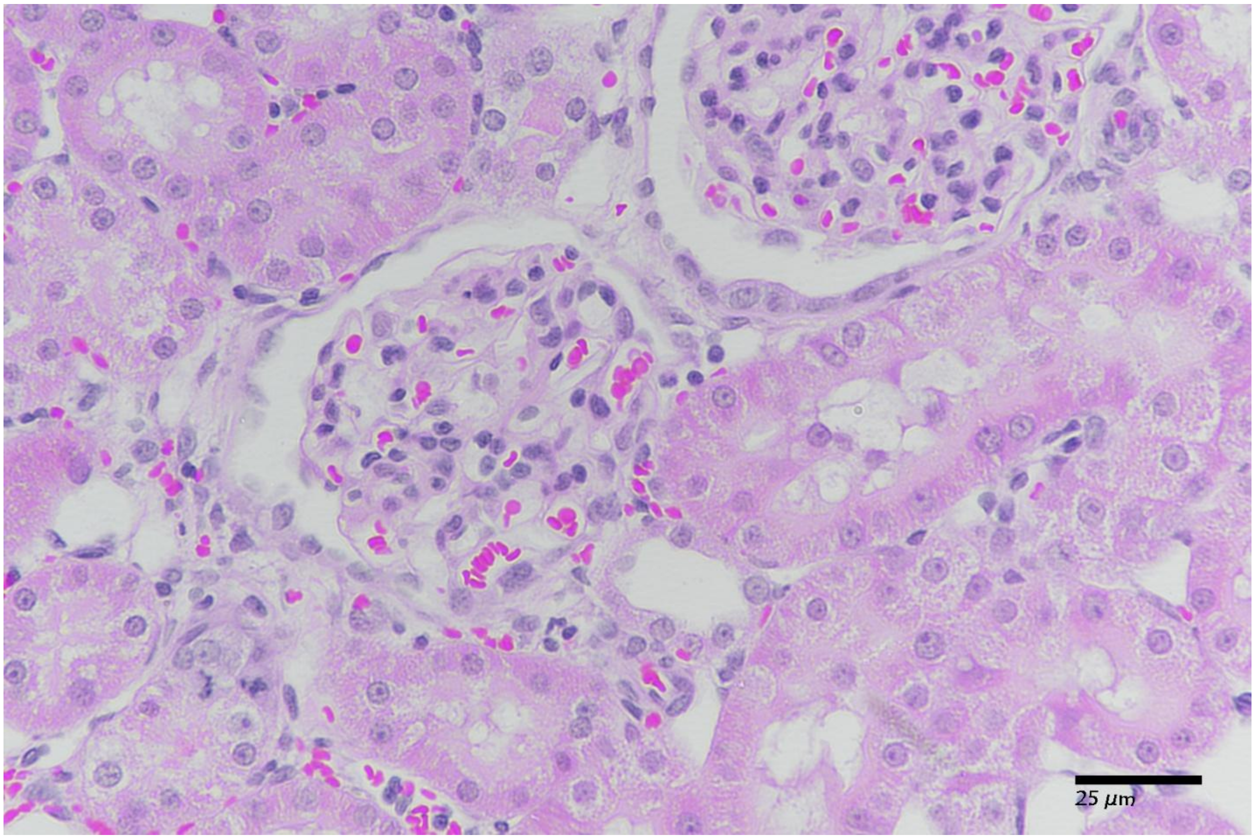
25 μm

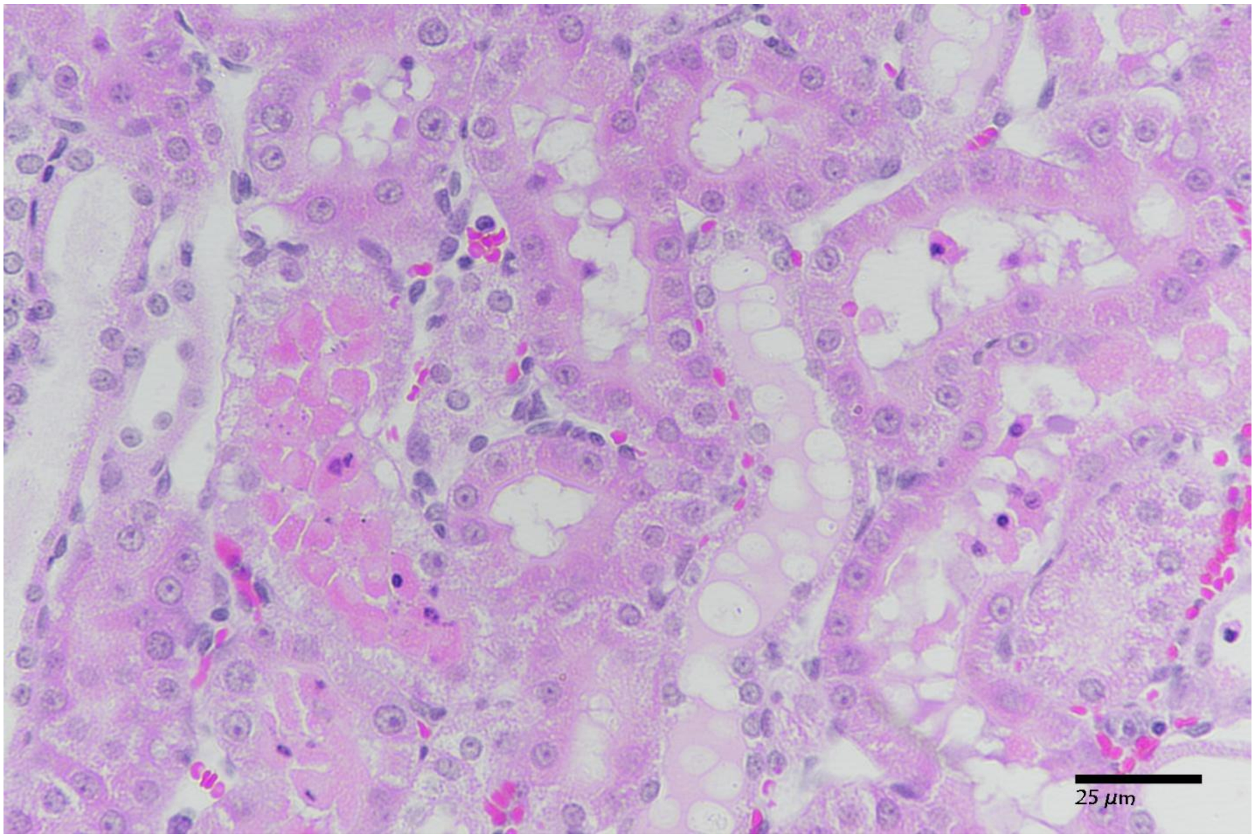
25 μm

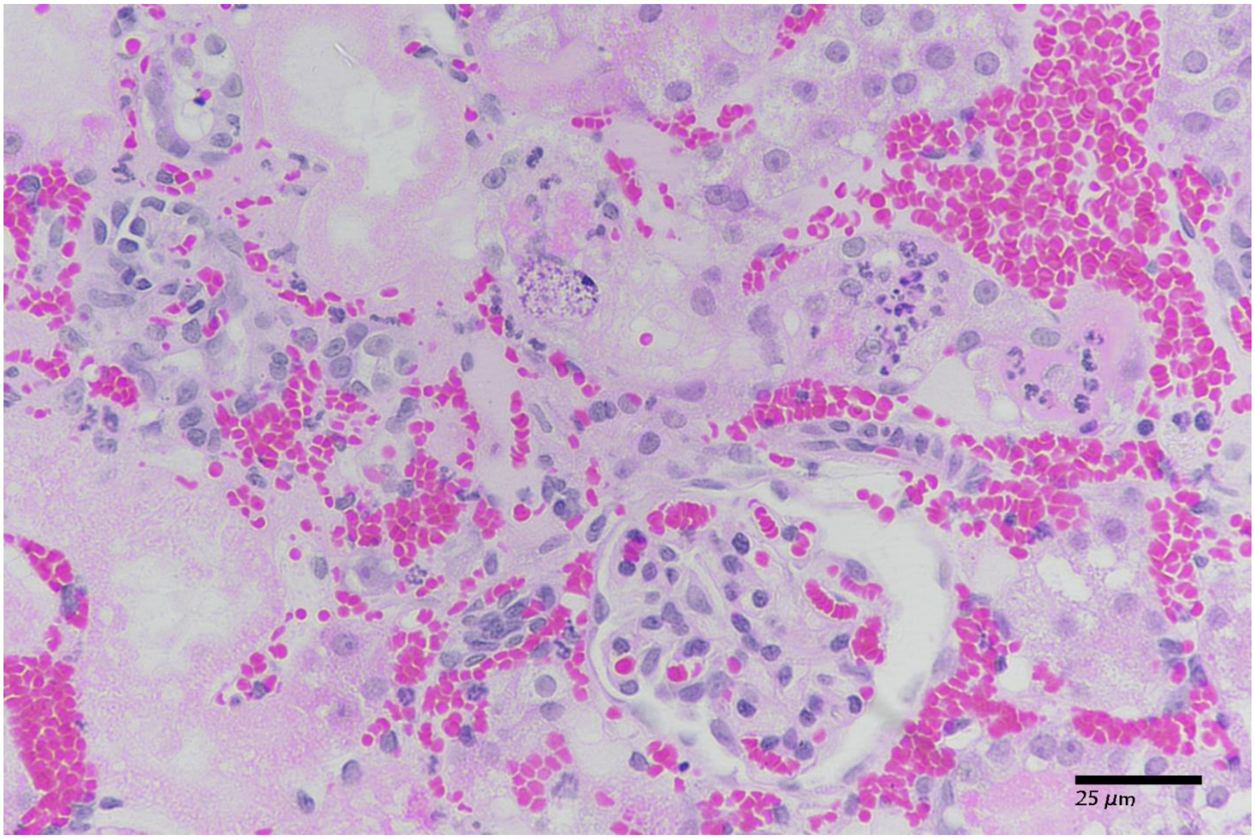
25 μm

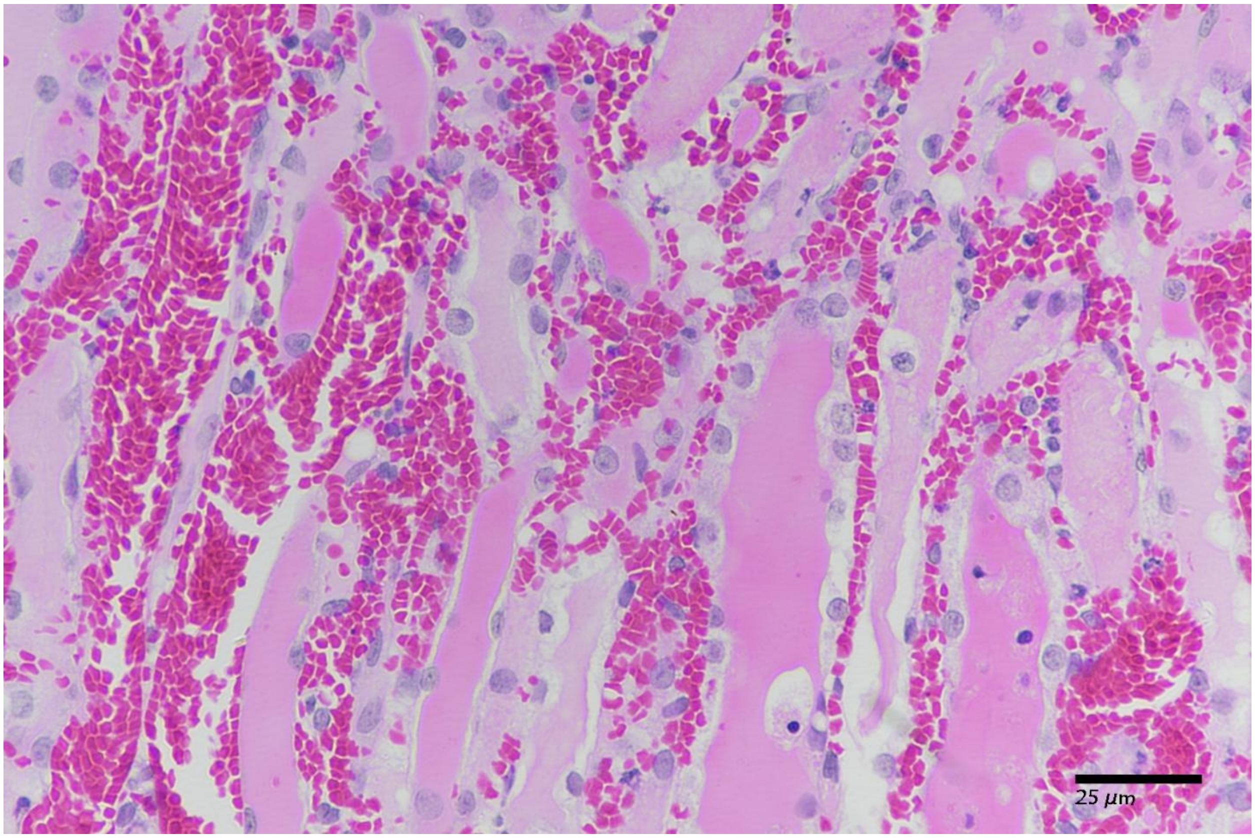
25 µm

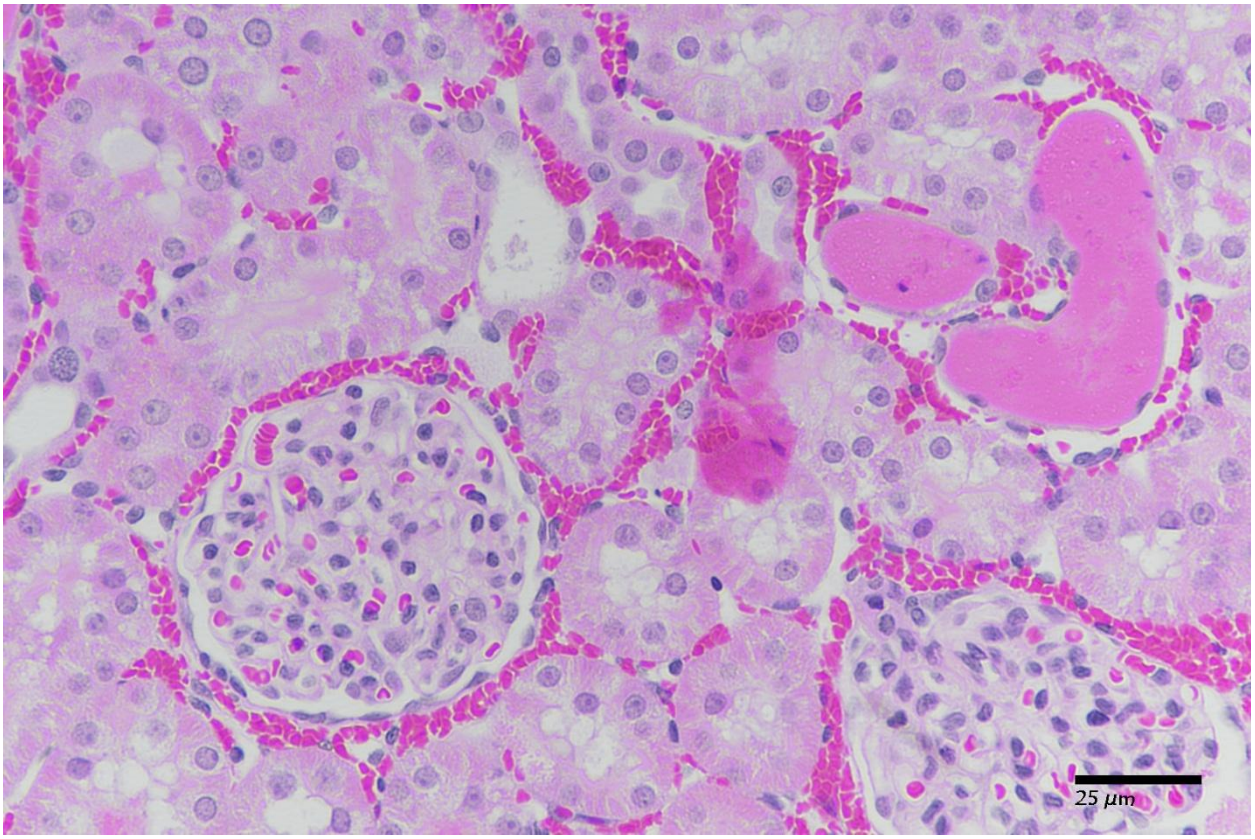
25 µm

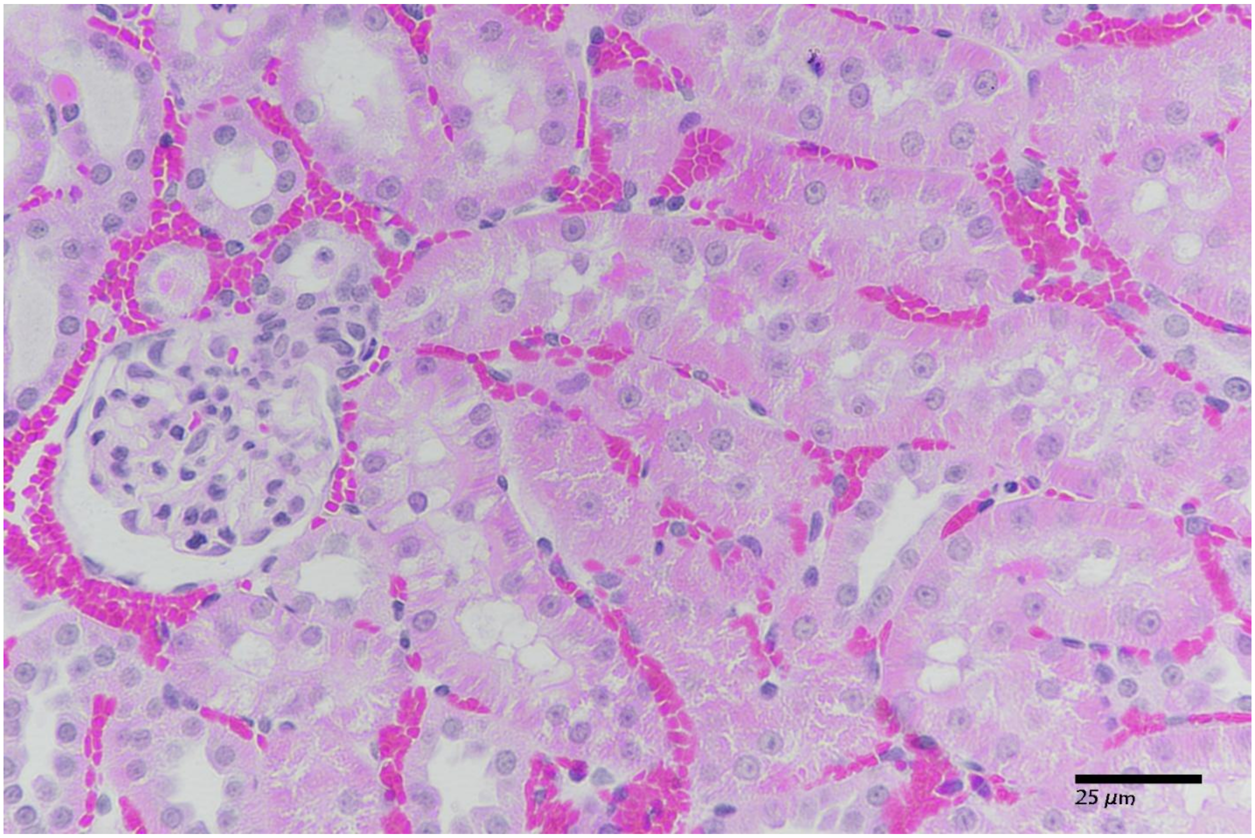
25 μm

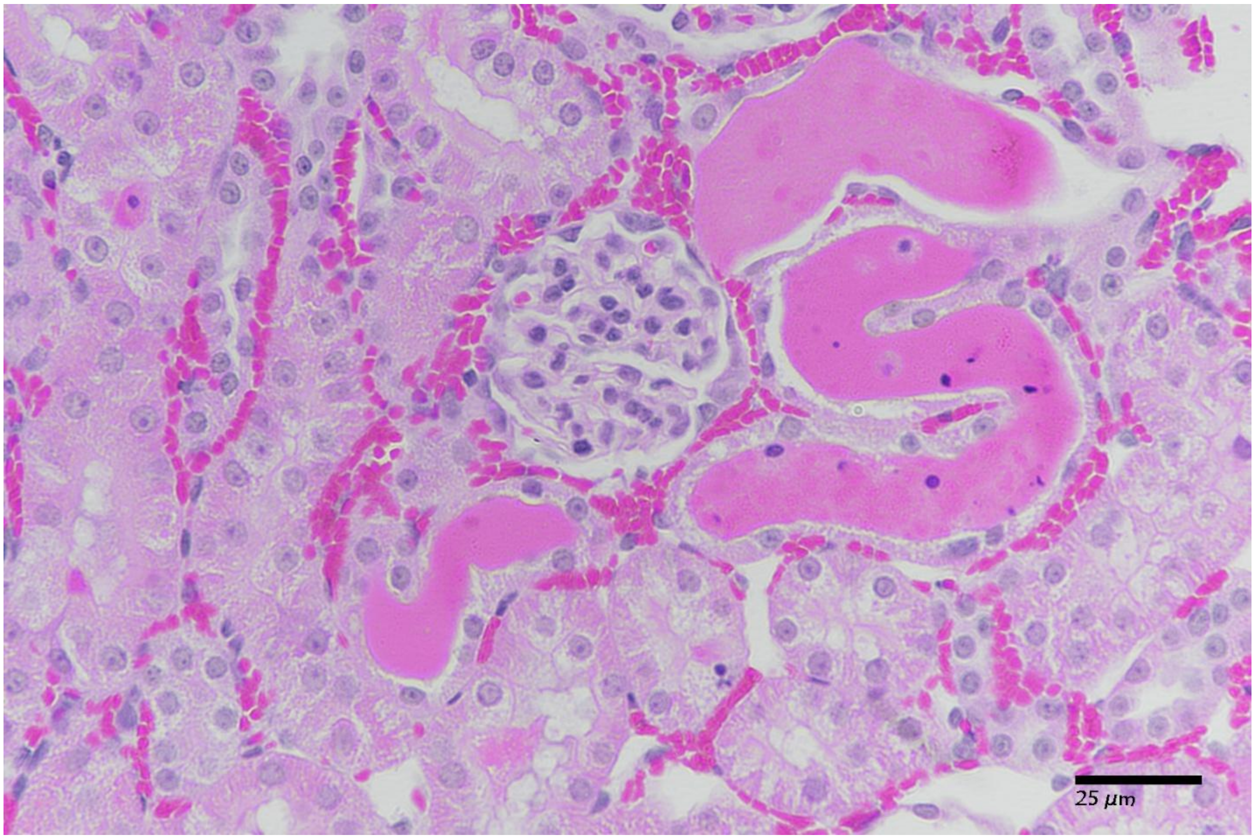
25 μm

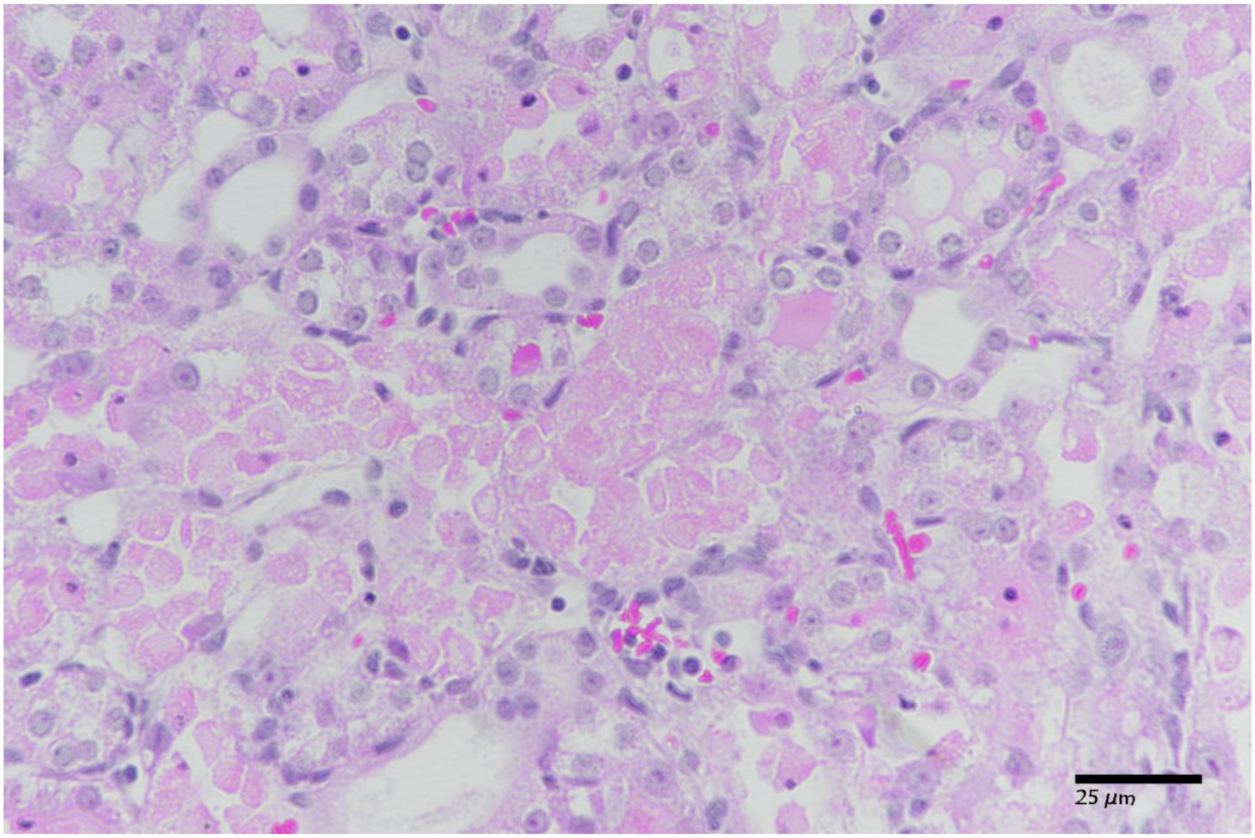
25 μm

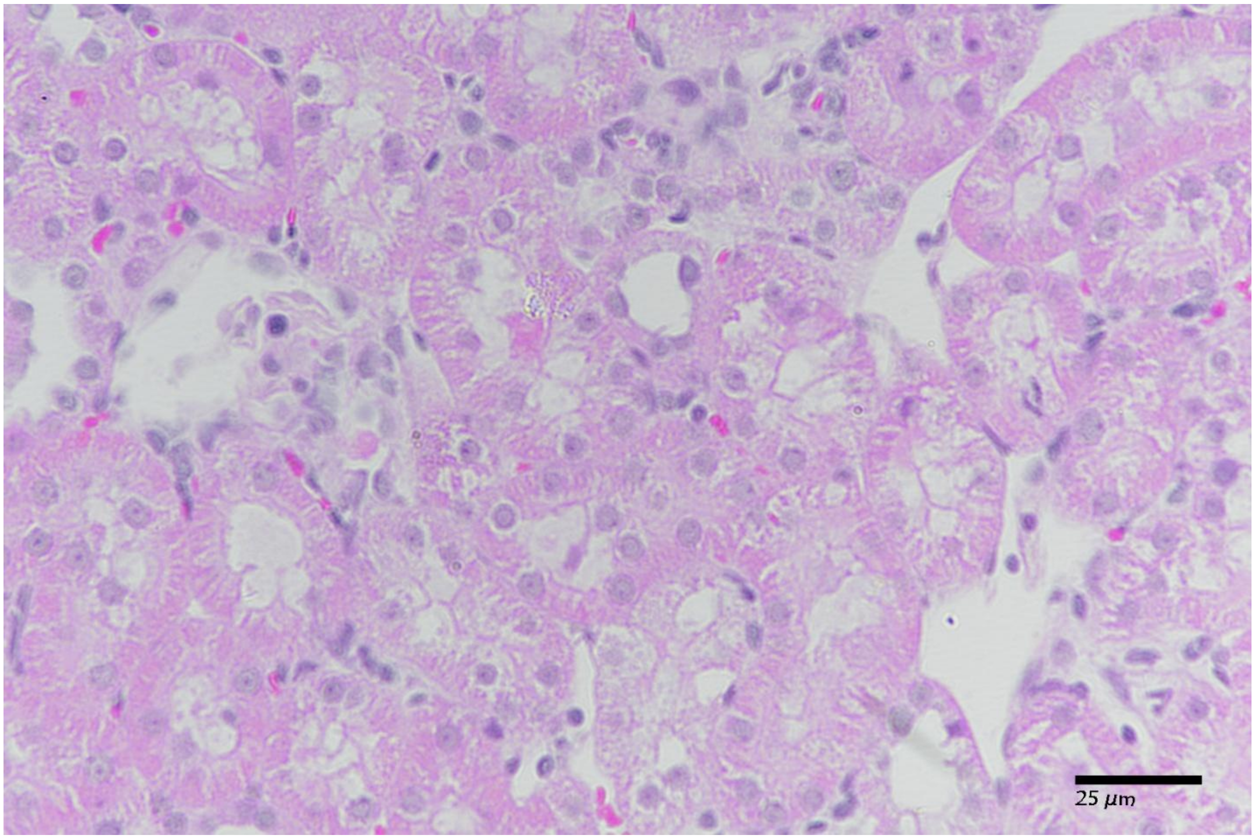
25 μm

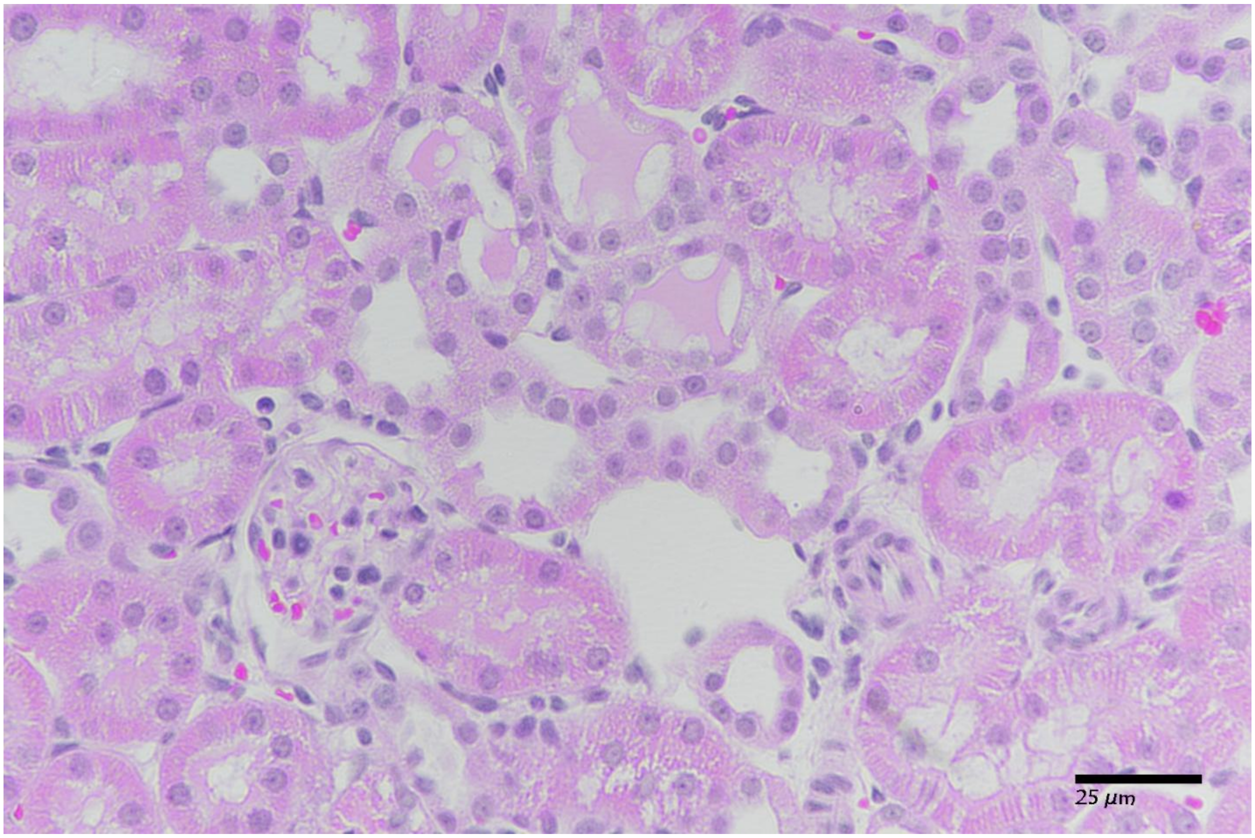
25 μm

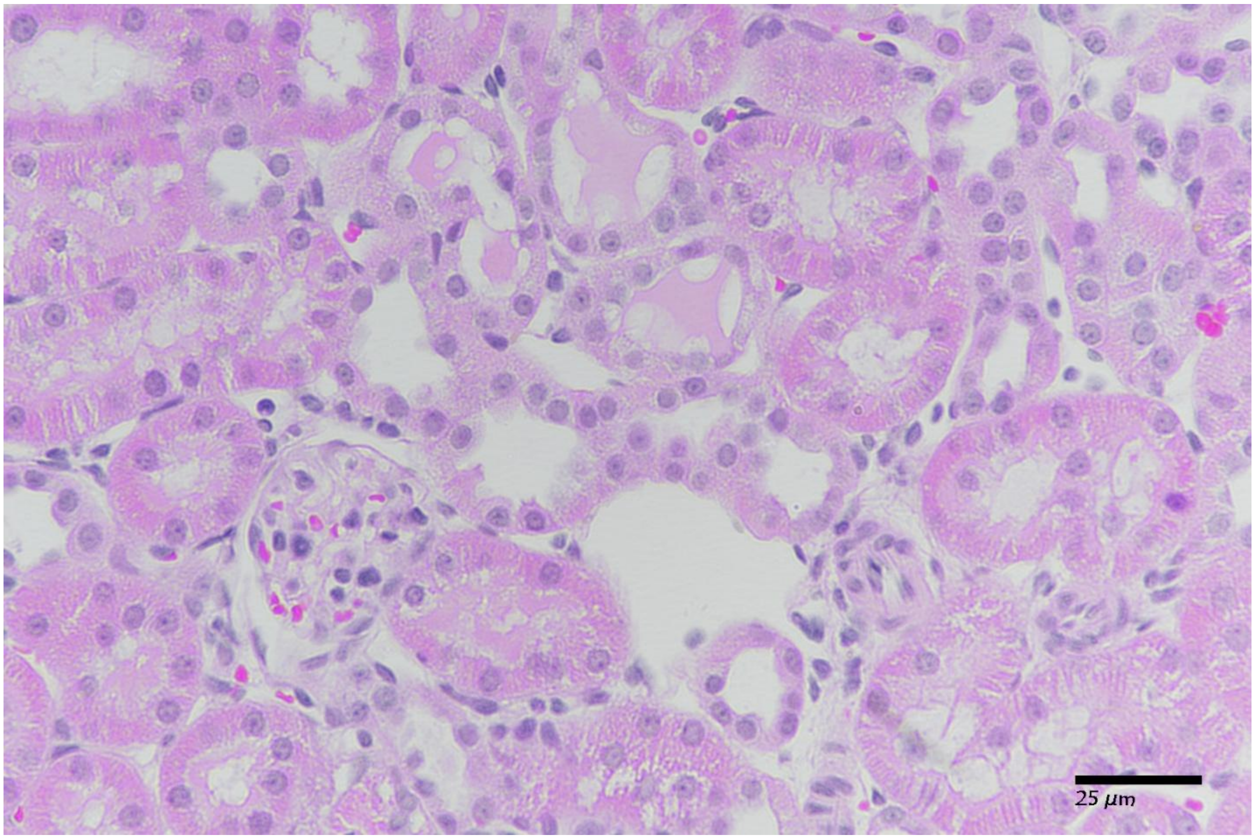
25 μm

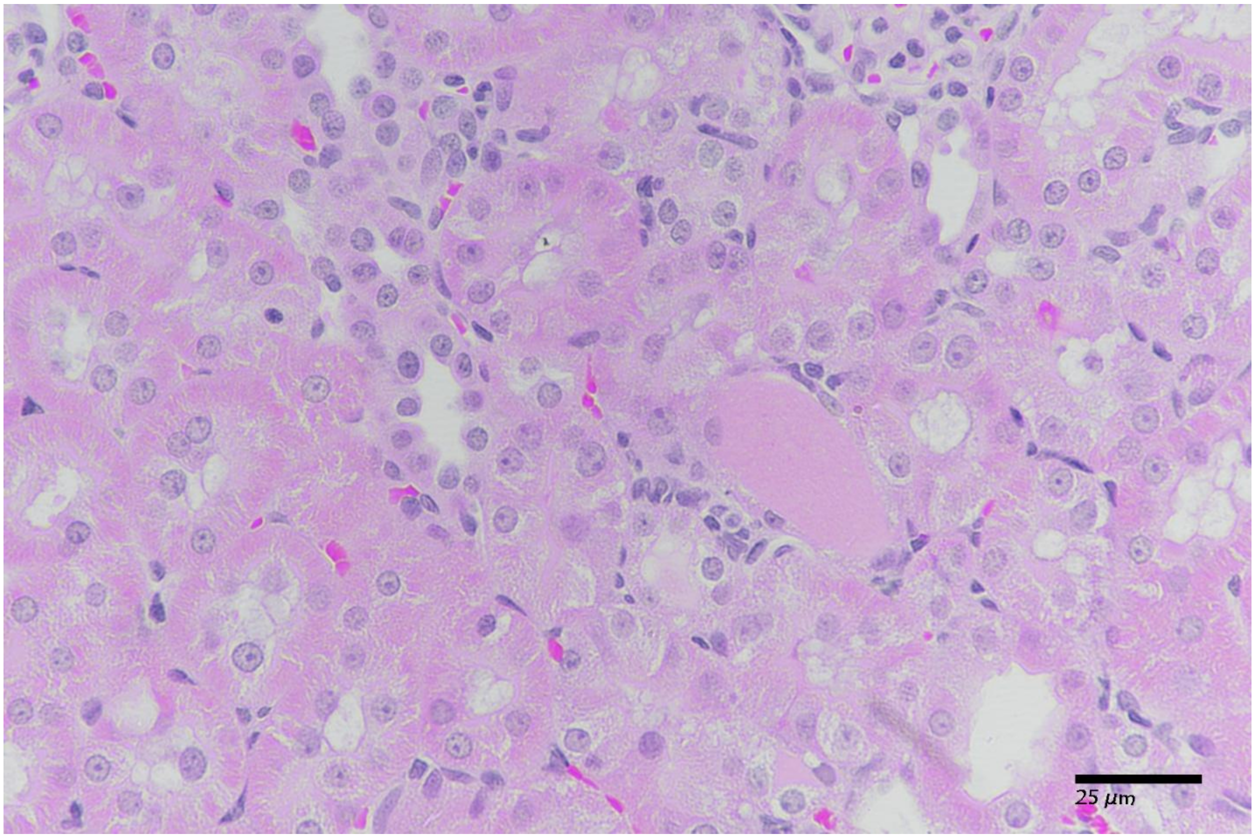
25 μm
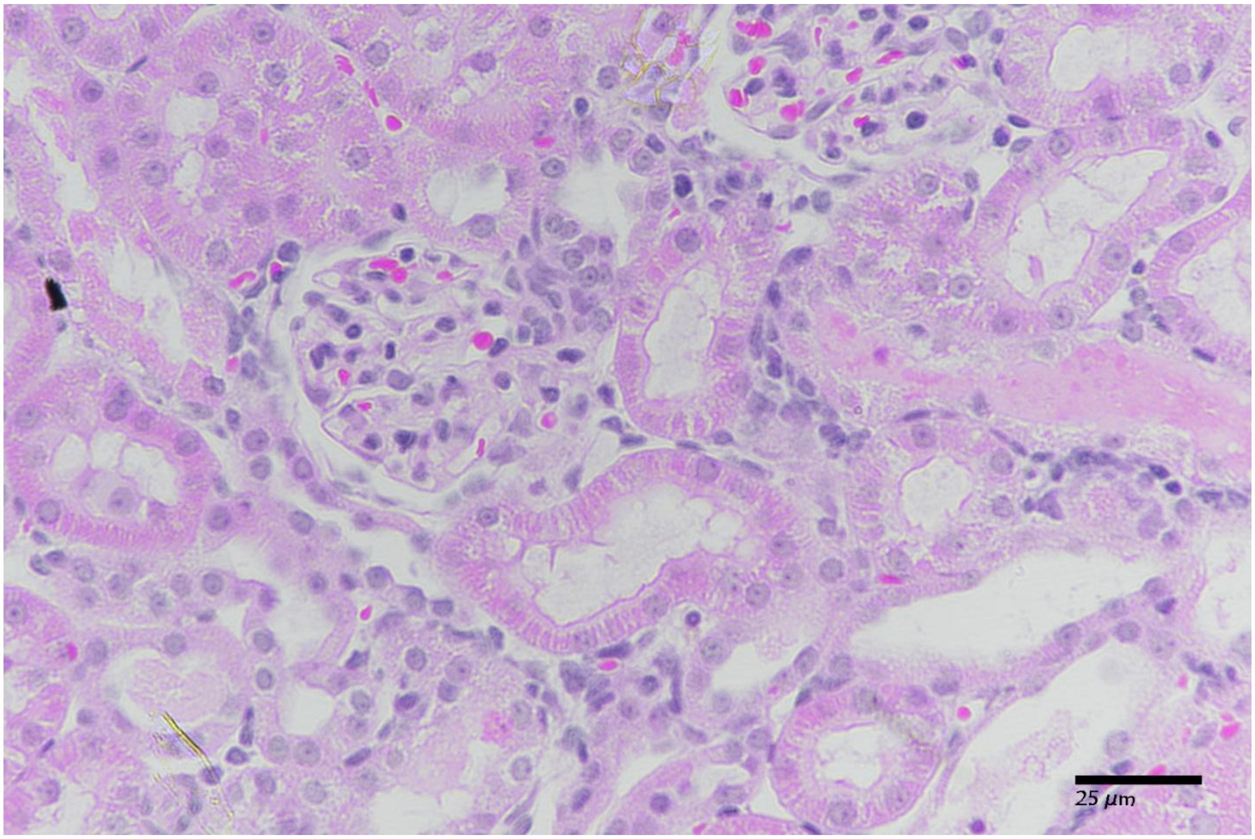
25 μm

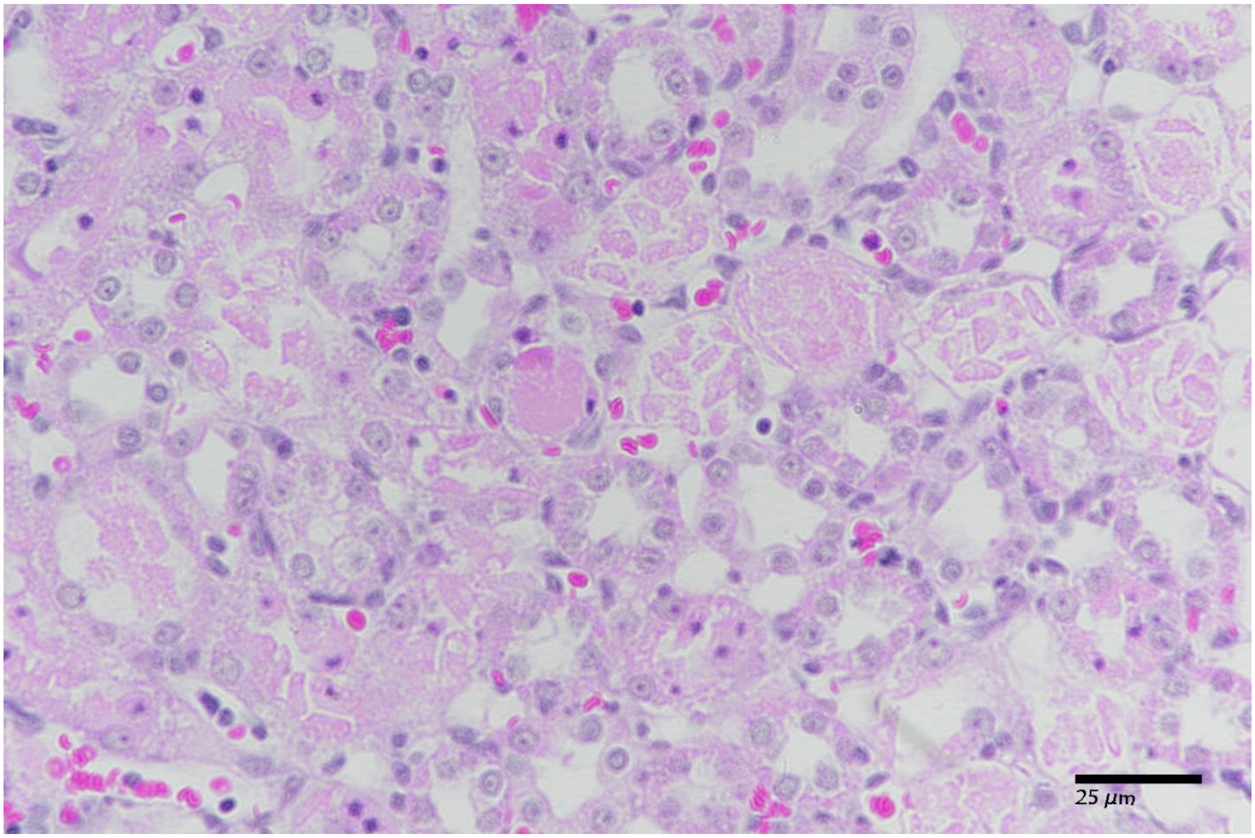
25 μm

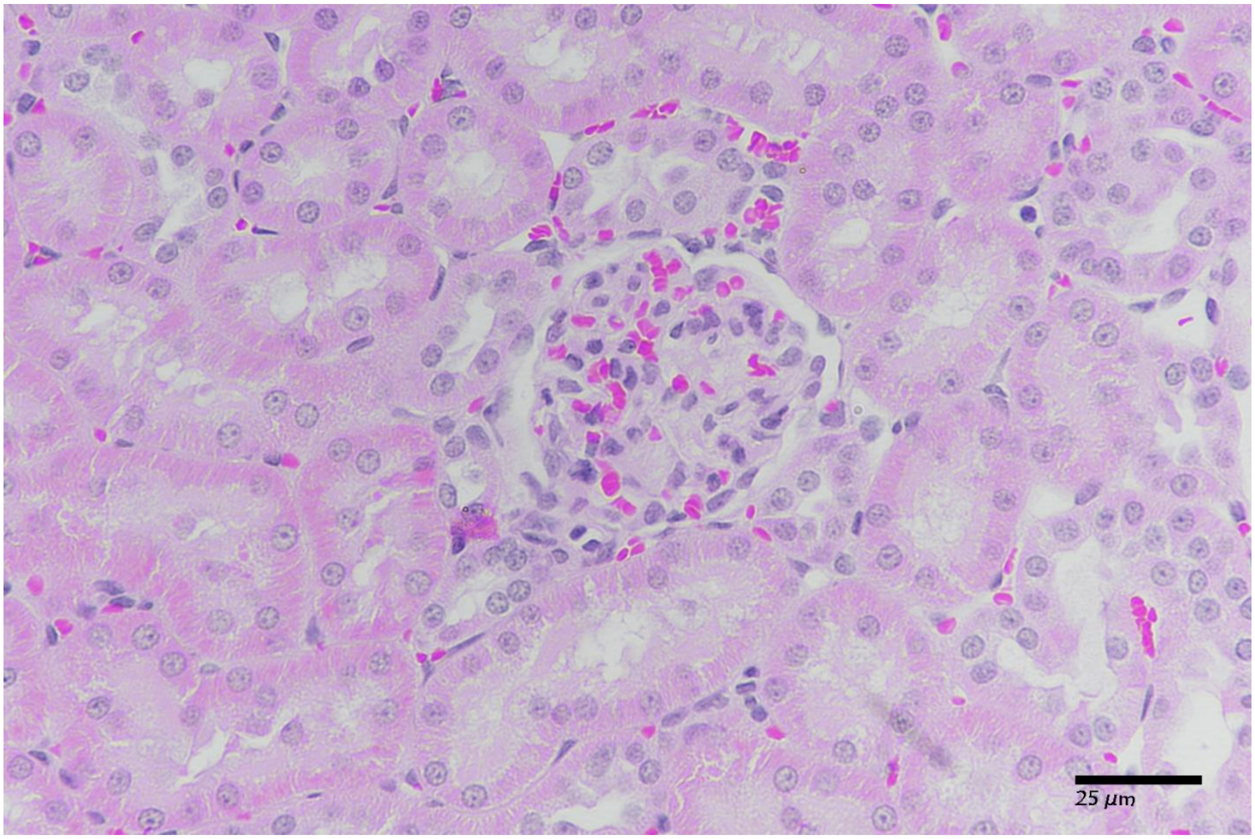
25 μm

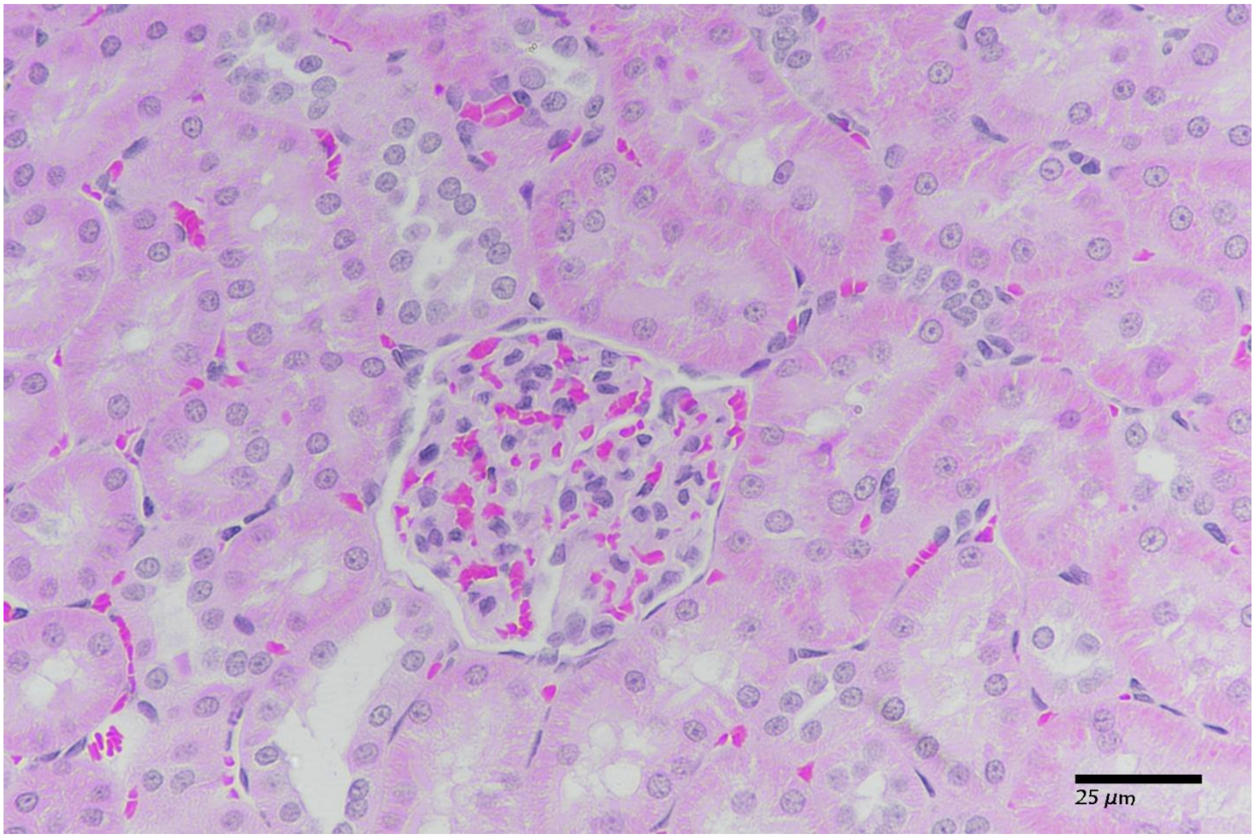
25 μm

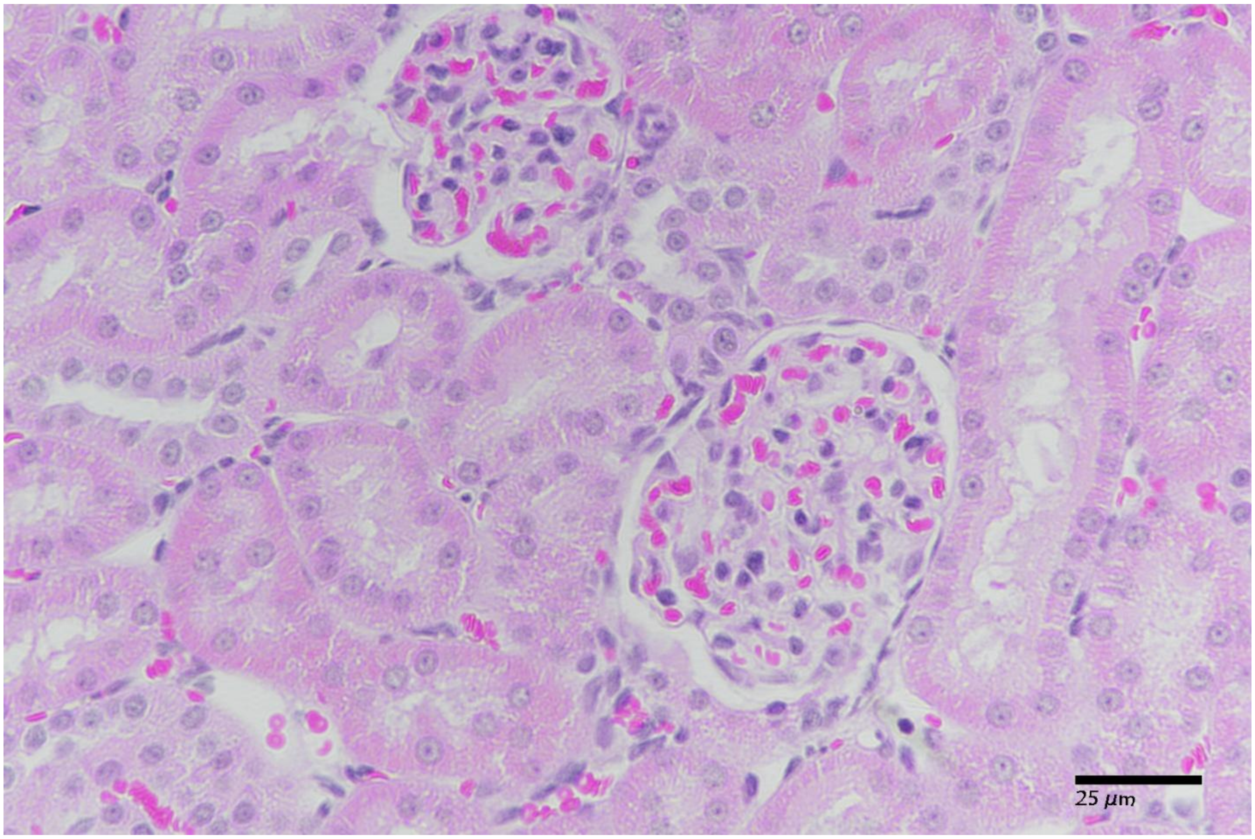
25 μm

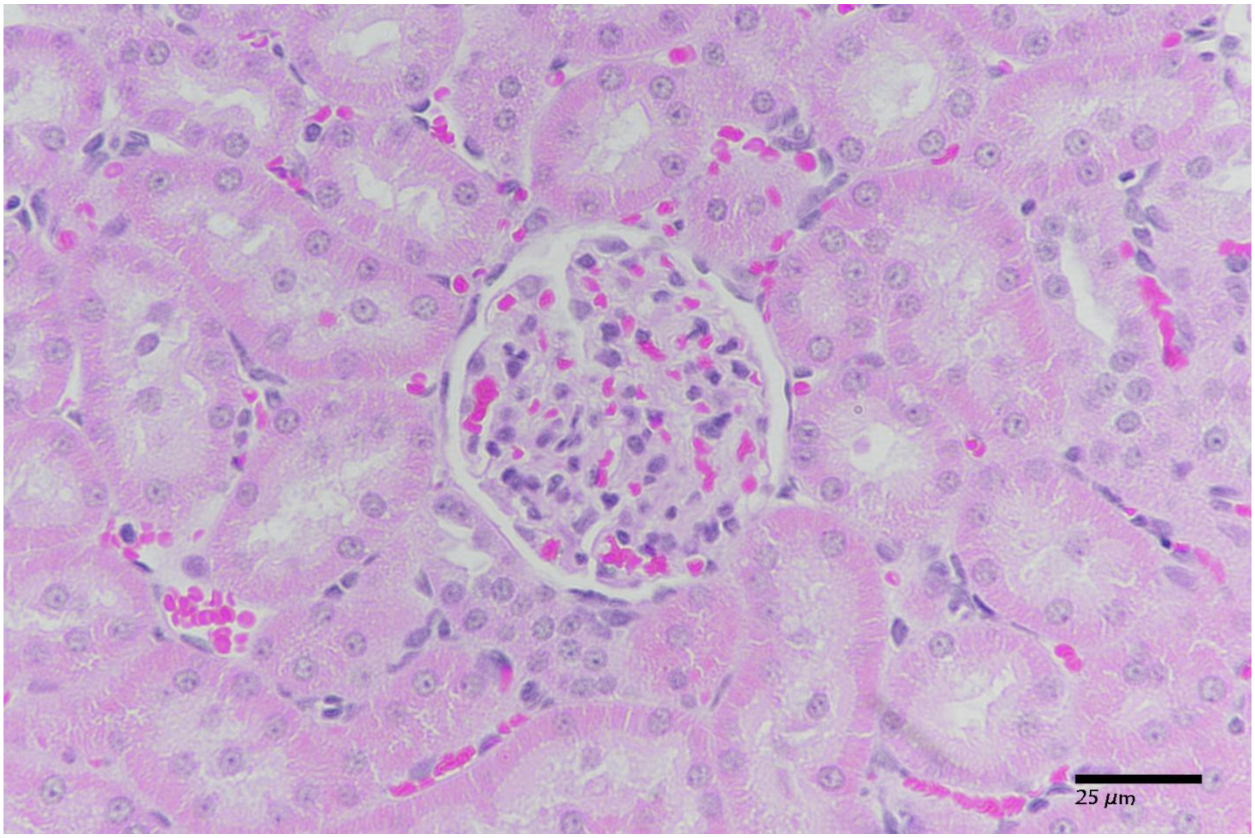
25 μm

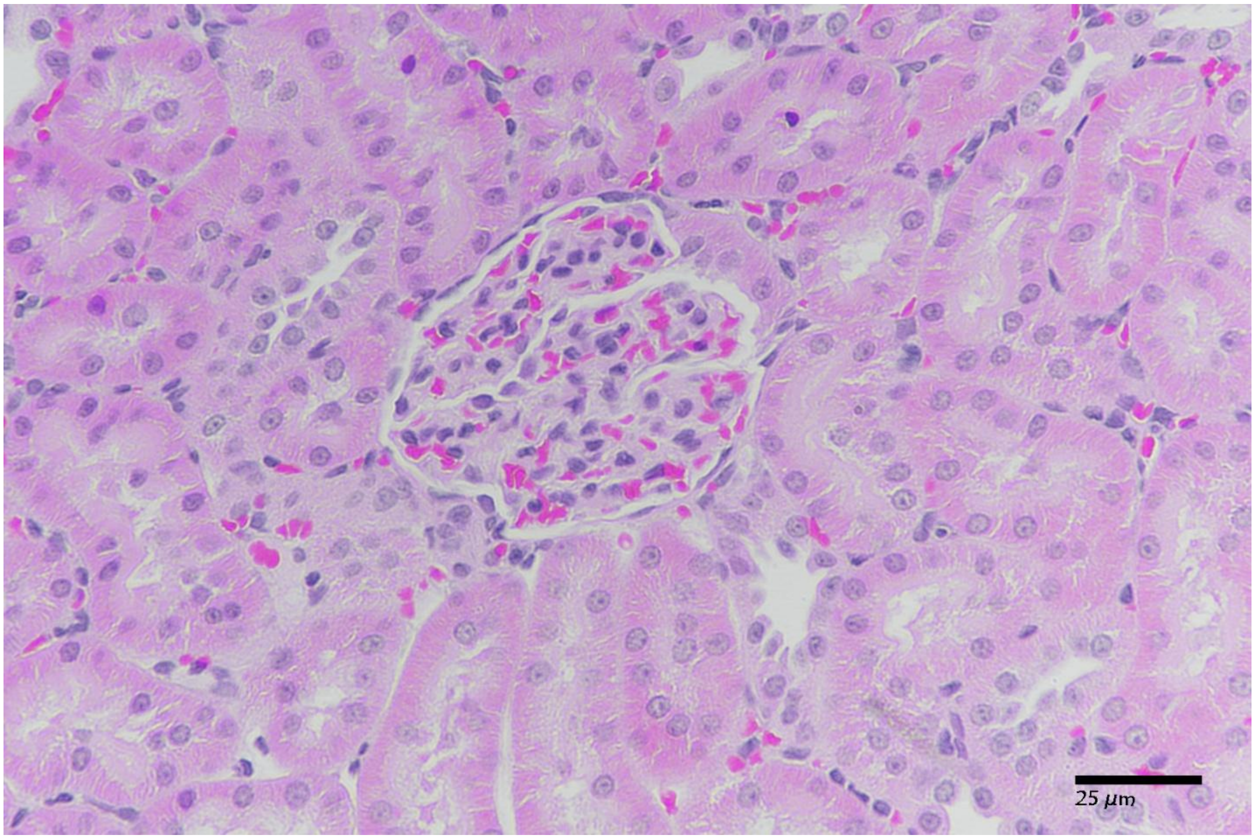
25 μm

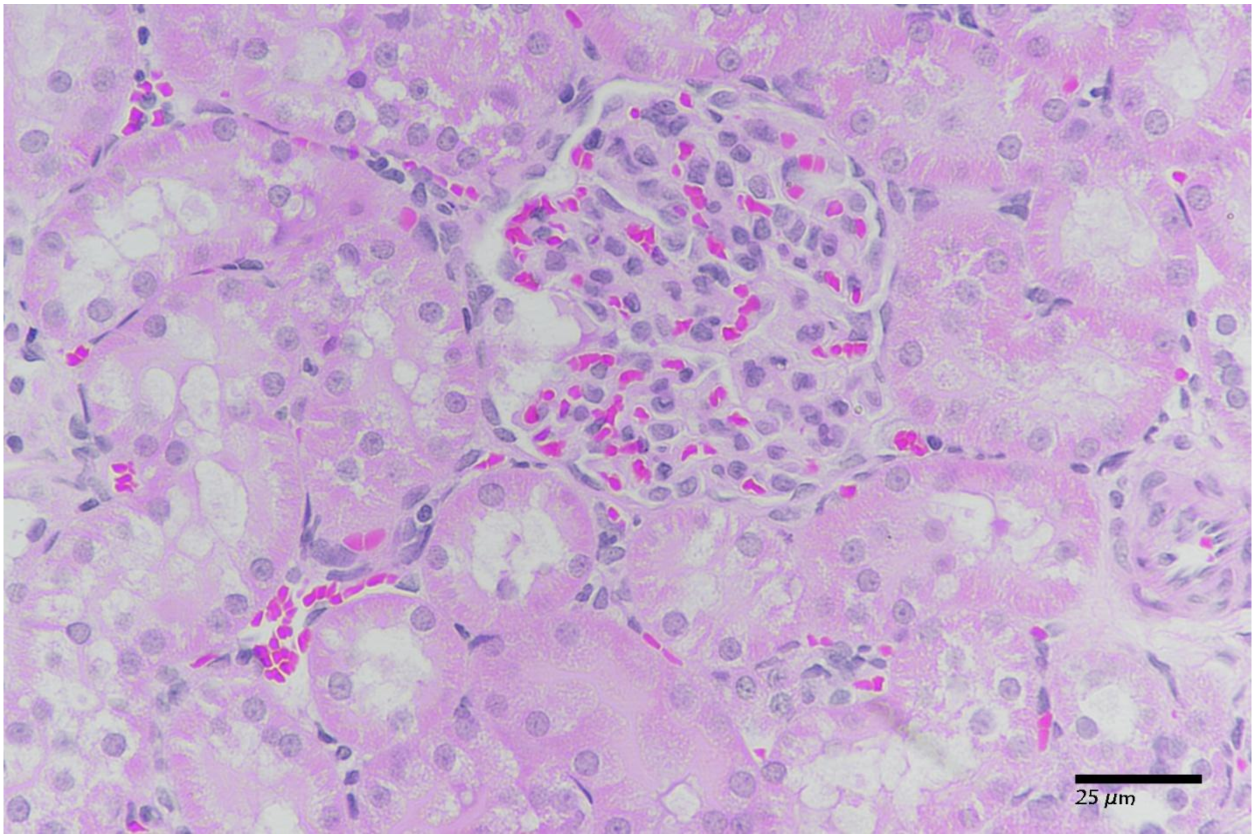
25 μm

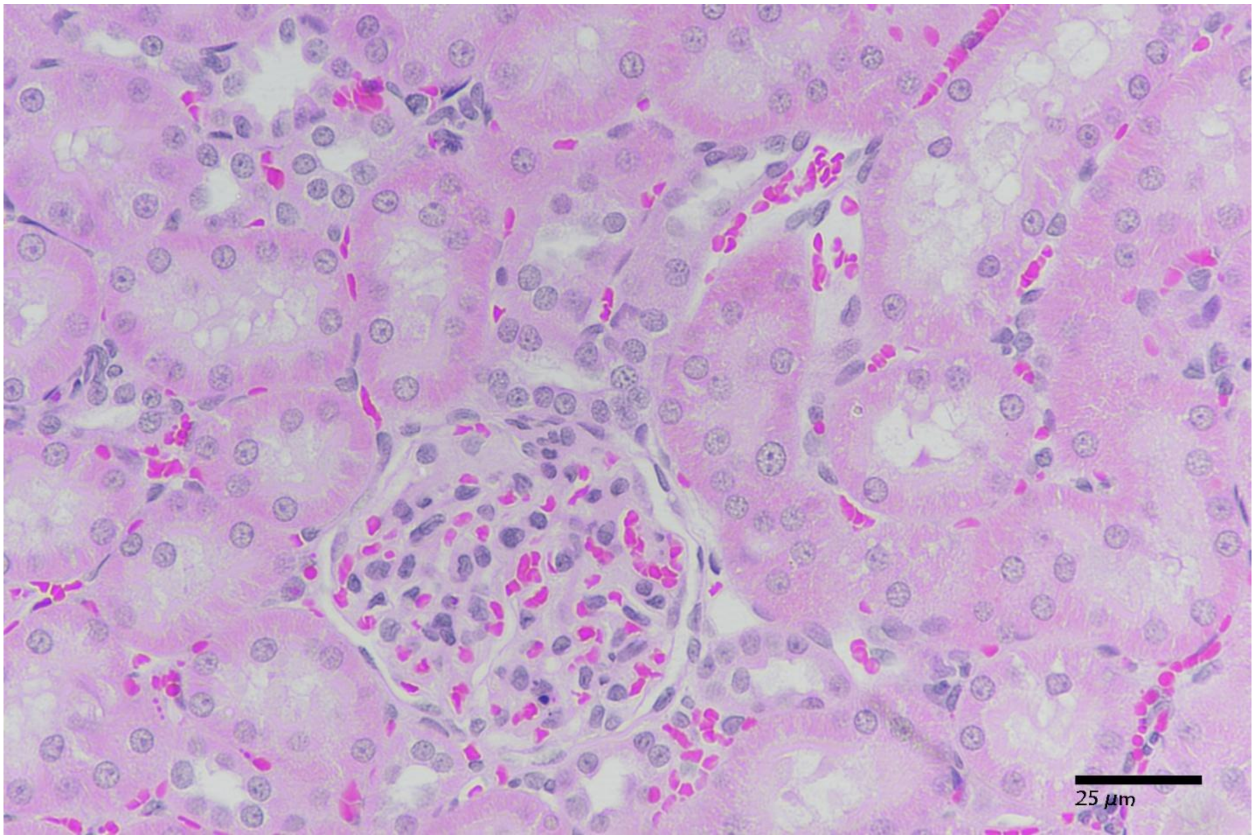
25 μm

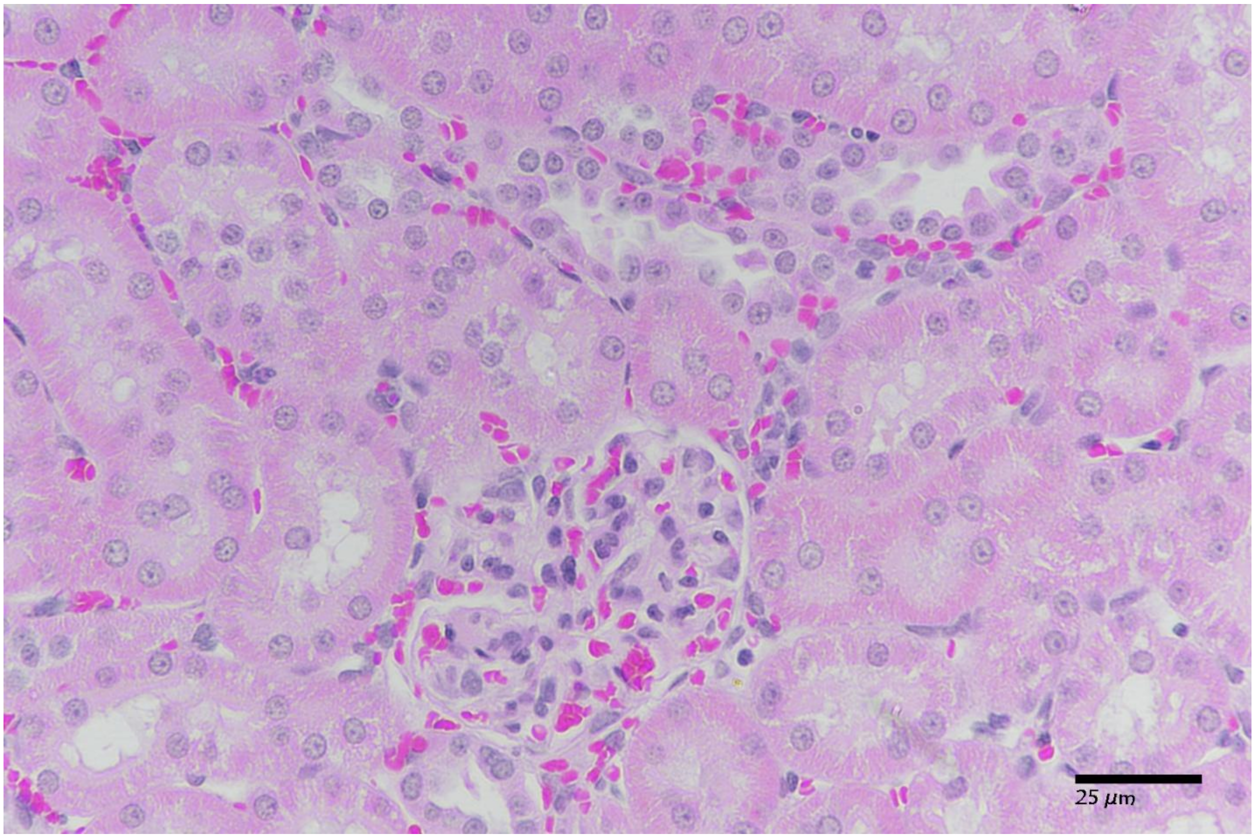
25 μm

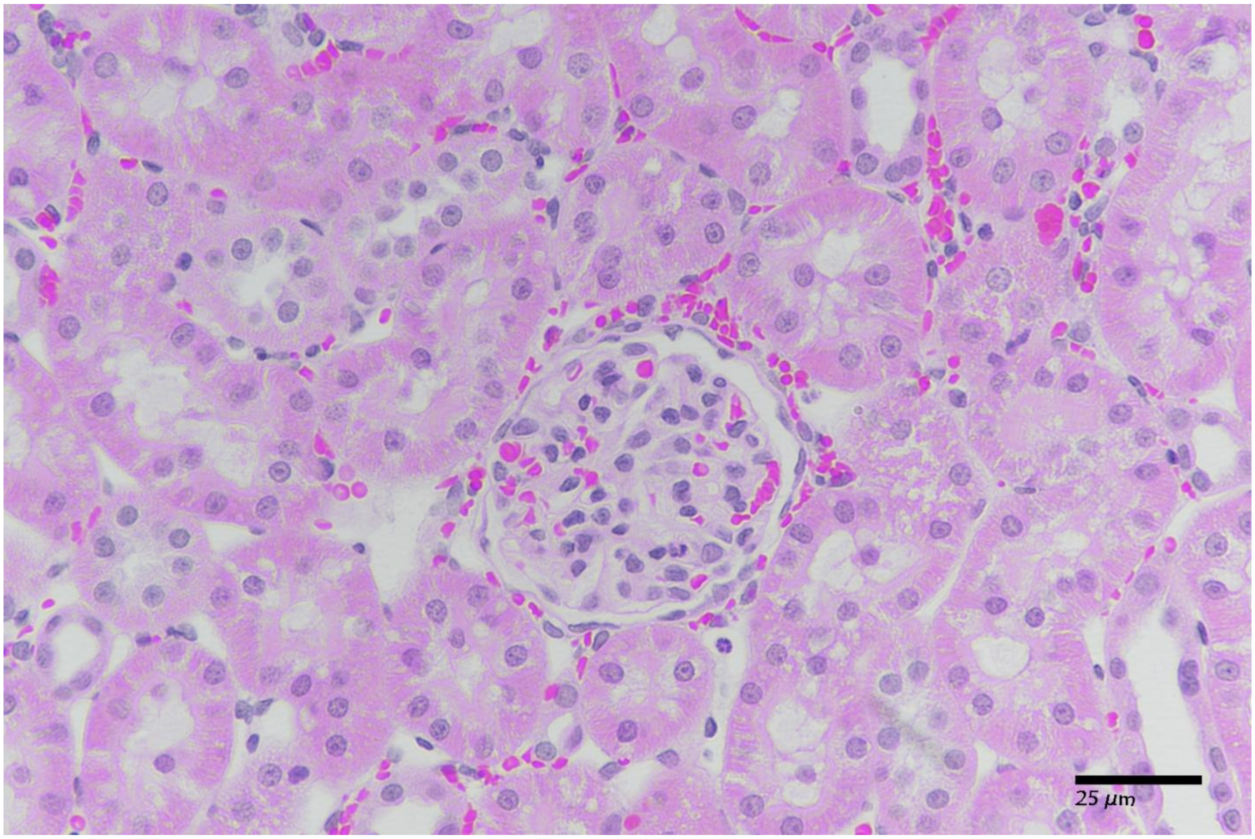
25 μm

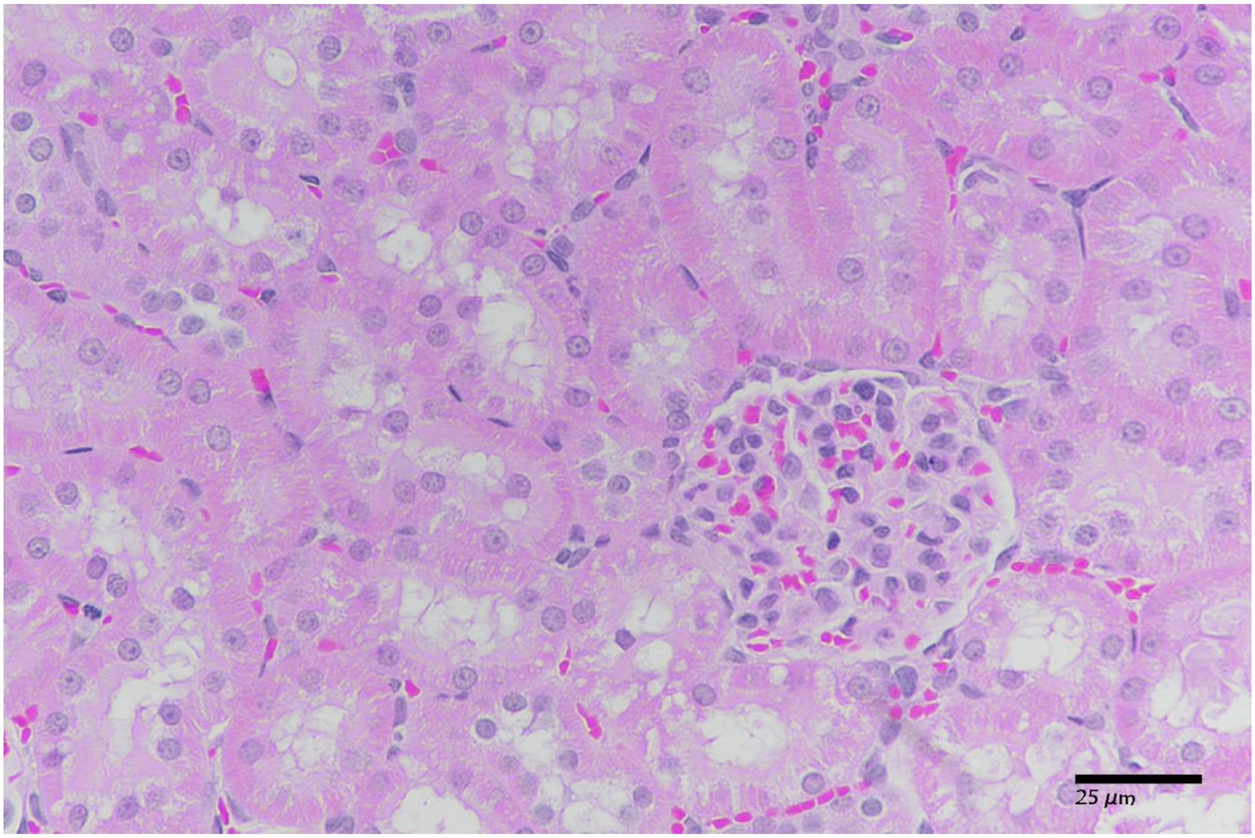
25 μm

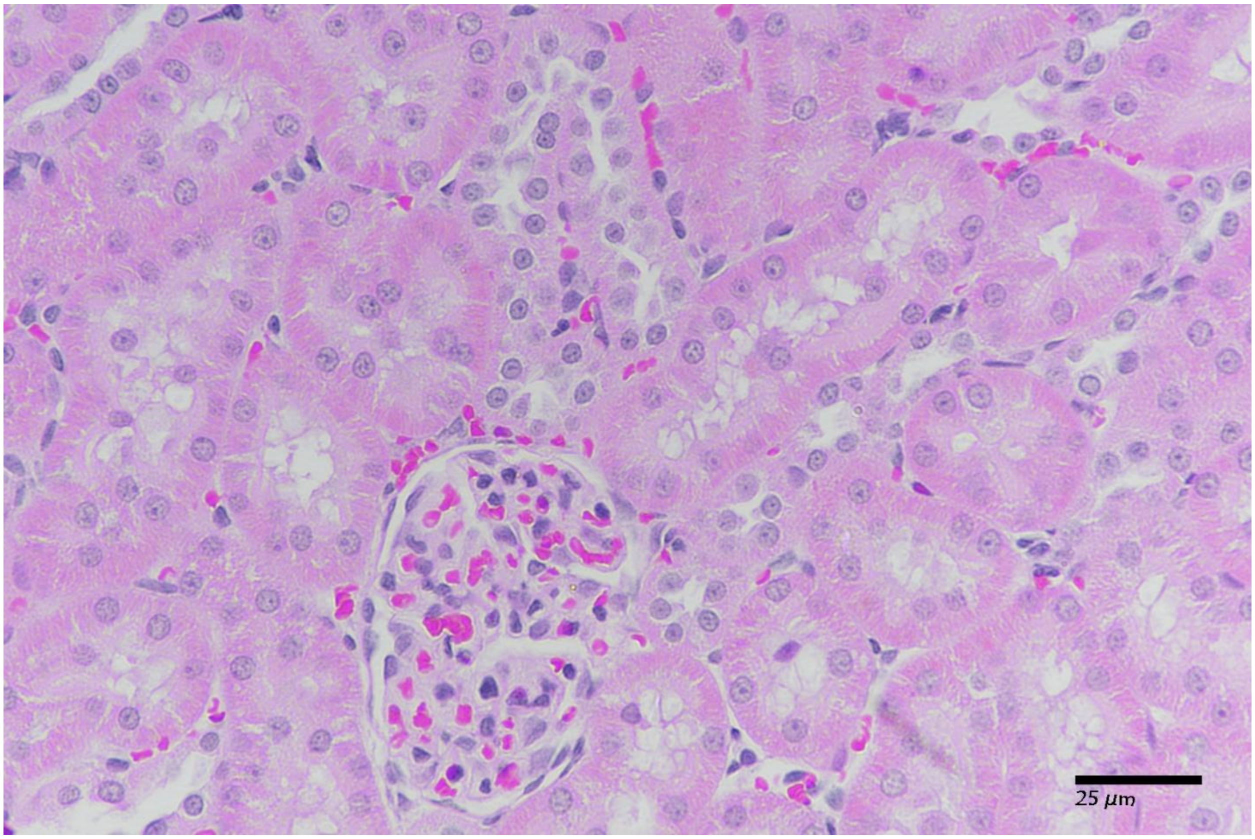
25 μm

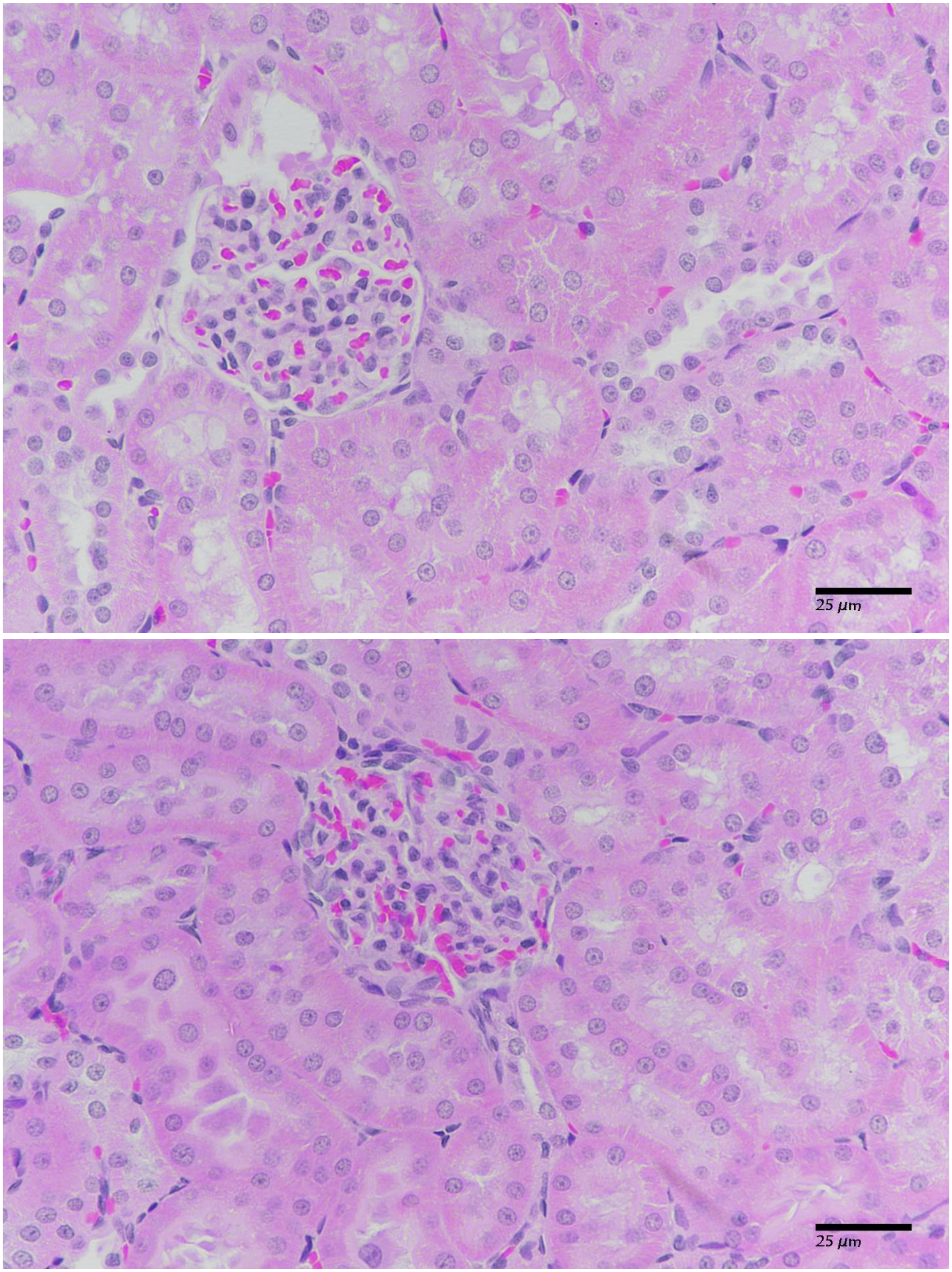
25 μm
25 μm

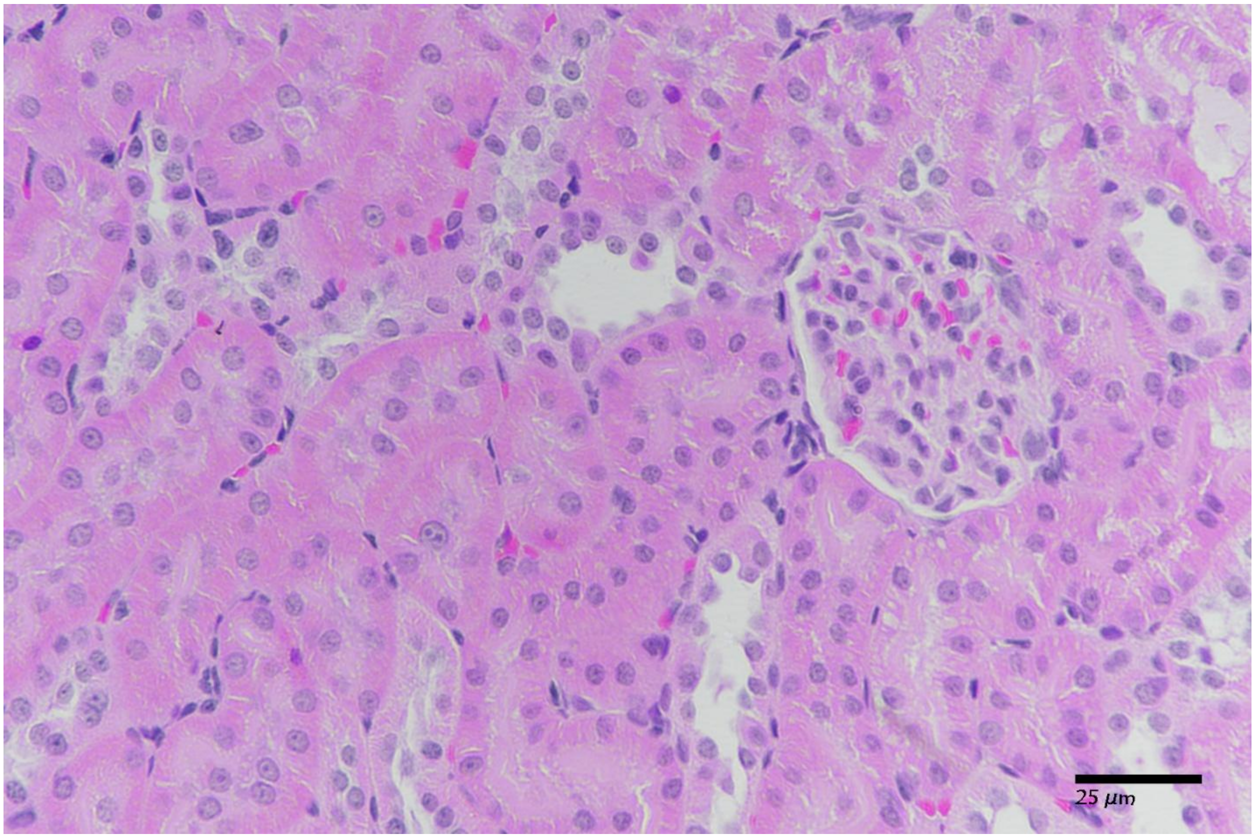
25 μm

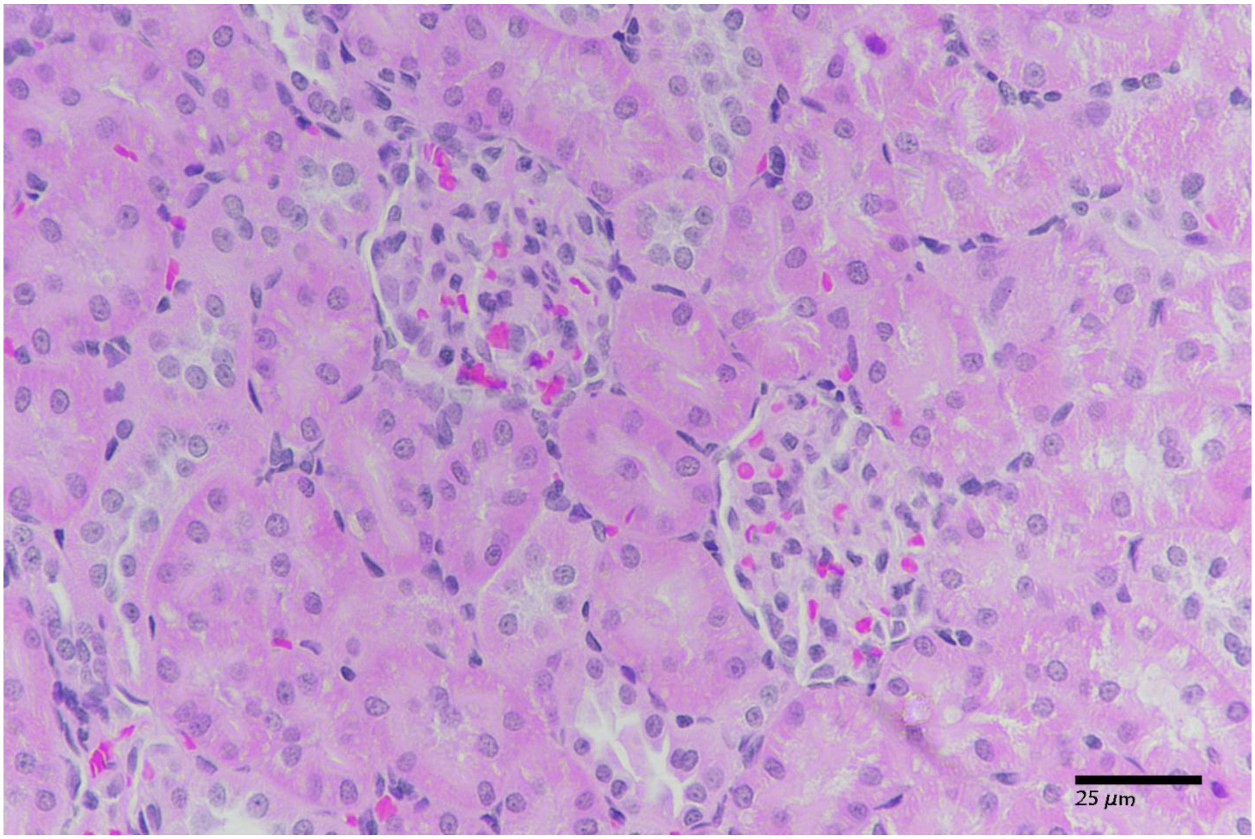
25 μm

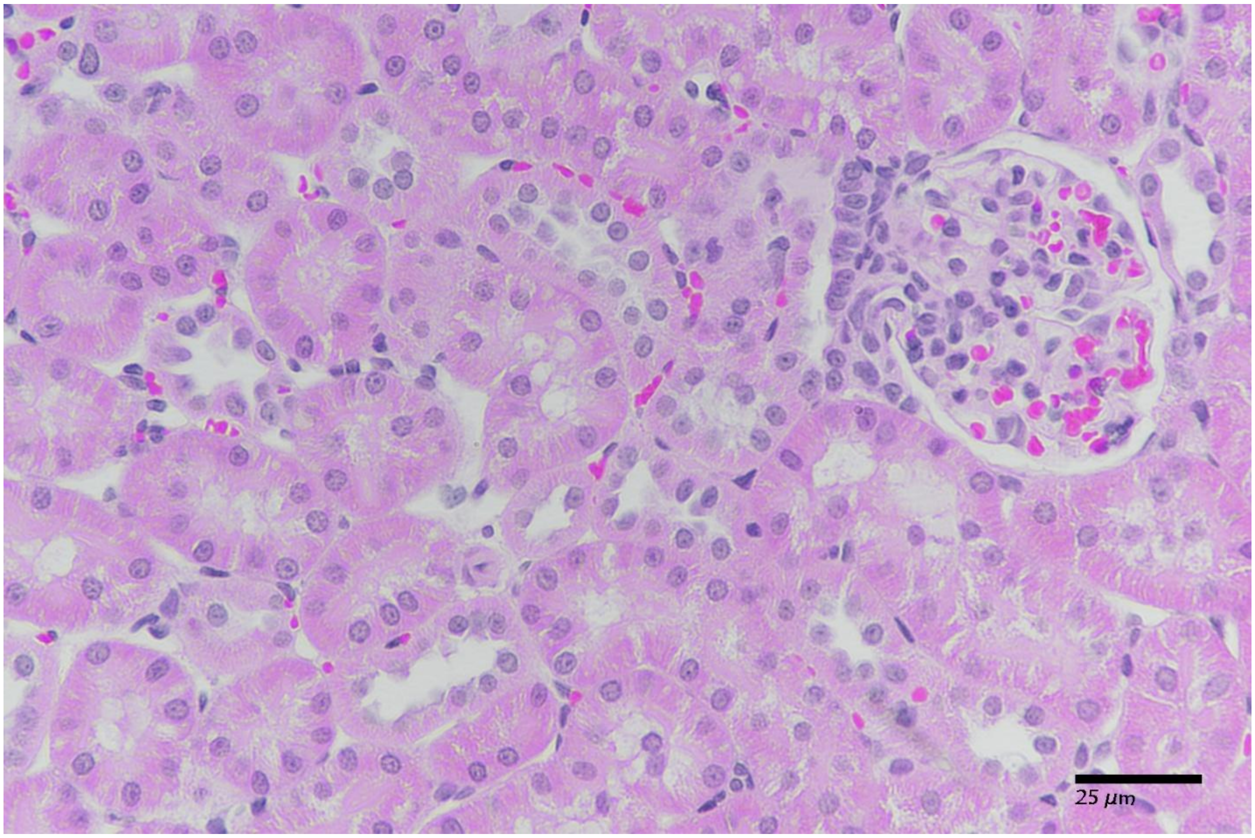
25 μm

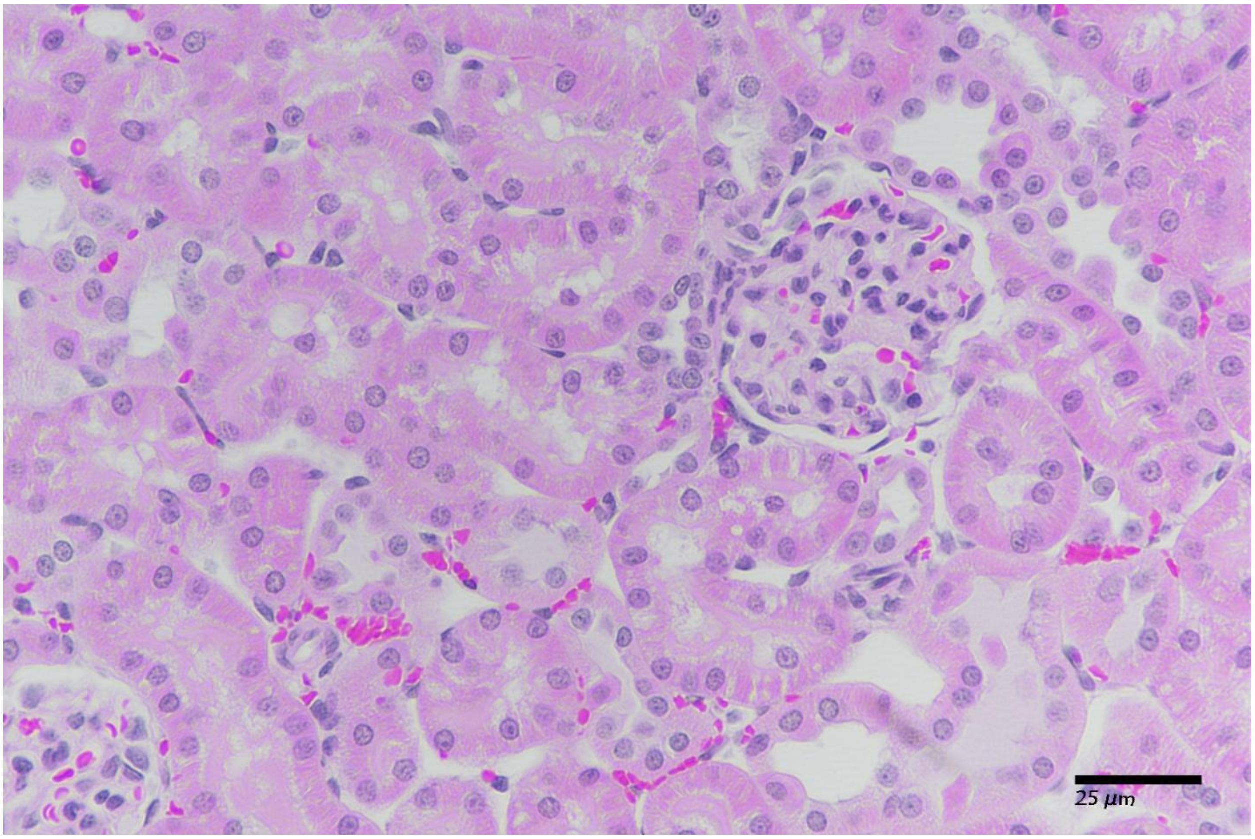
25 μm

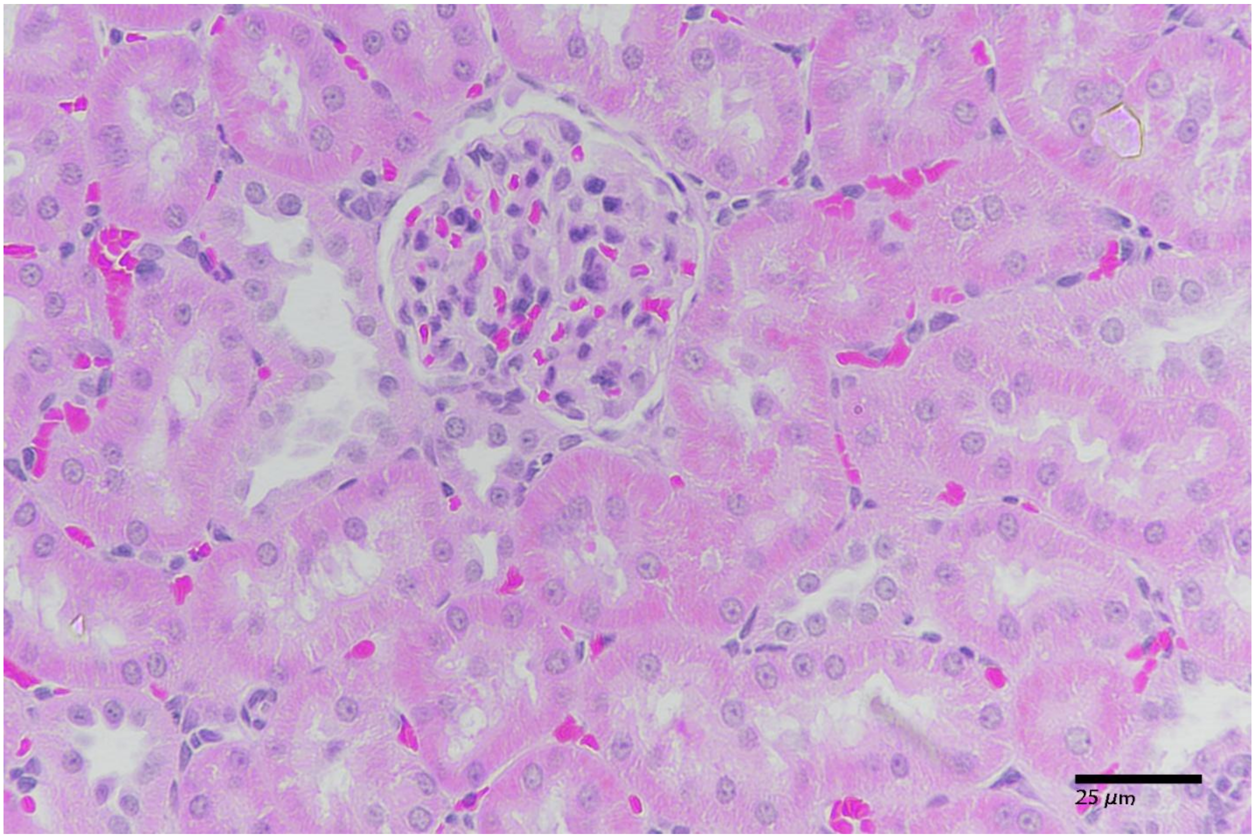
25 μm

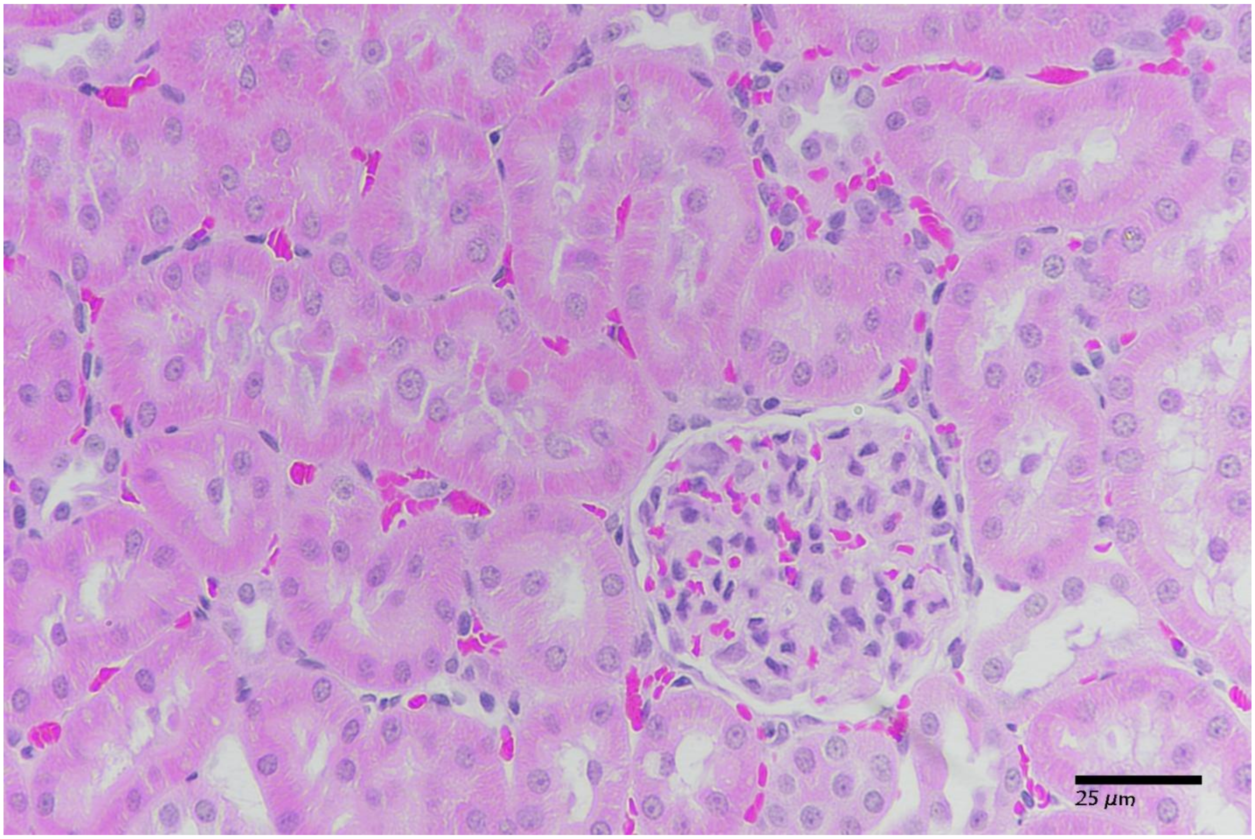
25 μm

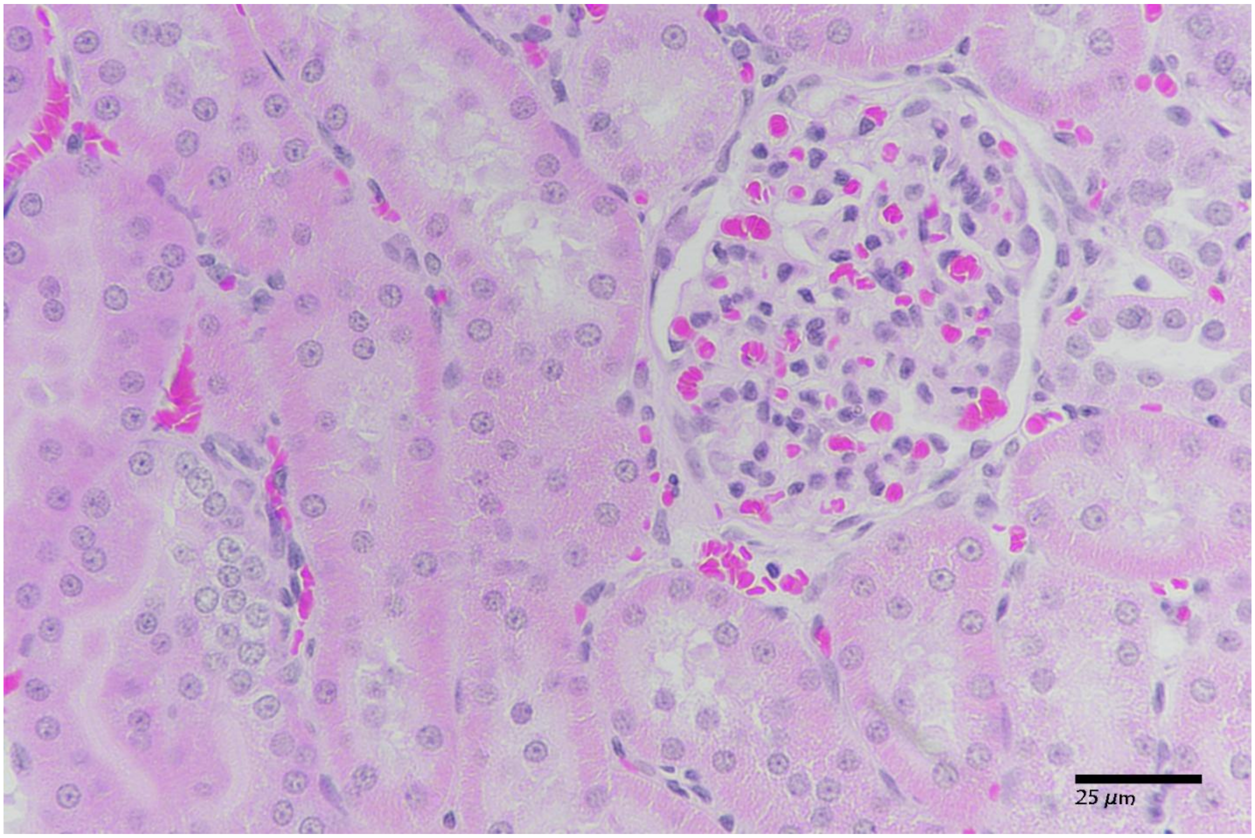
25 μm

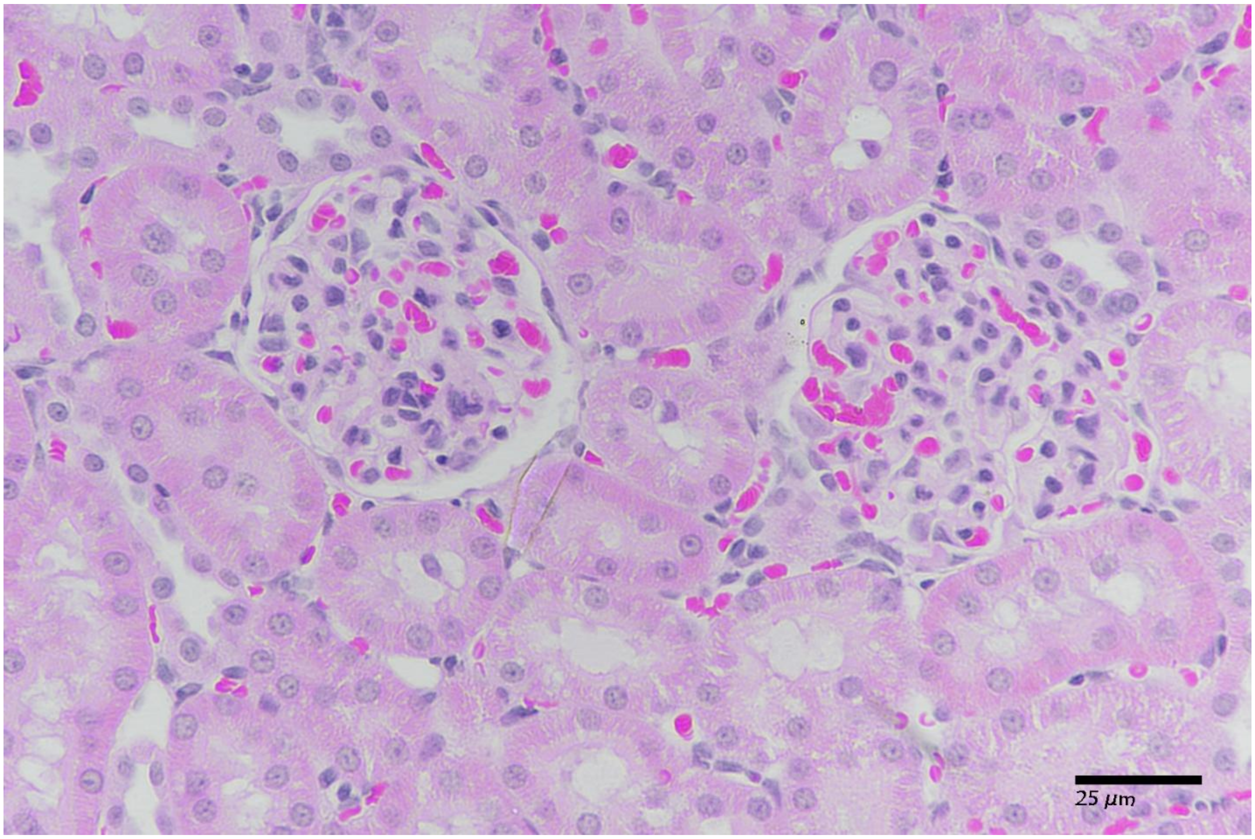
25 μm

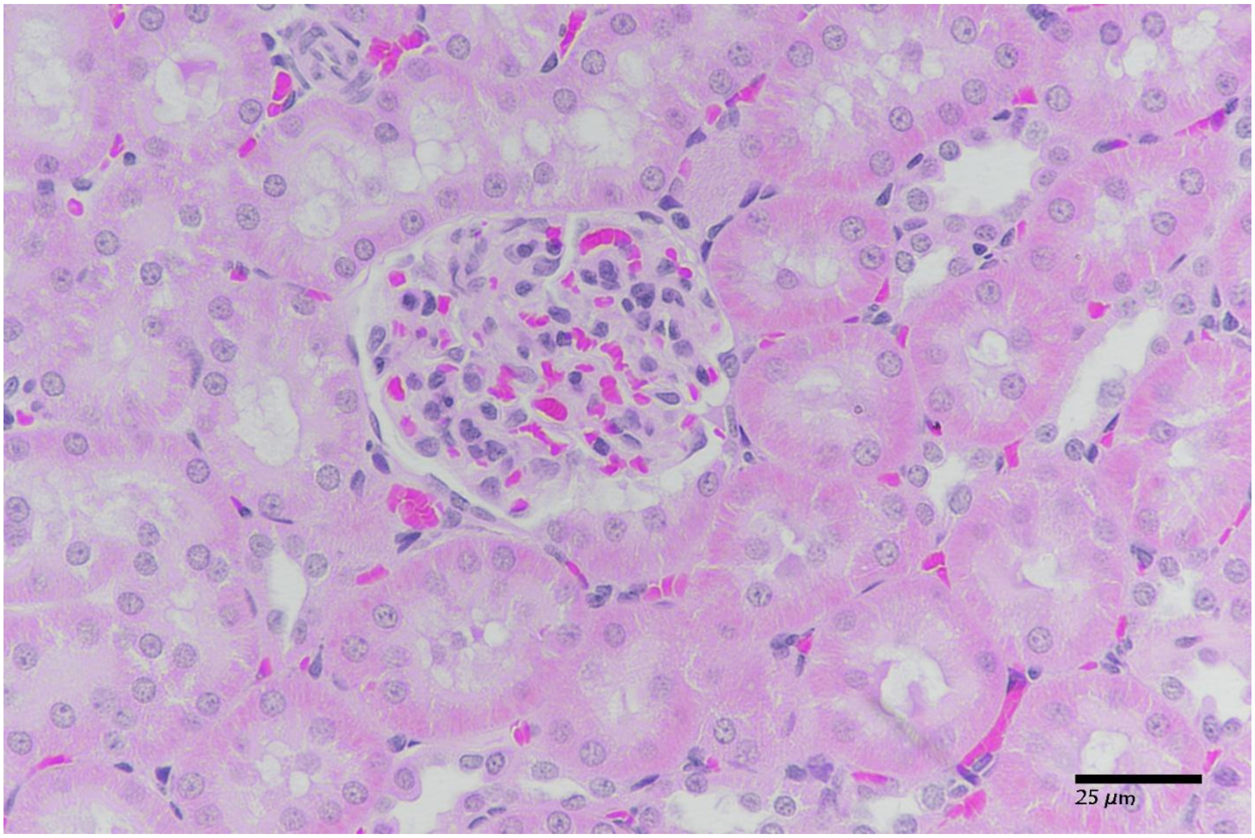
25 μm

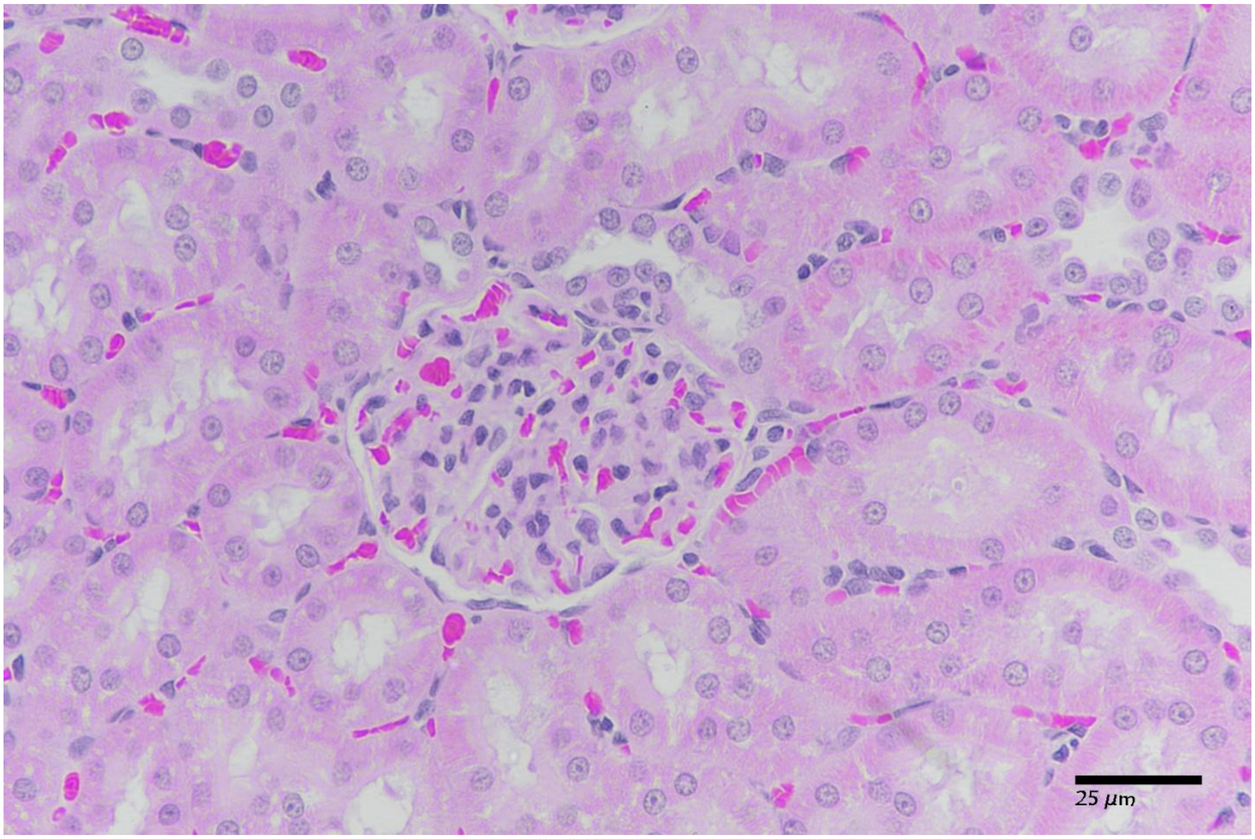
25 μm

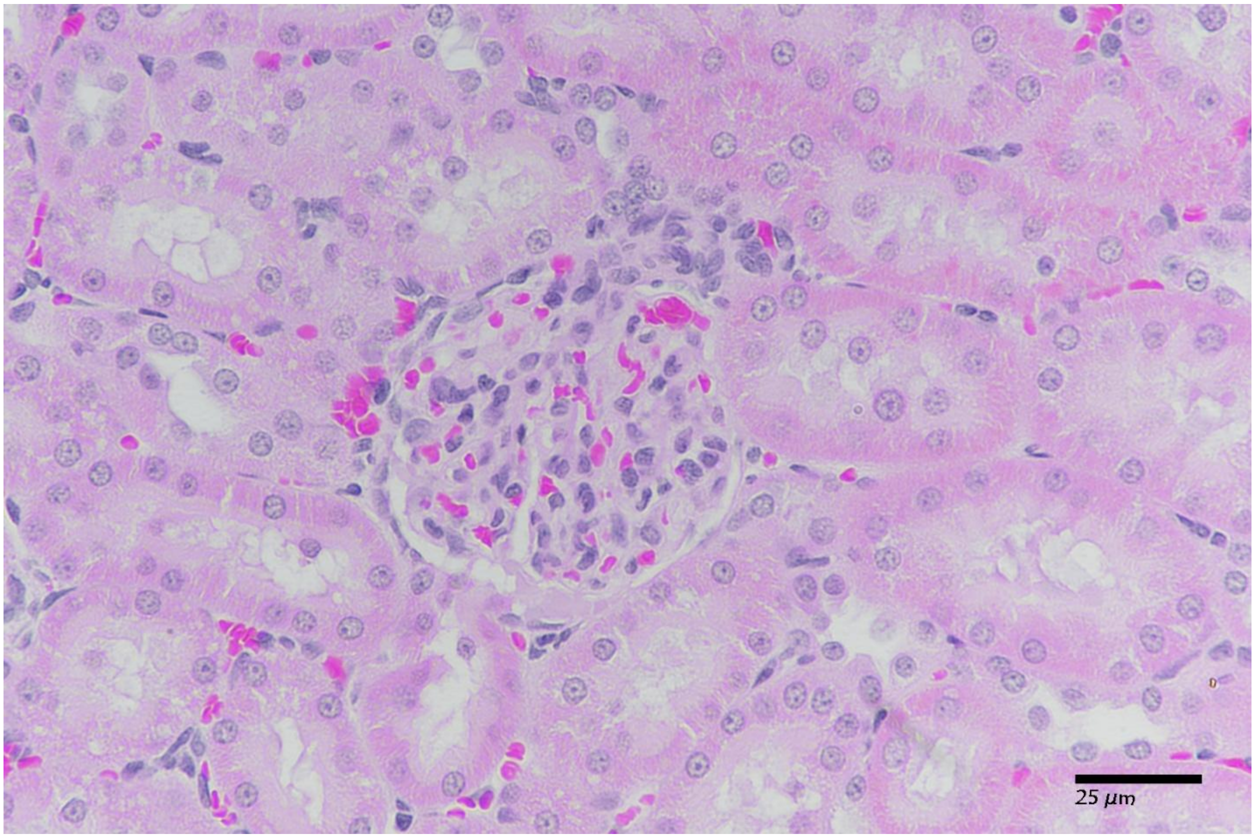
25 μm

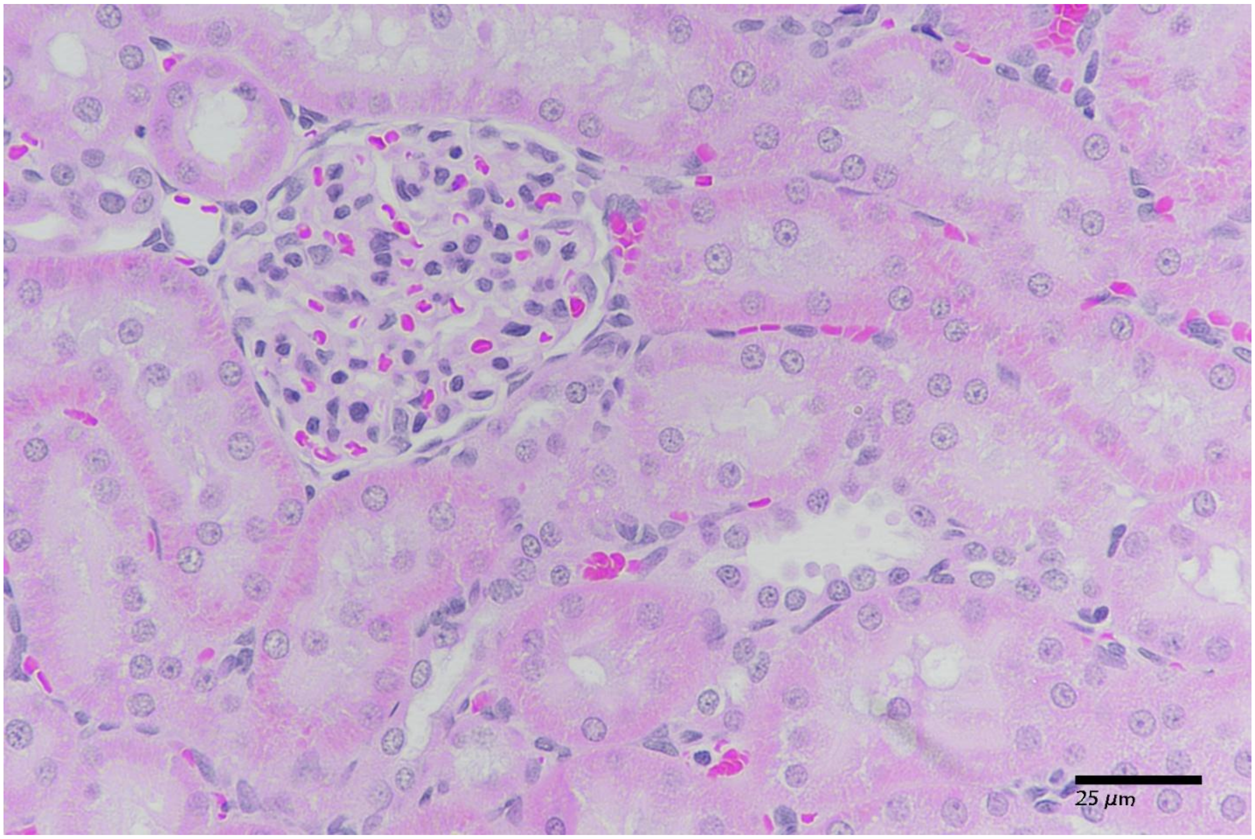
25 μm

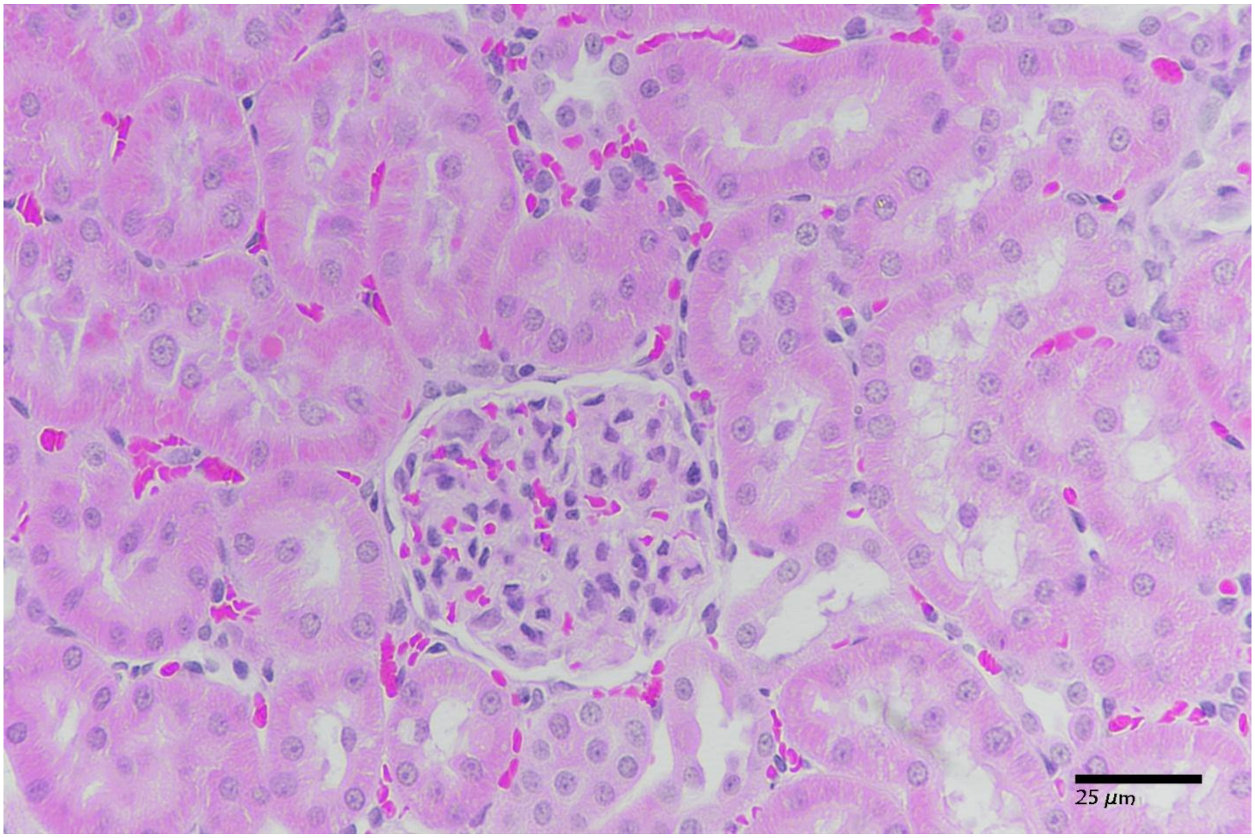
25 μm

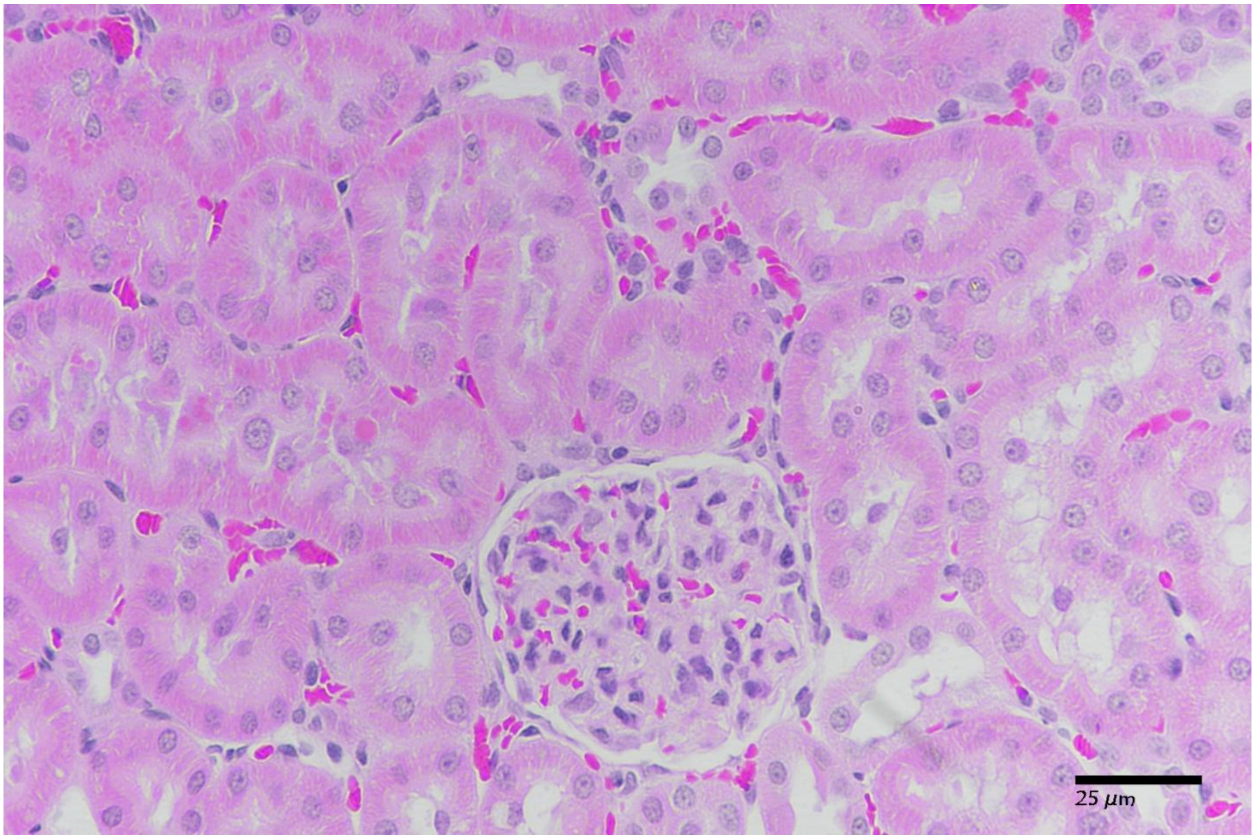
25 μm

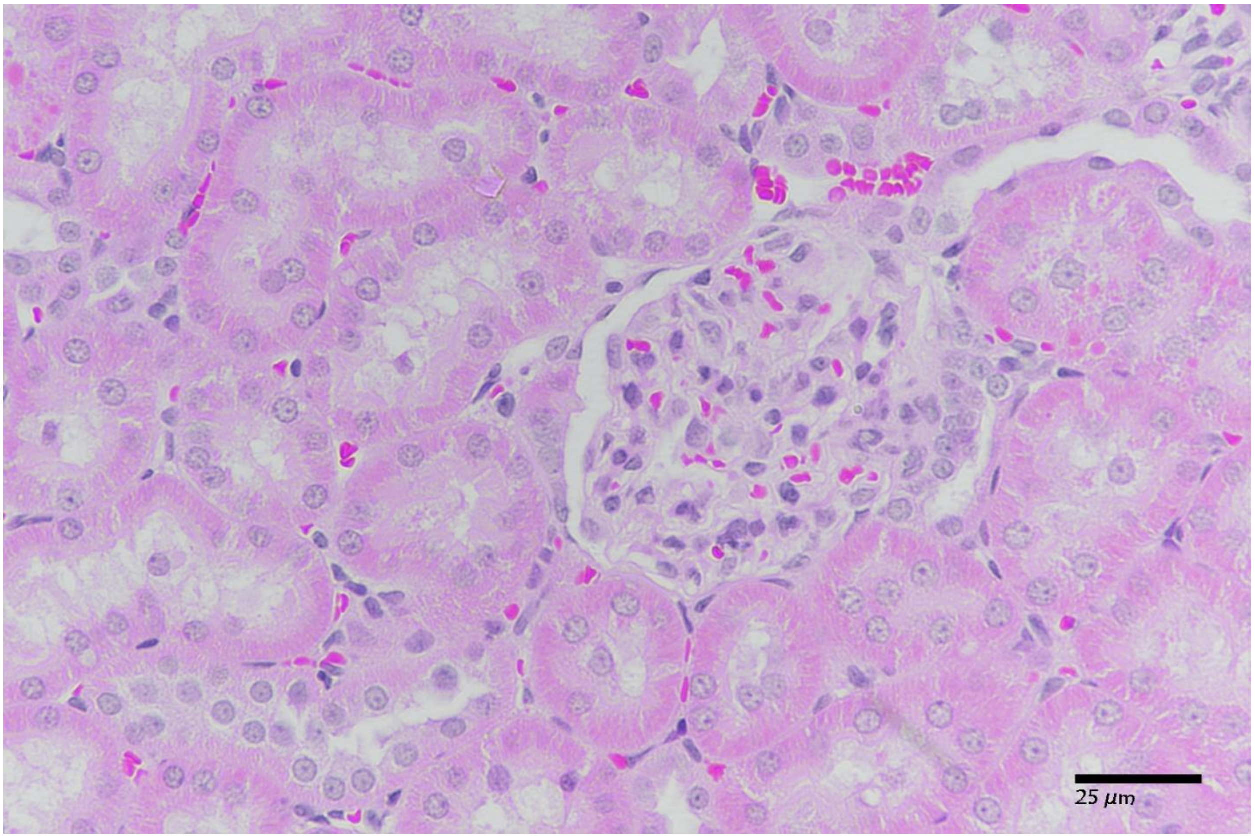
25 μm

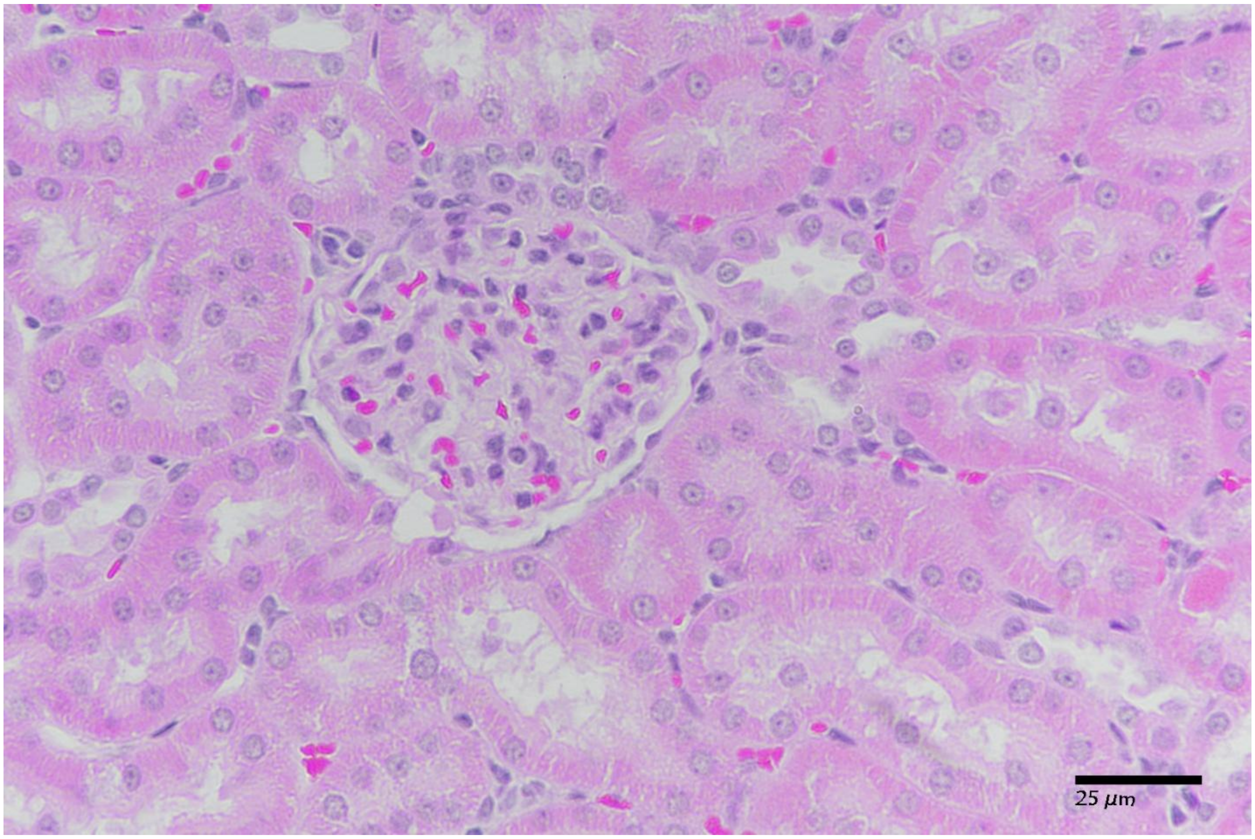
25 μm

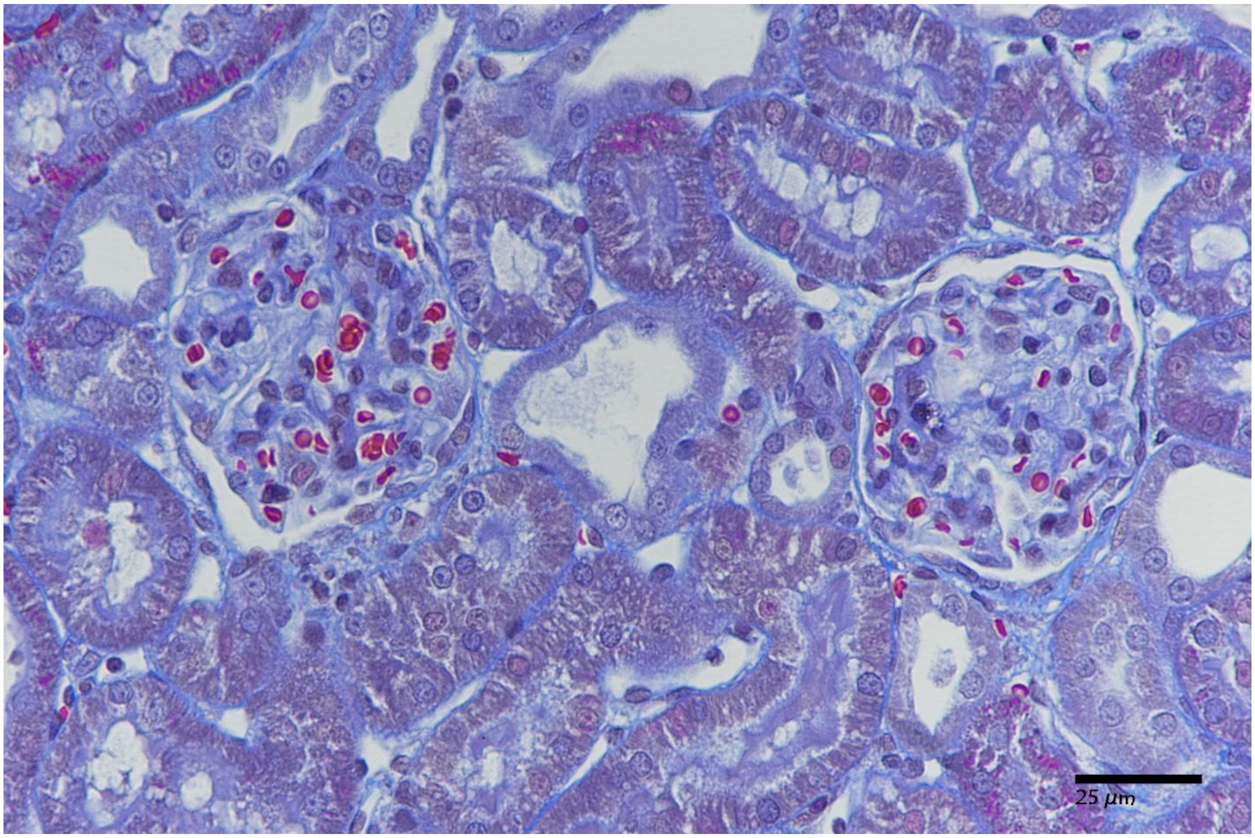
25 μm

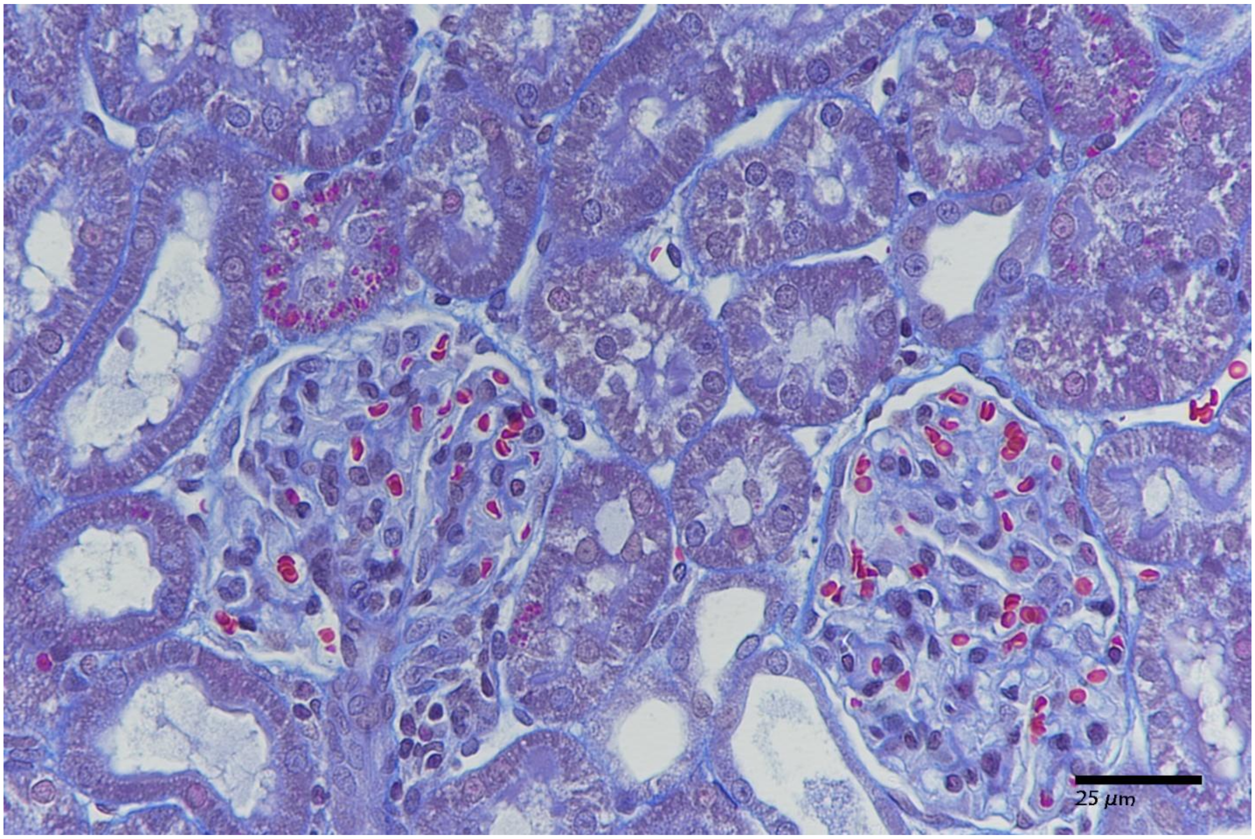
25 μm

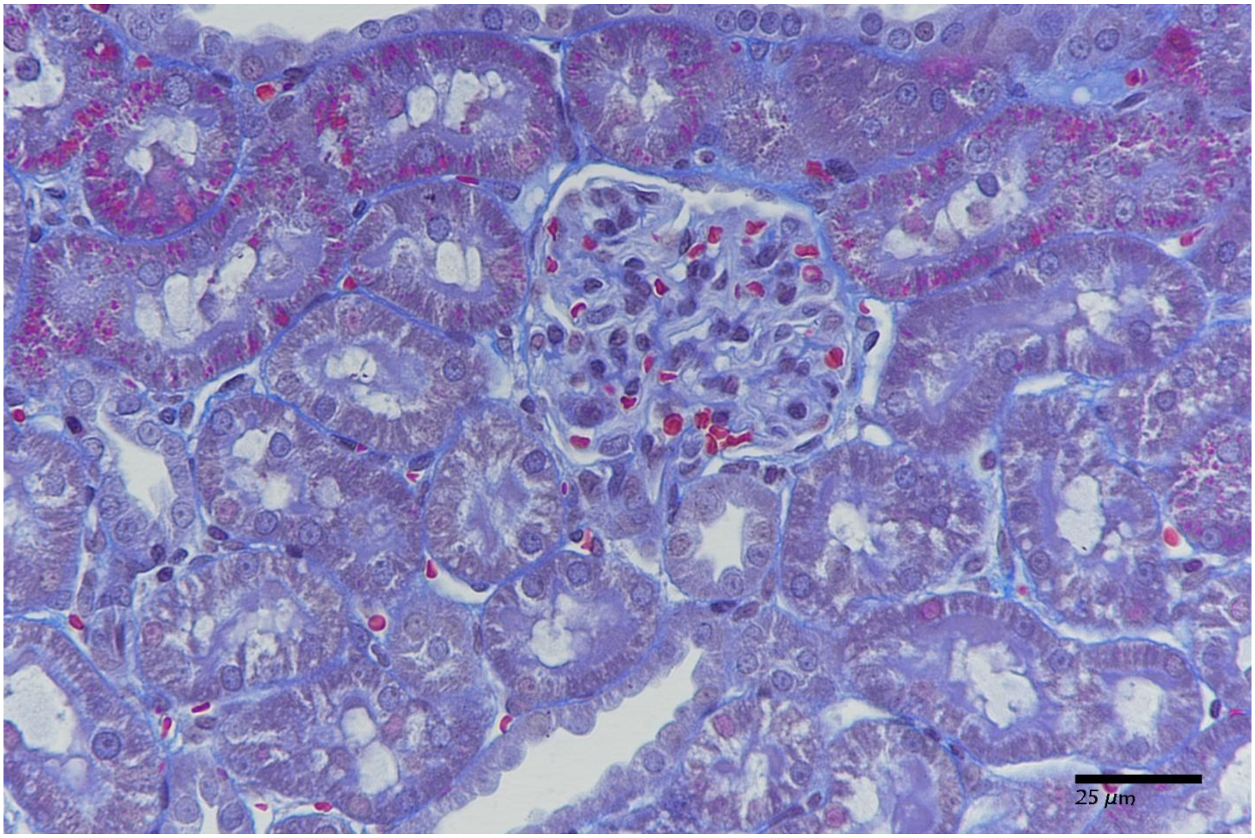
25 μm

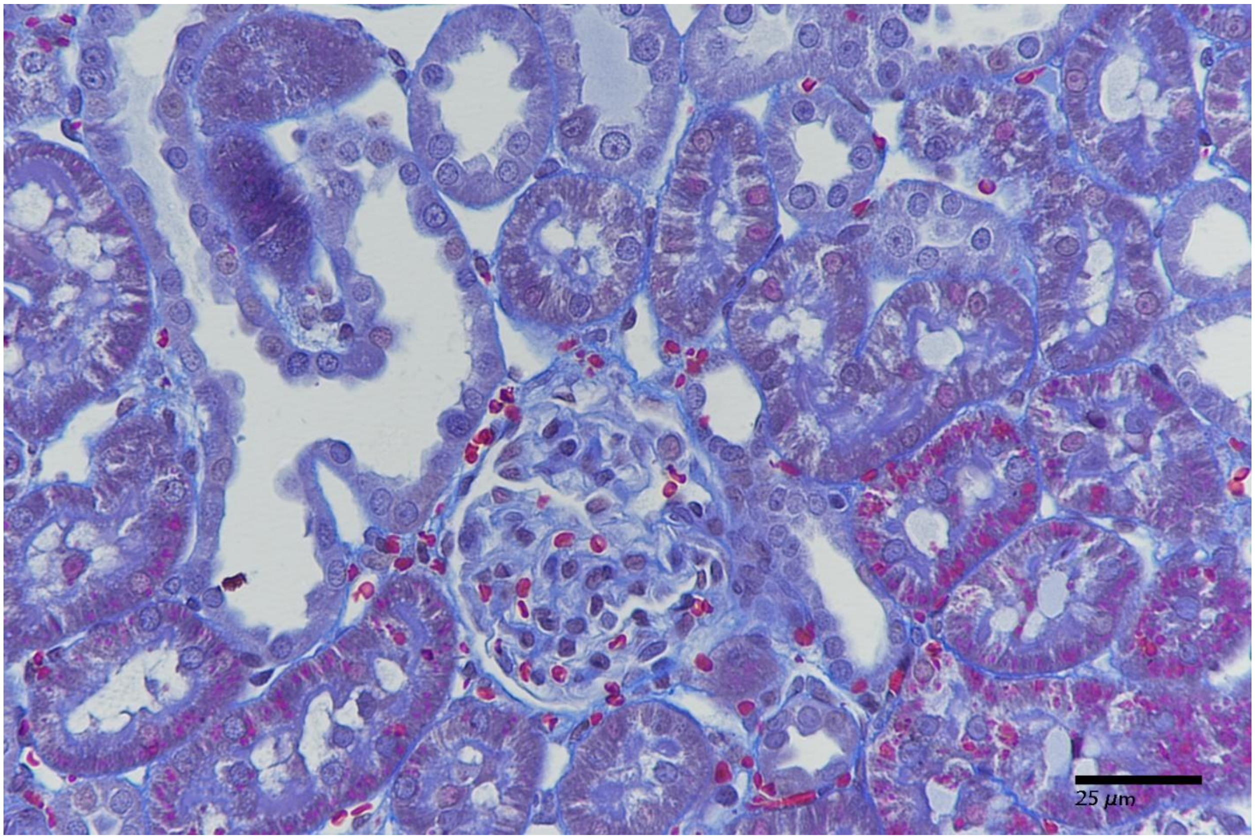
25 μm

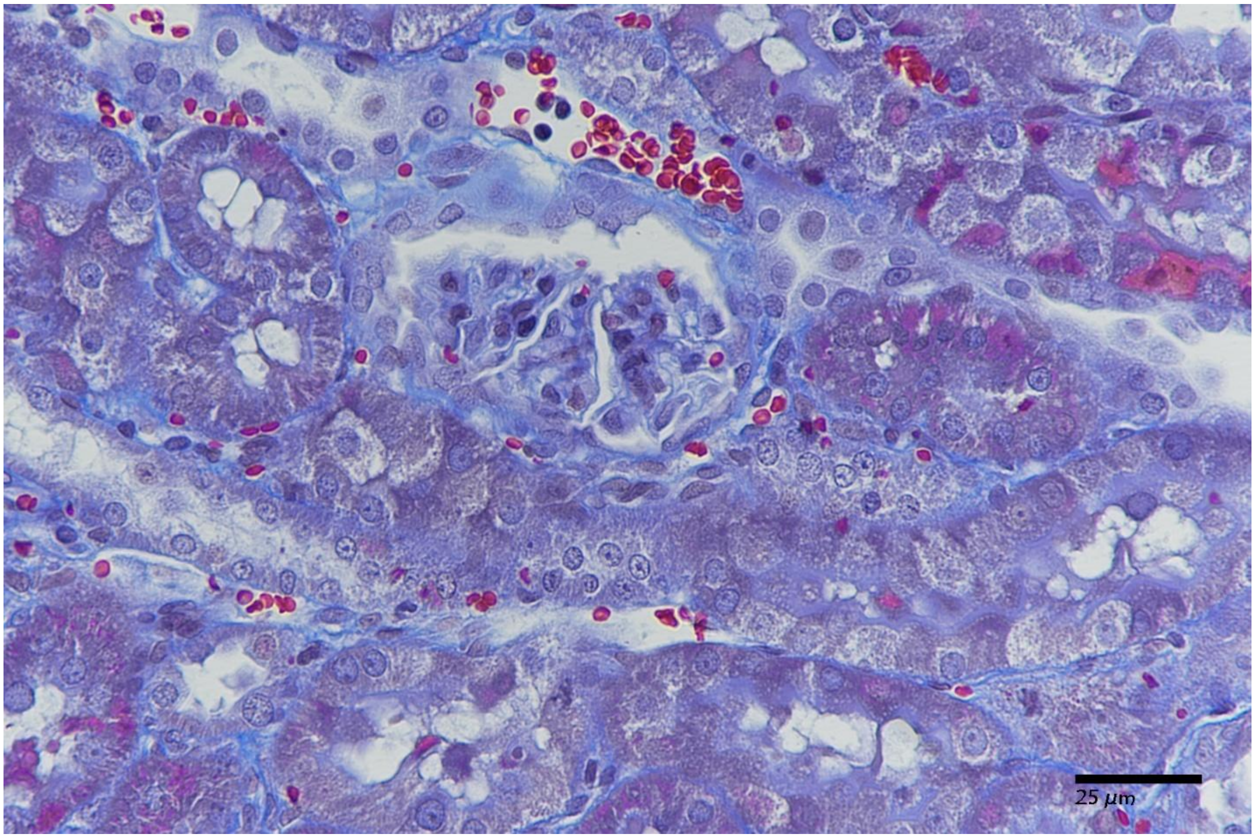
25 μm

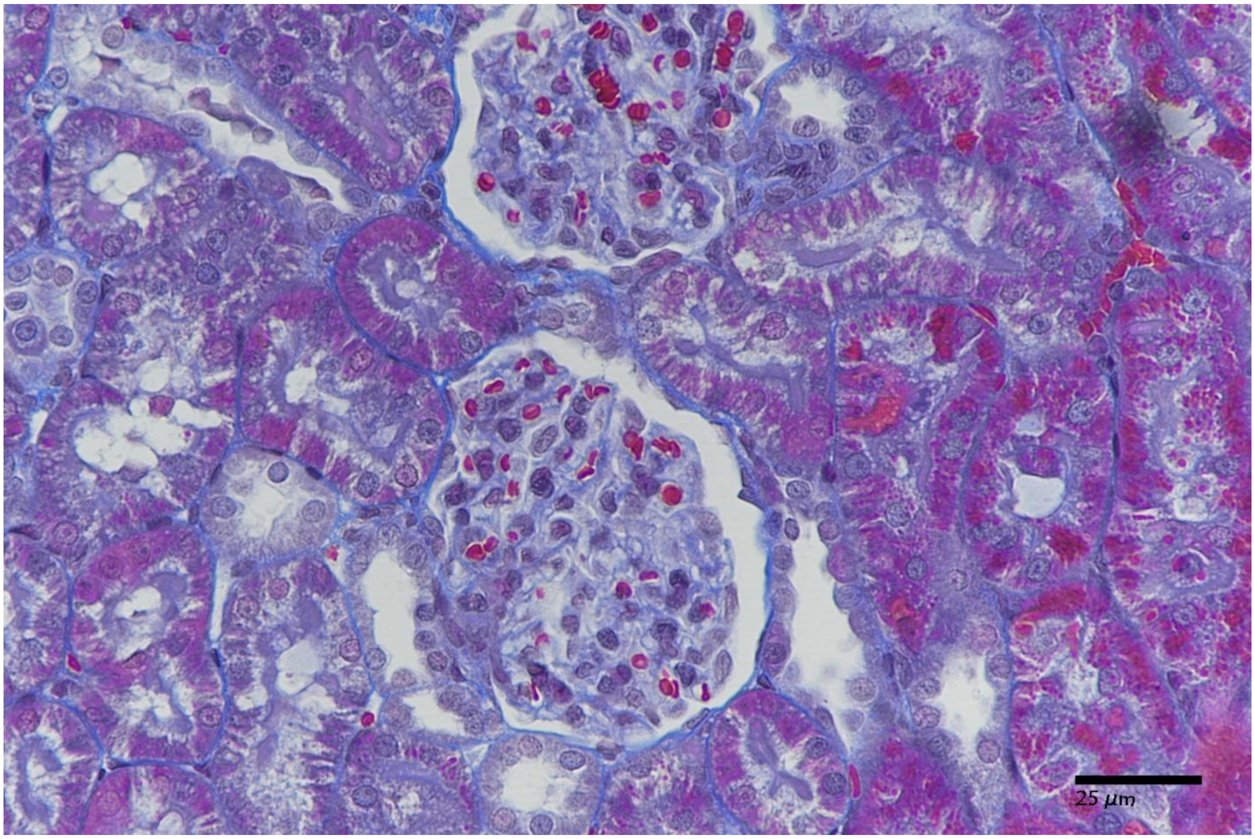
25 μm

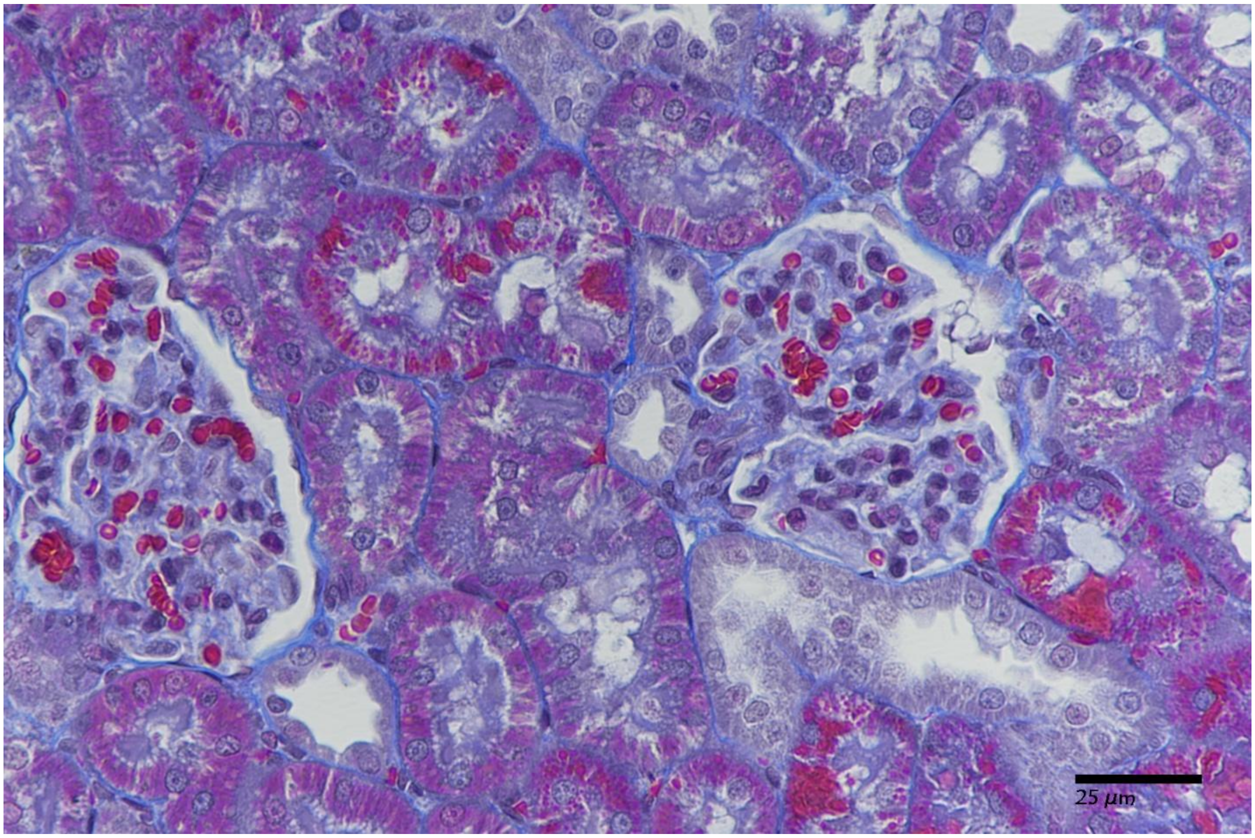
25 µm

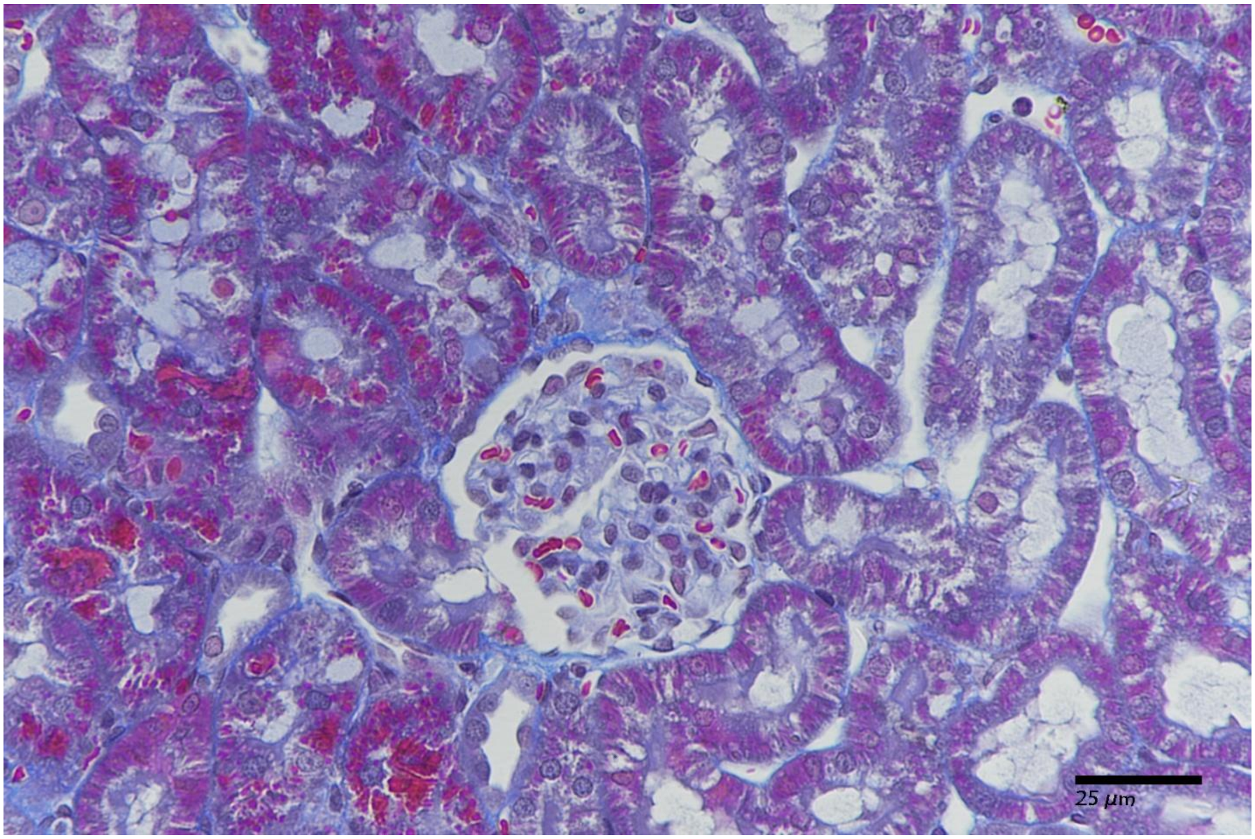
25 µm

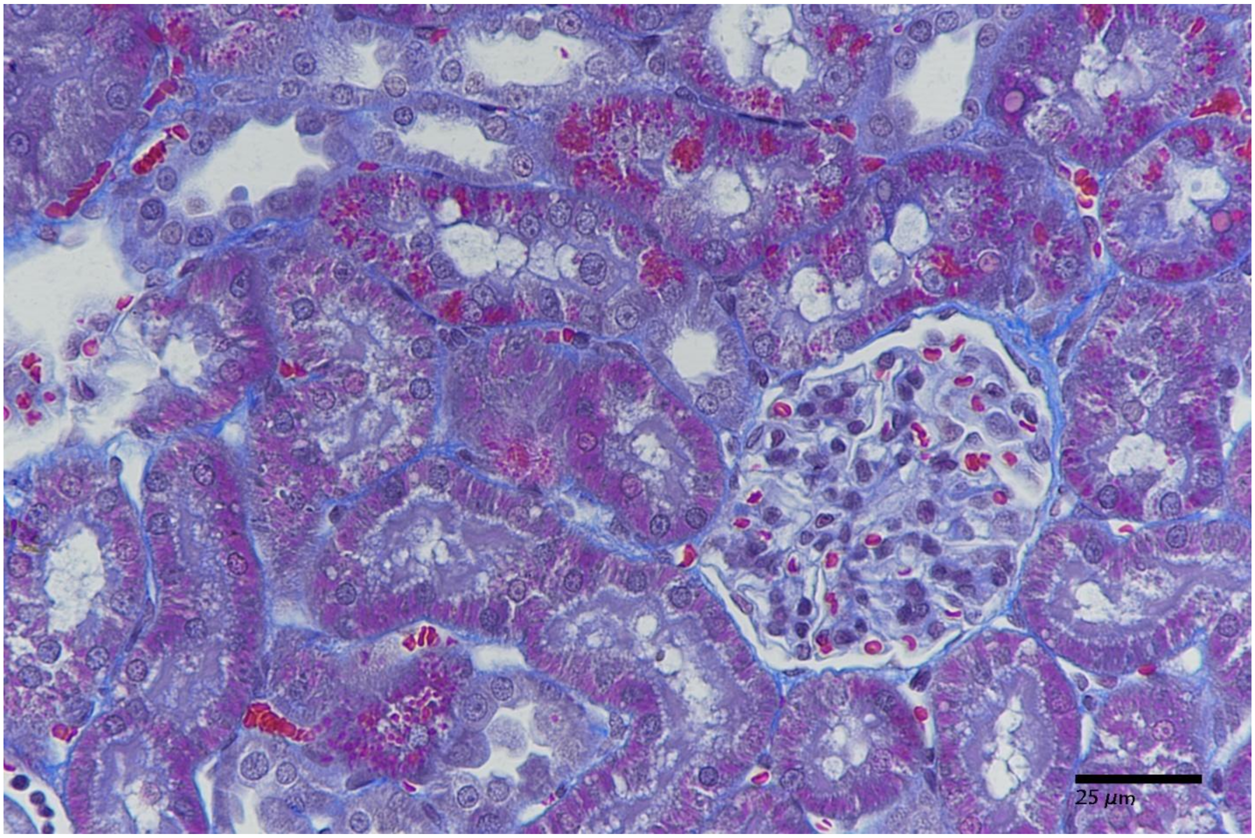
25 μm

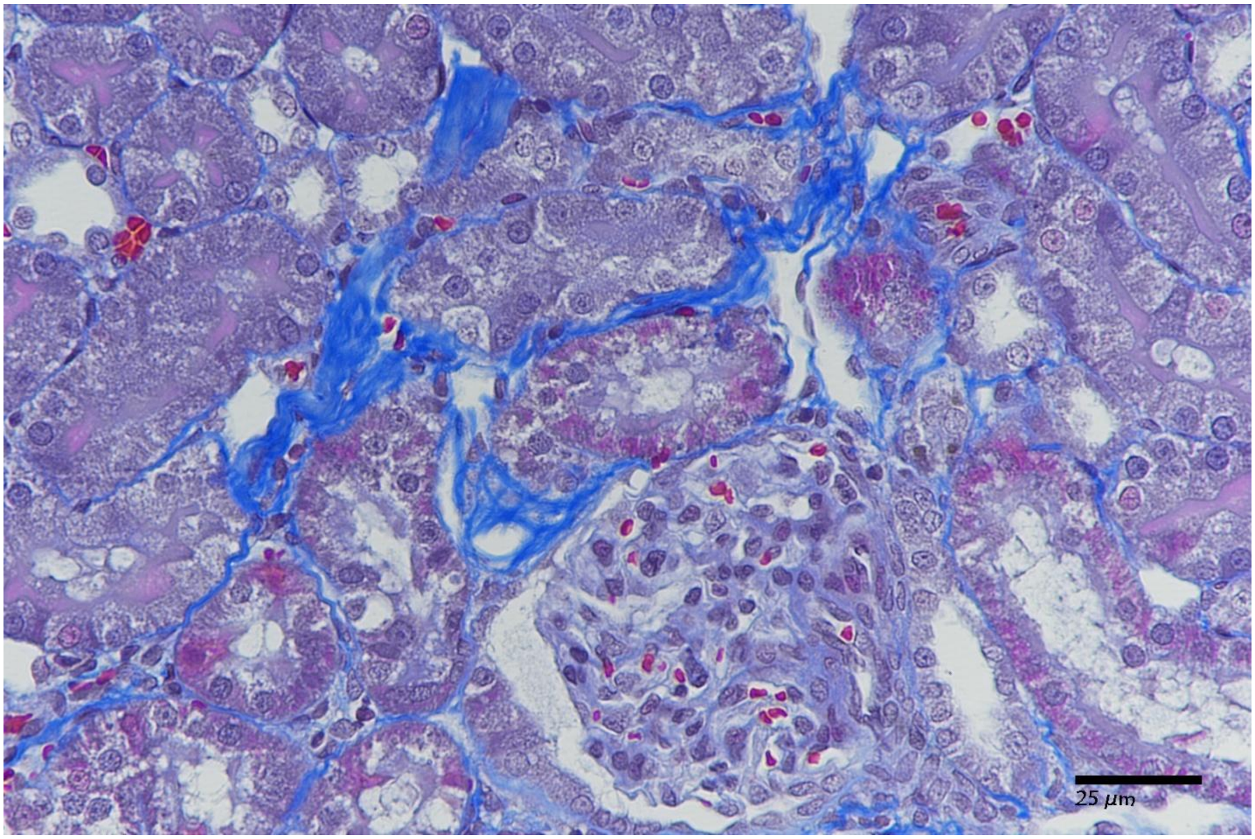
25 μm

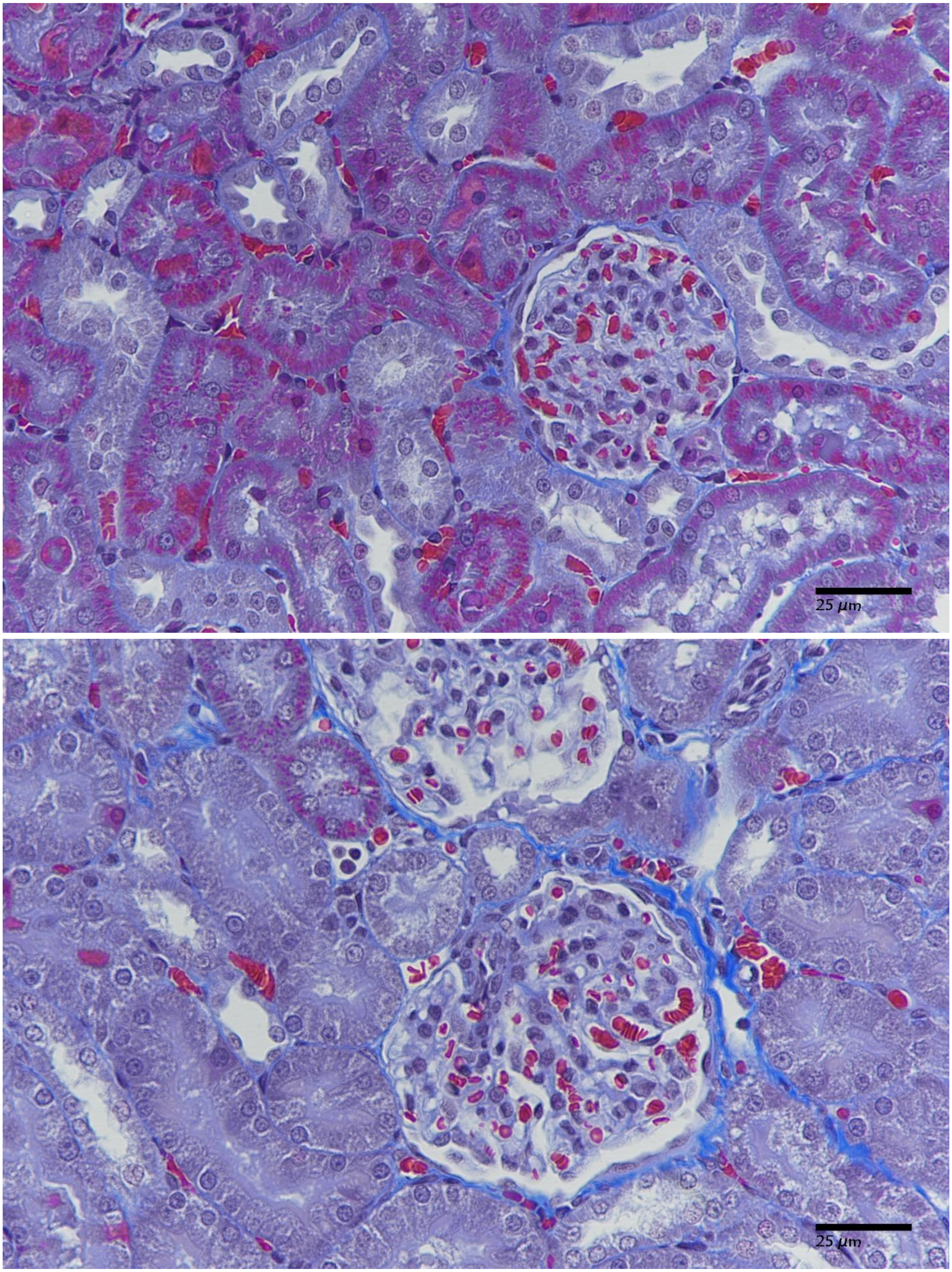
25 μm
25 μm

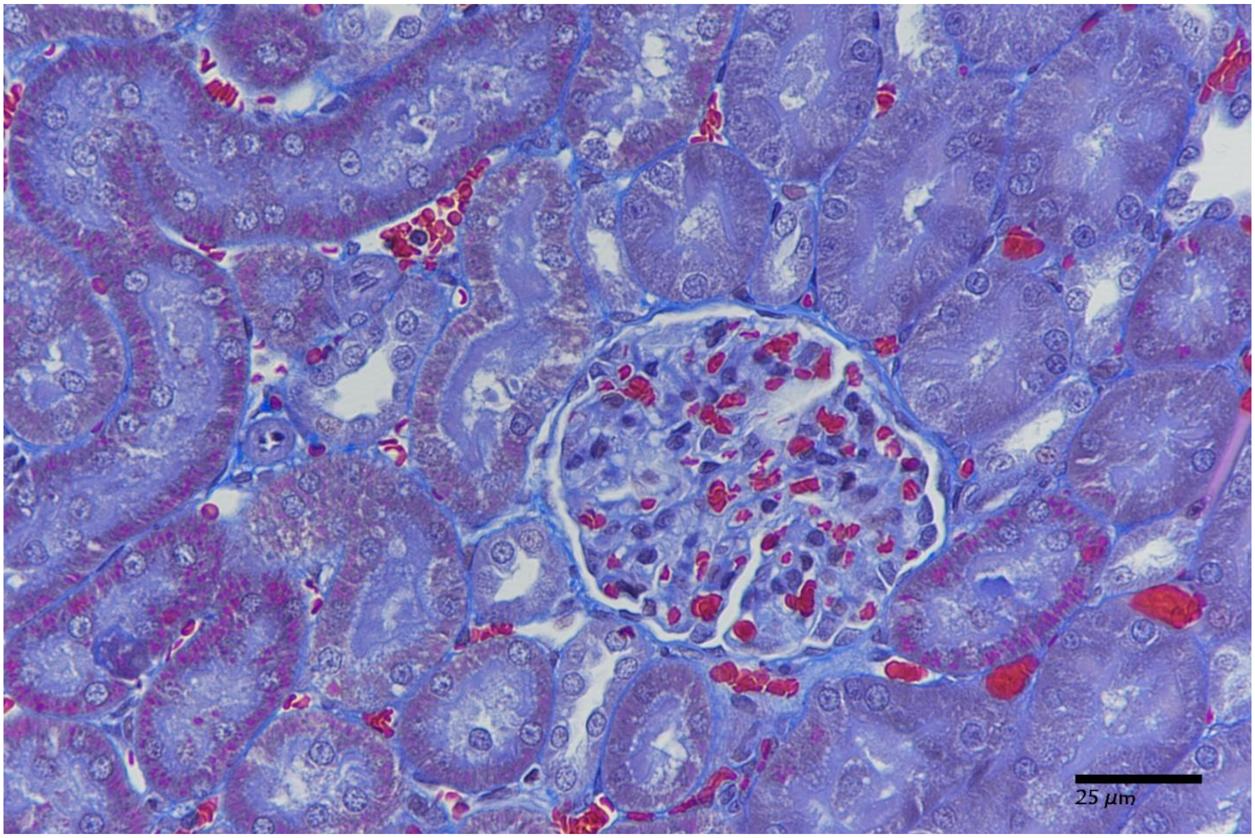
25 μm

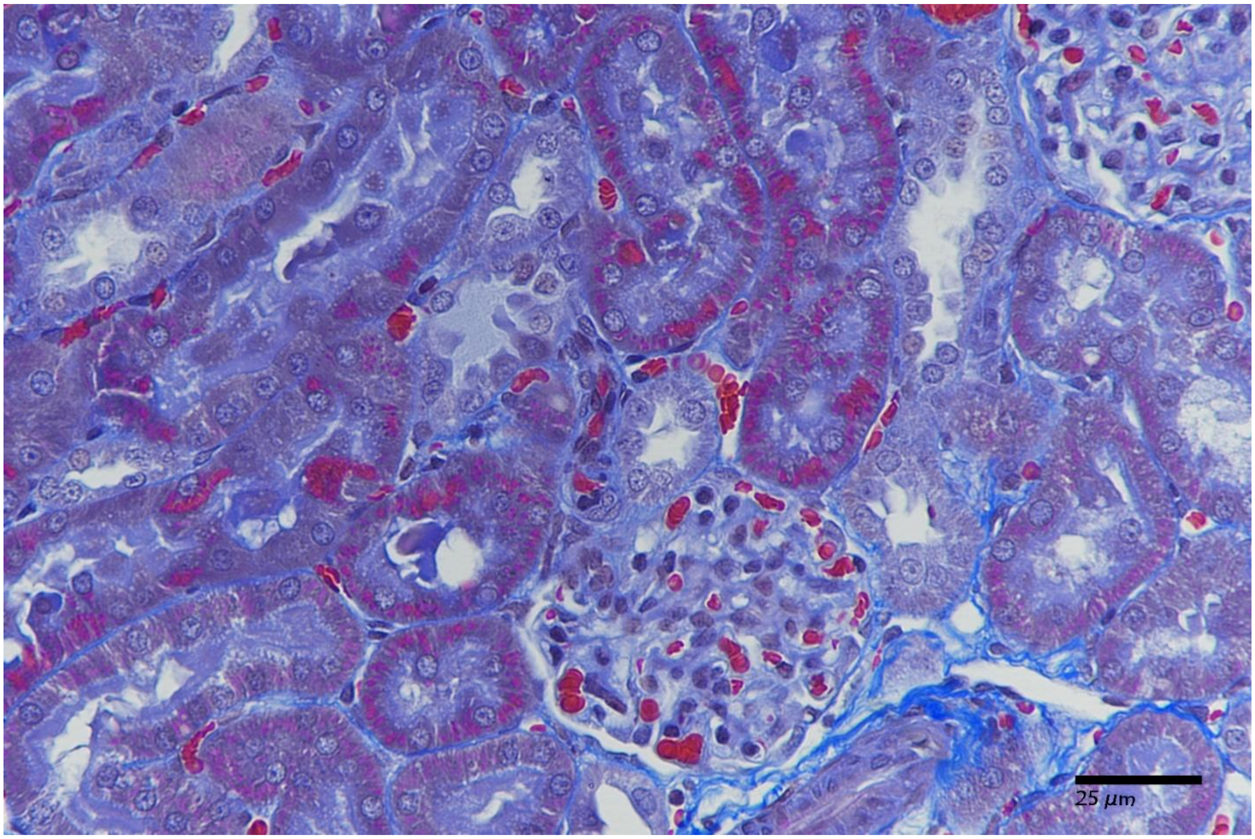
25 μm

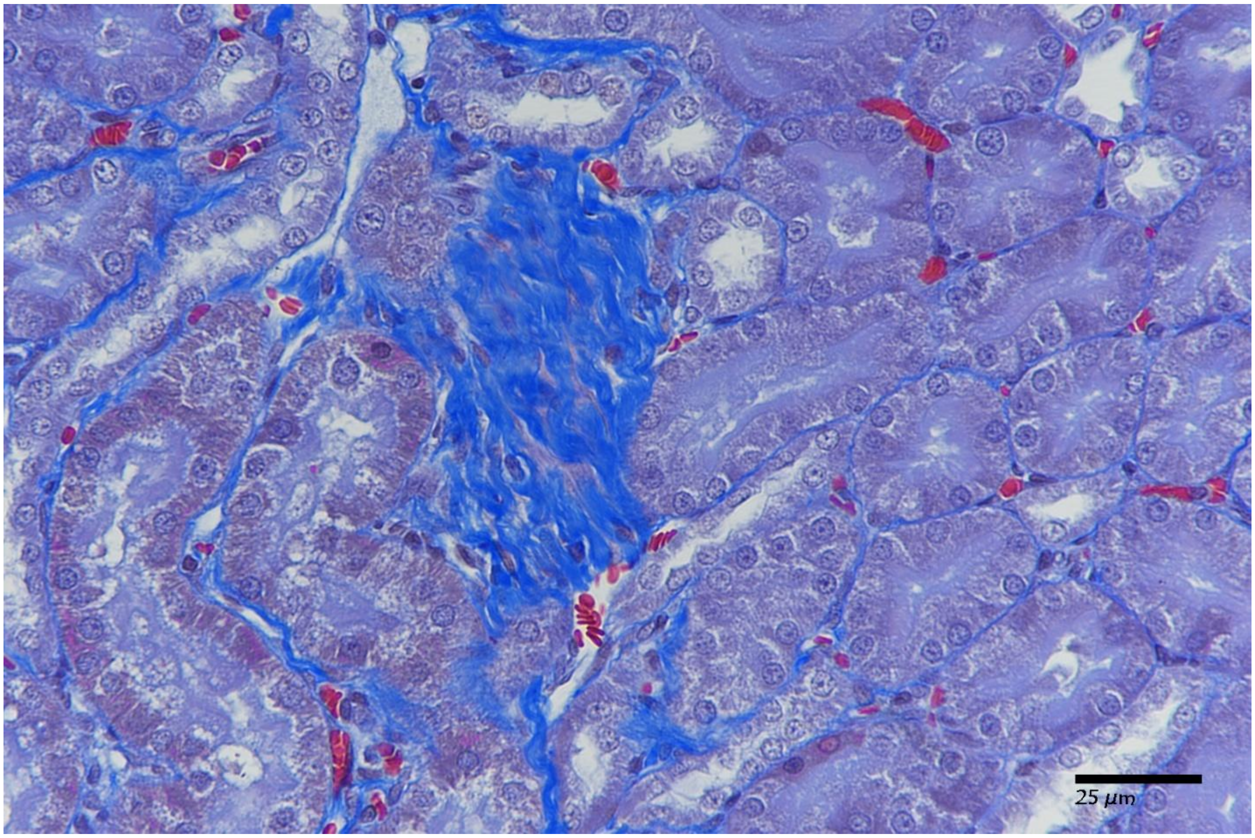
25 μm

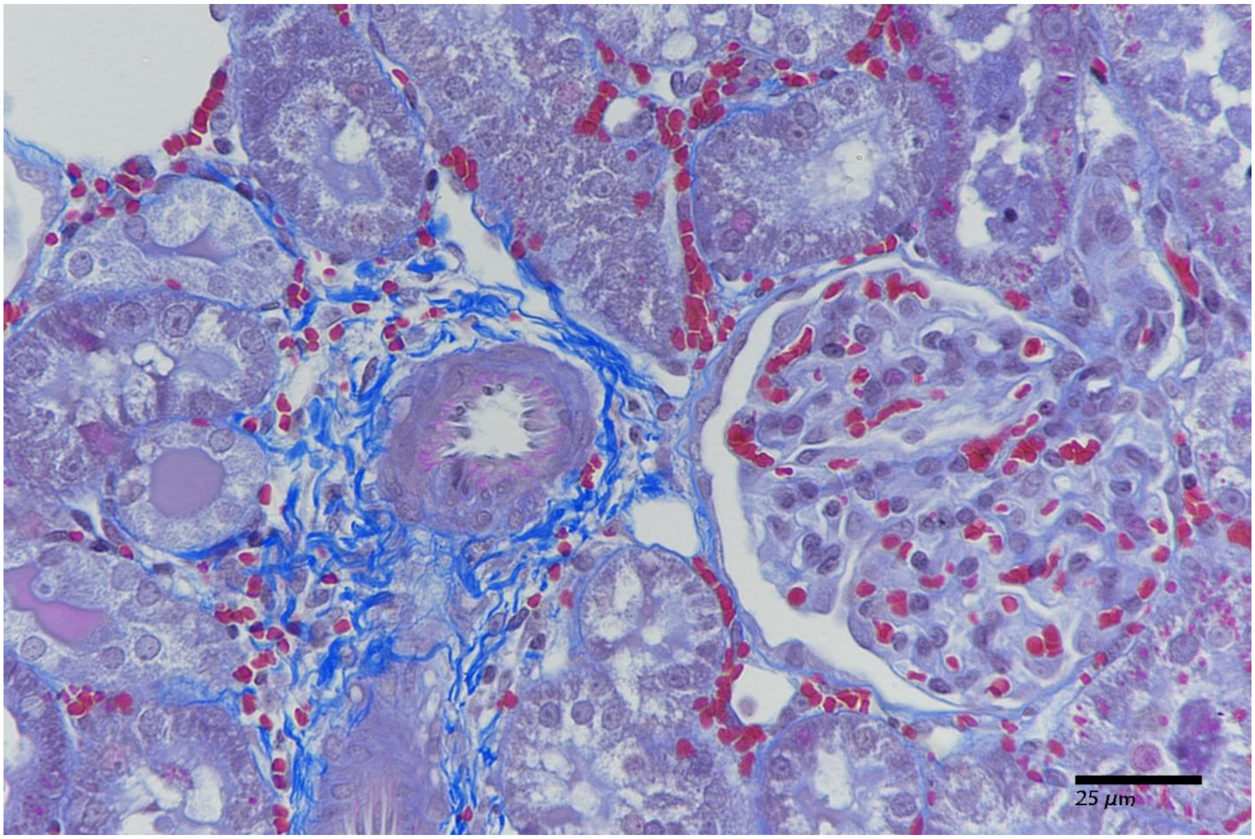
25 μm

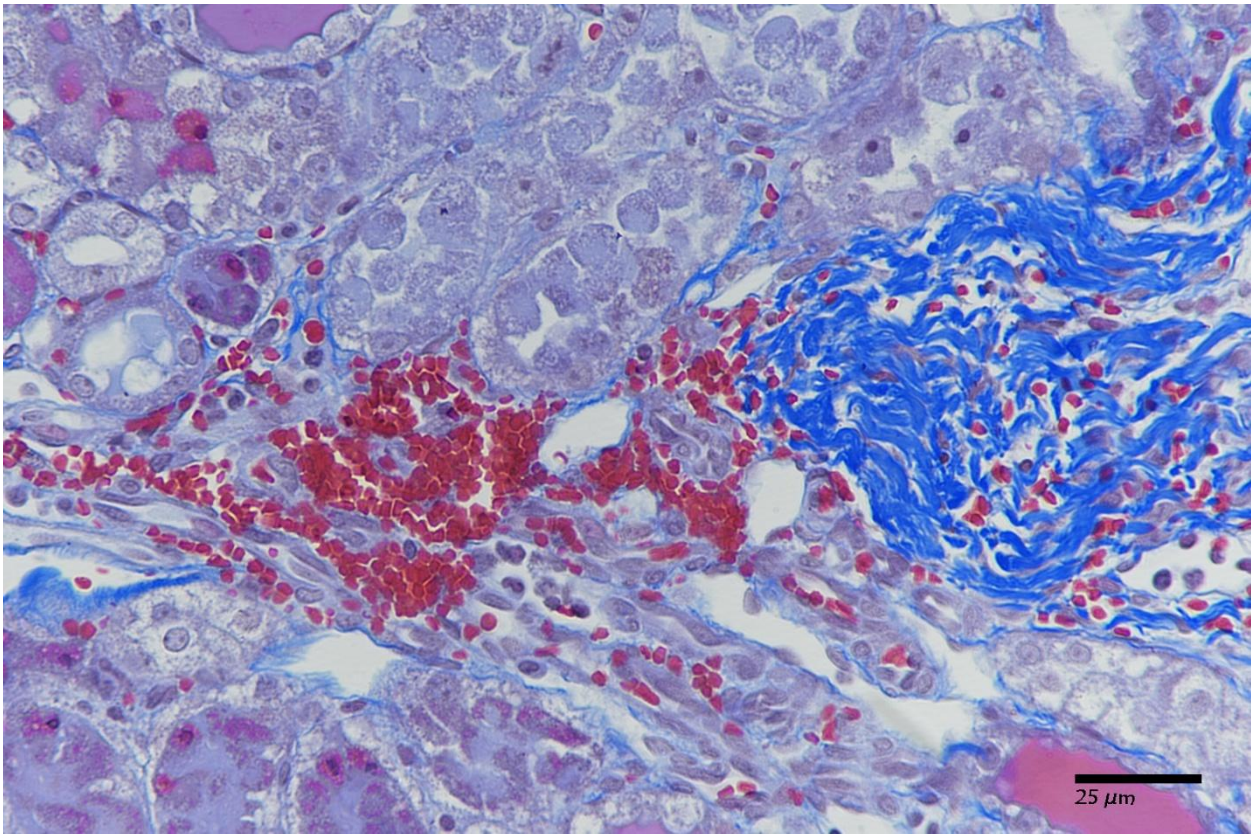
25 μm

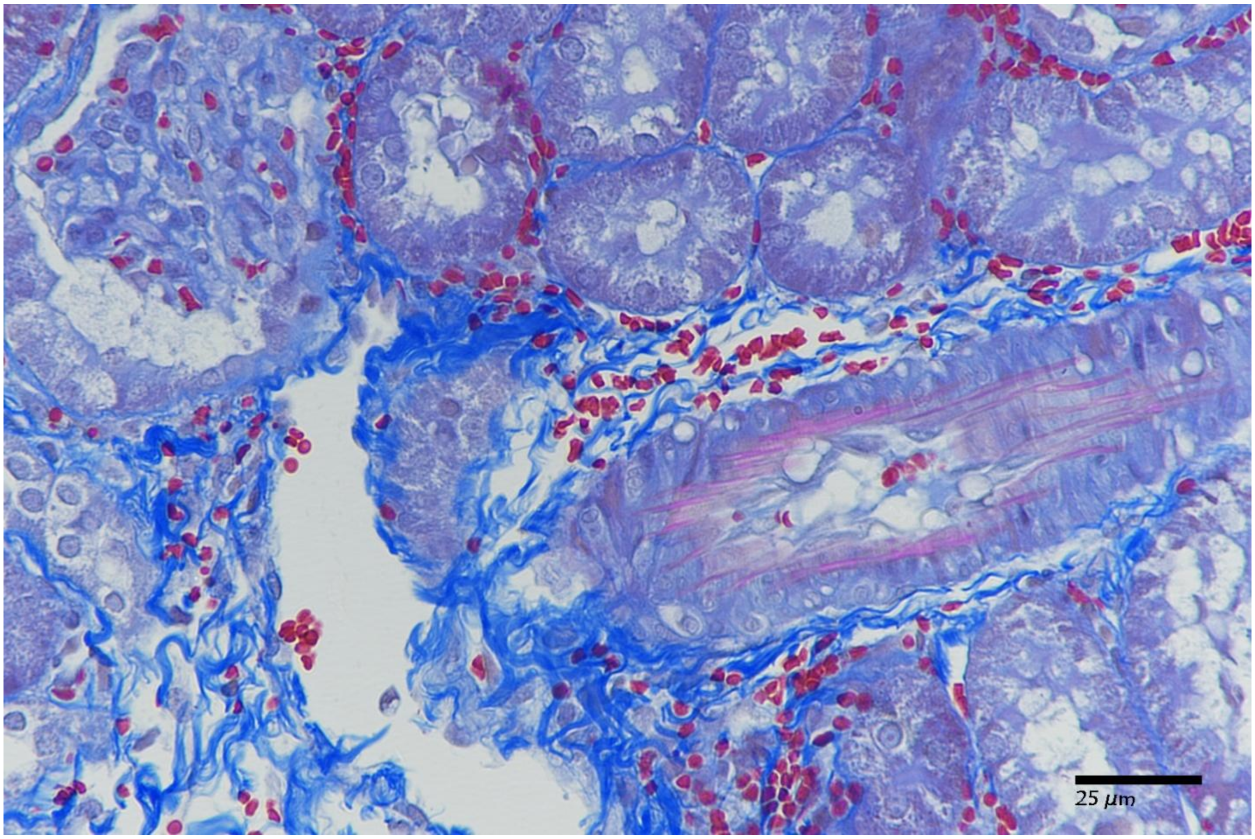
25 μm

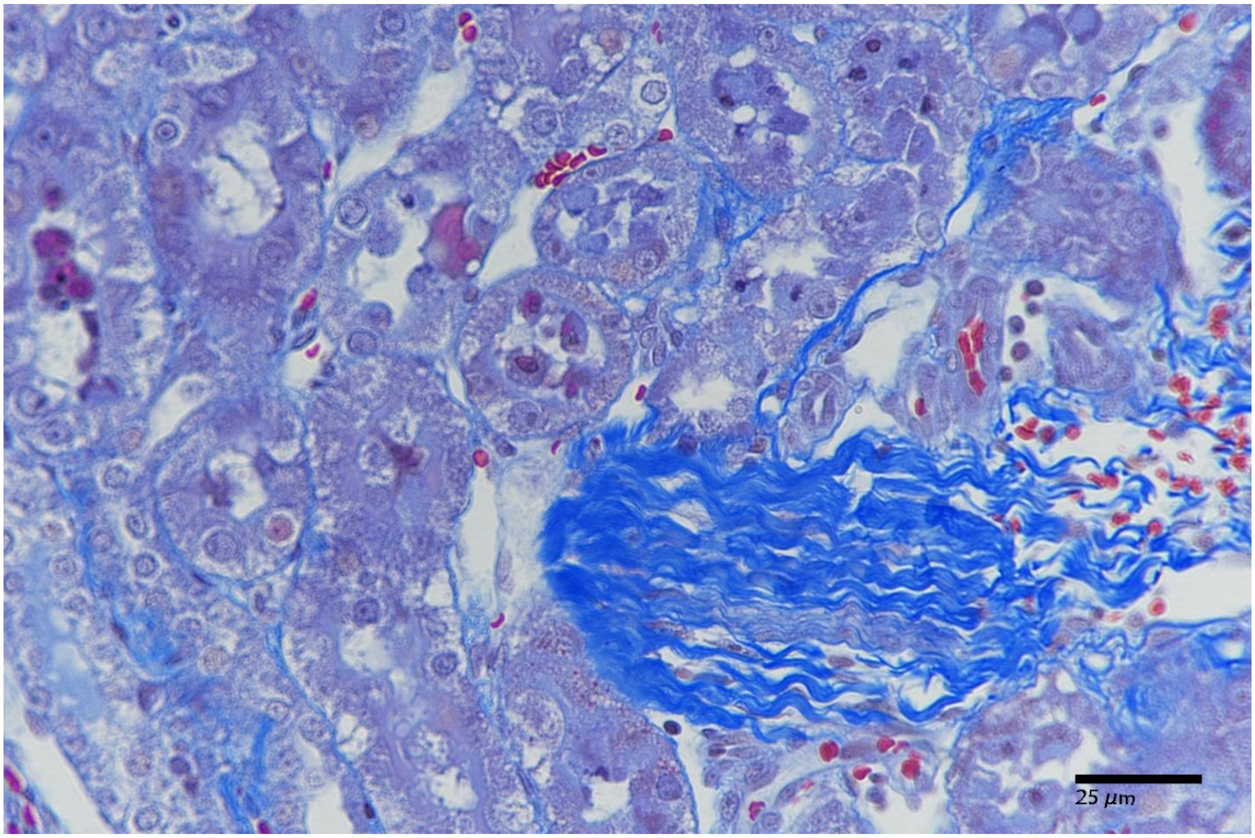
25 μm

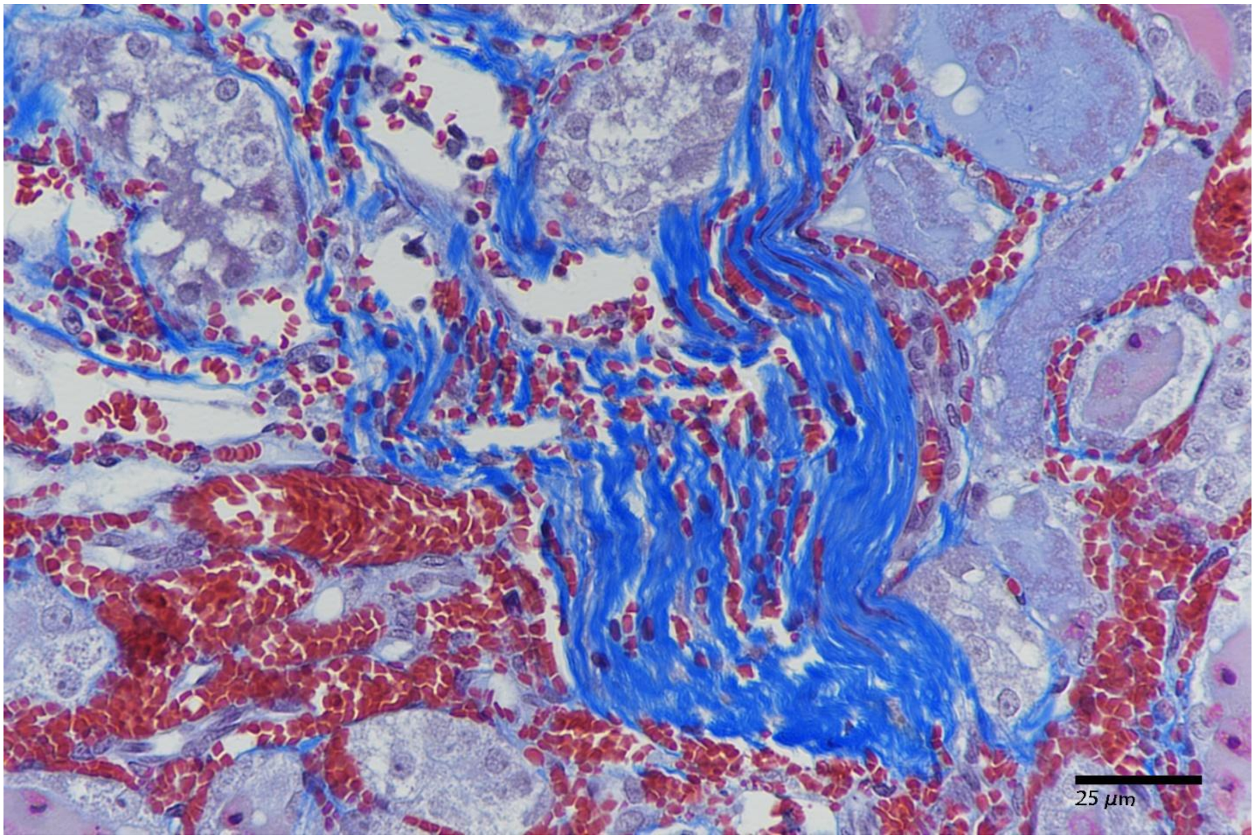
25 μm

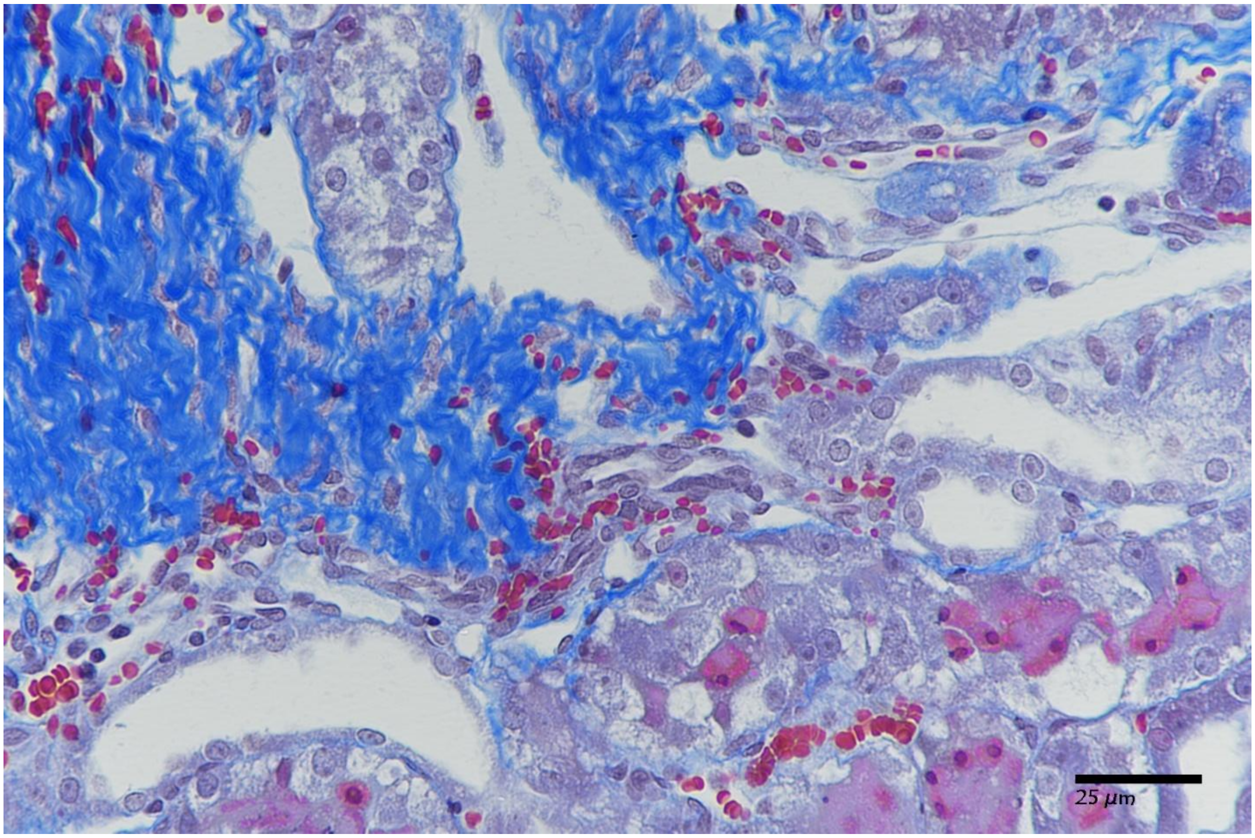
25 μm

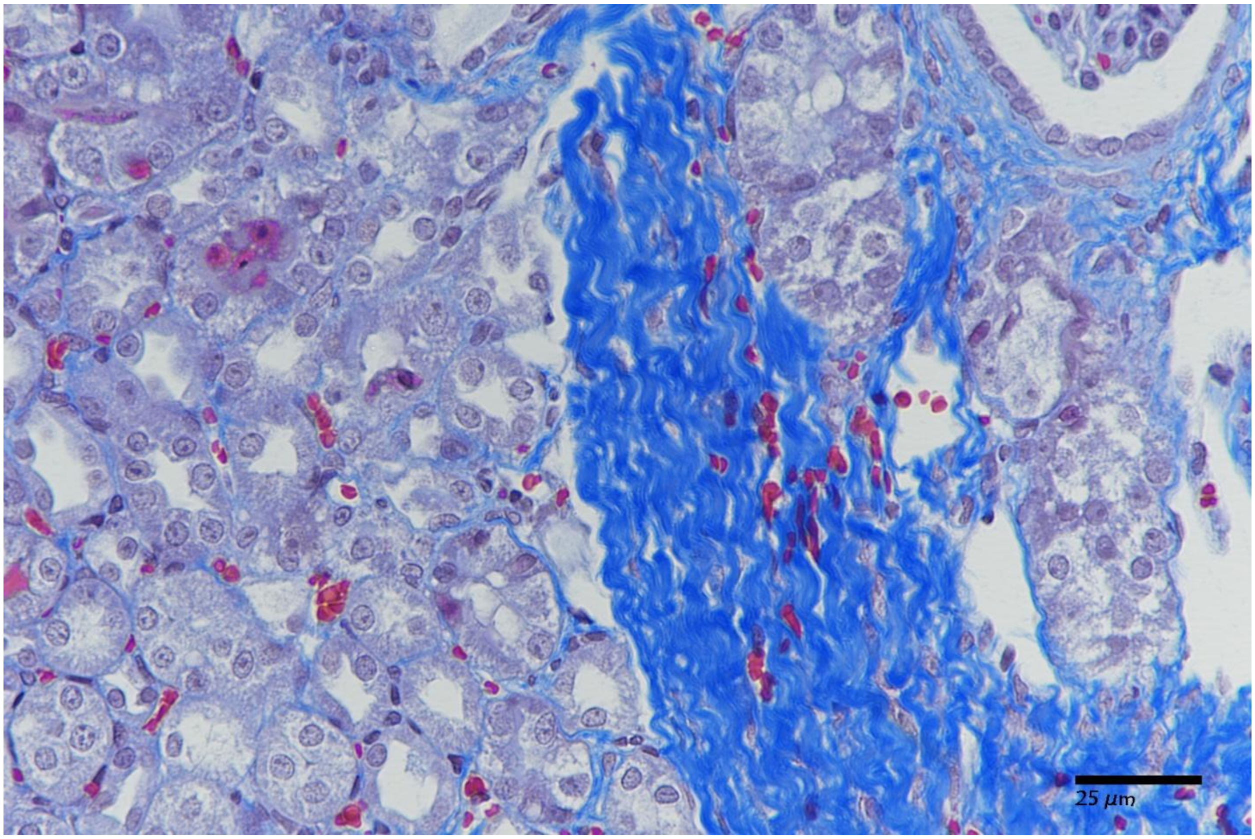
25 μm

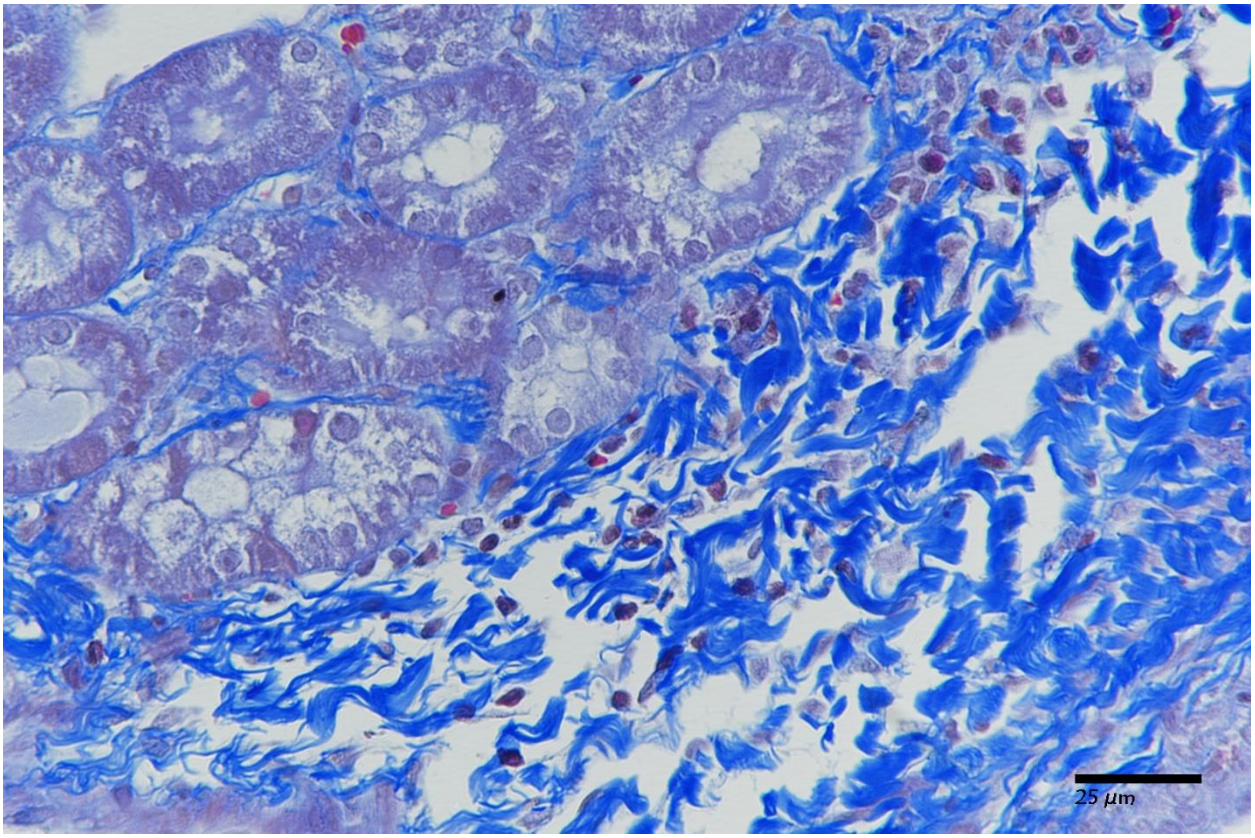
25 μm

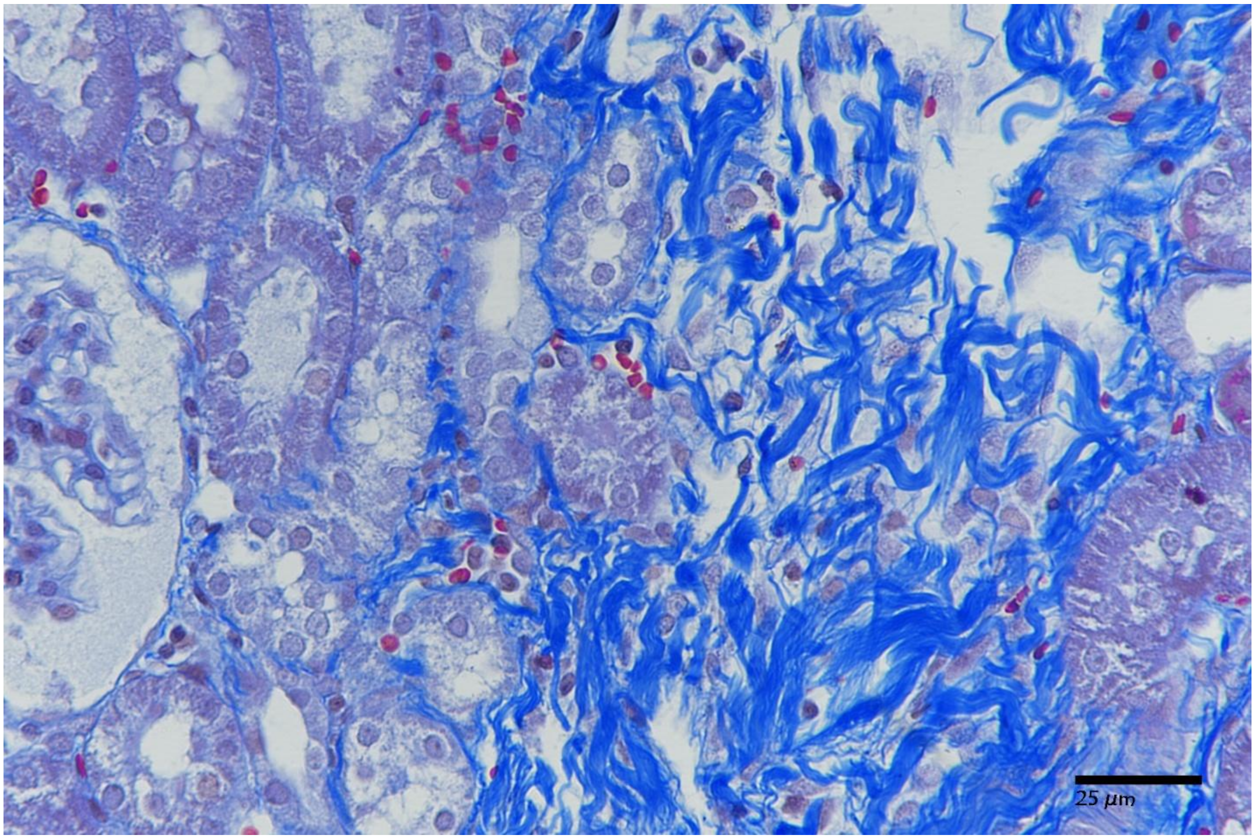
25 μm

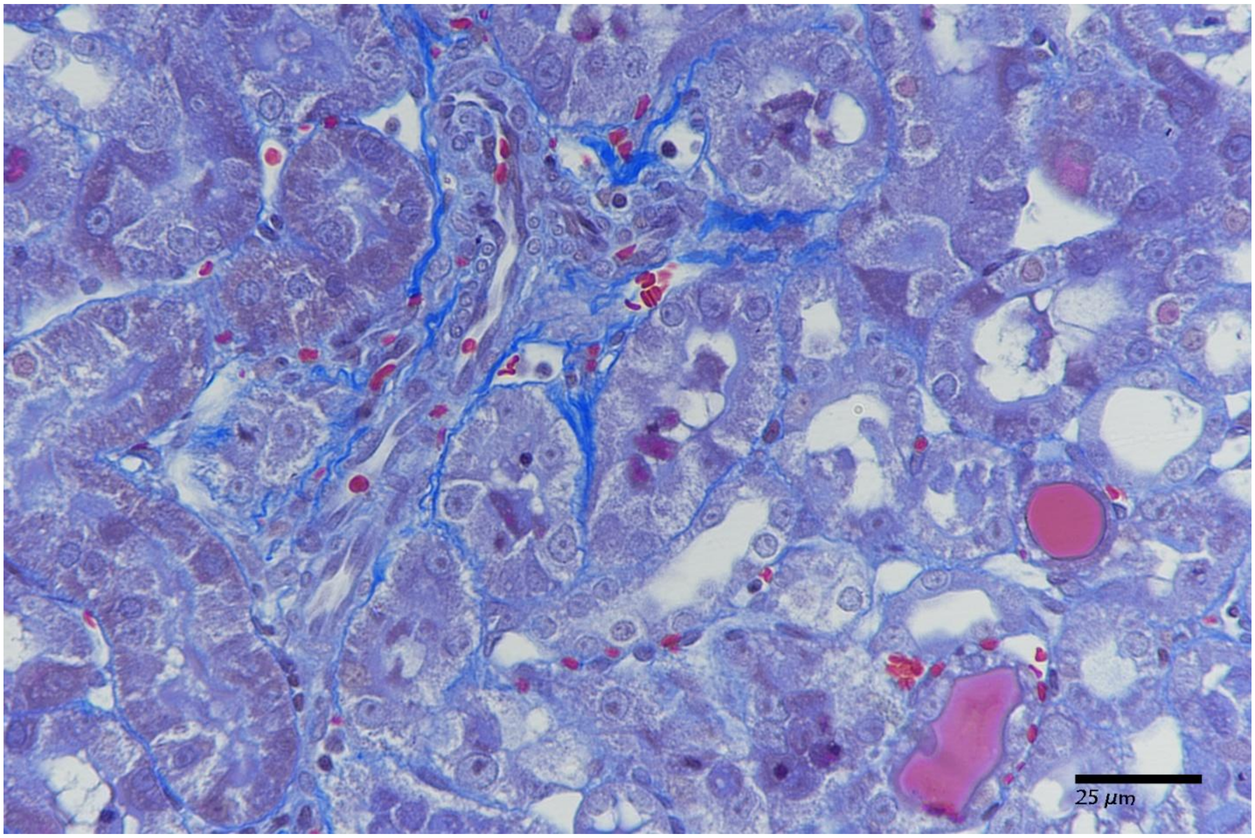
25 μm

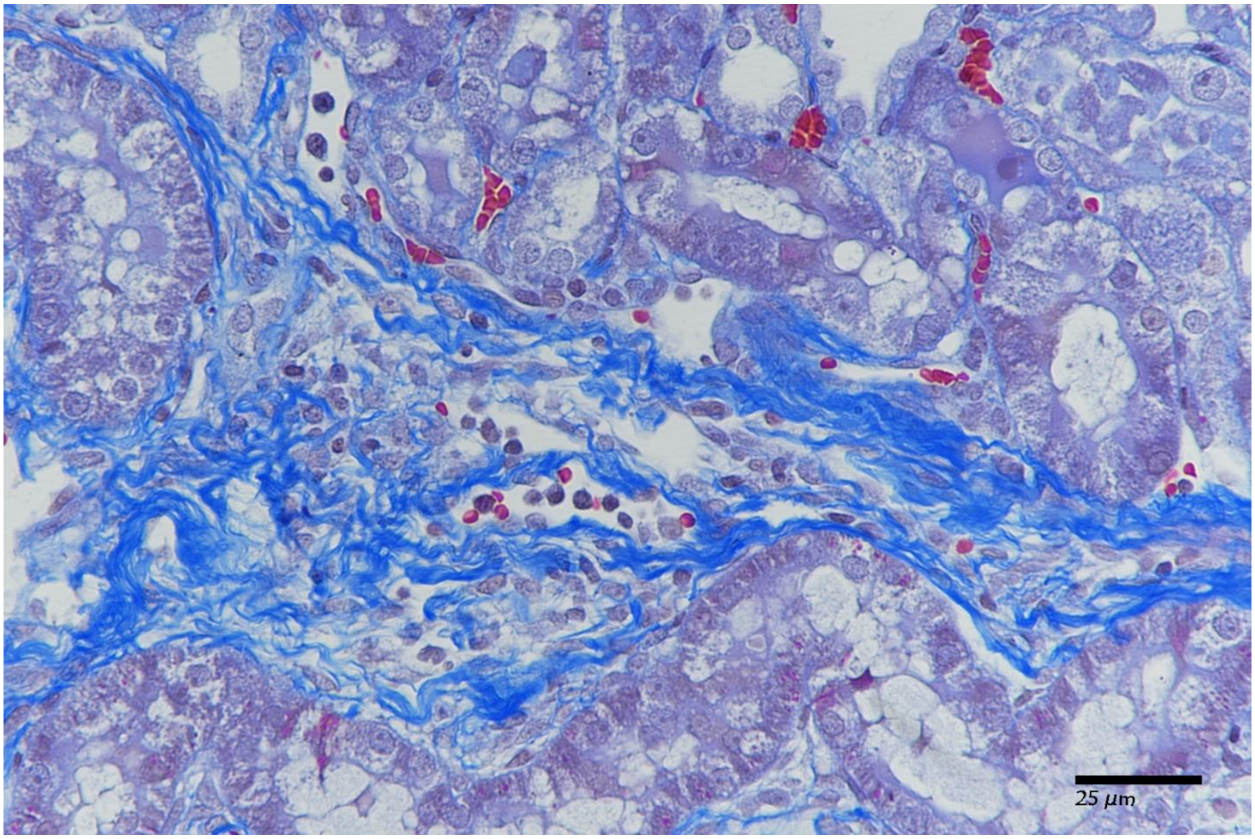
25 μm

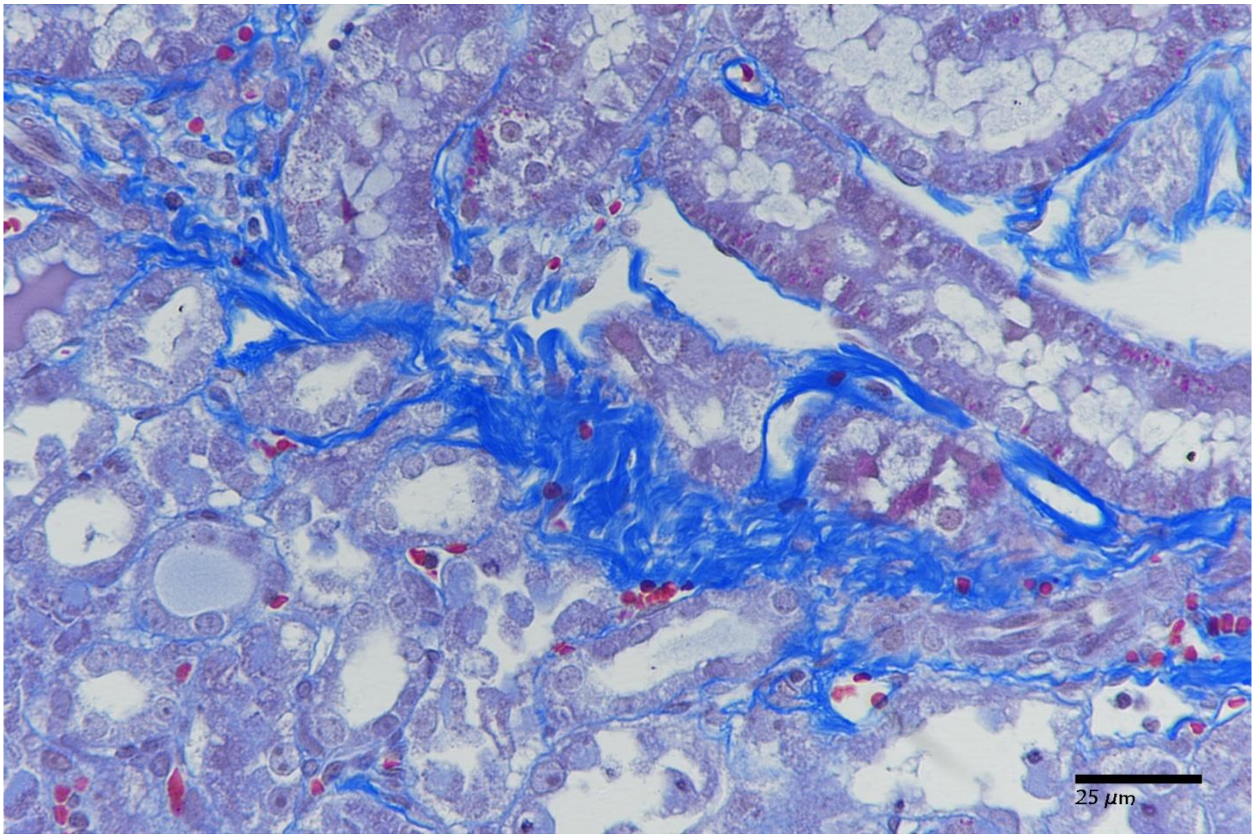
25 μm

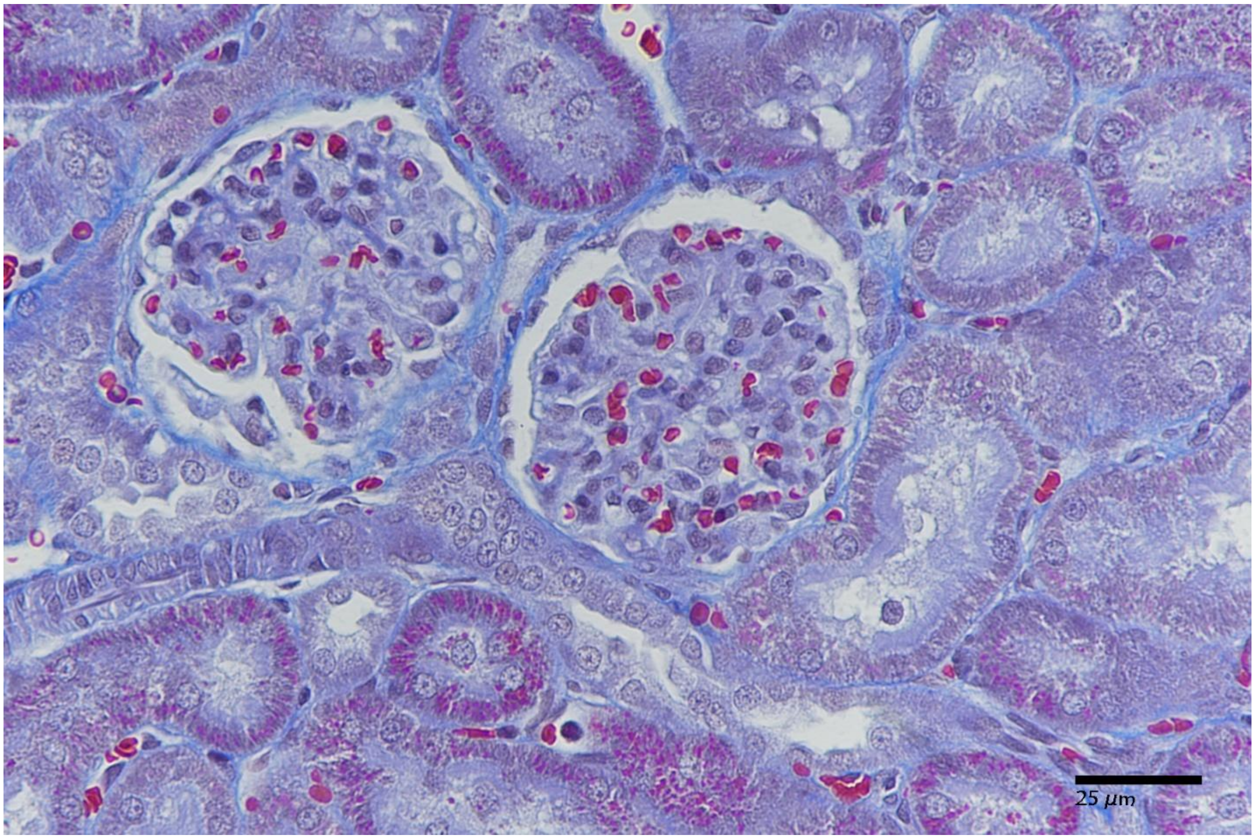
25 μm

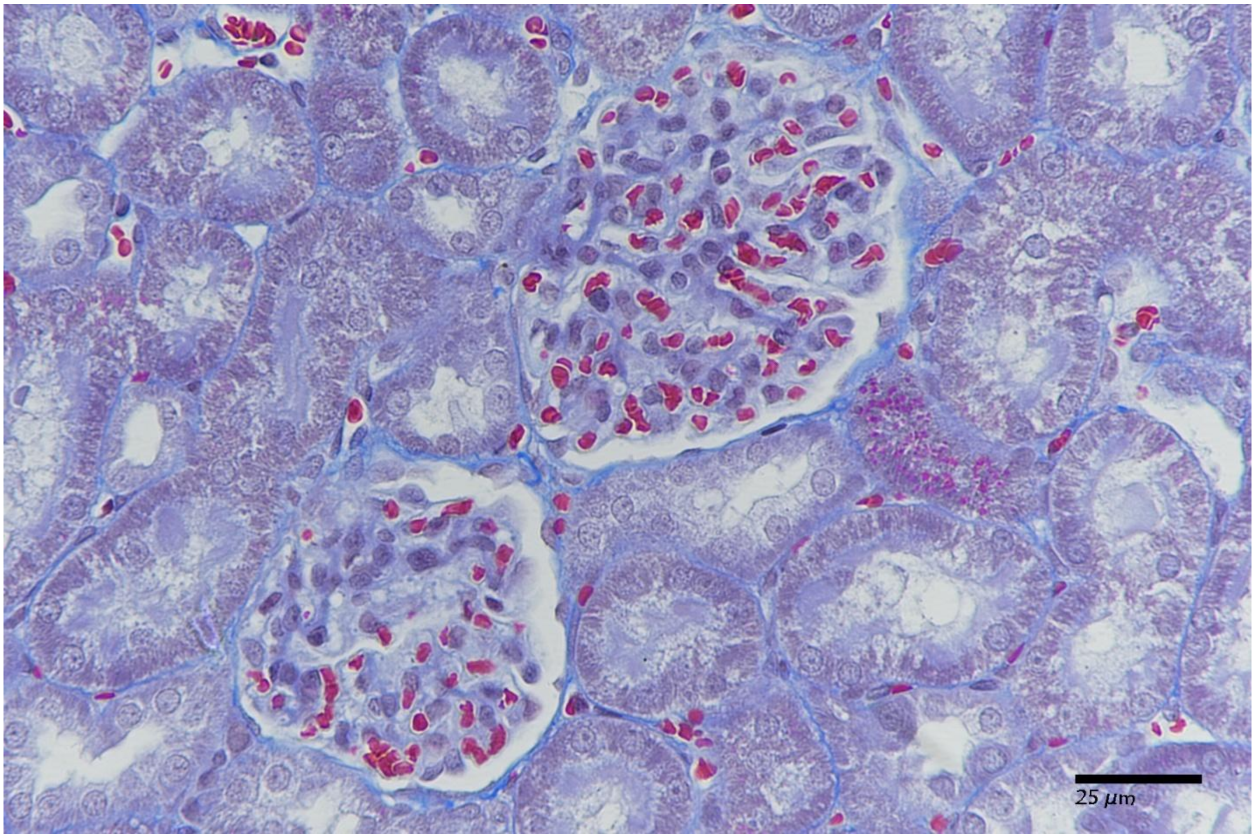
25 μm

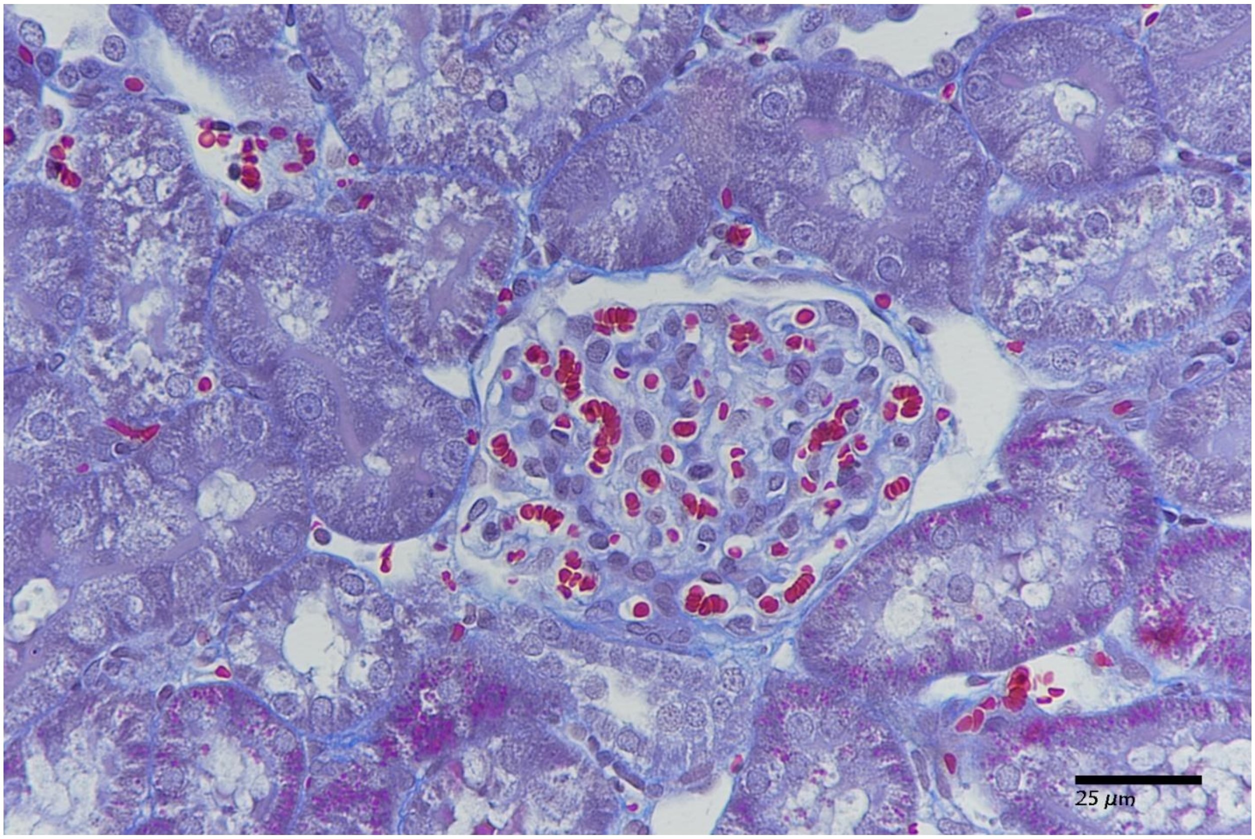
25 μm

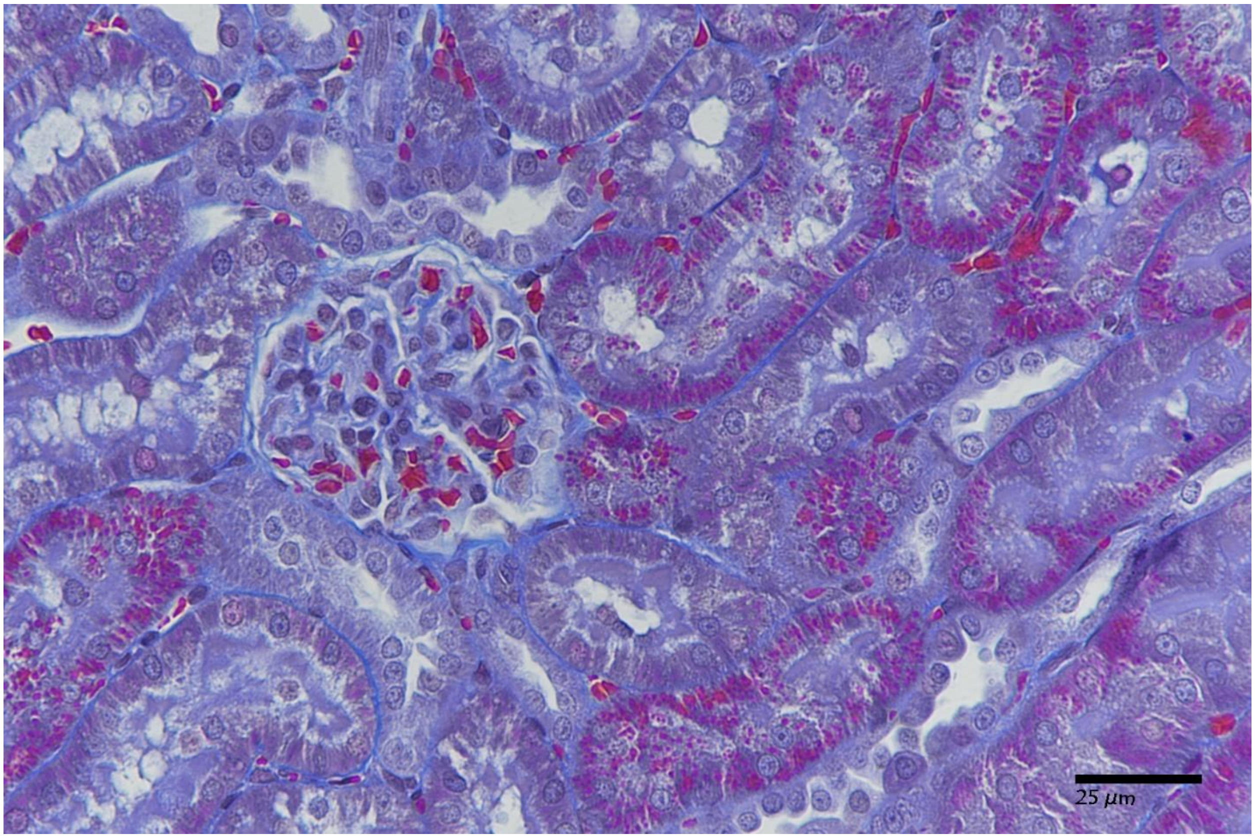
25 μm
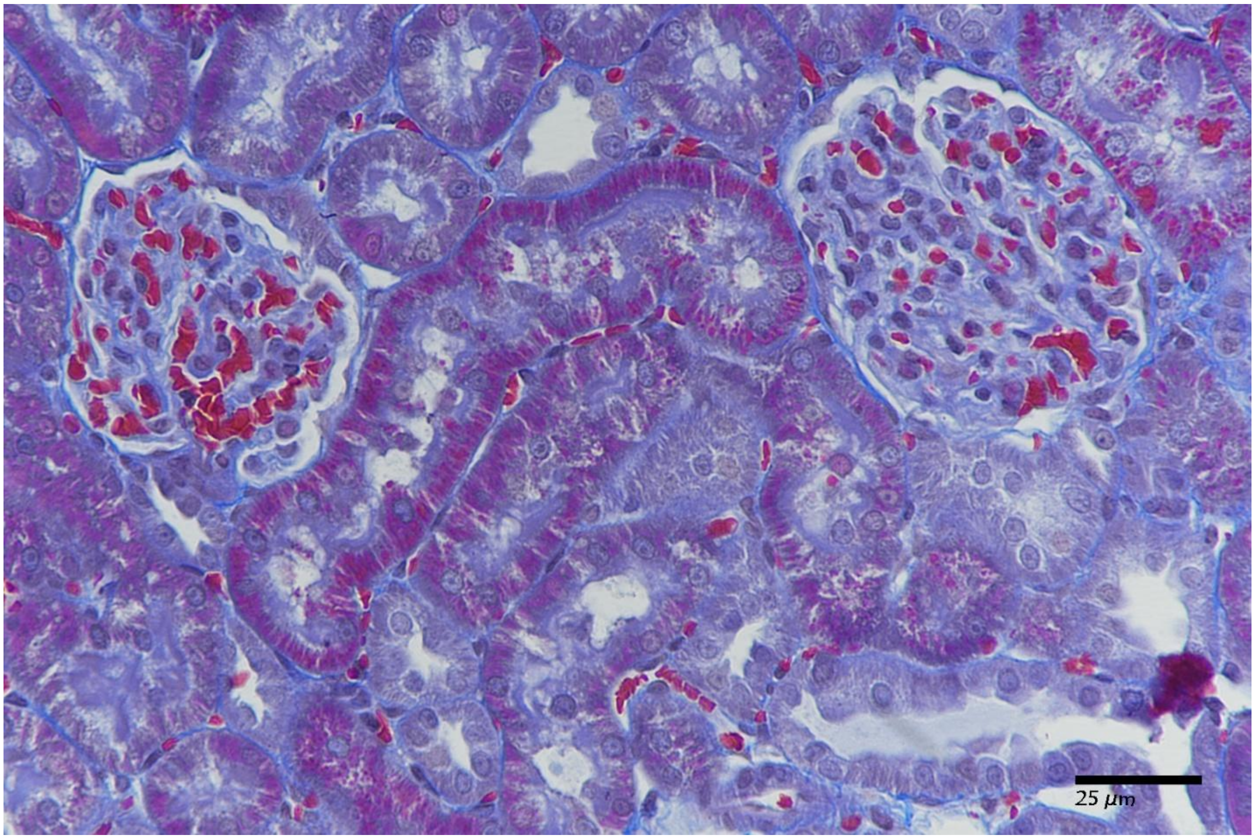
25 μm

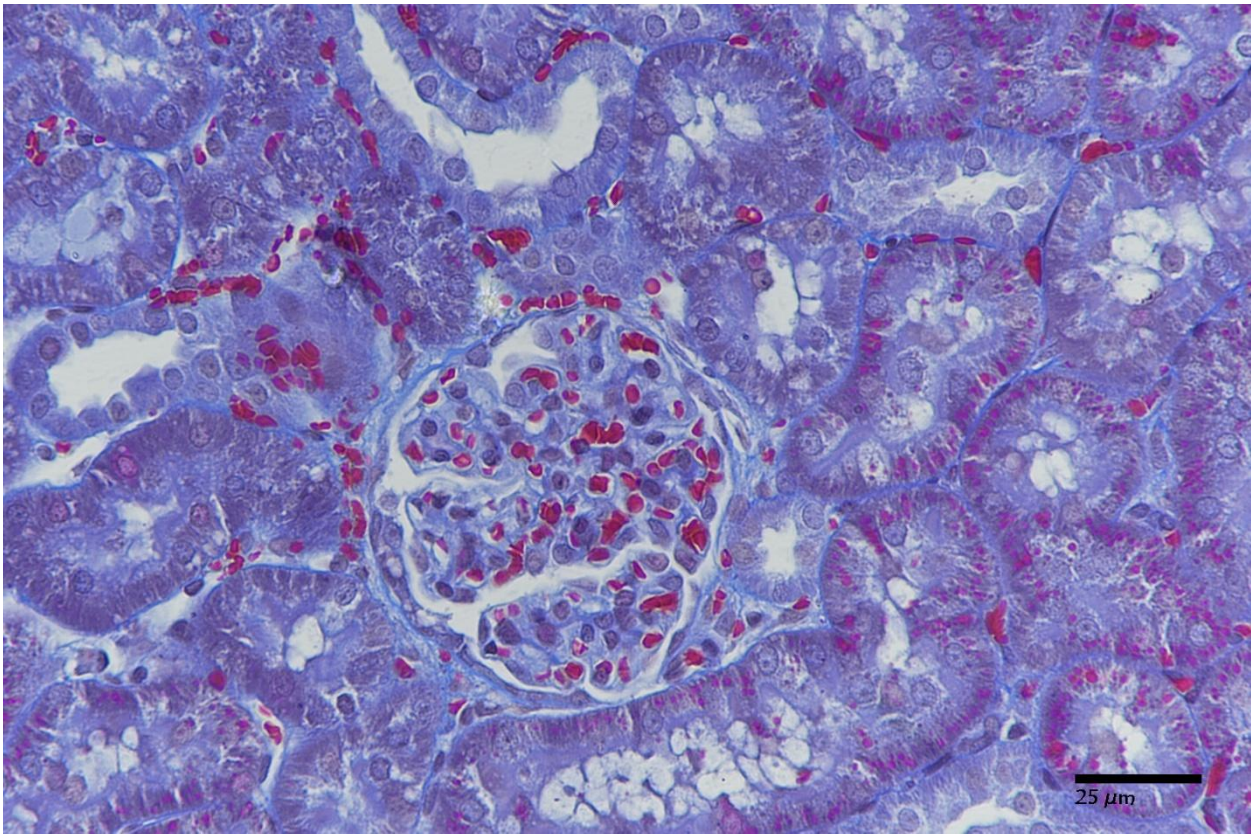
25 μm

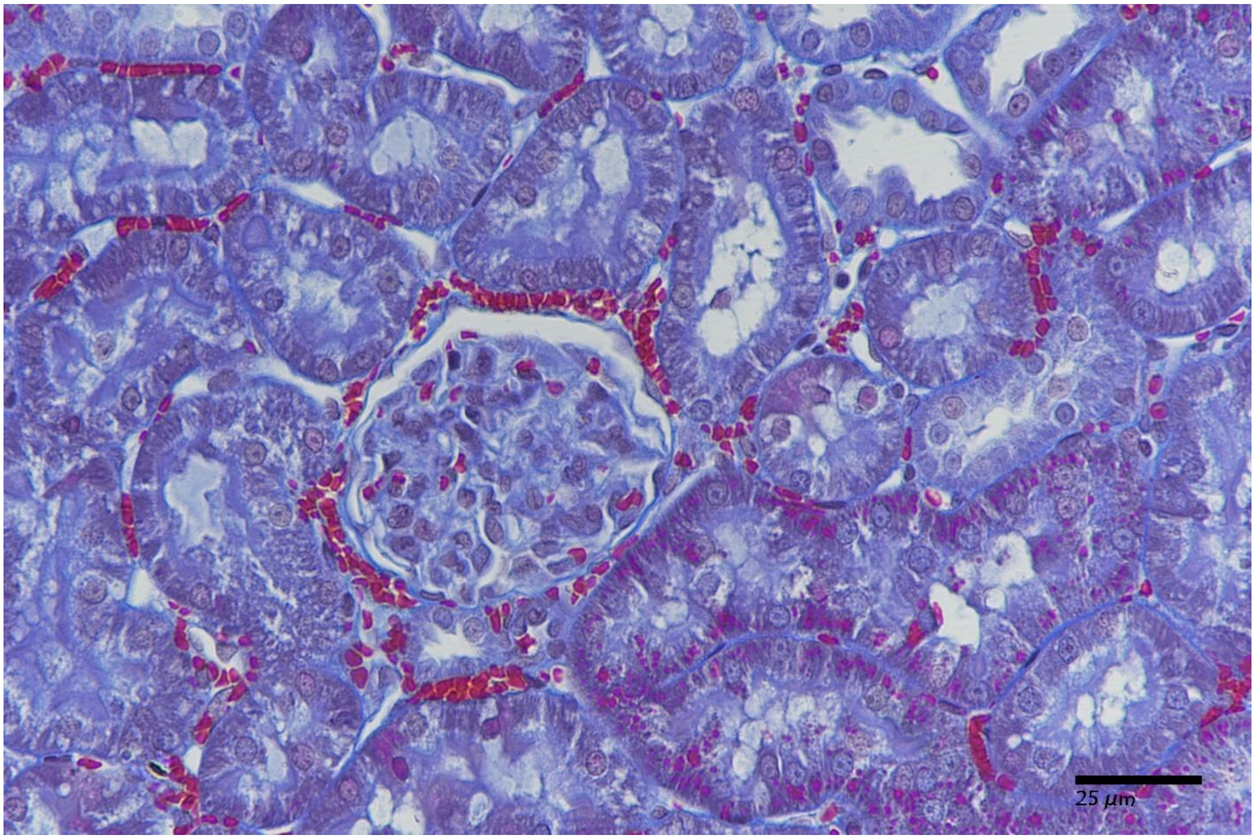
25 μm

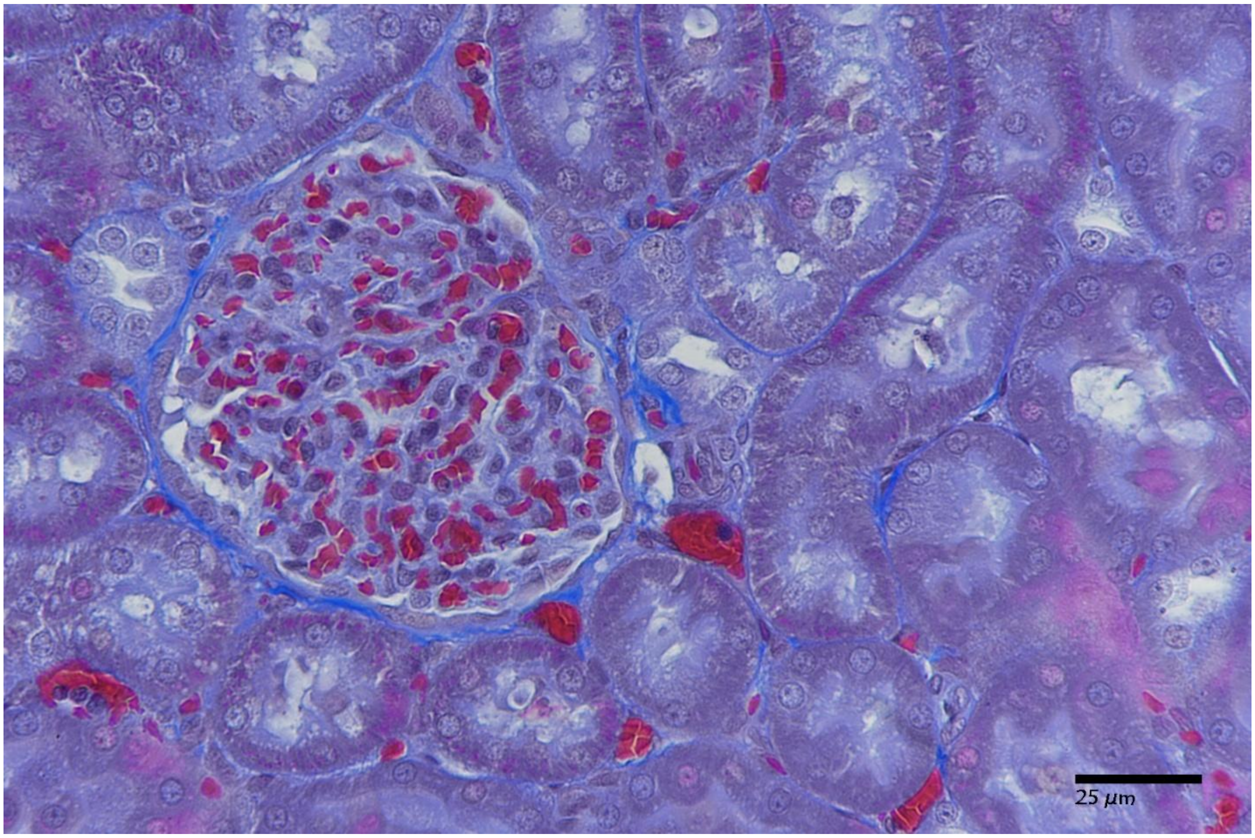
25 μm

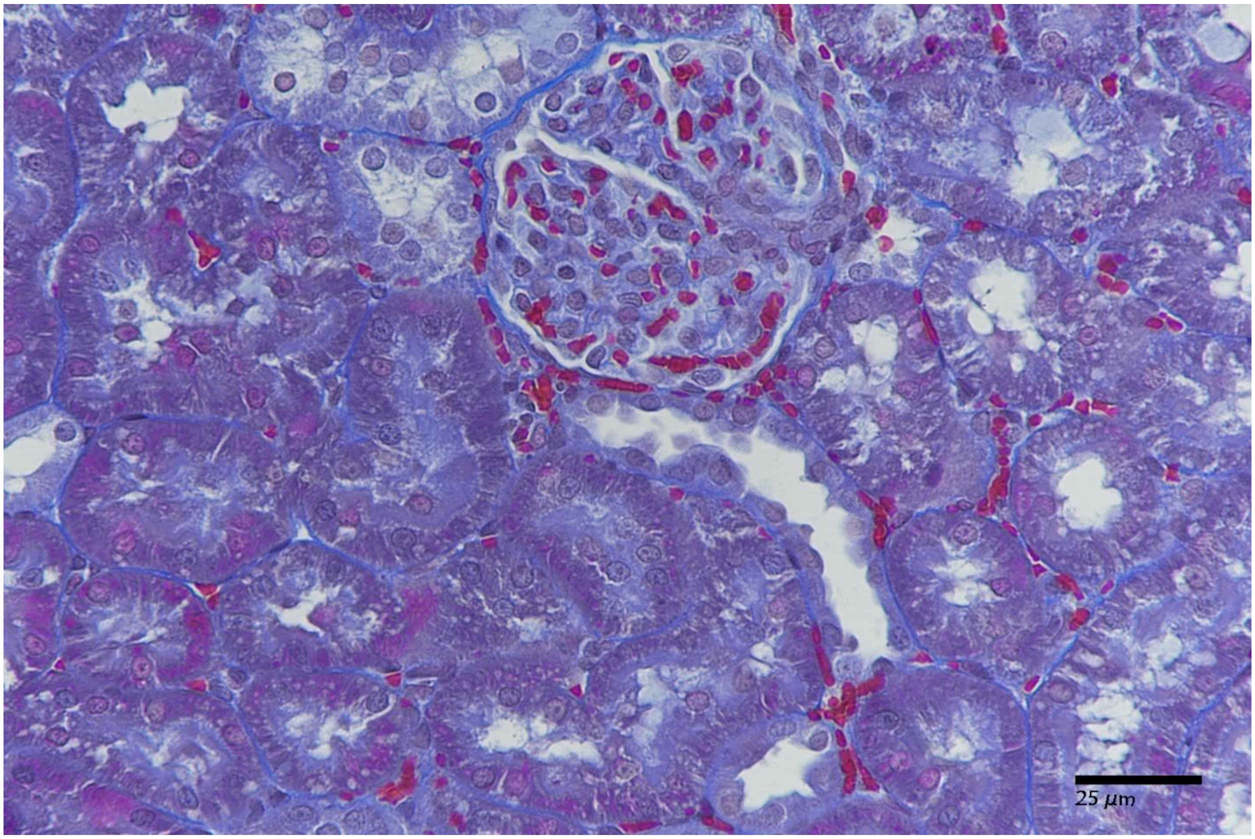
25 μm

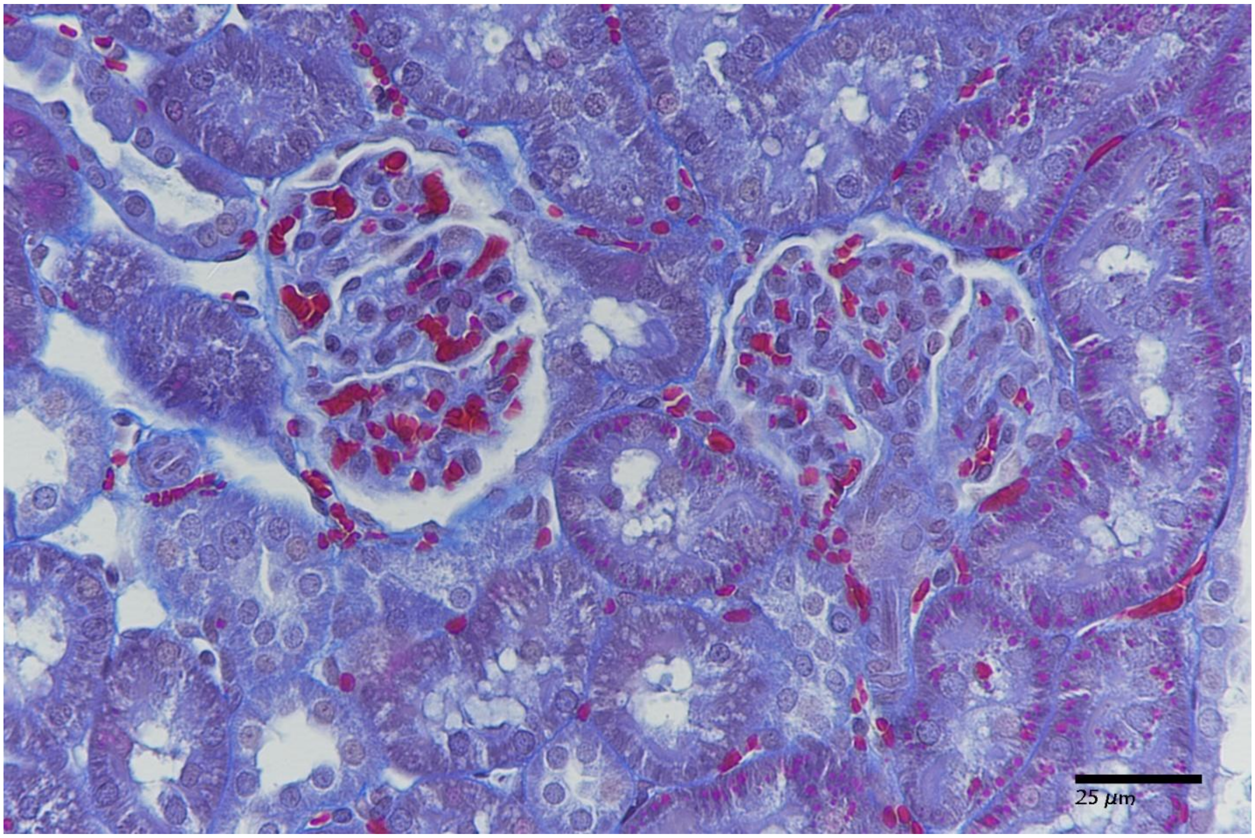
25 μm

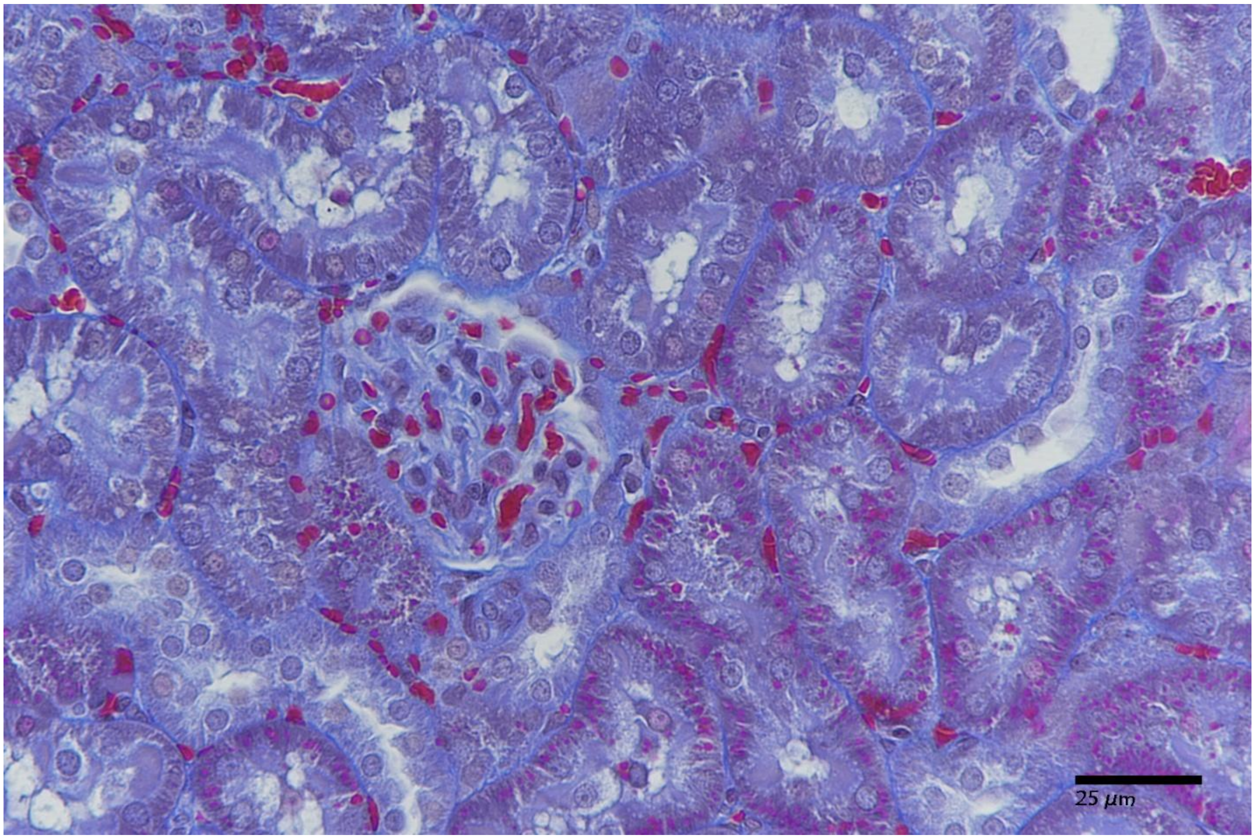
25 μm

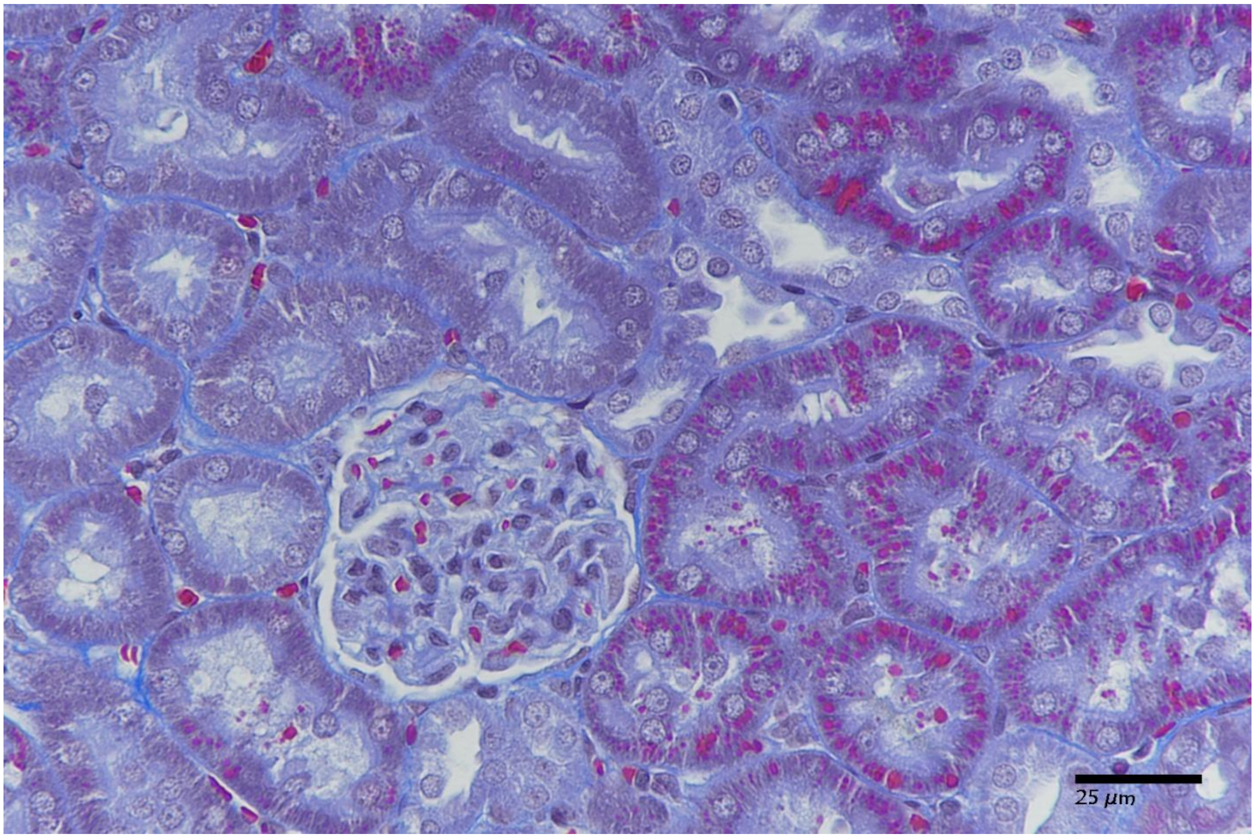
25 μm

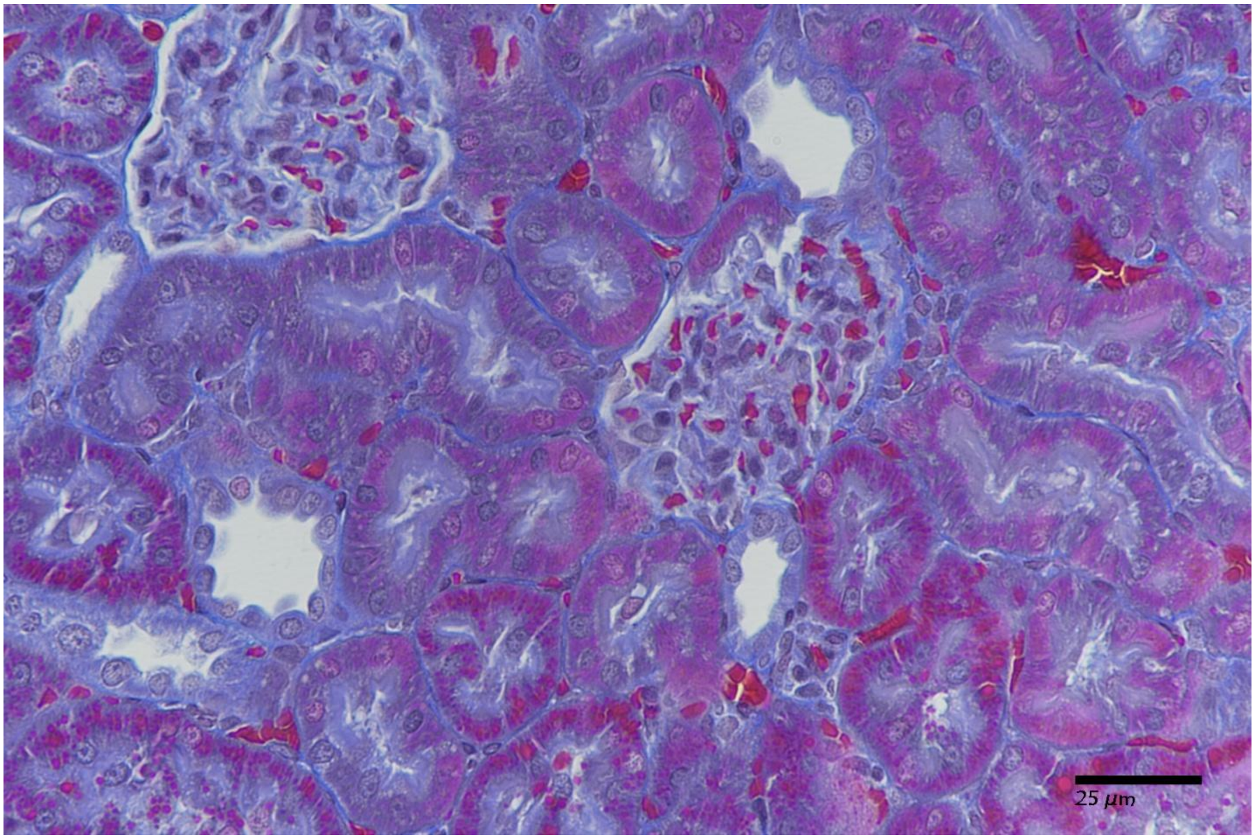
25 μm

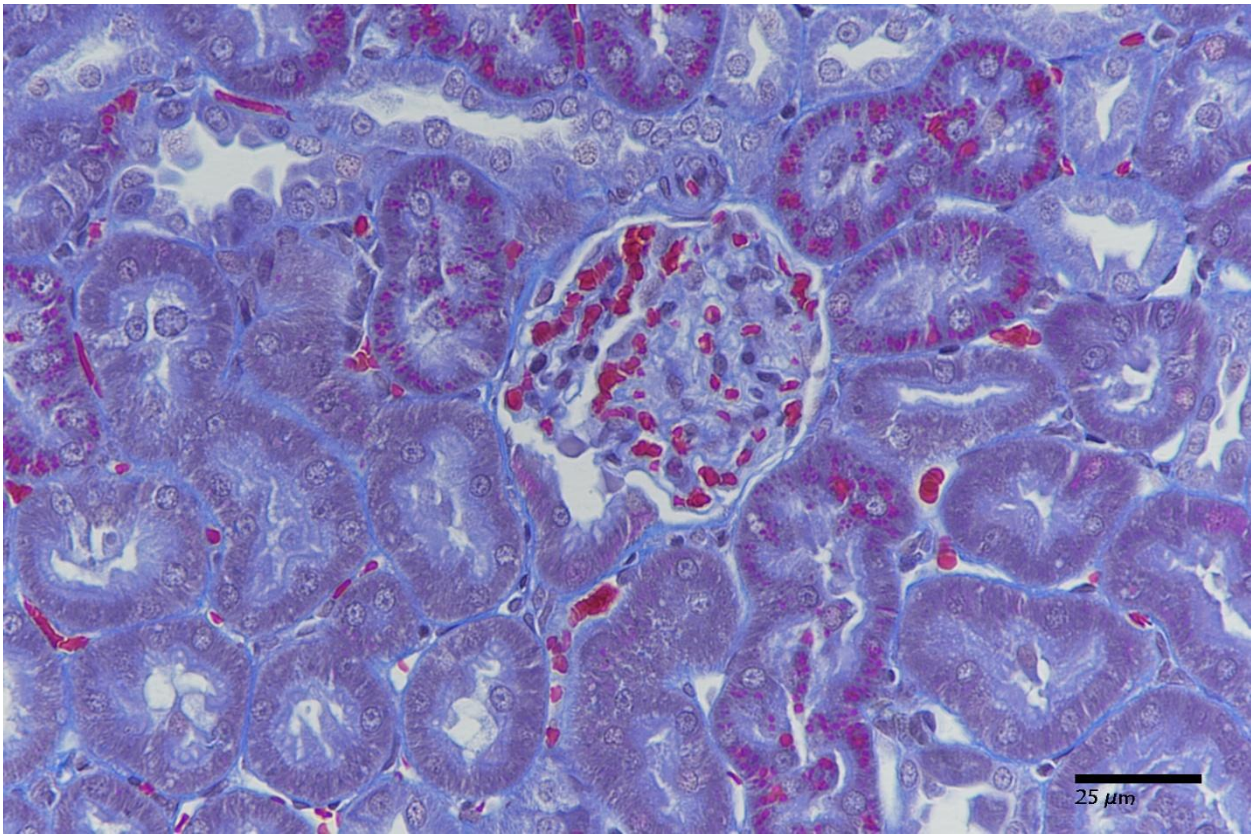
25 μm

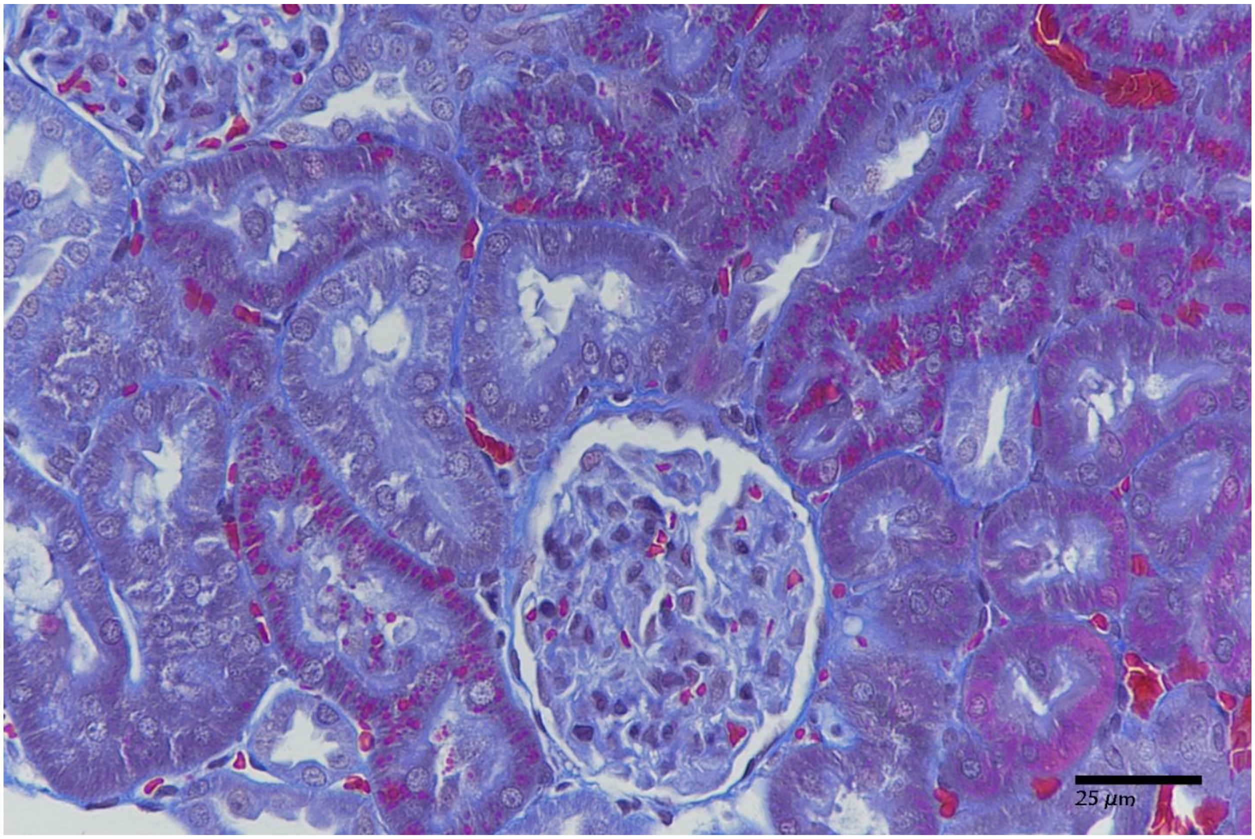
25 μm

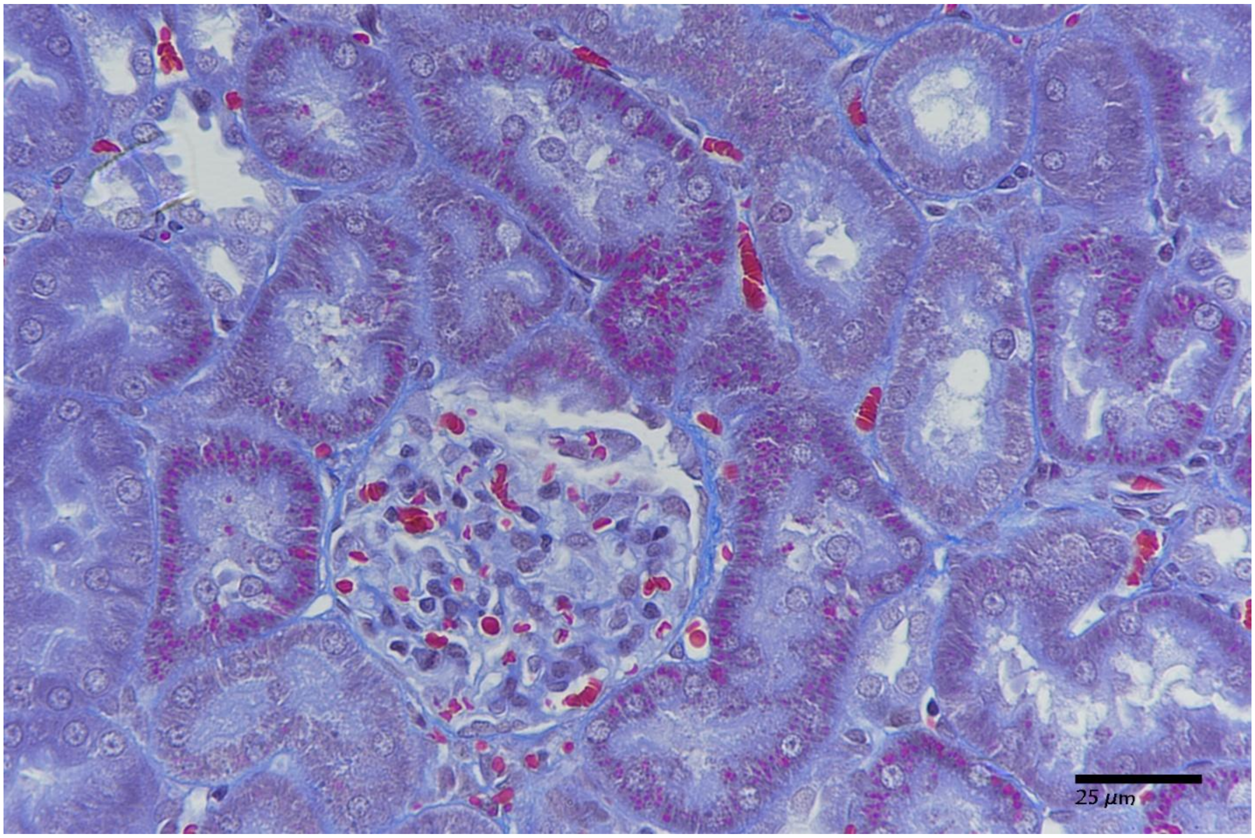
25 μm

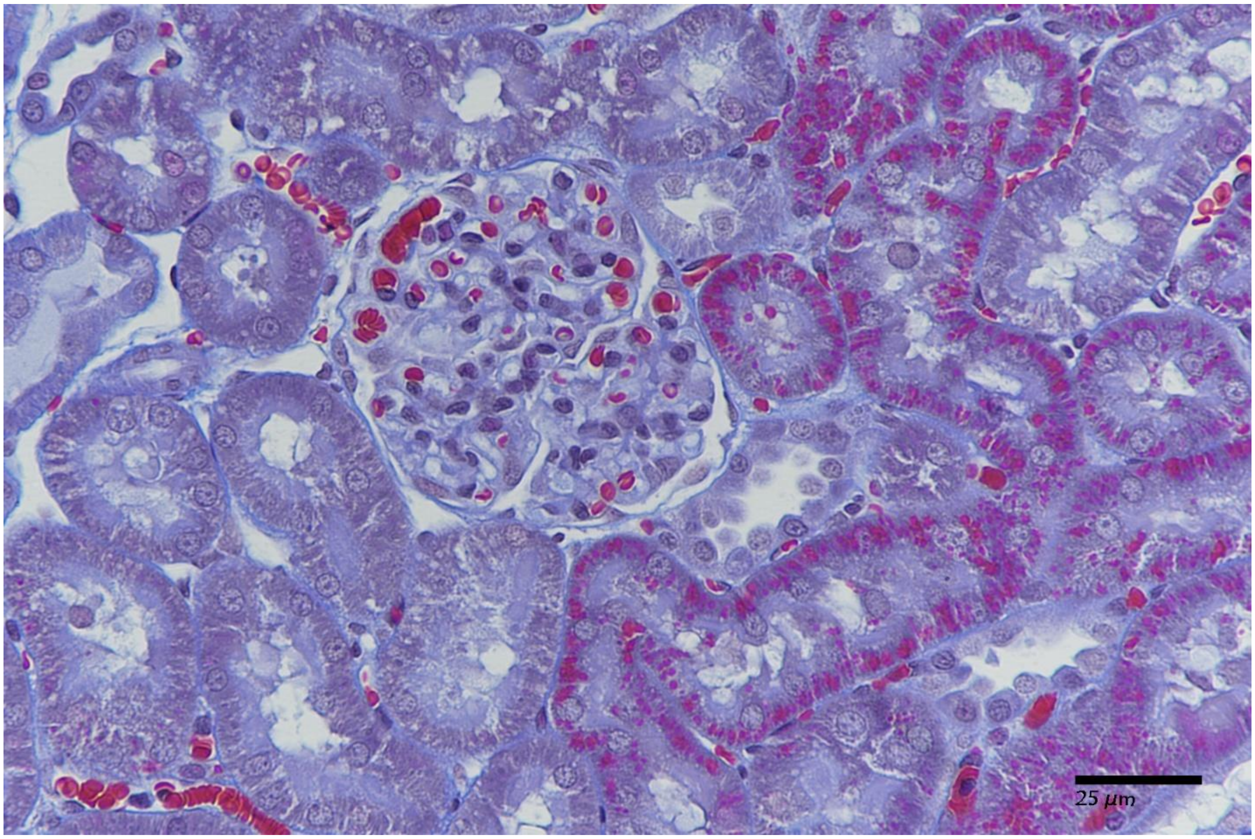
25 μm

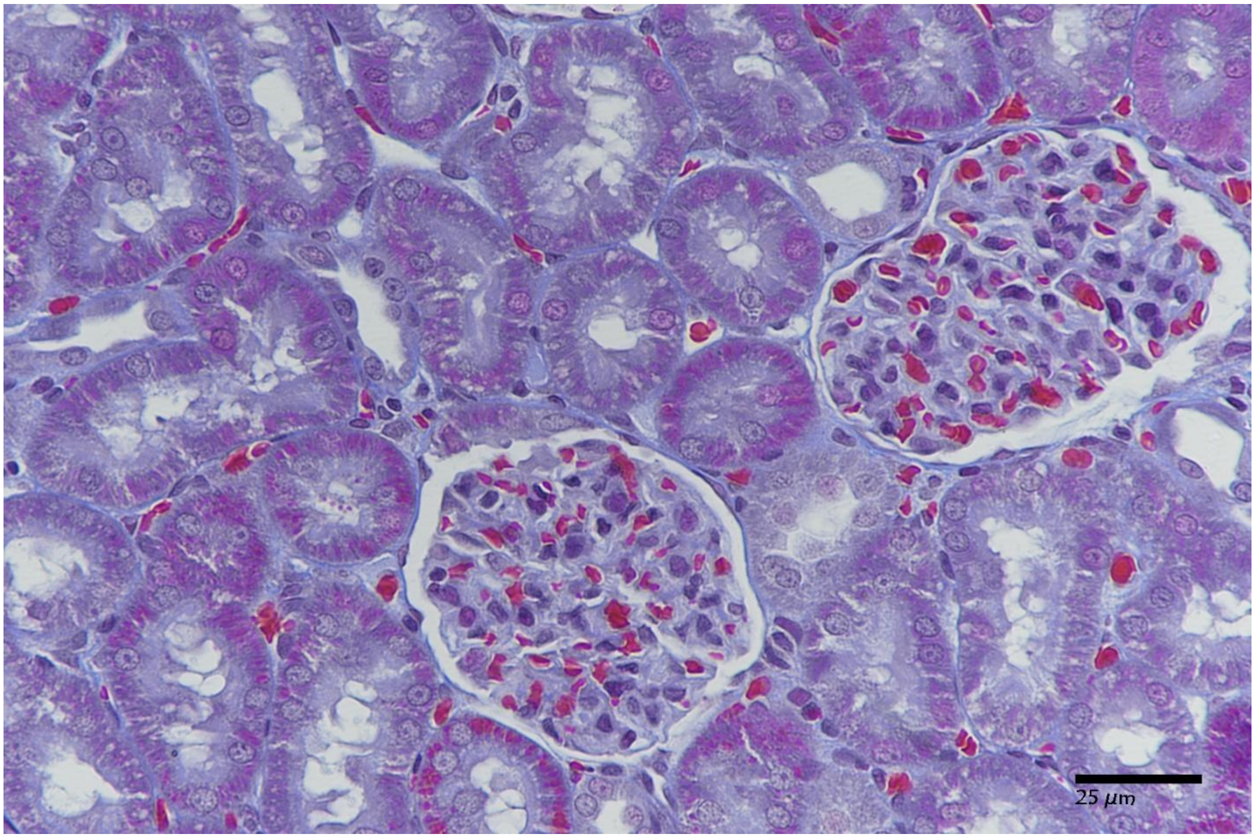
25 μm

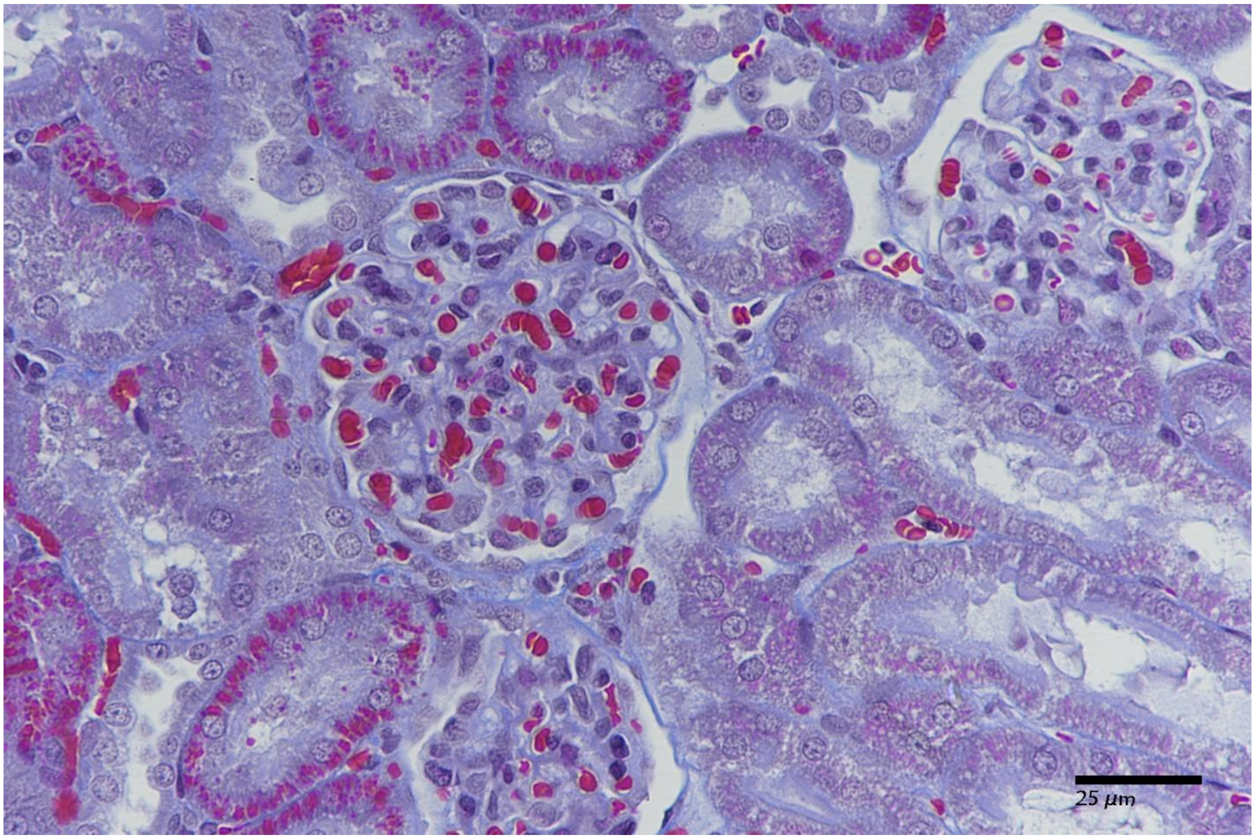
25 μm

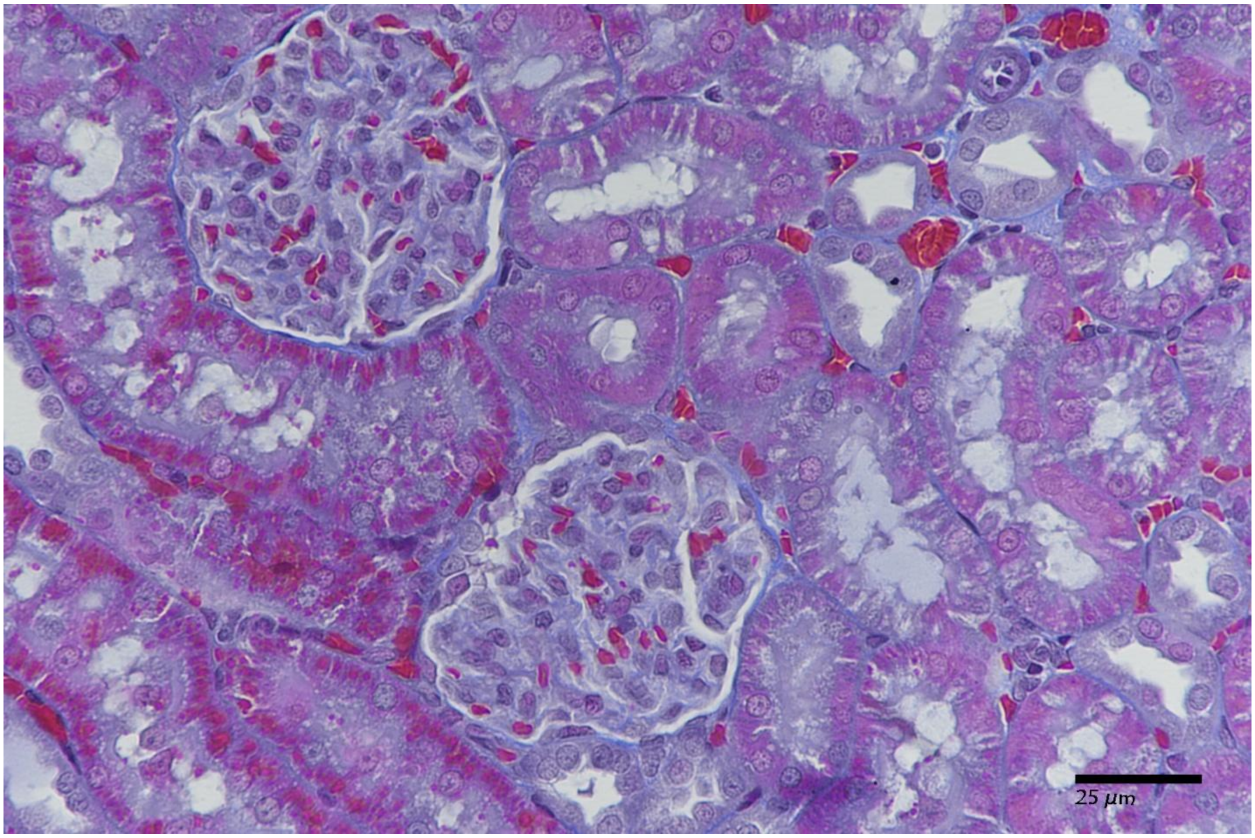
25 μm

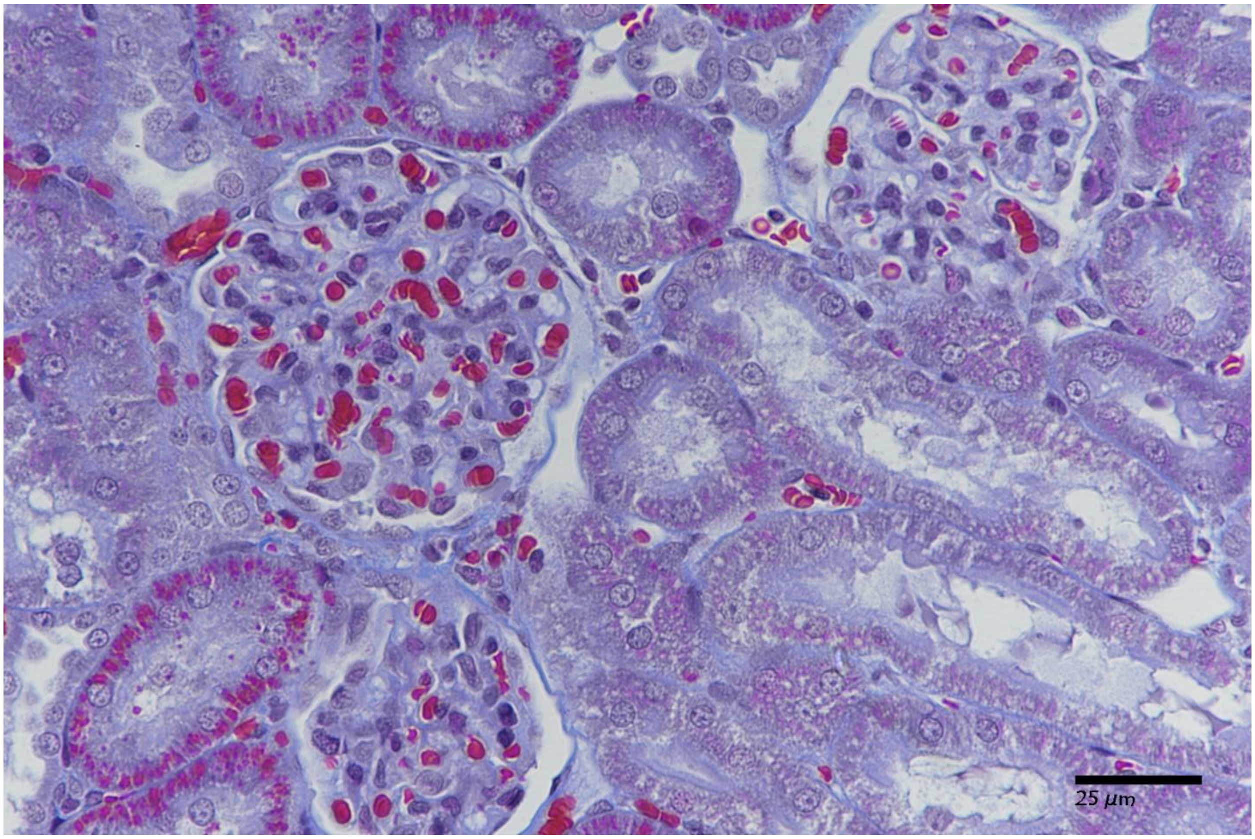
25 μm

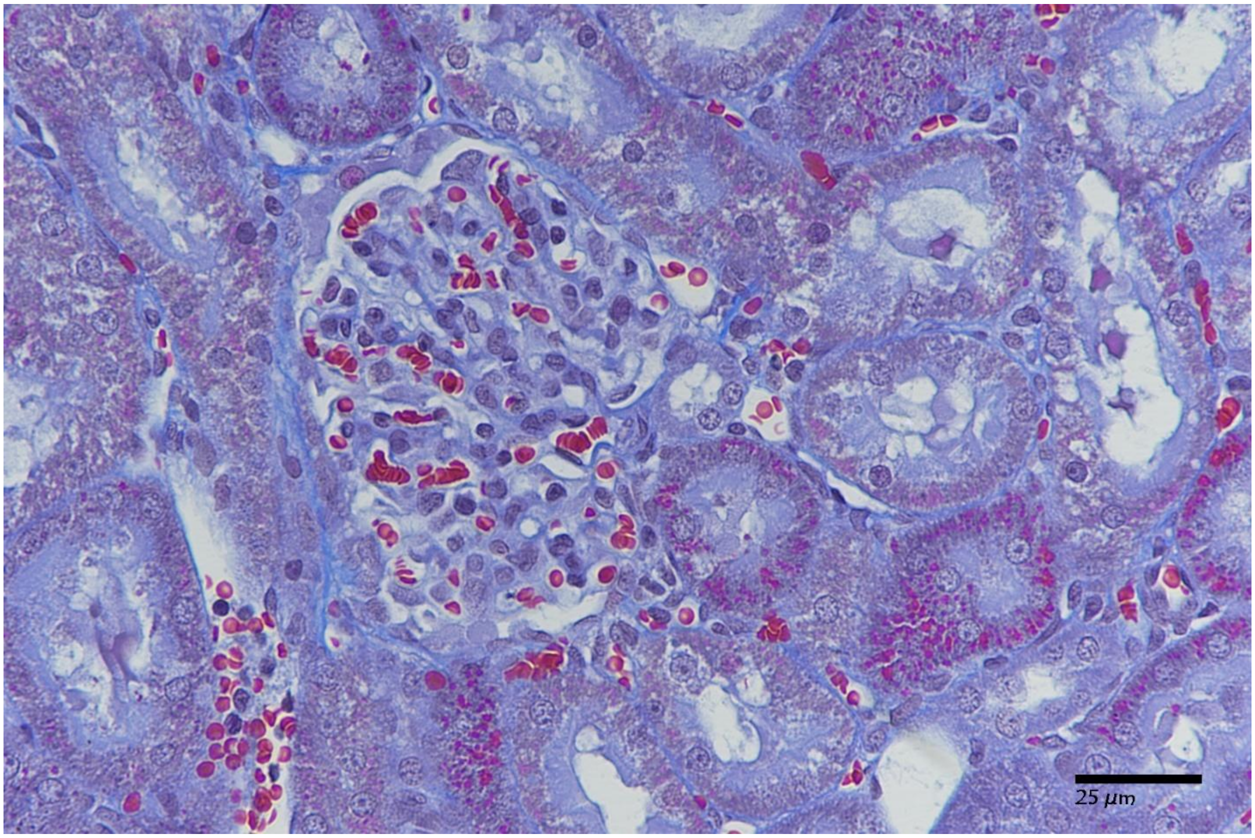
25 μm

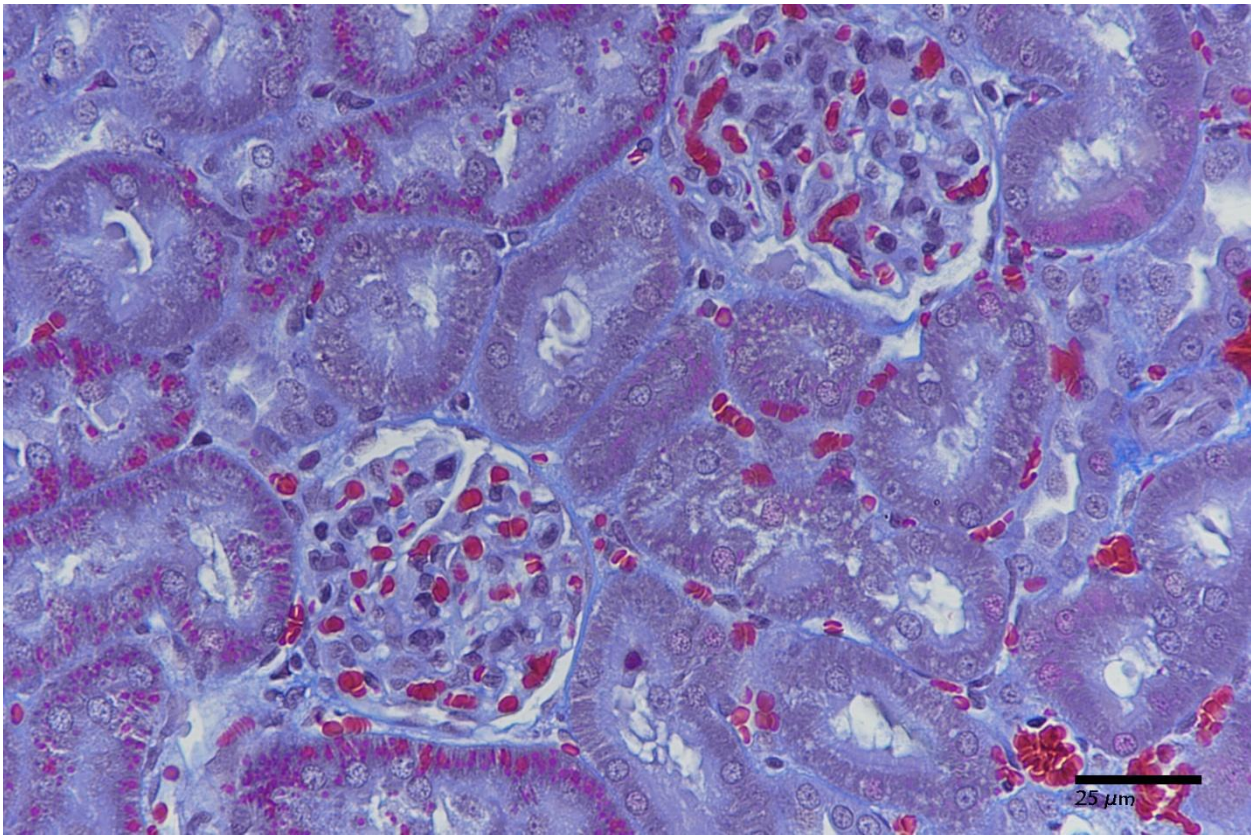
25 μm

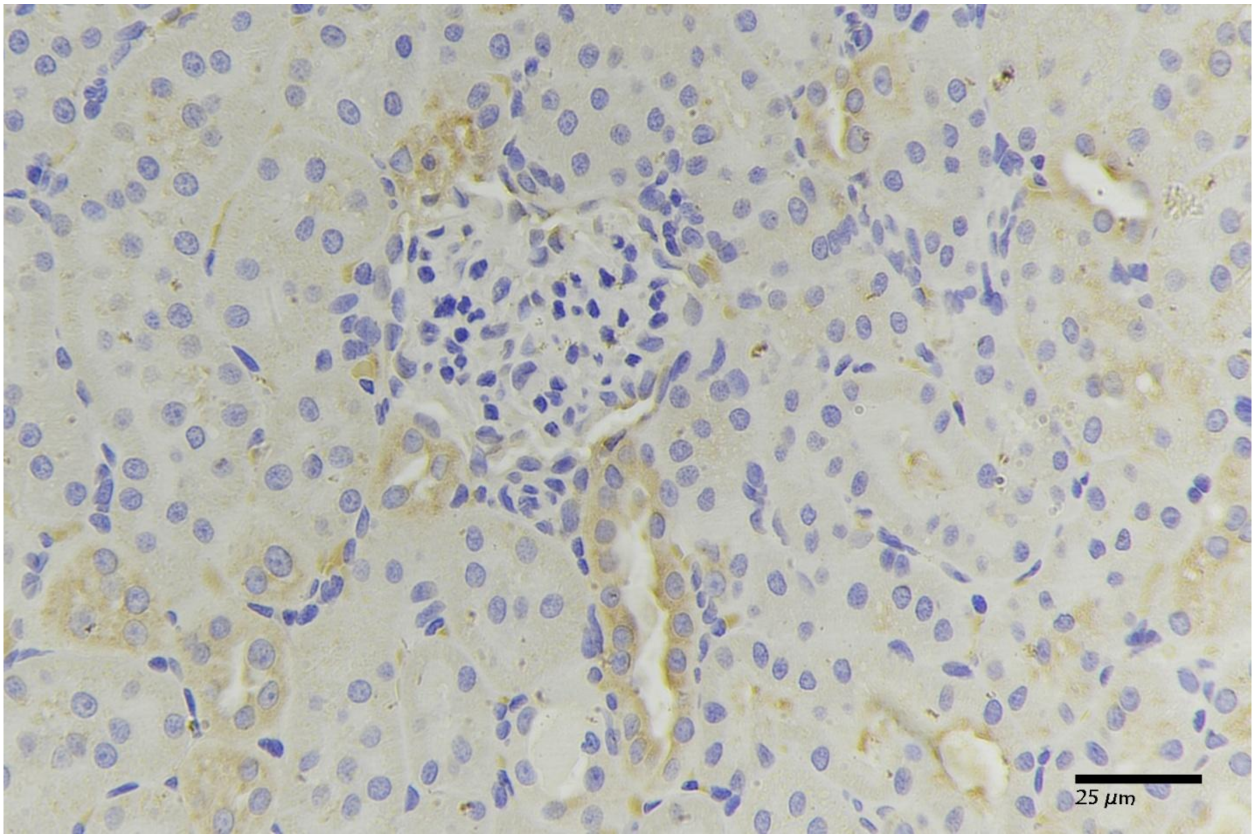
25 μm

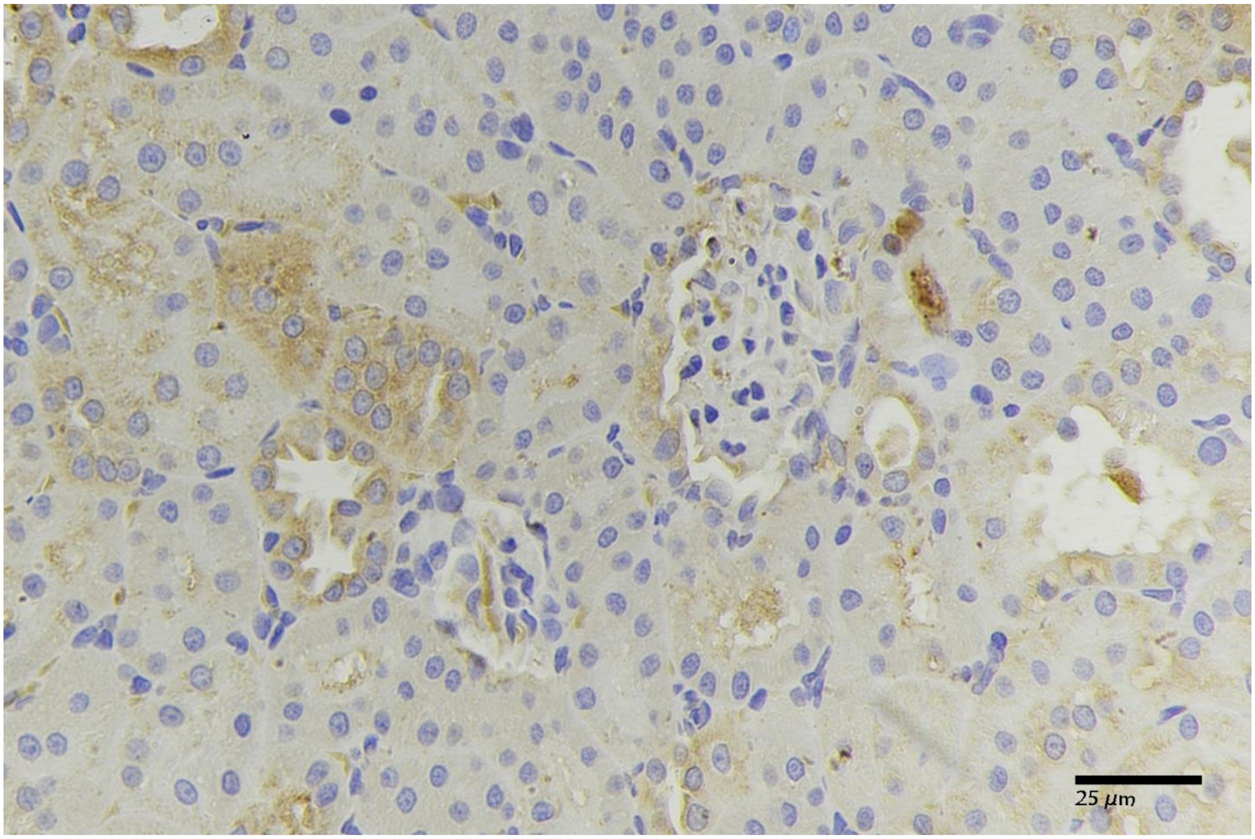
25 μm

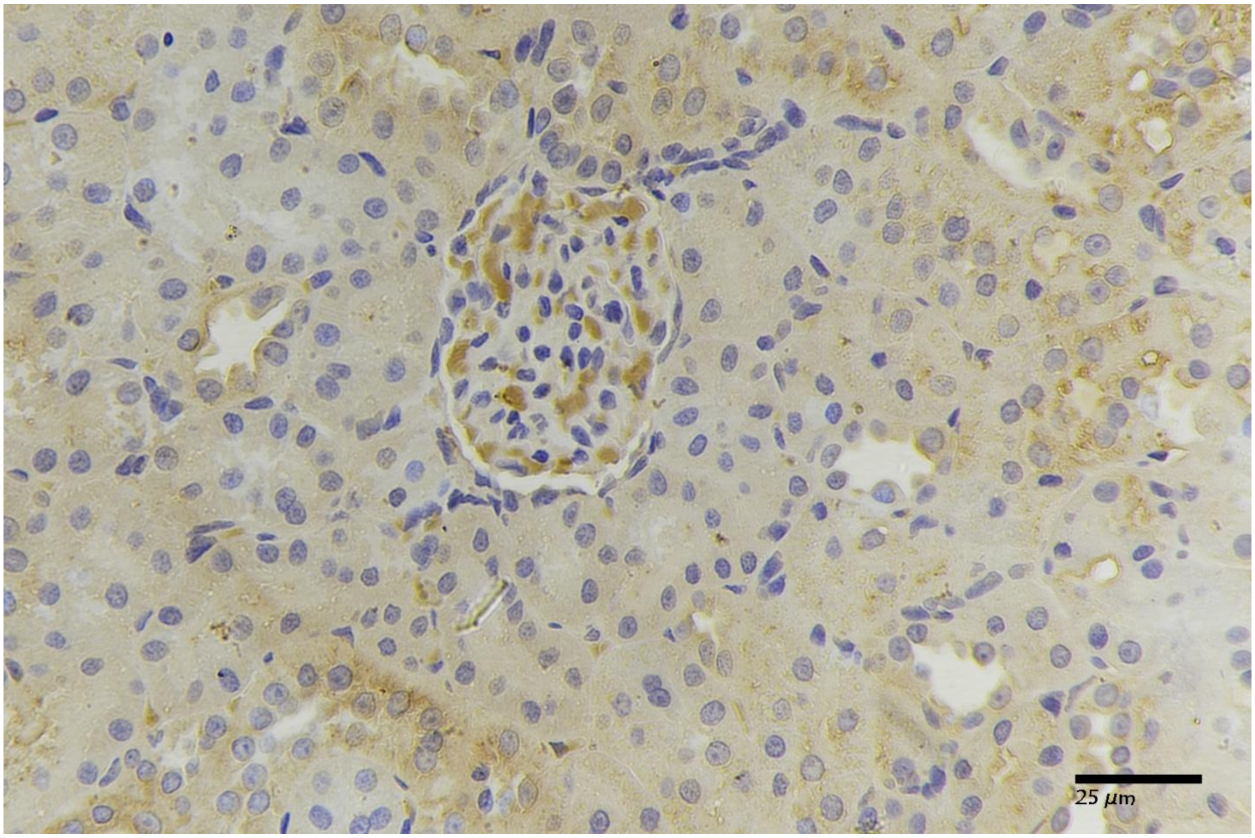
25 μm

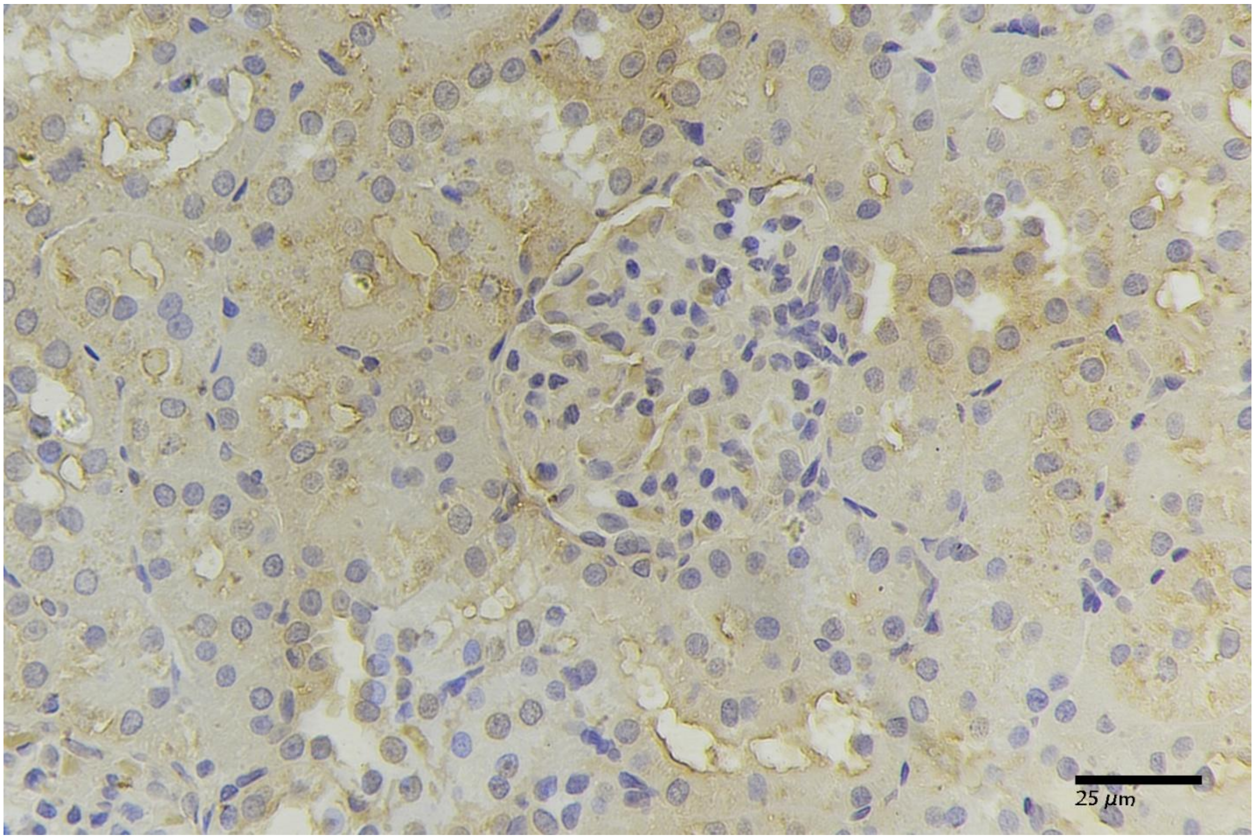
25 μm

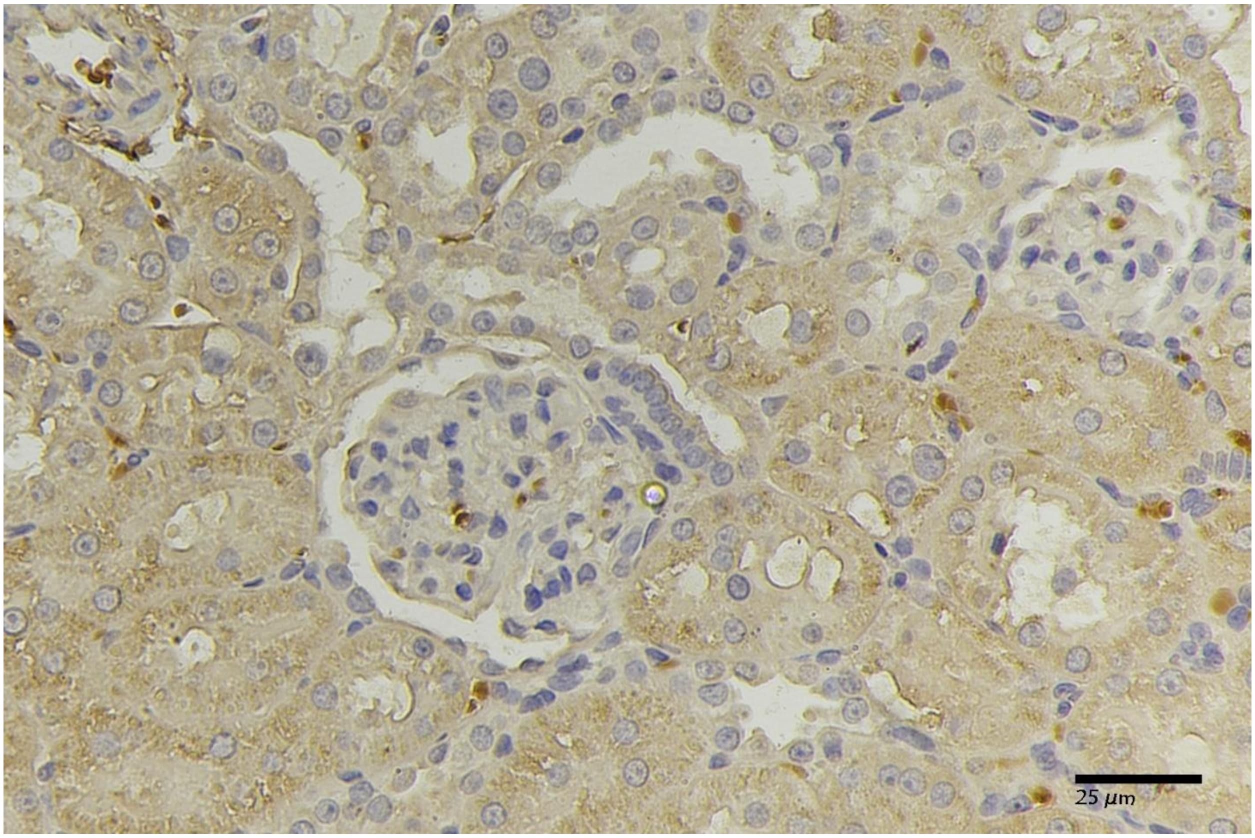
25 μm

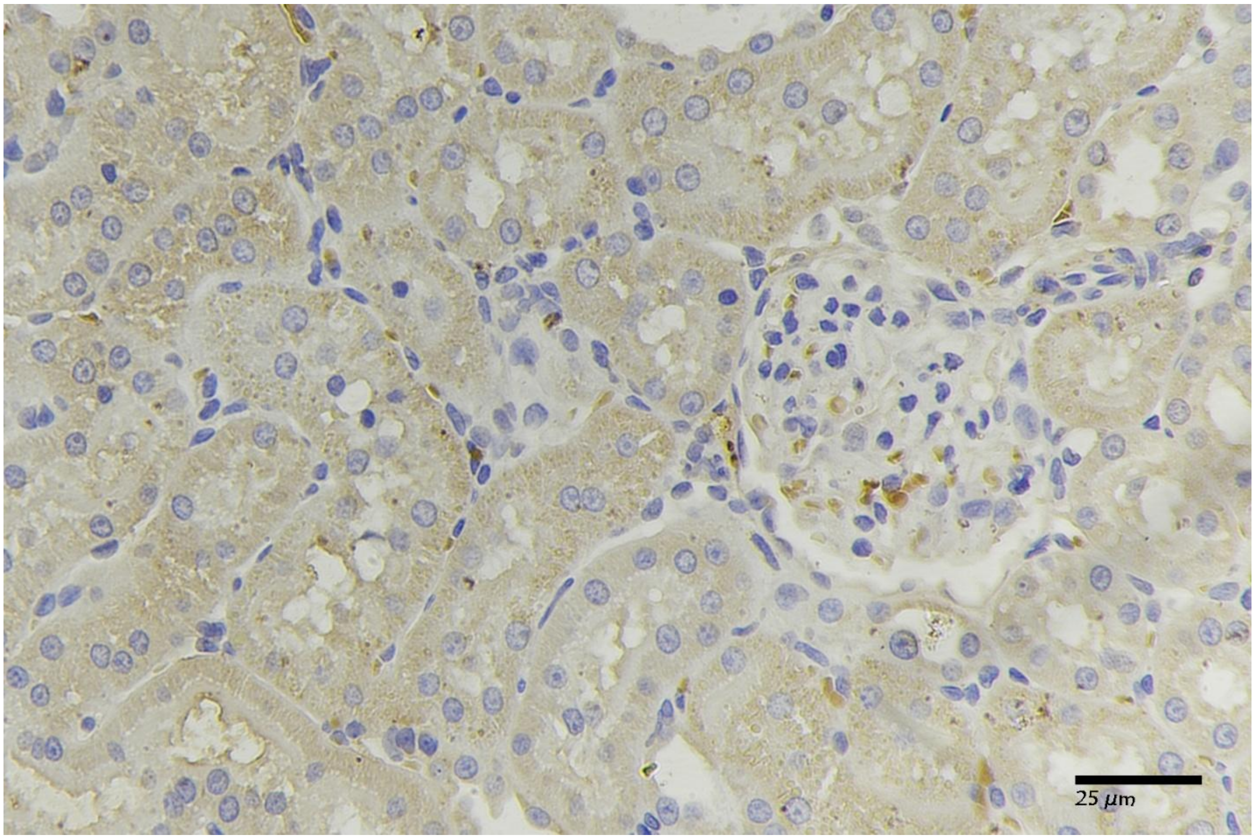
25 μm

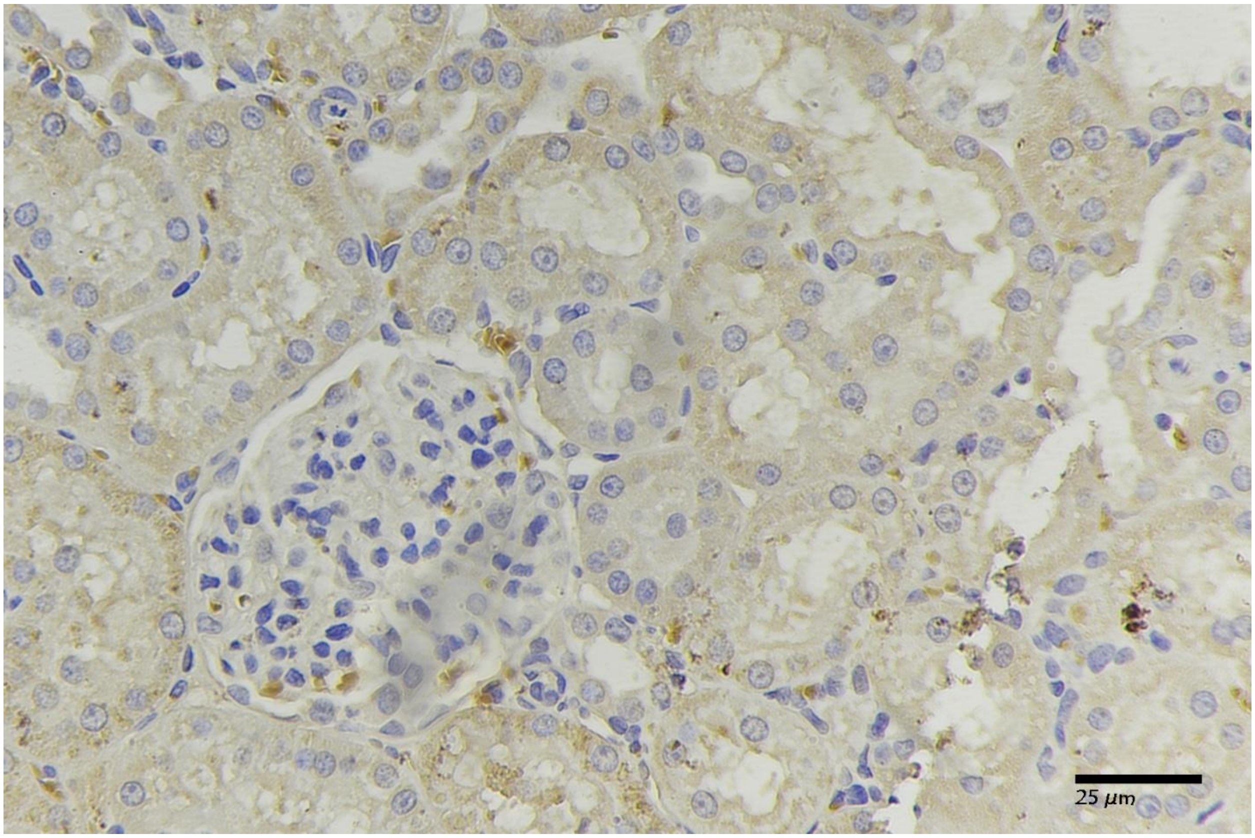
25 μm

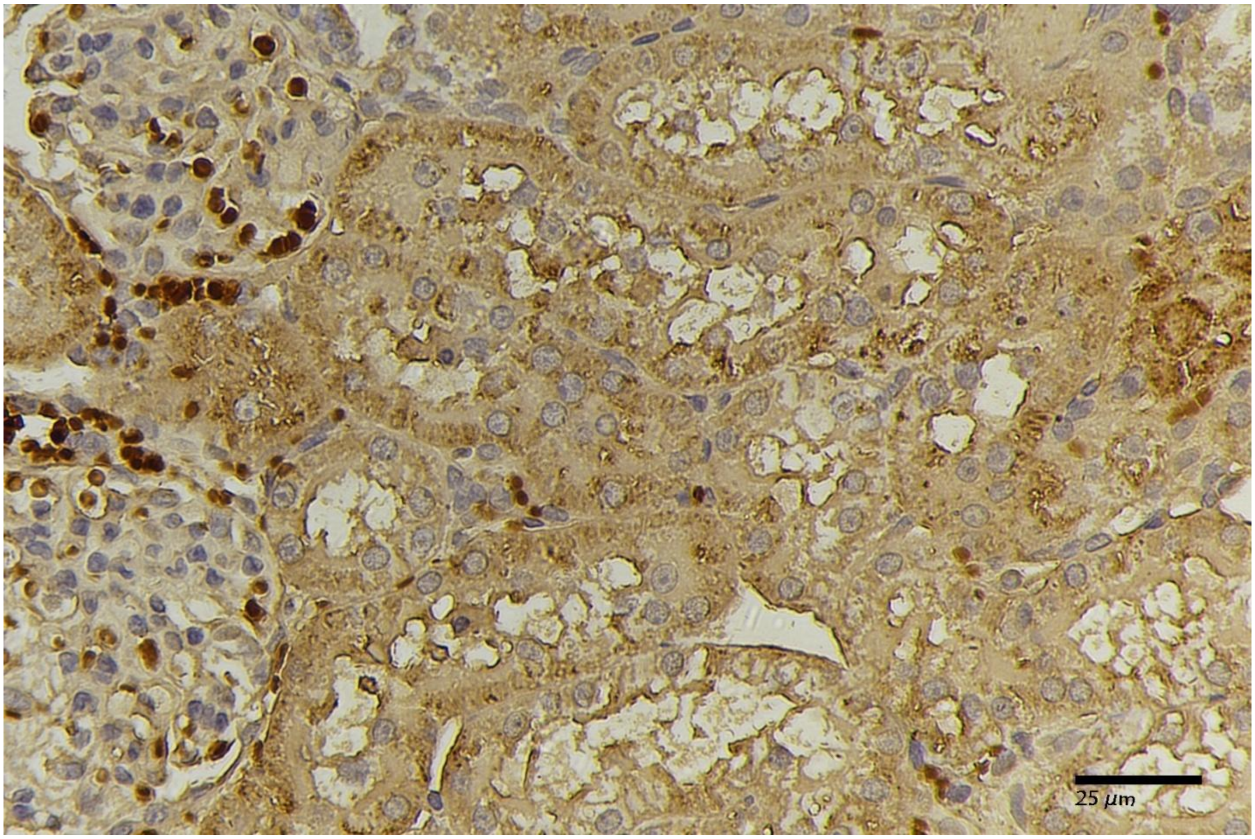
25 μm

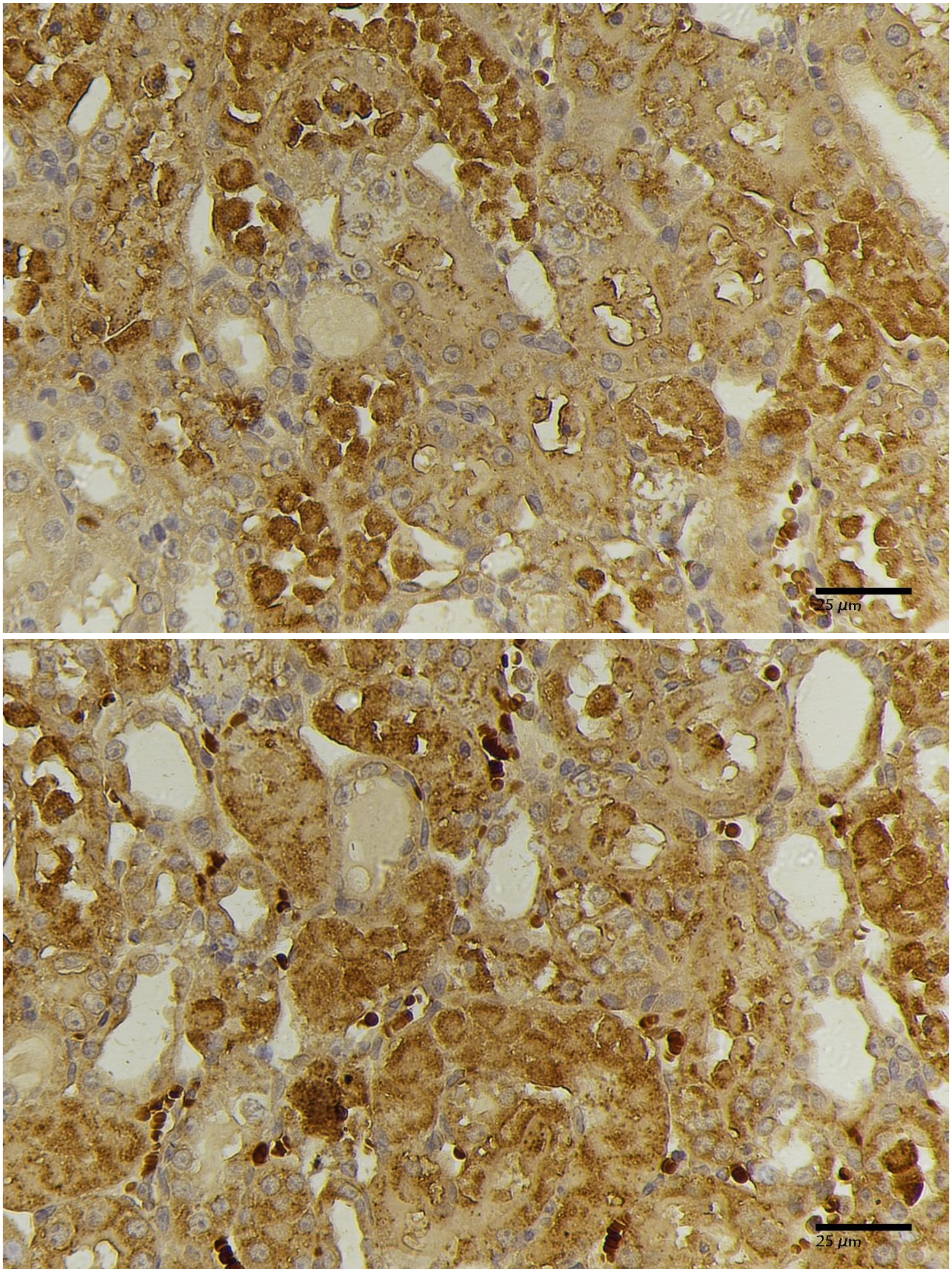
25 μm
25 μm

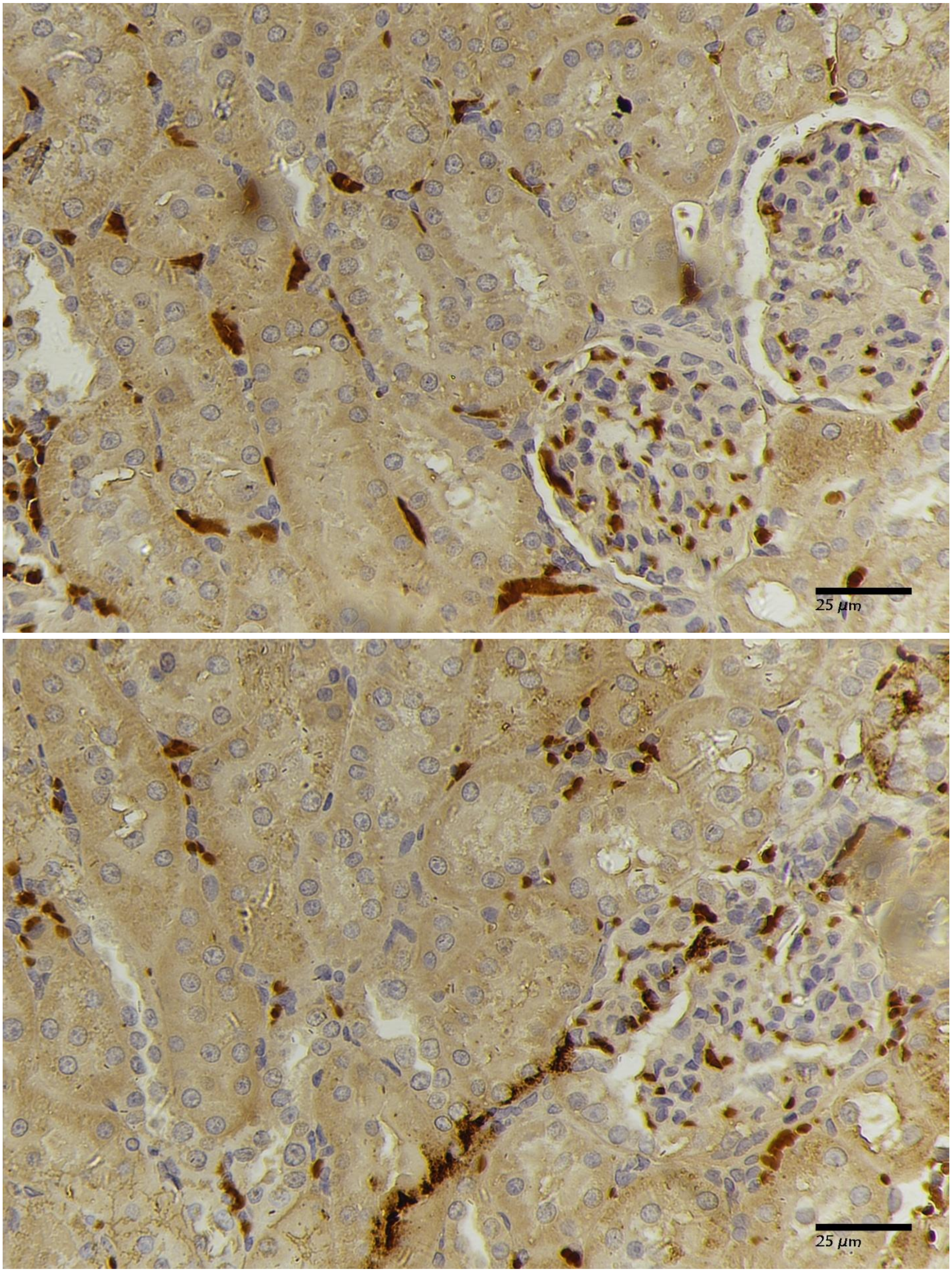
25 μm
25 μm

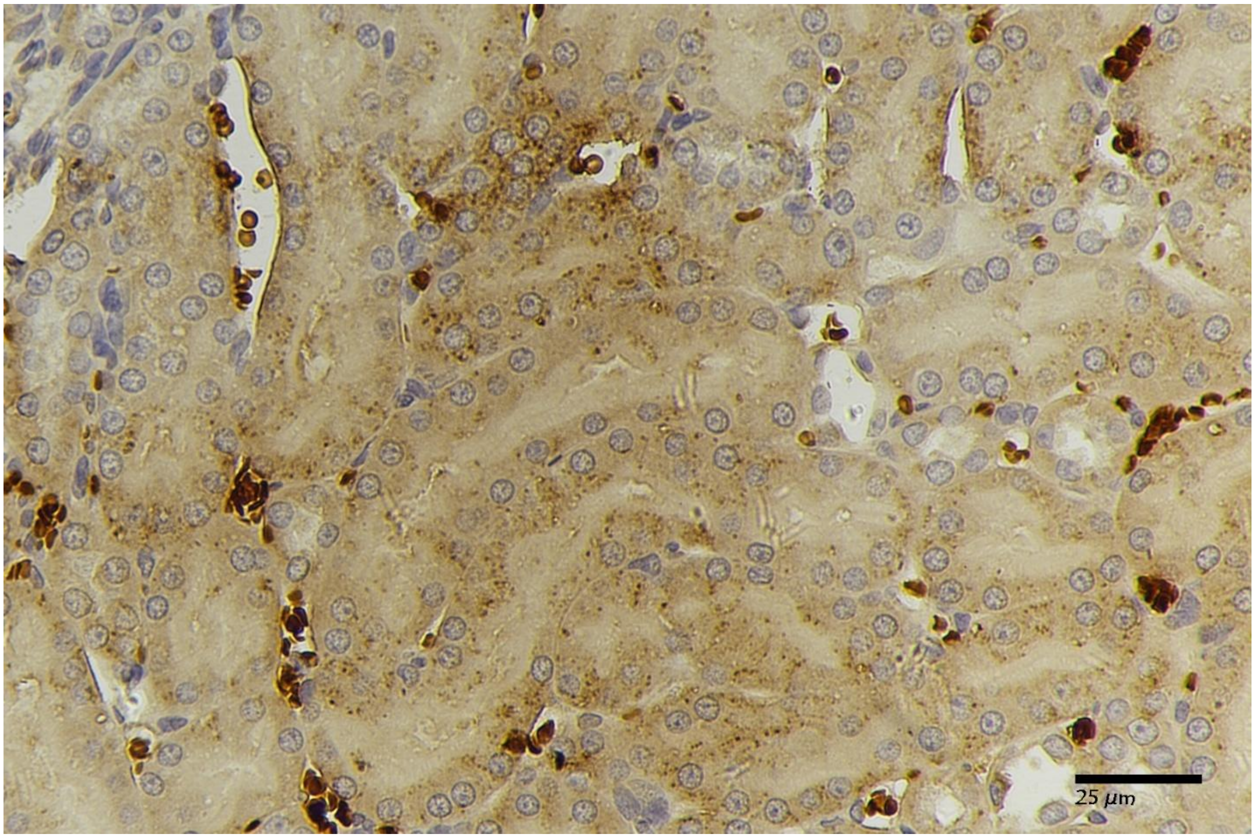
25 μm

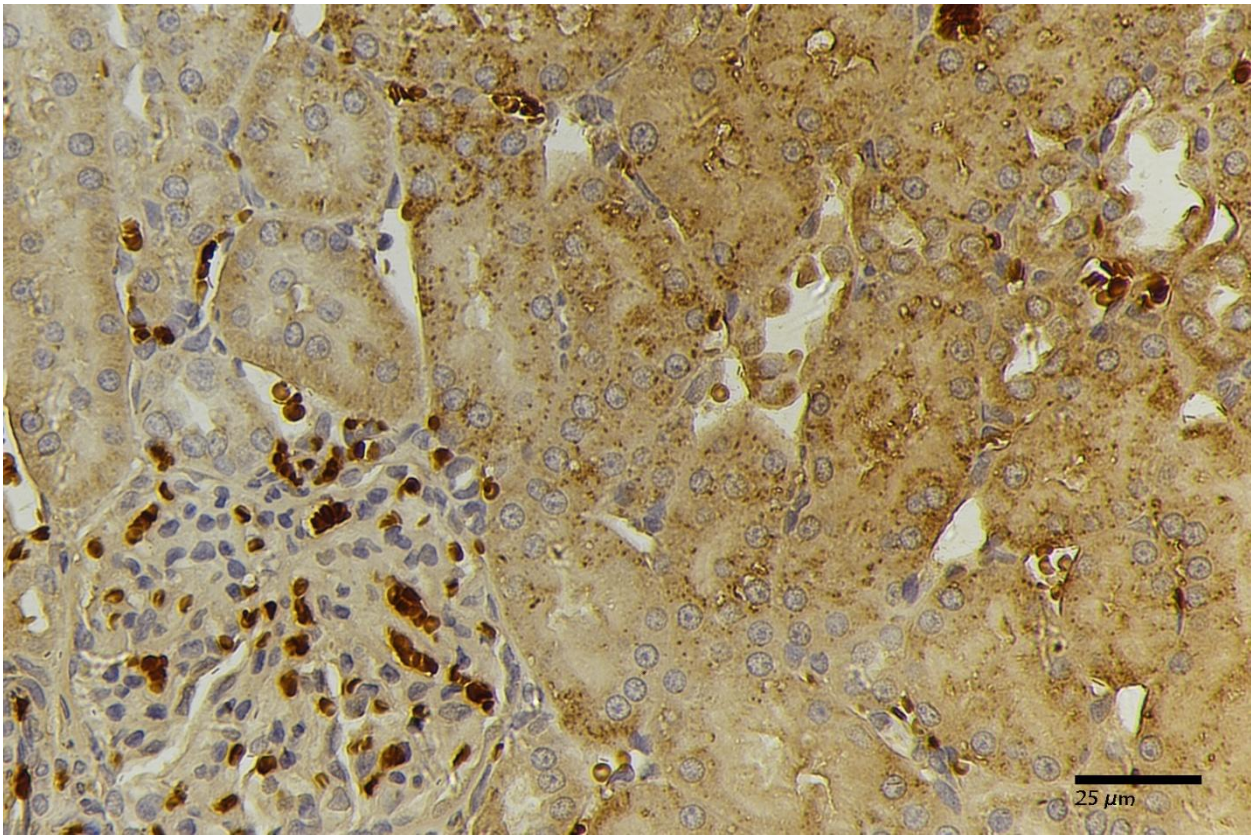
25 μm

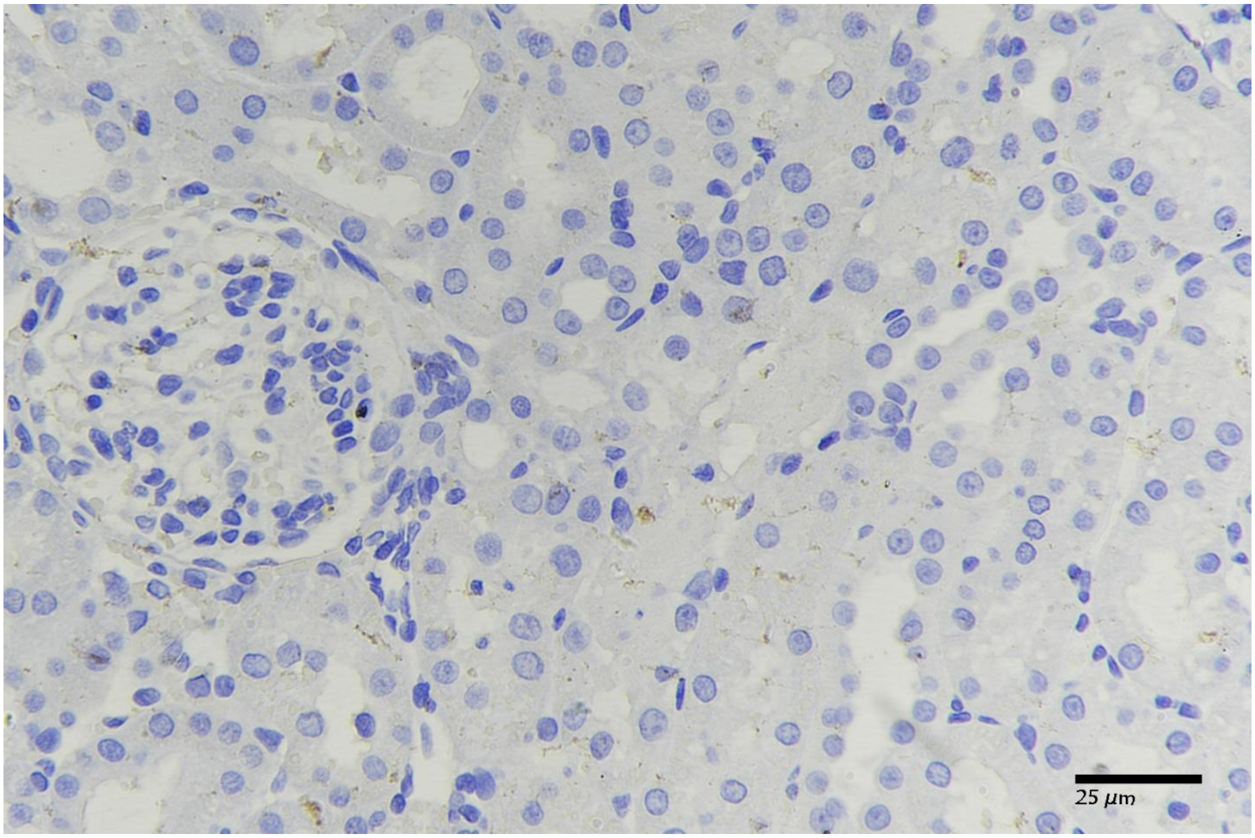
25 μm

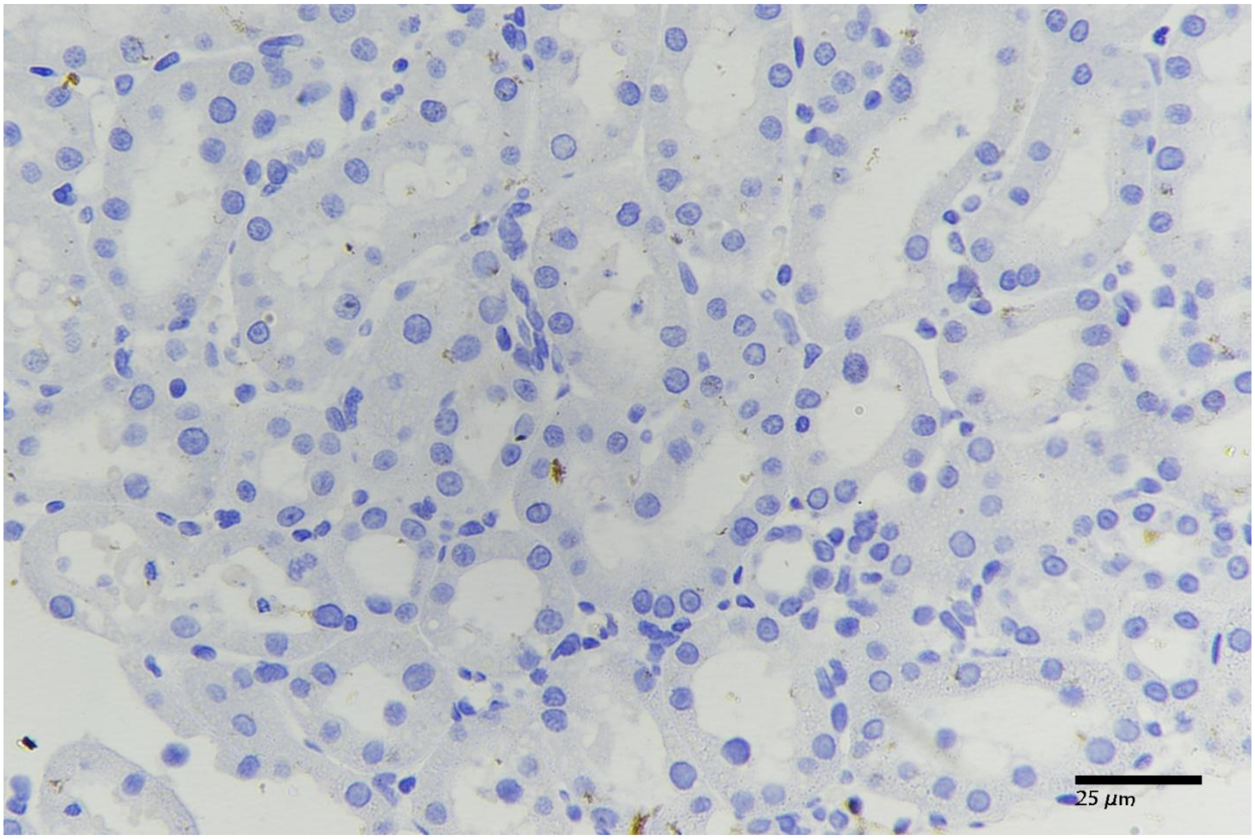
25 μm

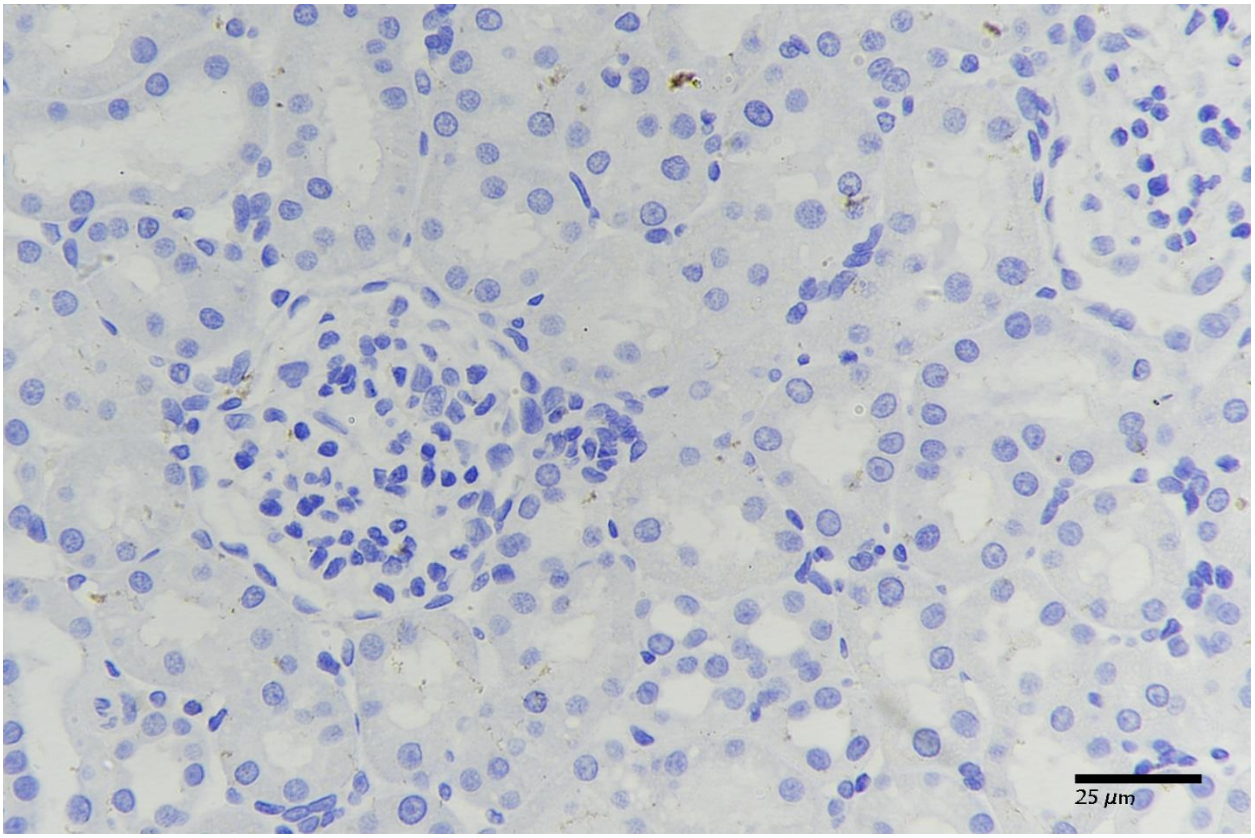
25 μm

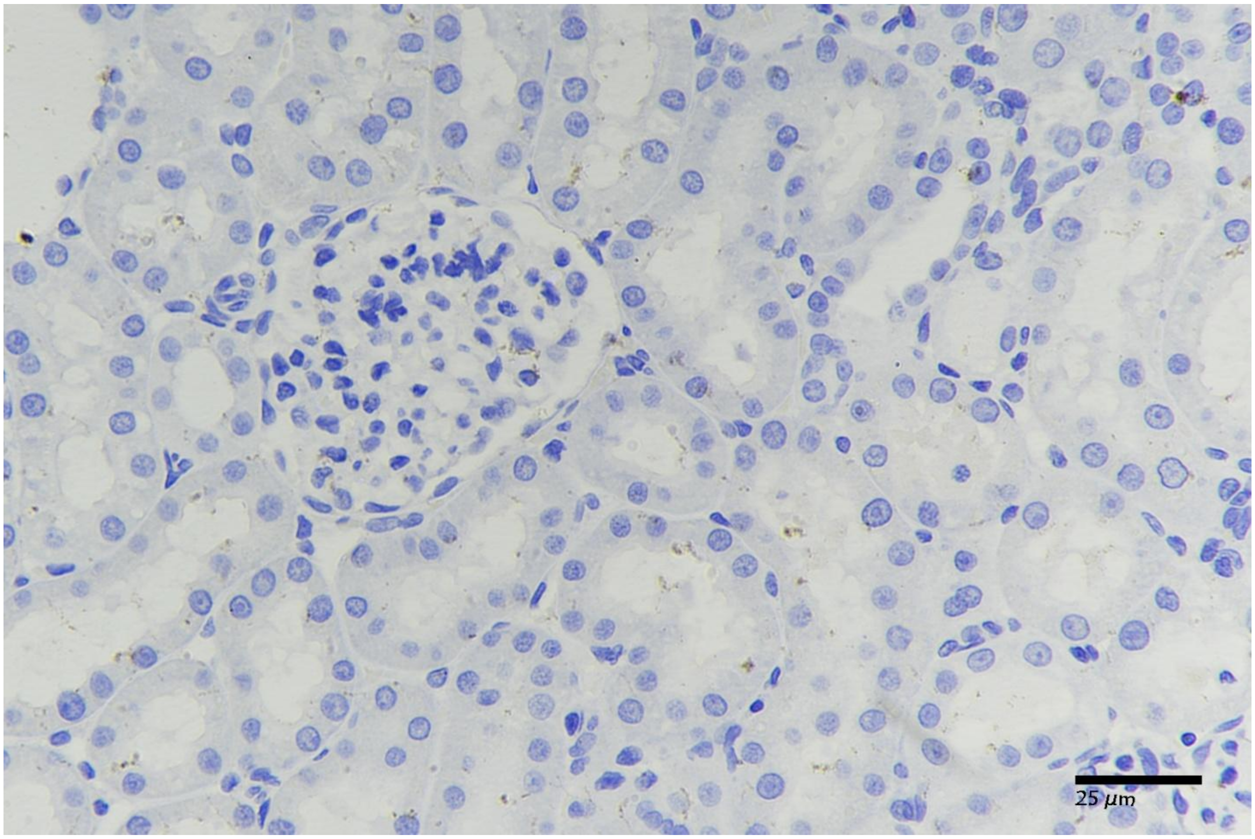
25 μm

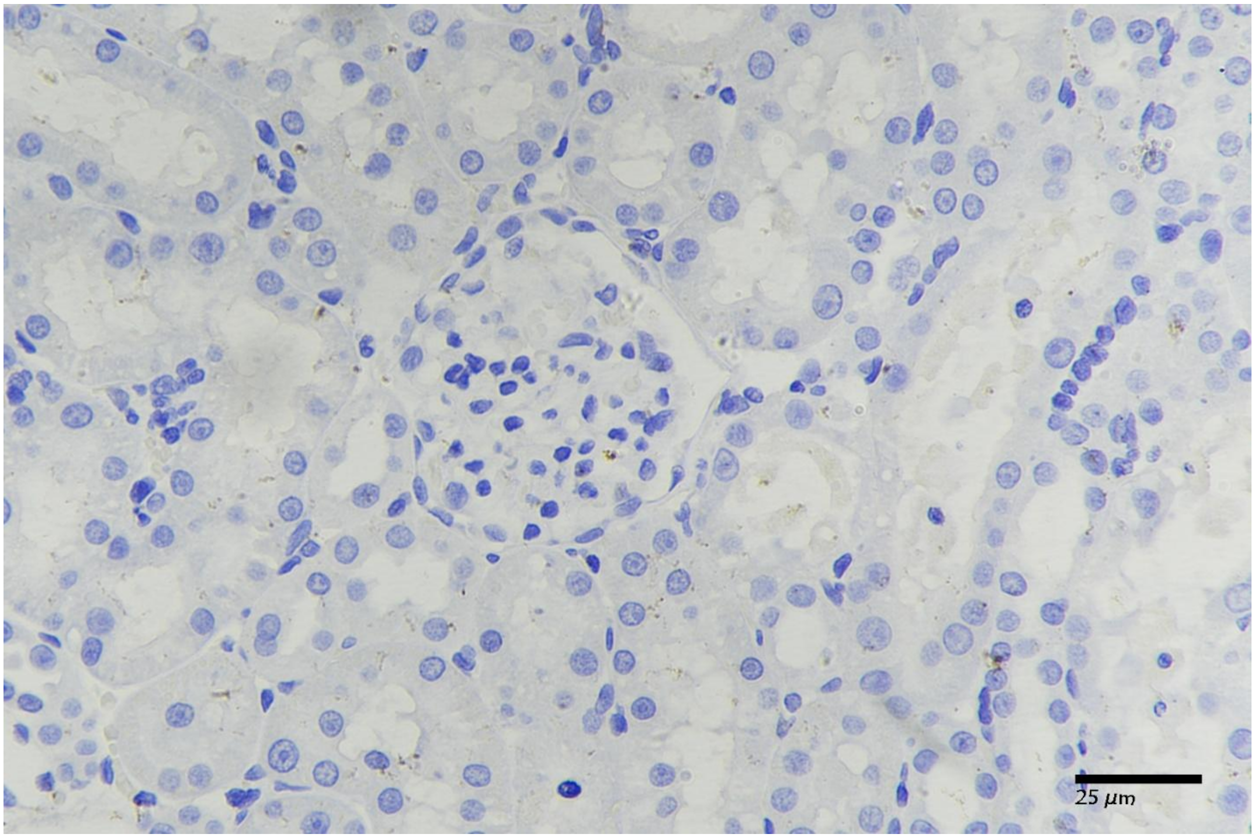
25 µm

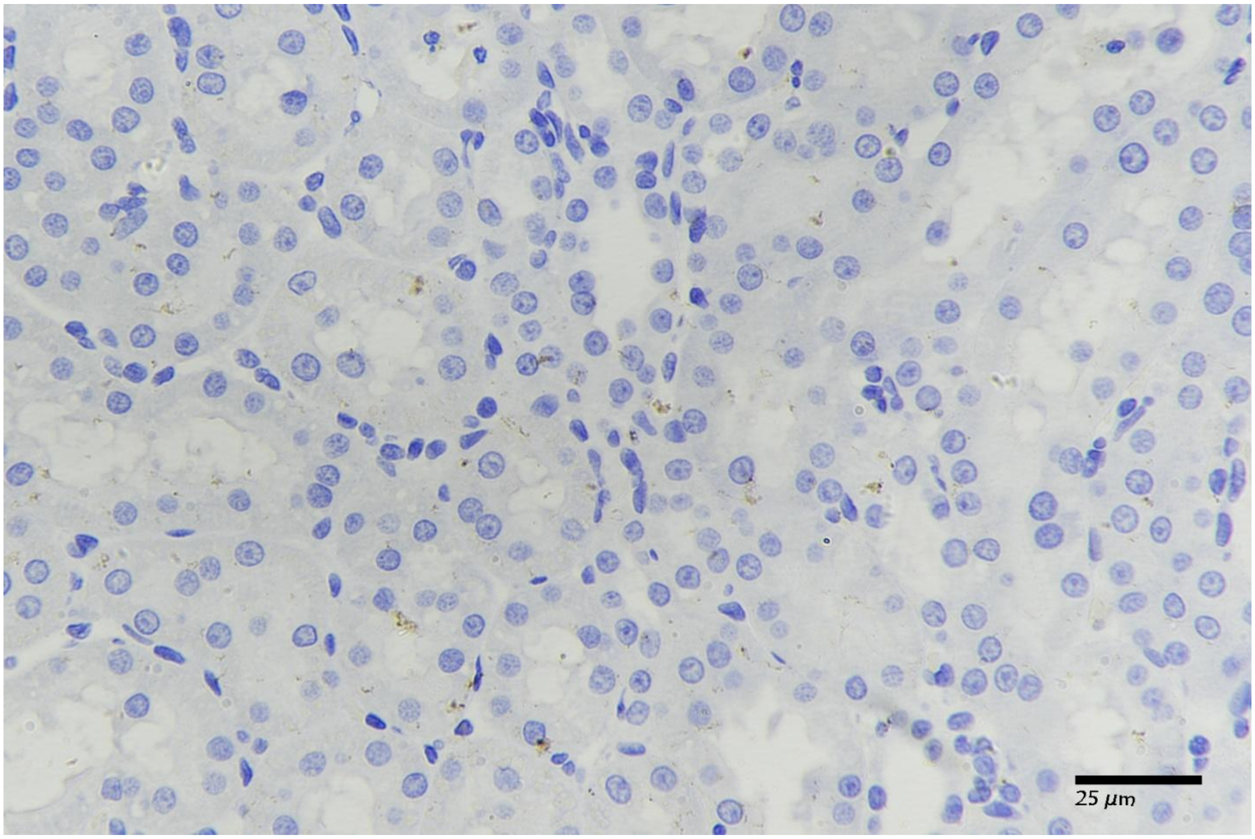
25 µm

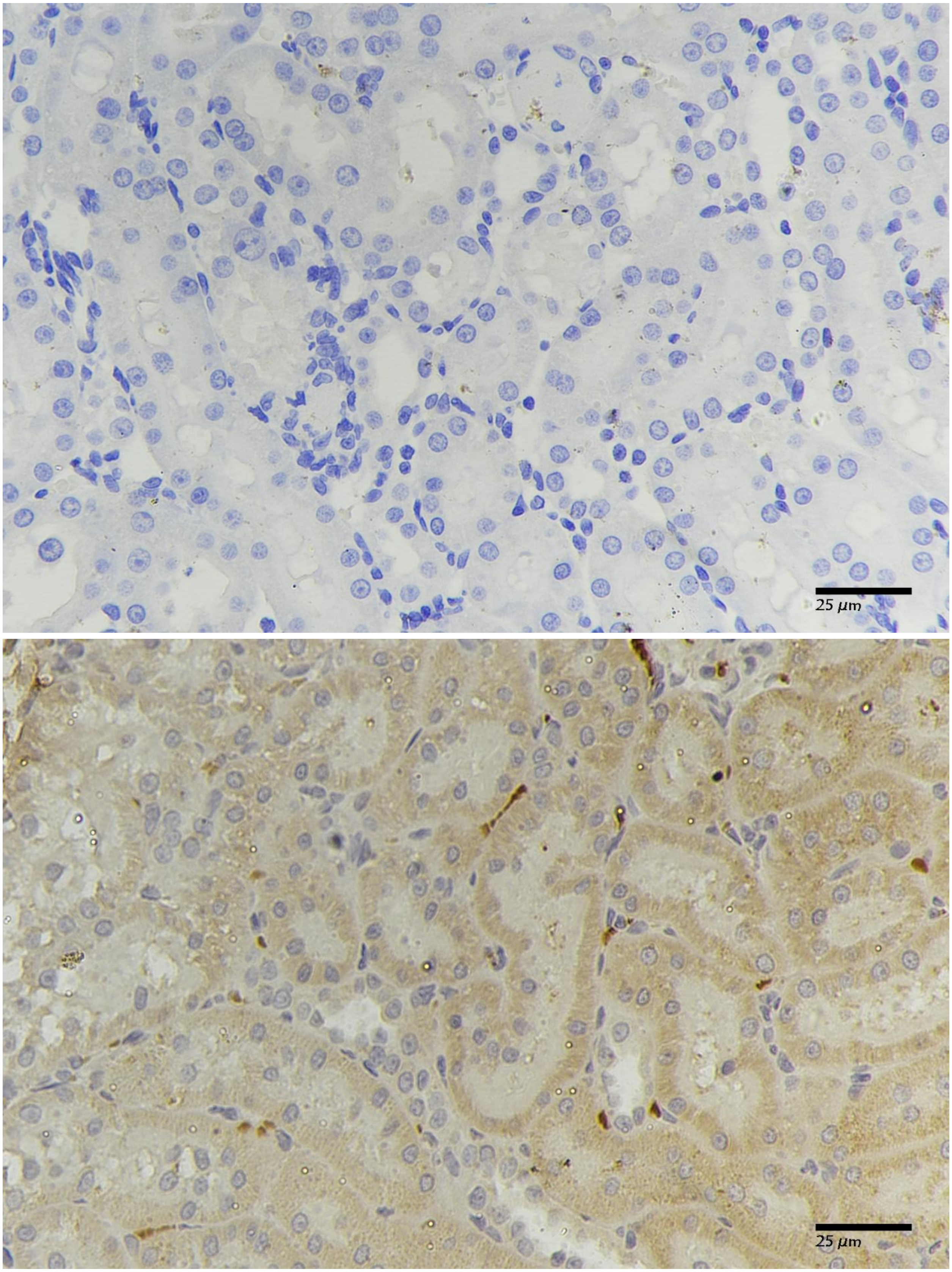
25 μm
25 μm

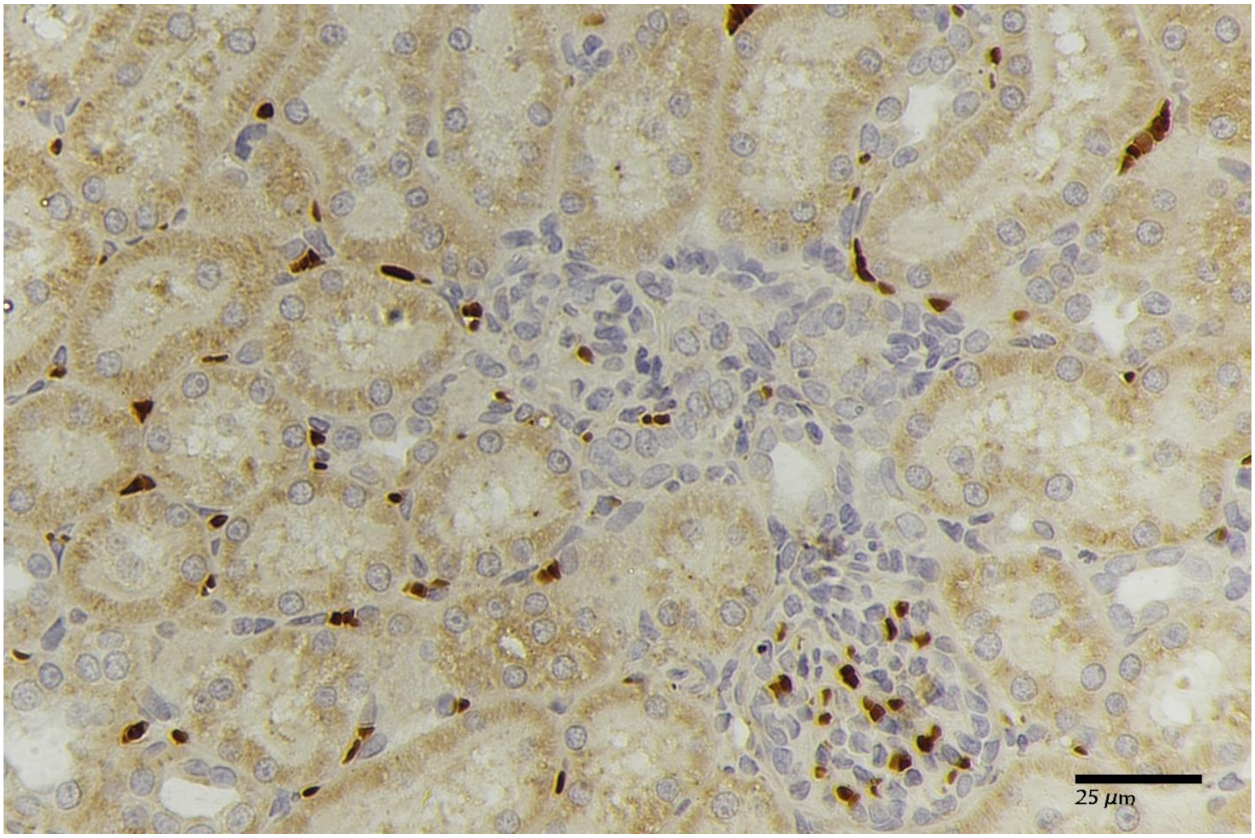
25 μm

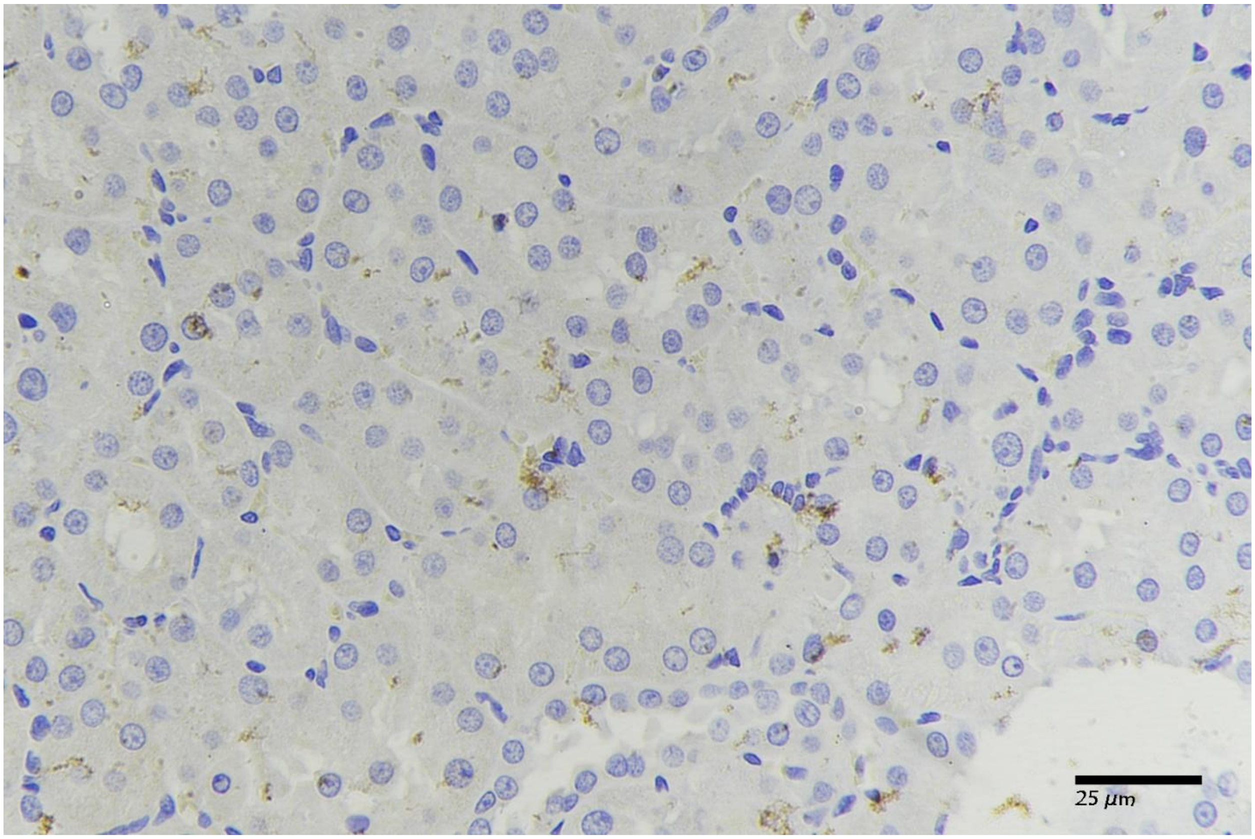
25 μm

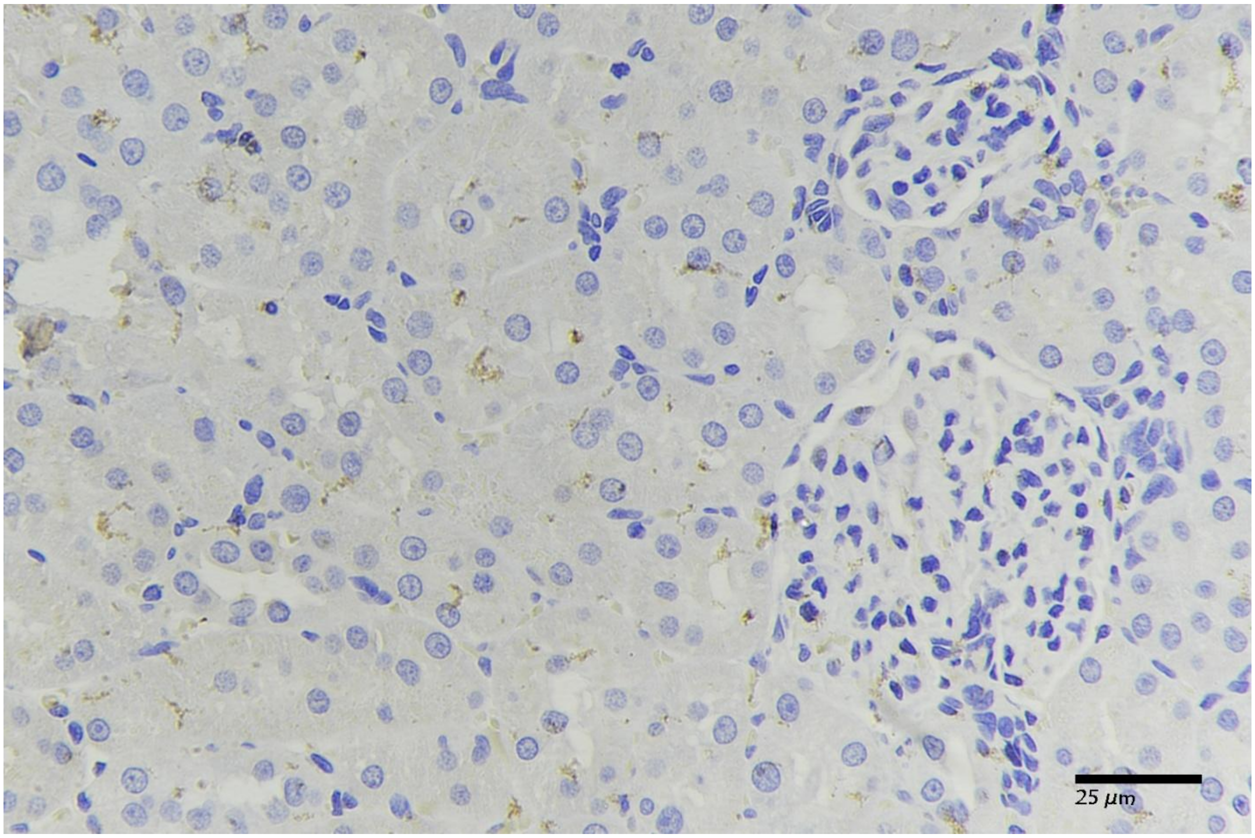
25 μm

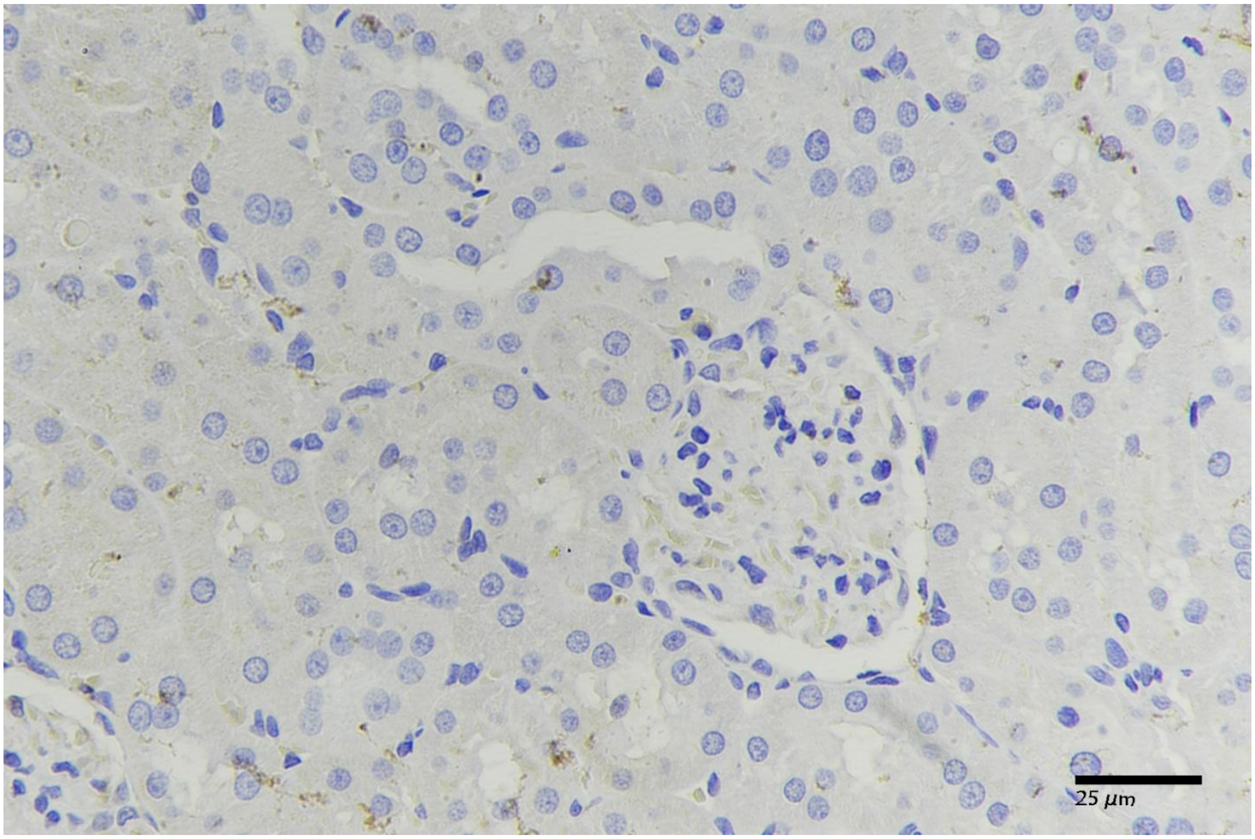
25 μm

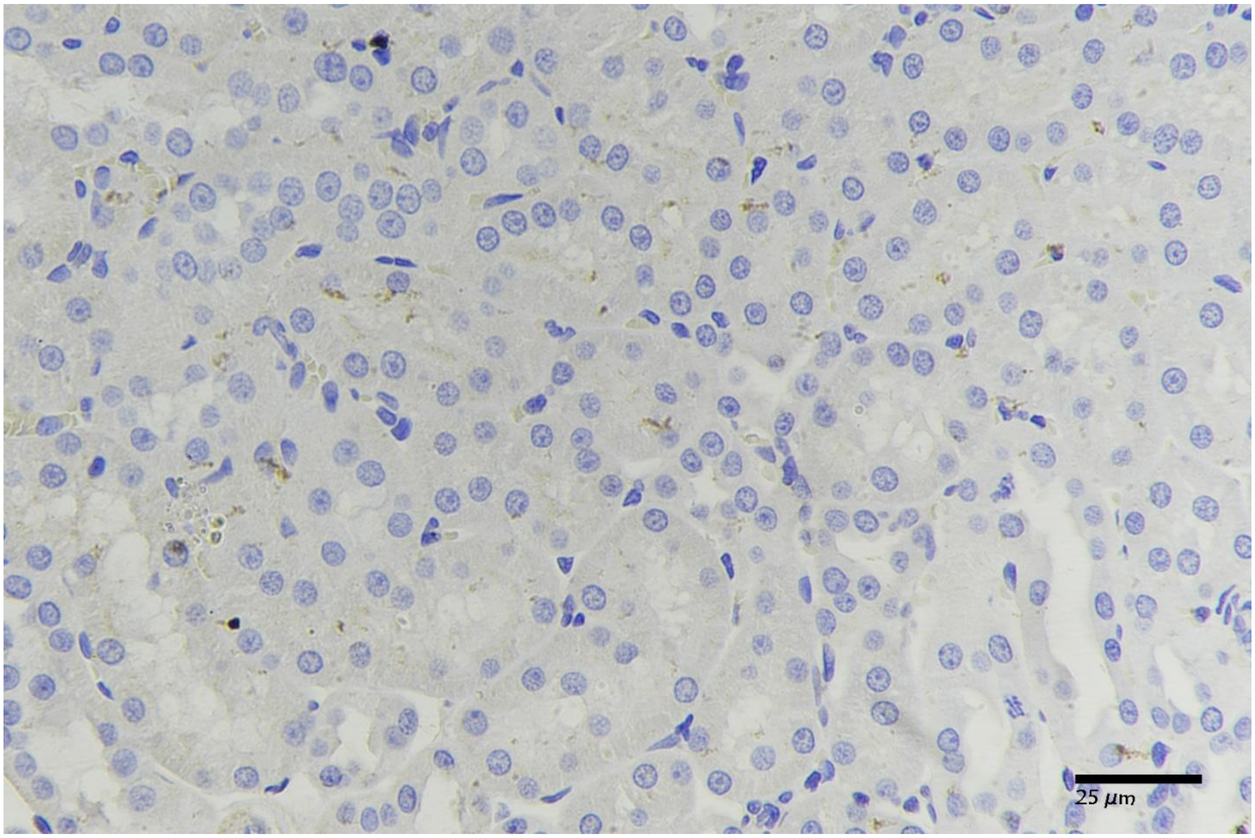
25 μm

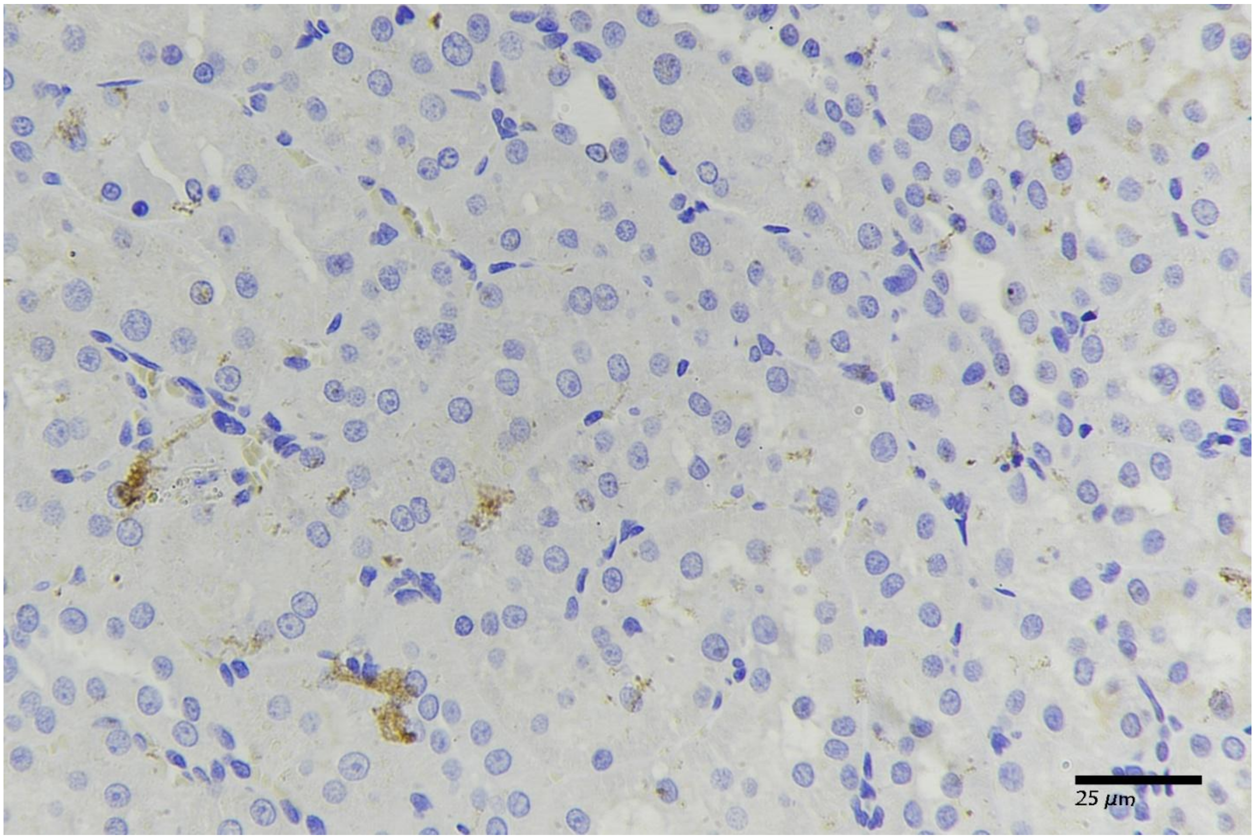
25 μm

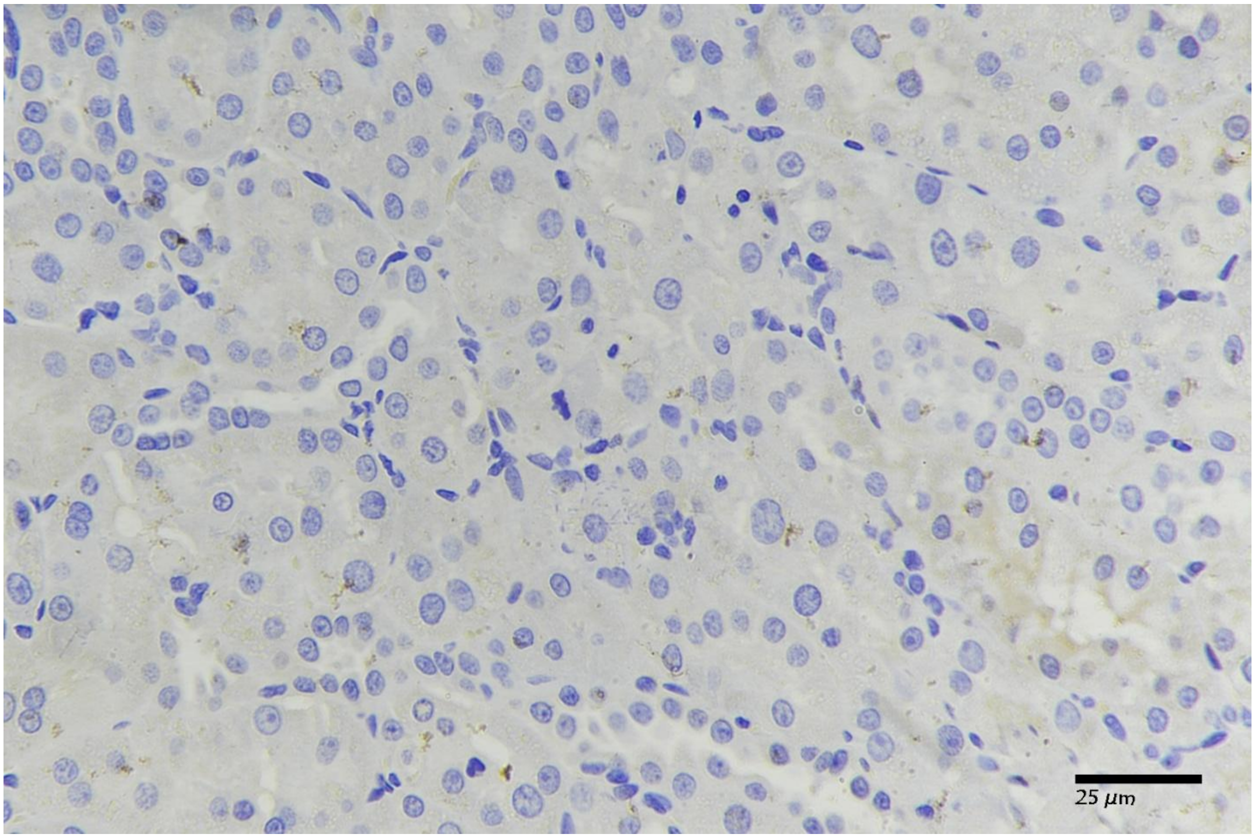
25 μm

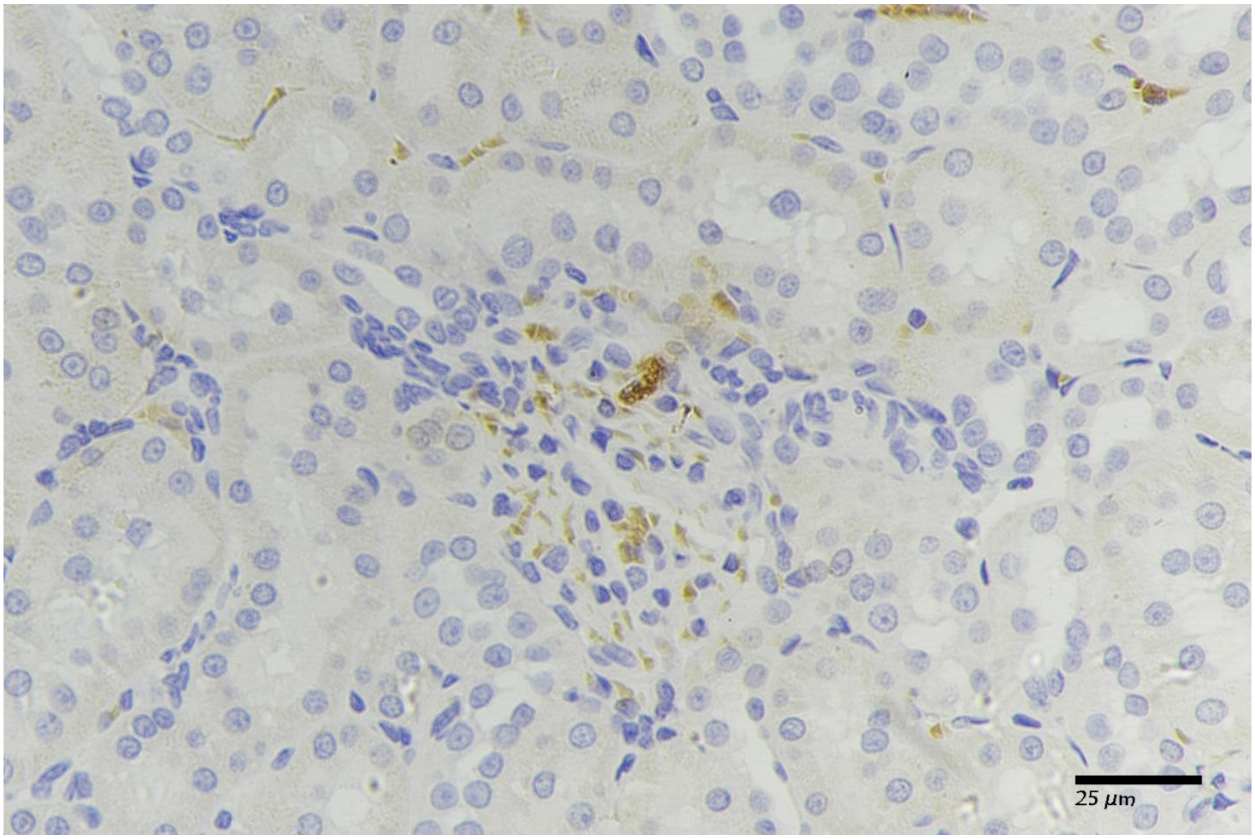
25 μm

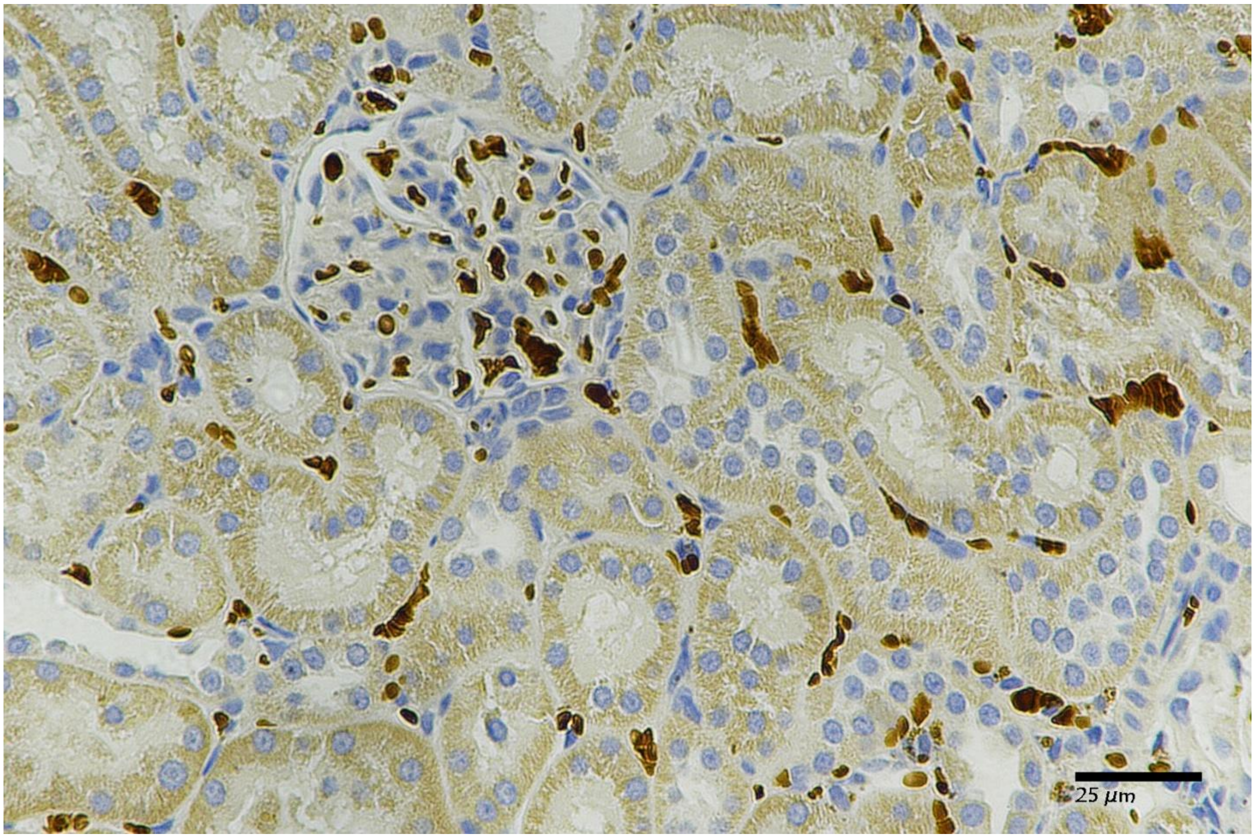
25 μm

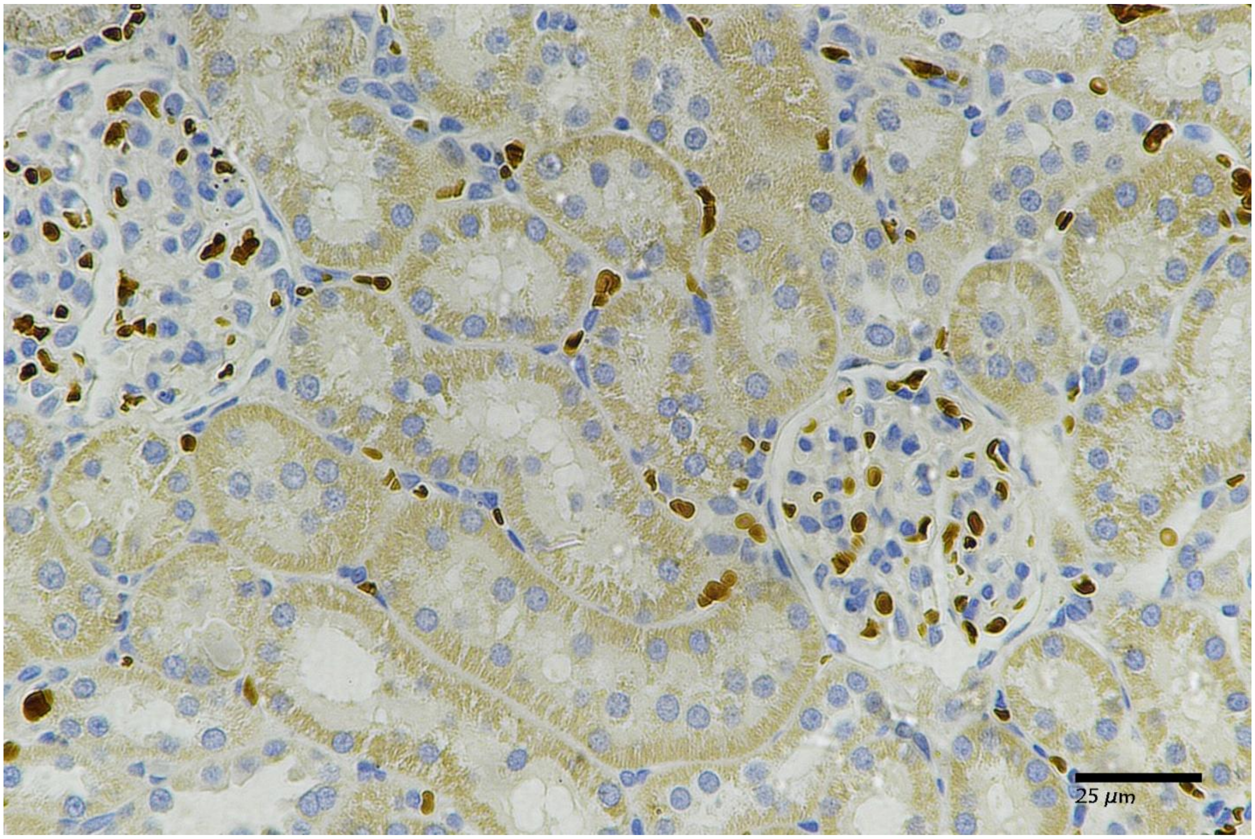
25 μm

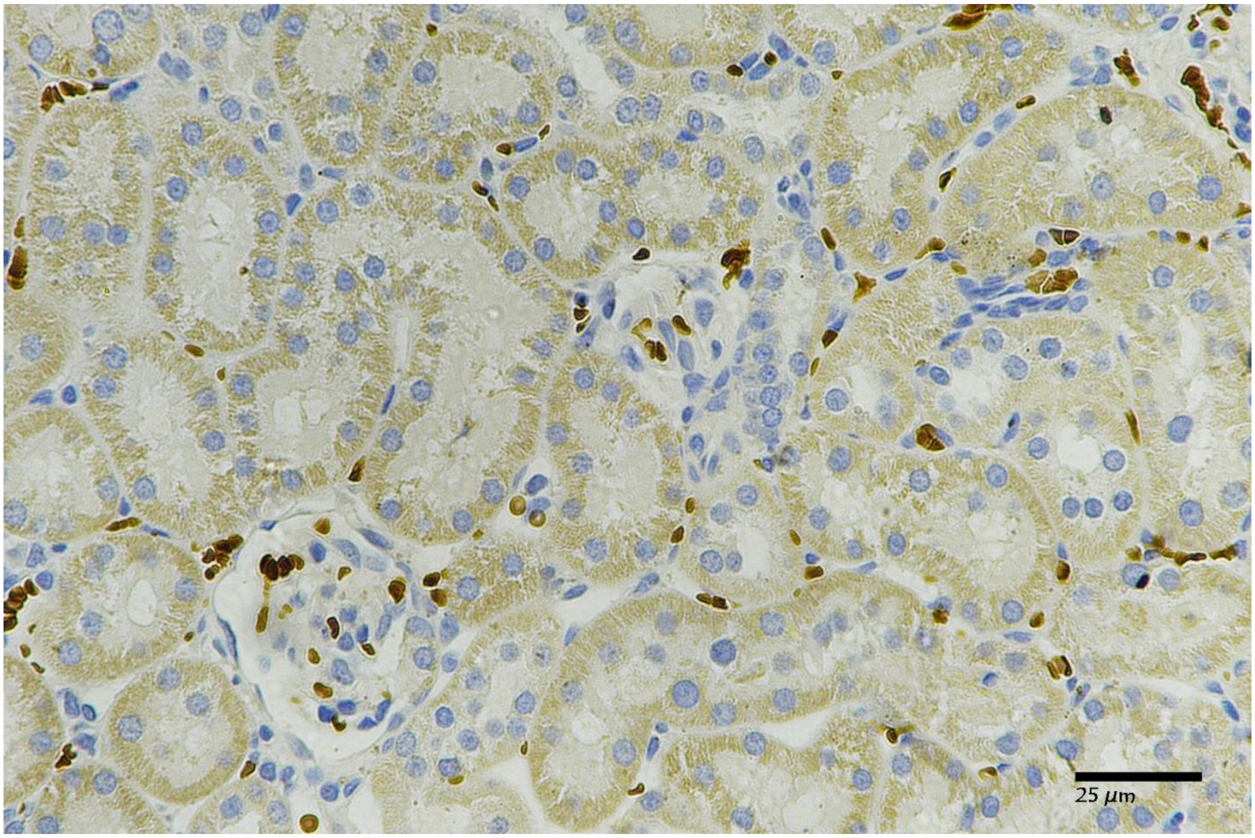
25 μm

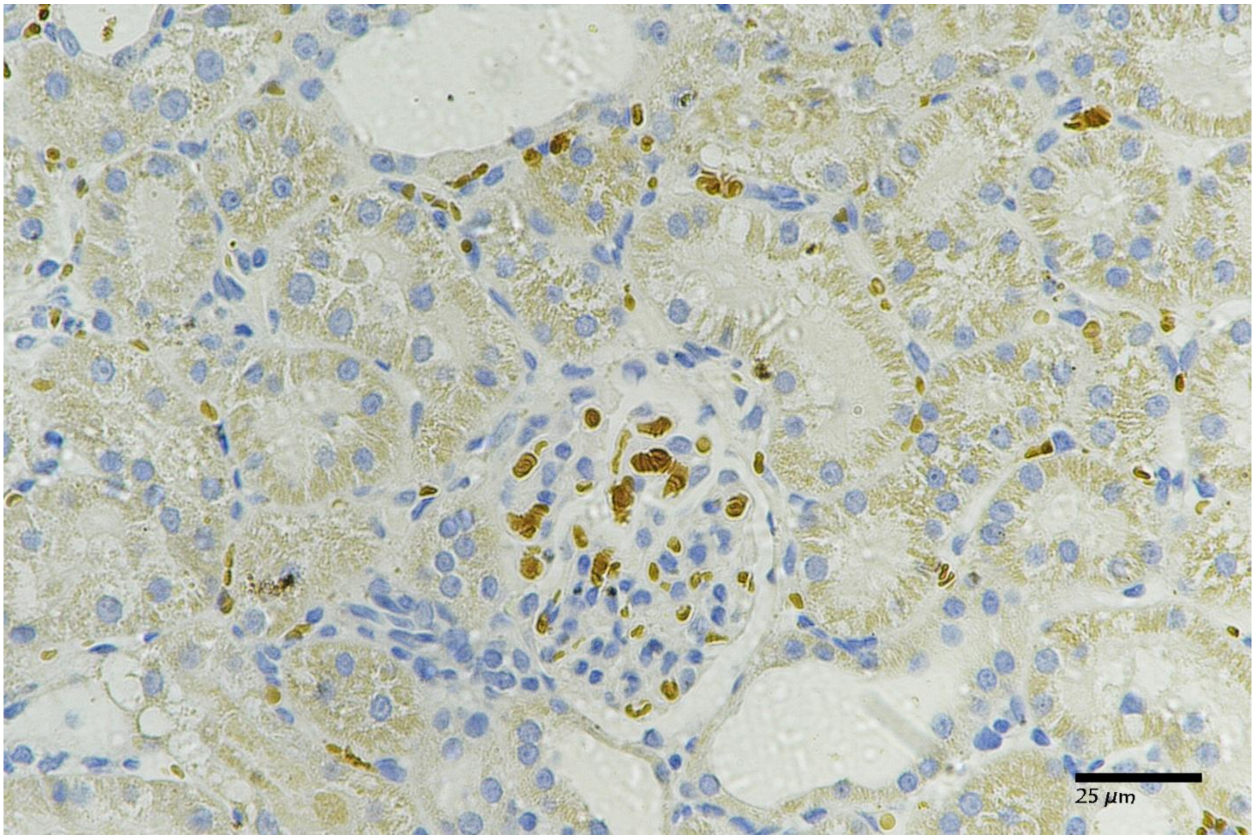
25 μm

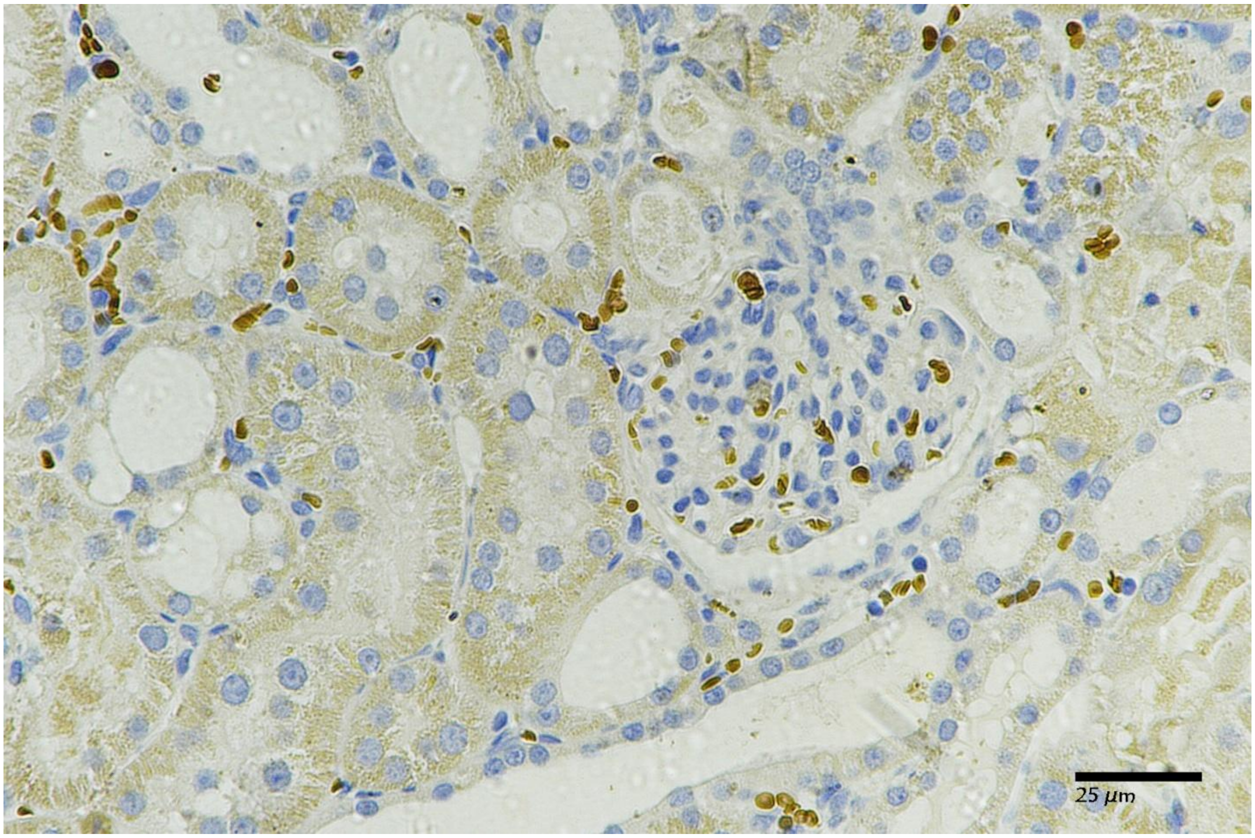
25 μm

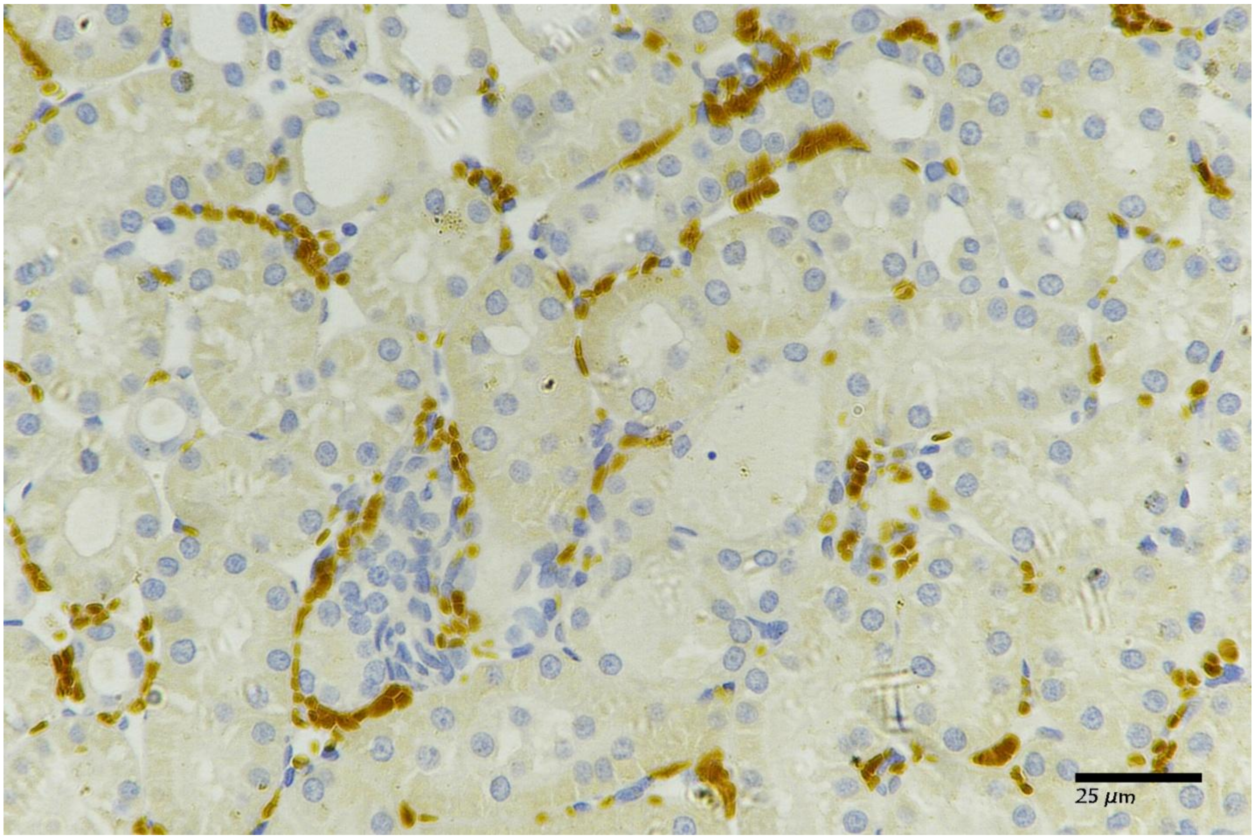
25 μm

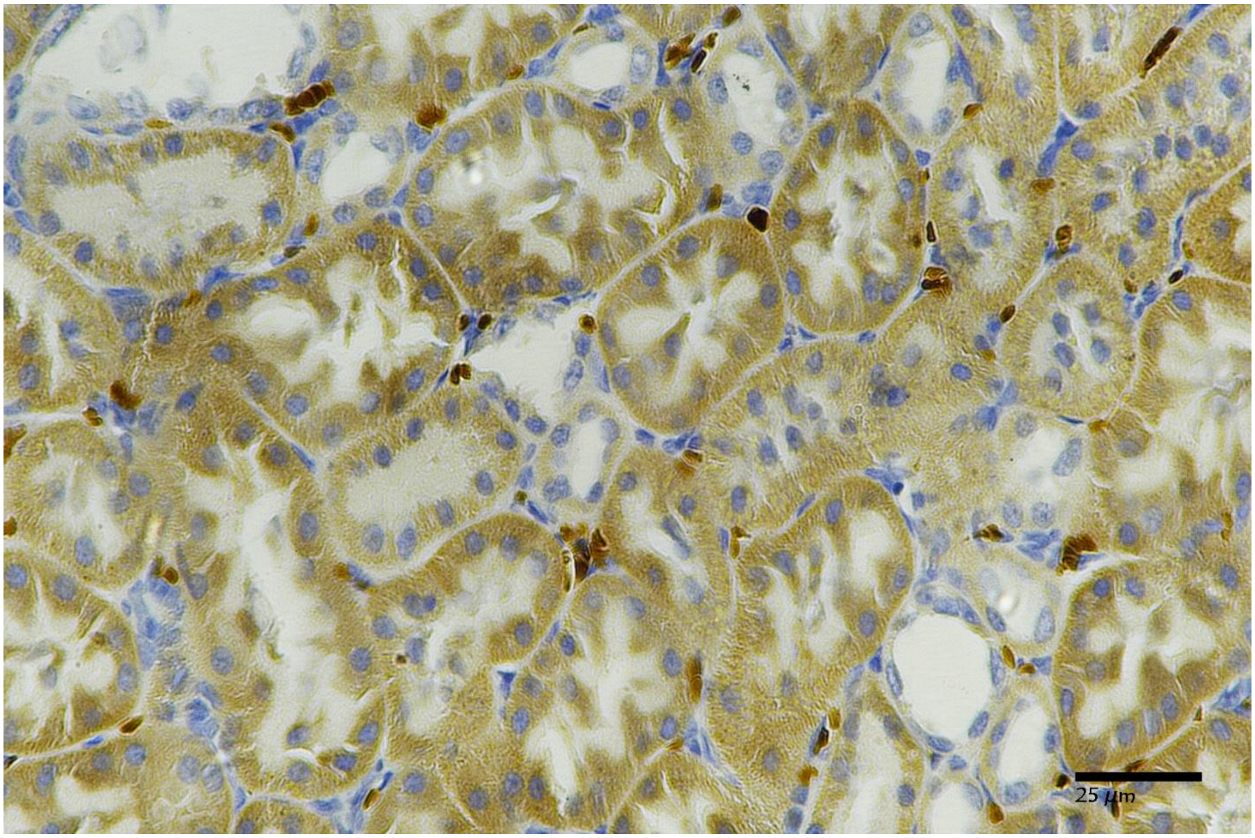
25 μm

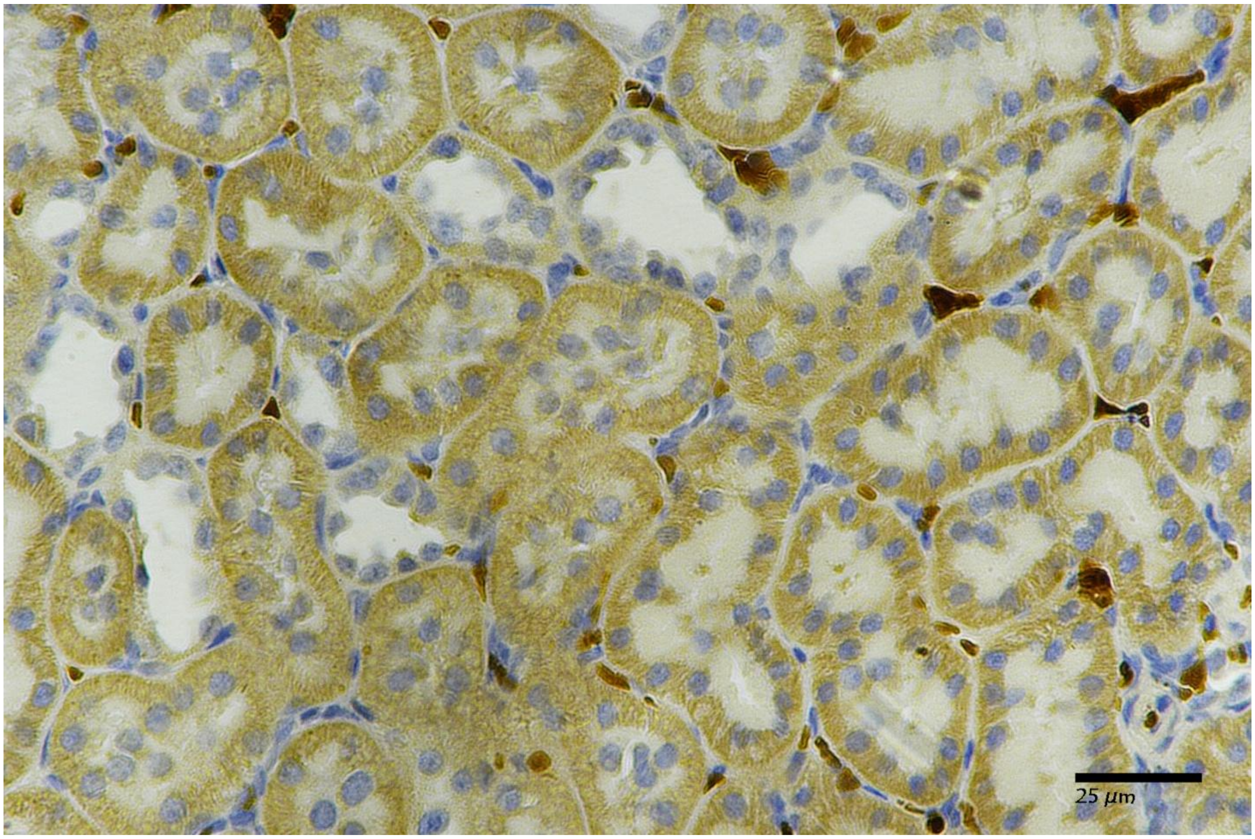
25 μm

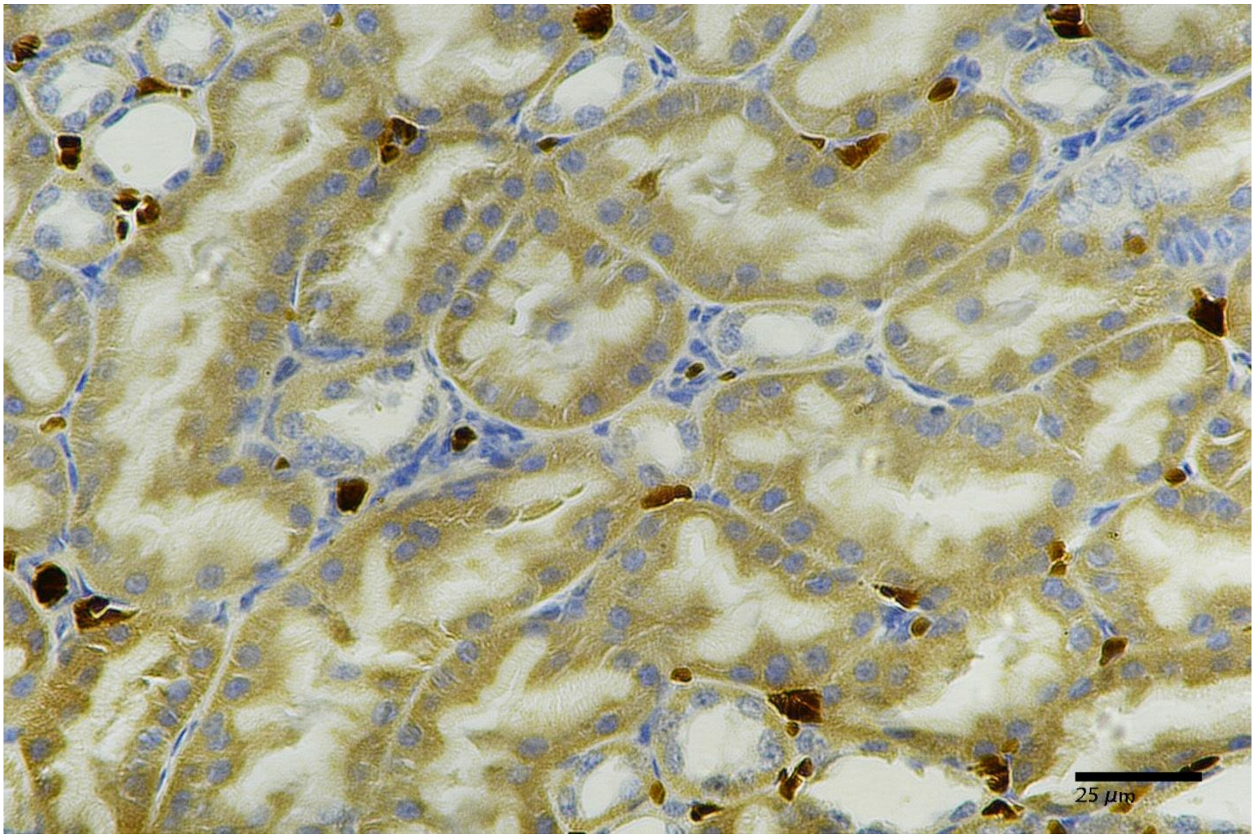
25 µm

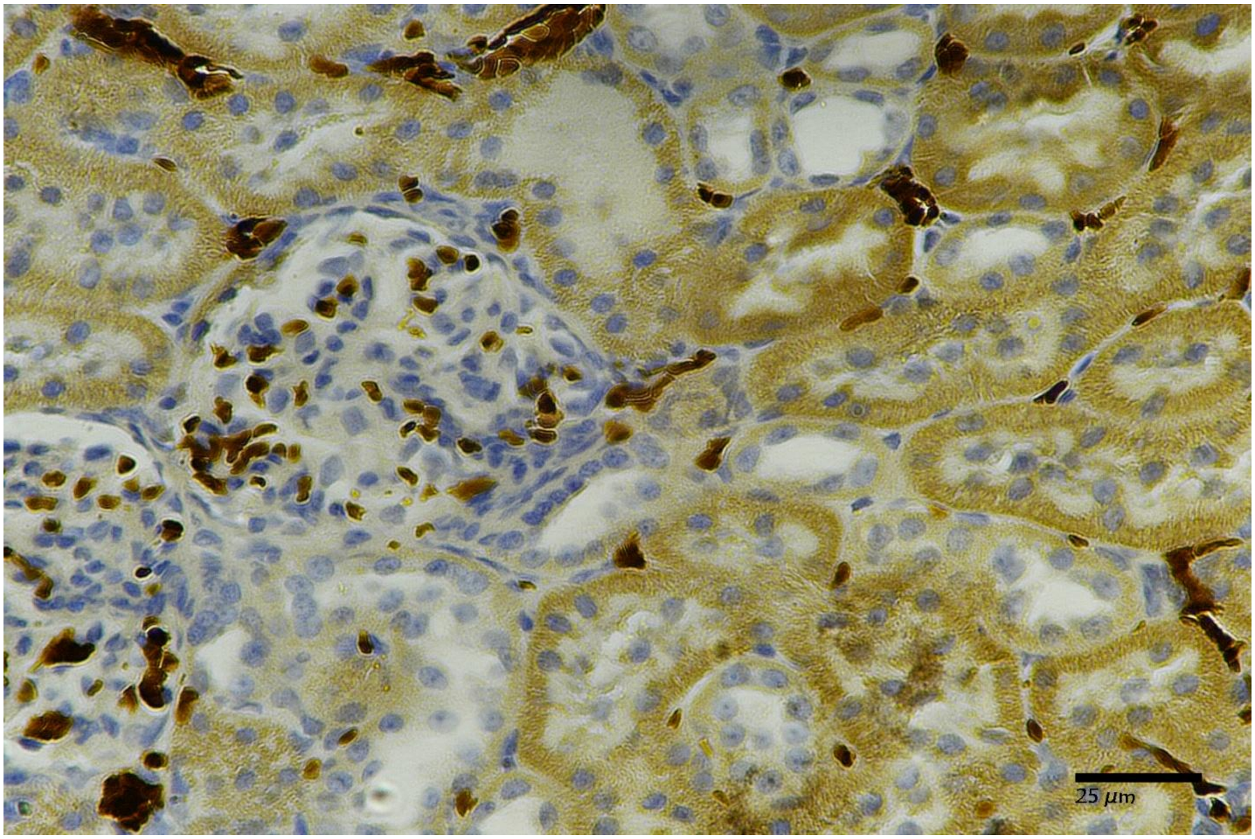
25 µm

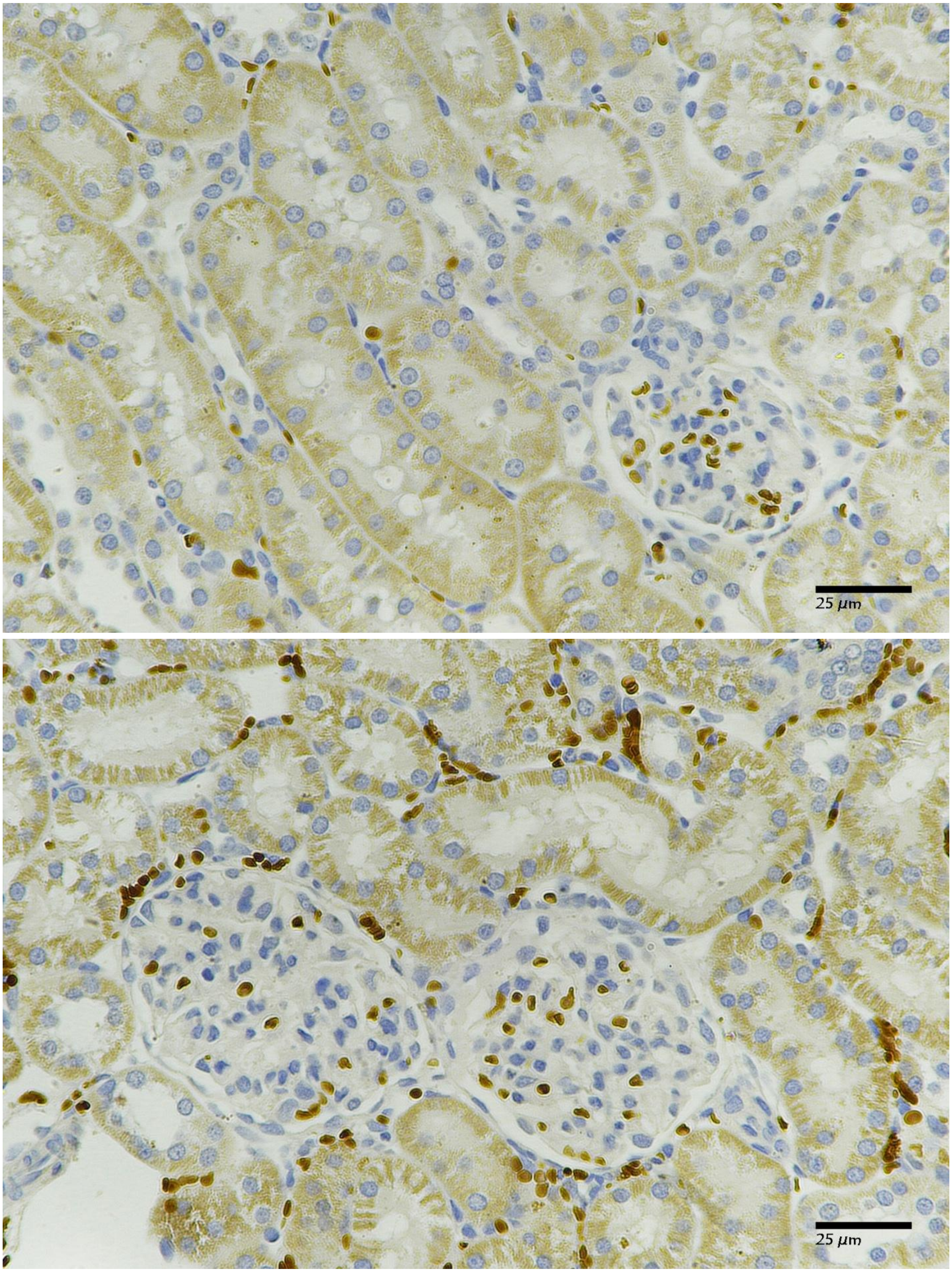
25 μm
25 μm

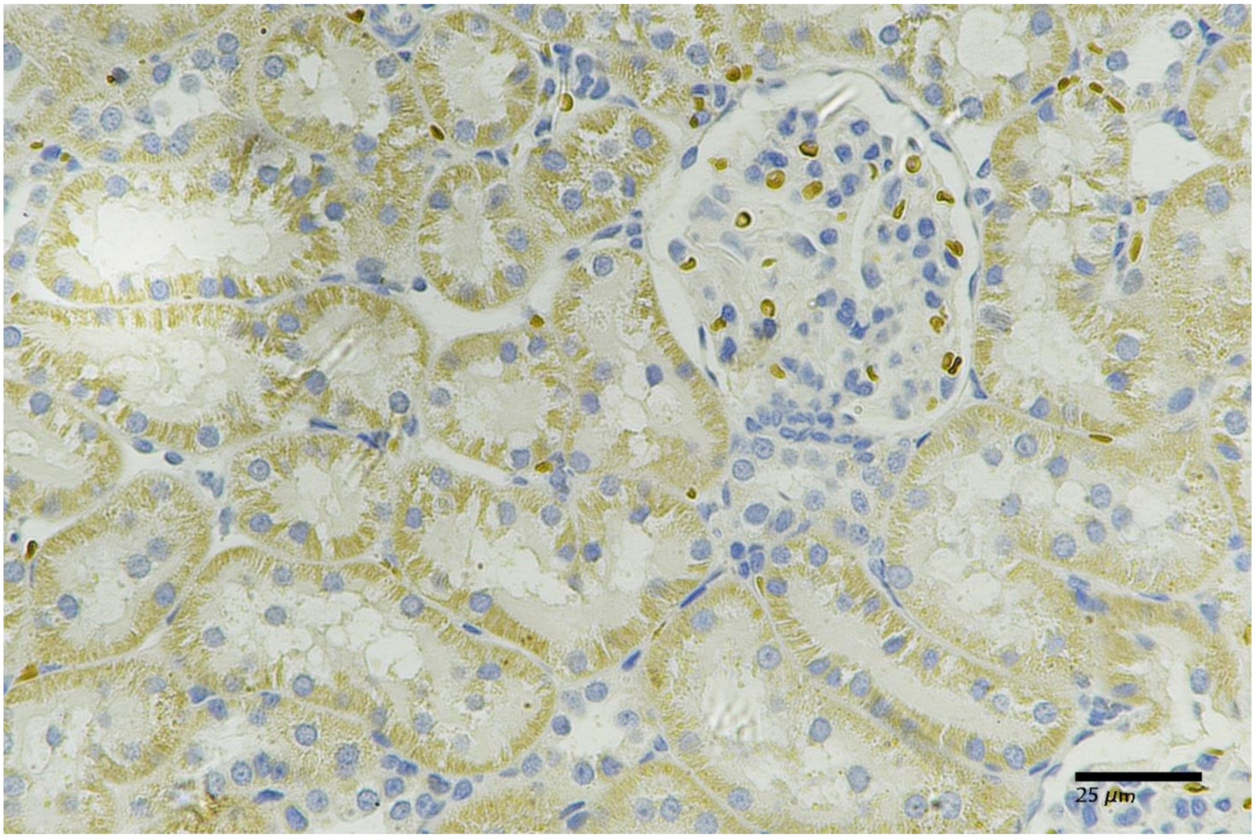
25 μm

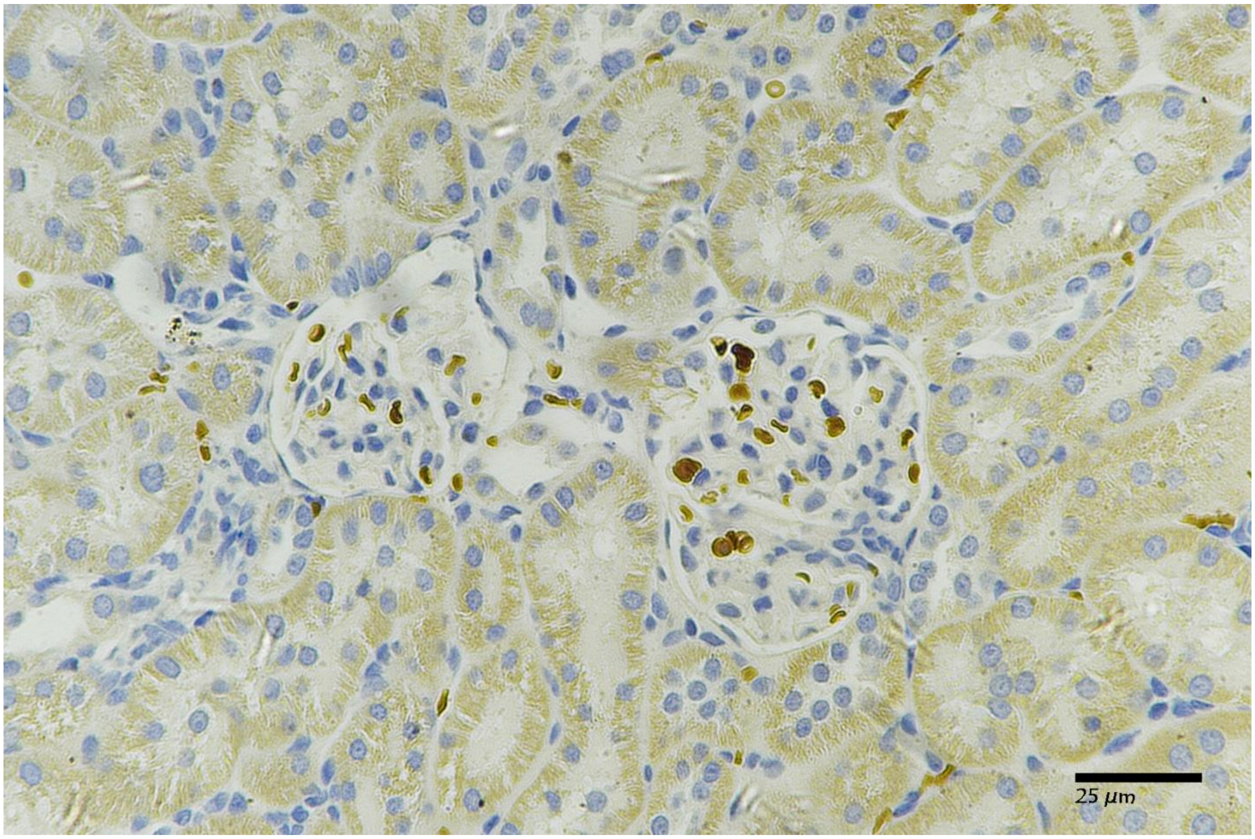
25 μm

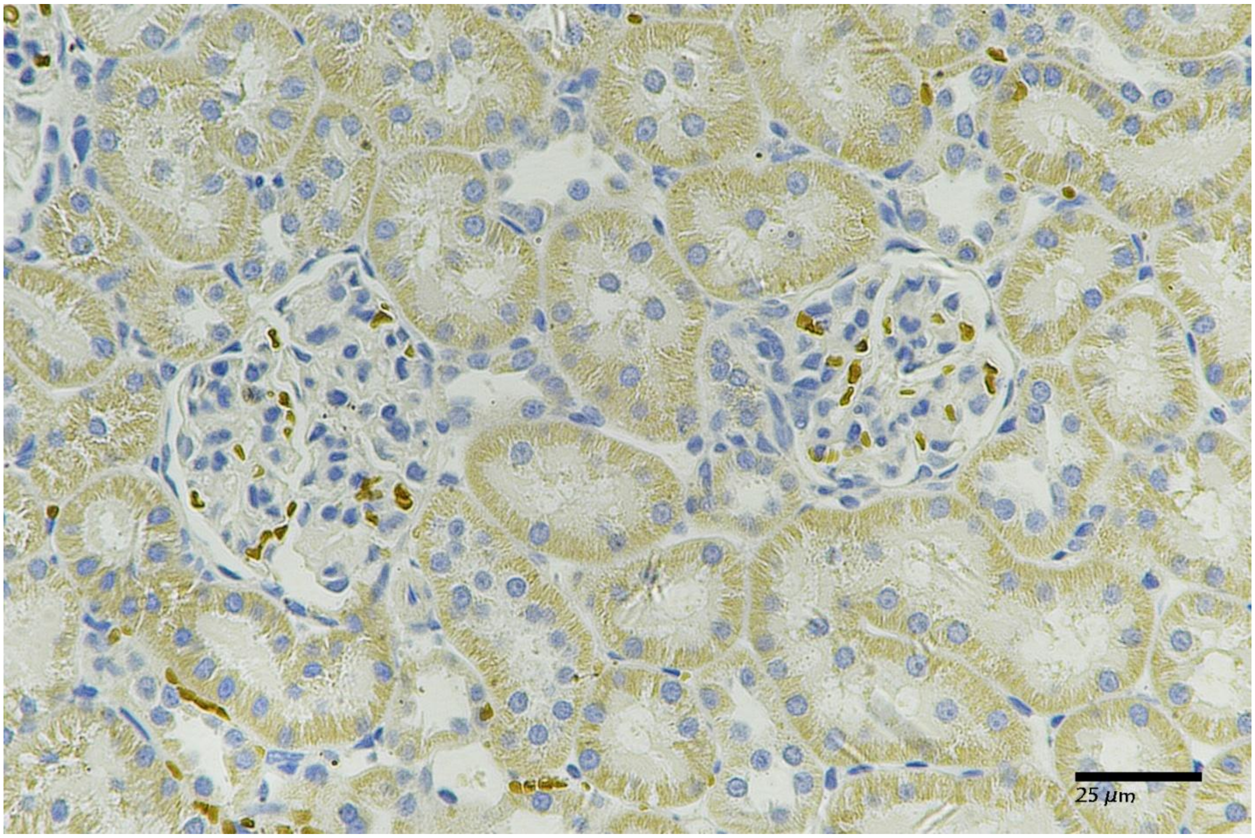
25 μm

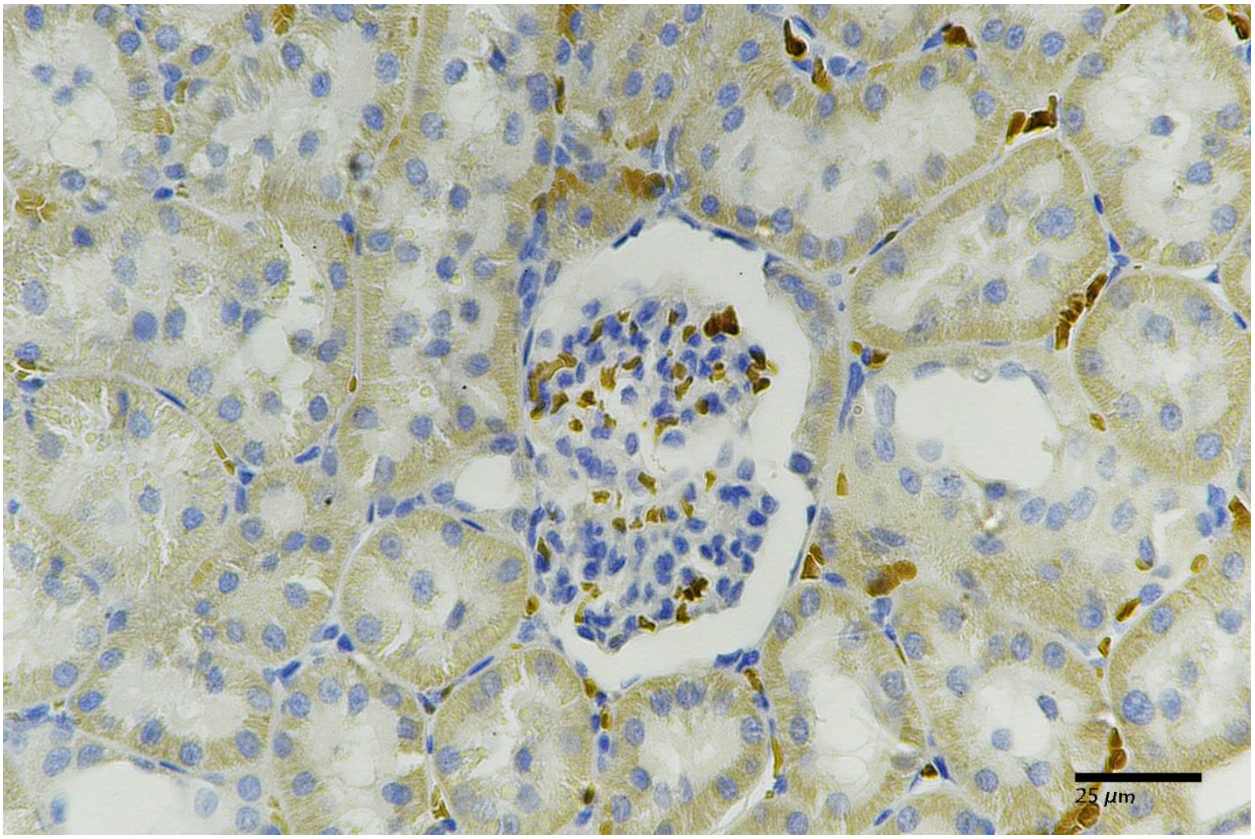
25 μm

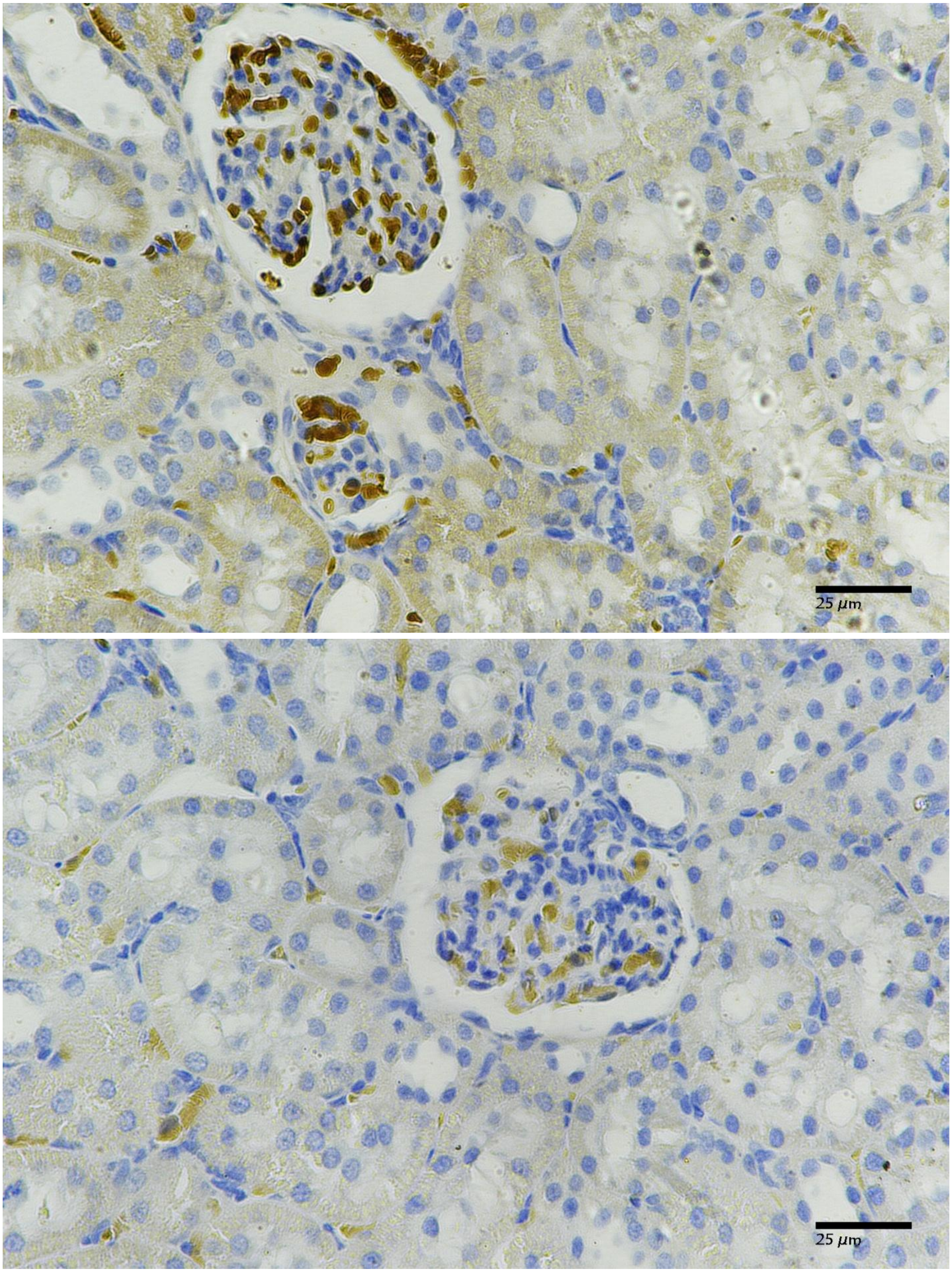
25 μm
25 μm

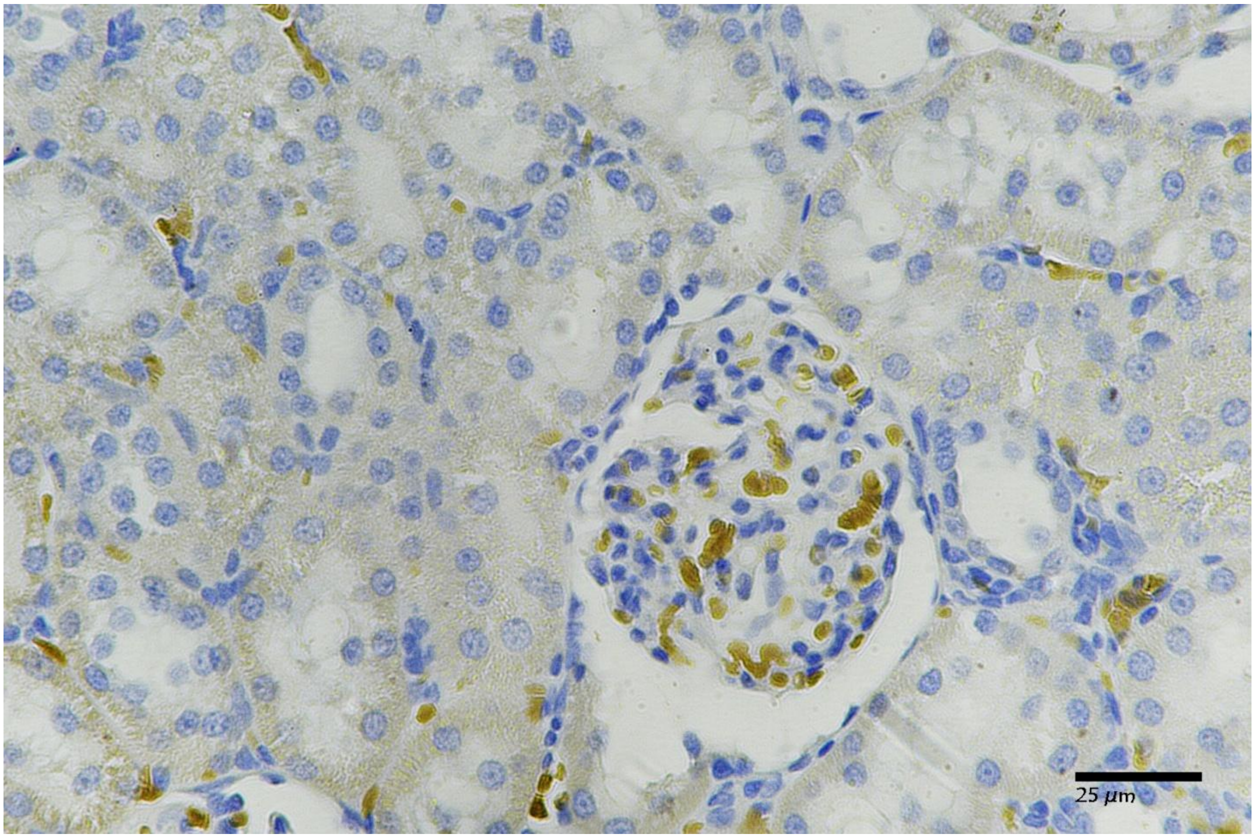
25 μm

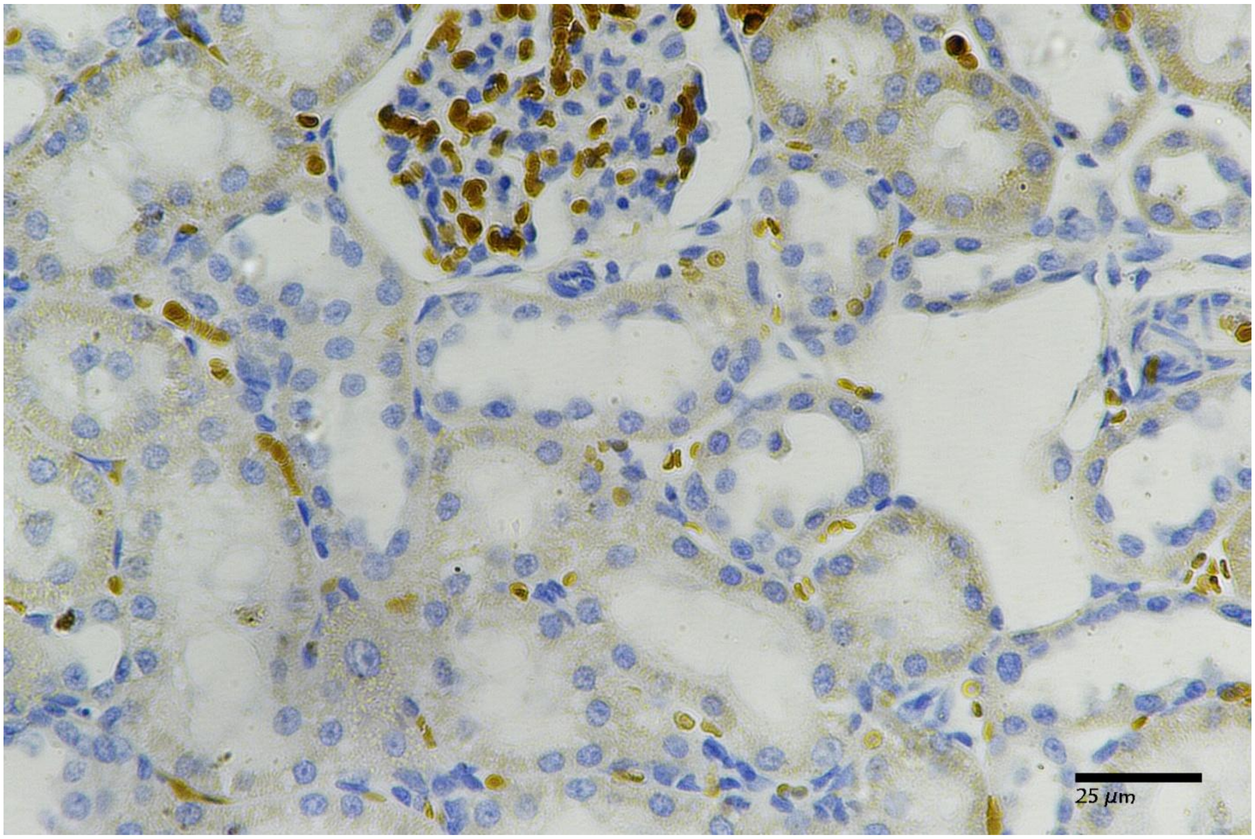
25 μm

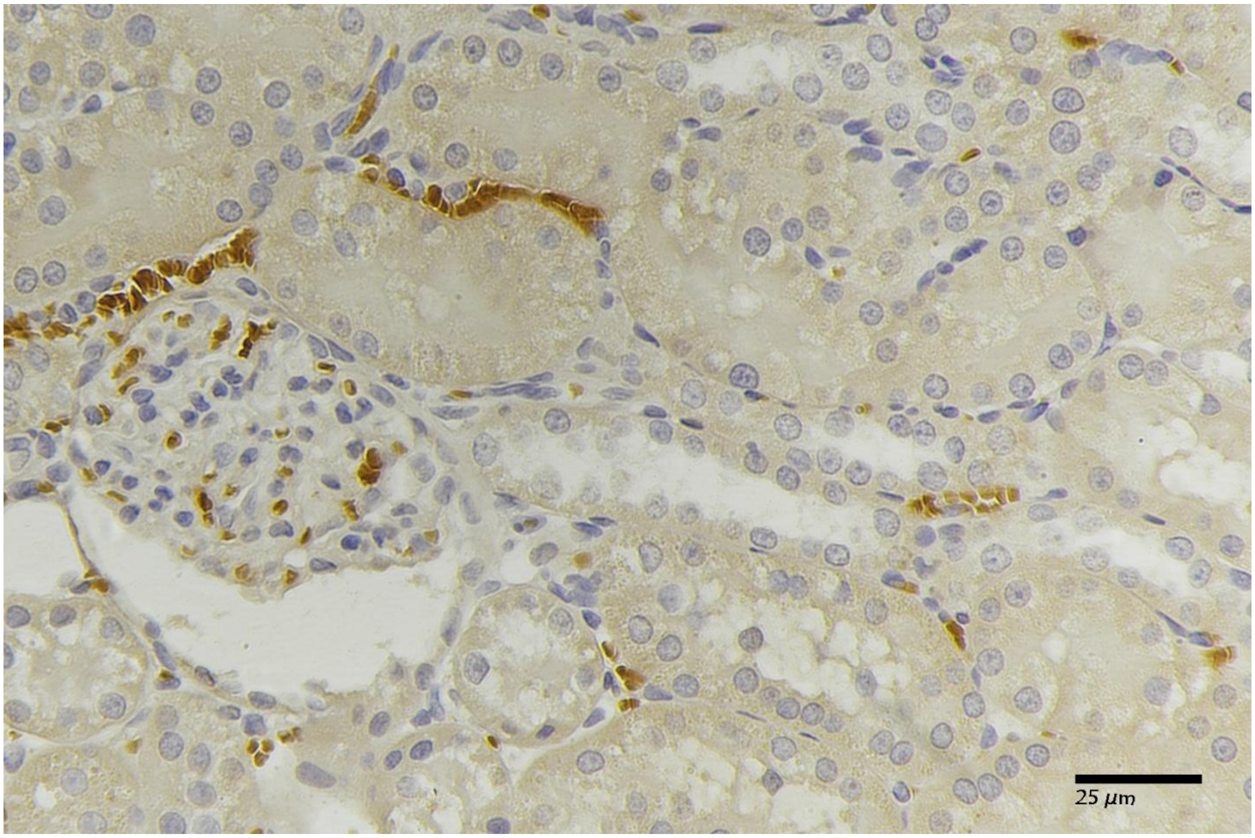
25 µm

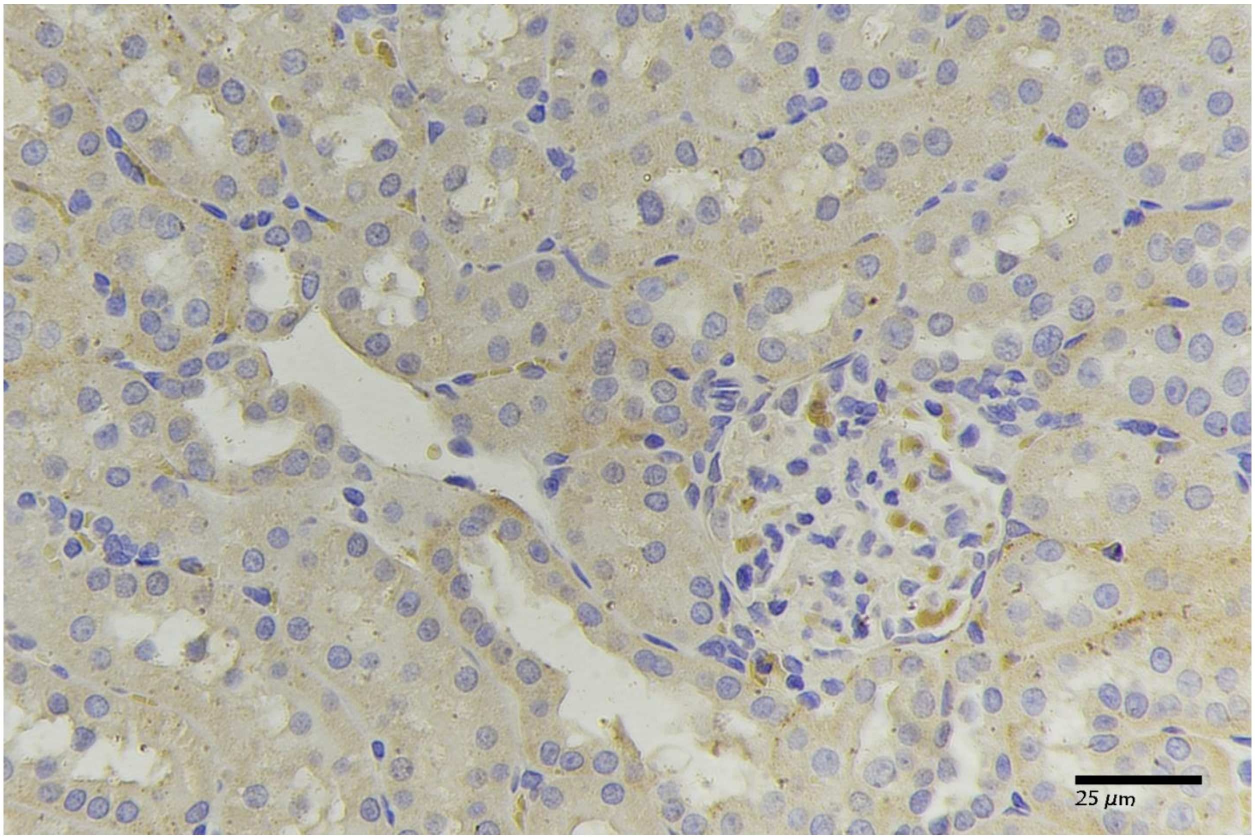
25 µm

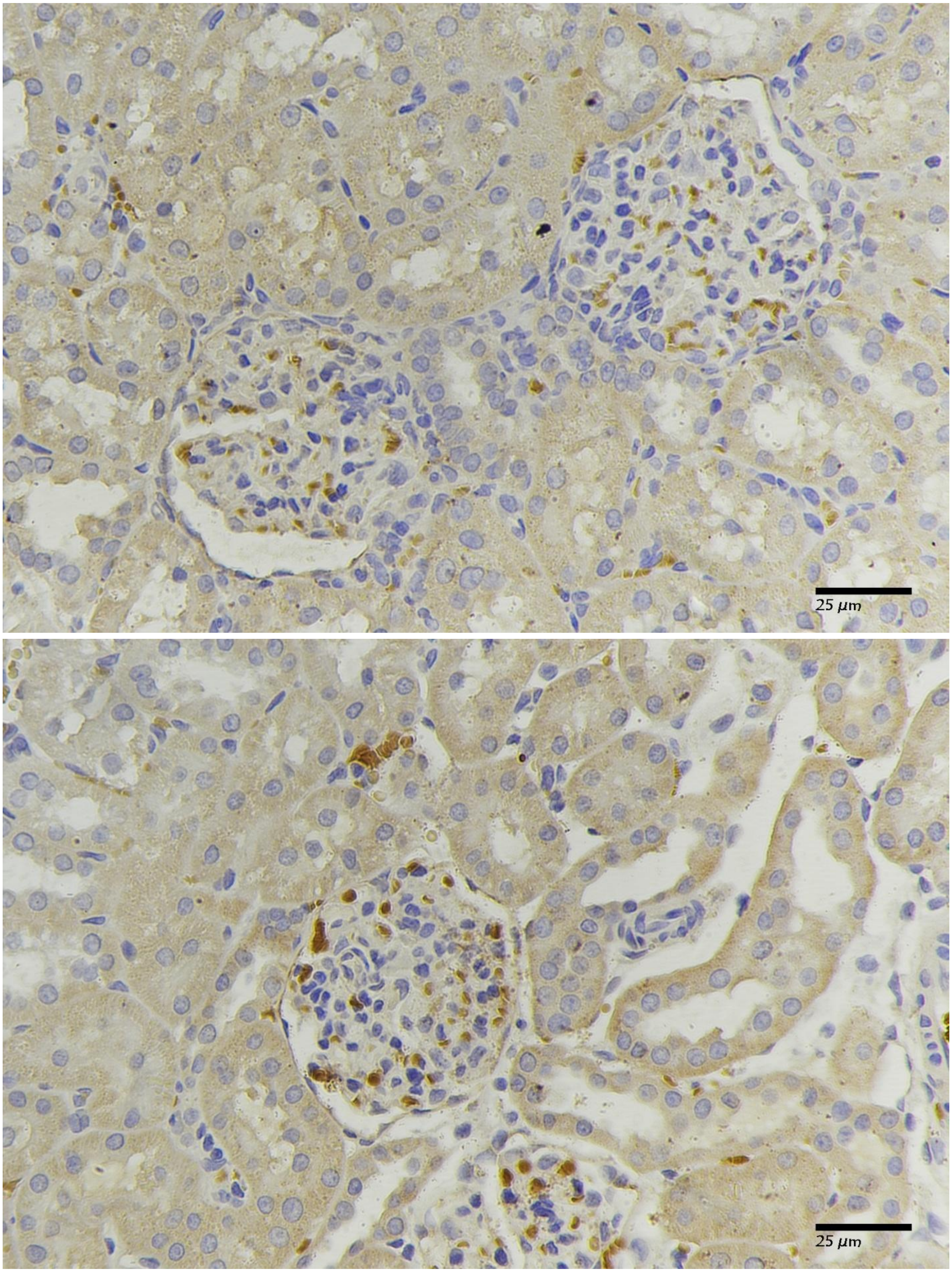
25 μm
25 μm

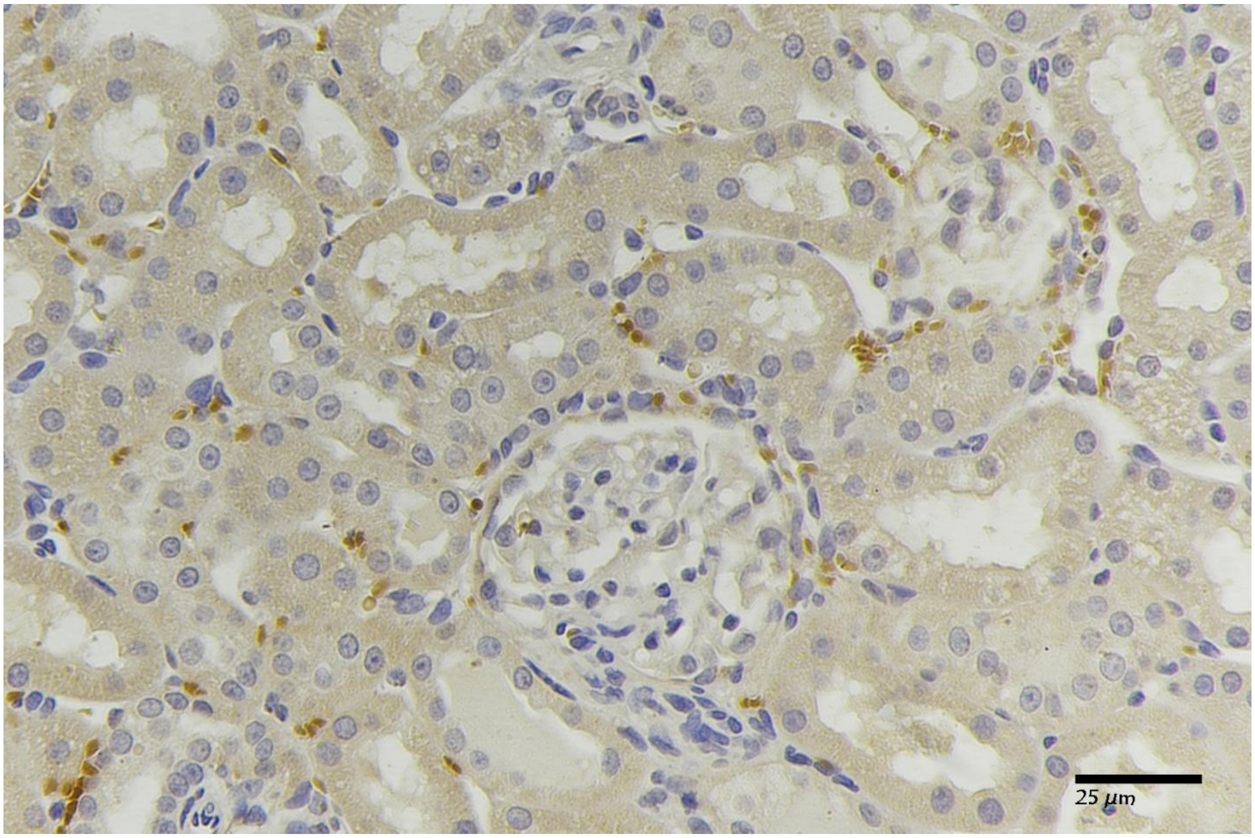
25 μm

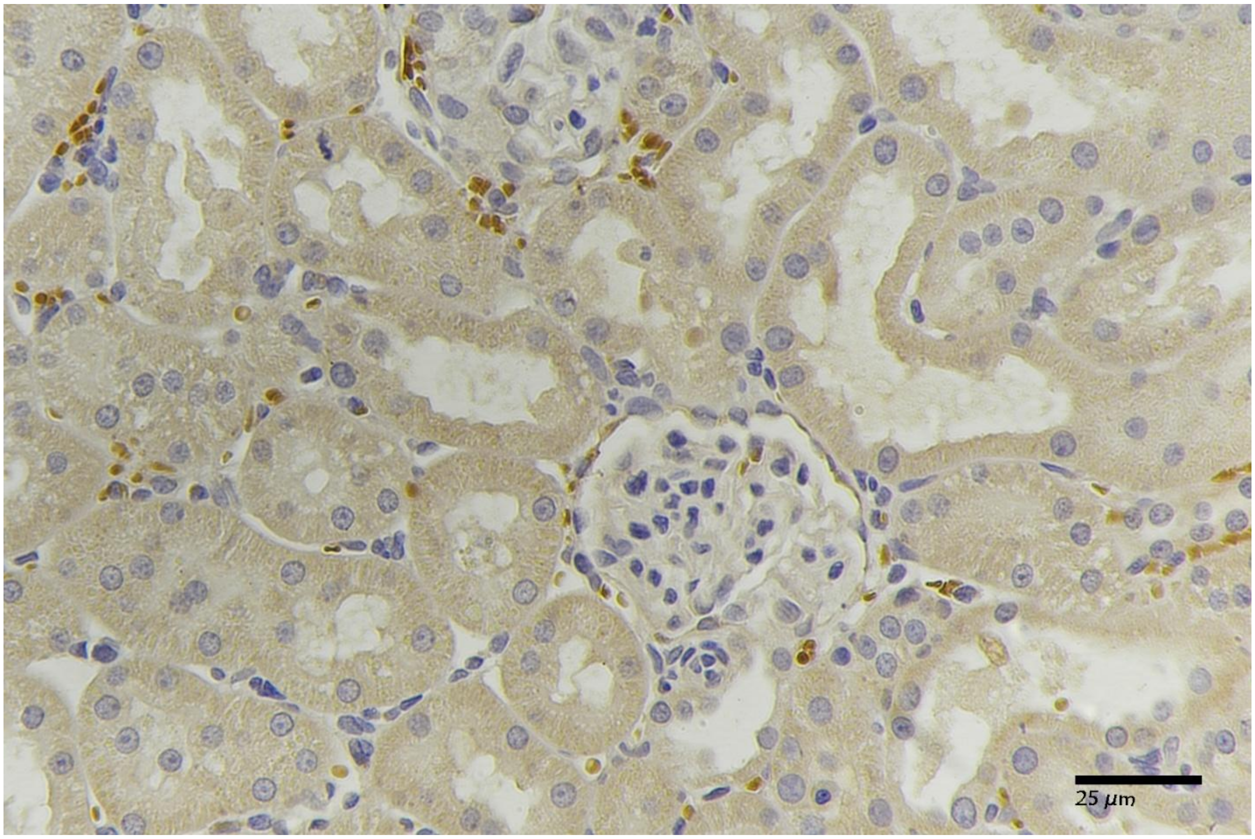
25 μm

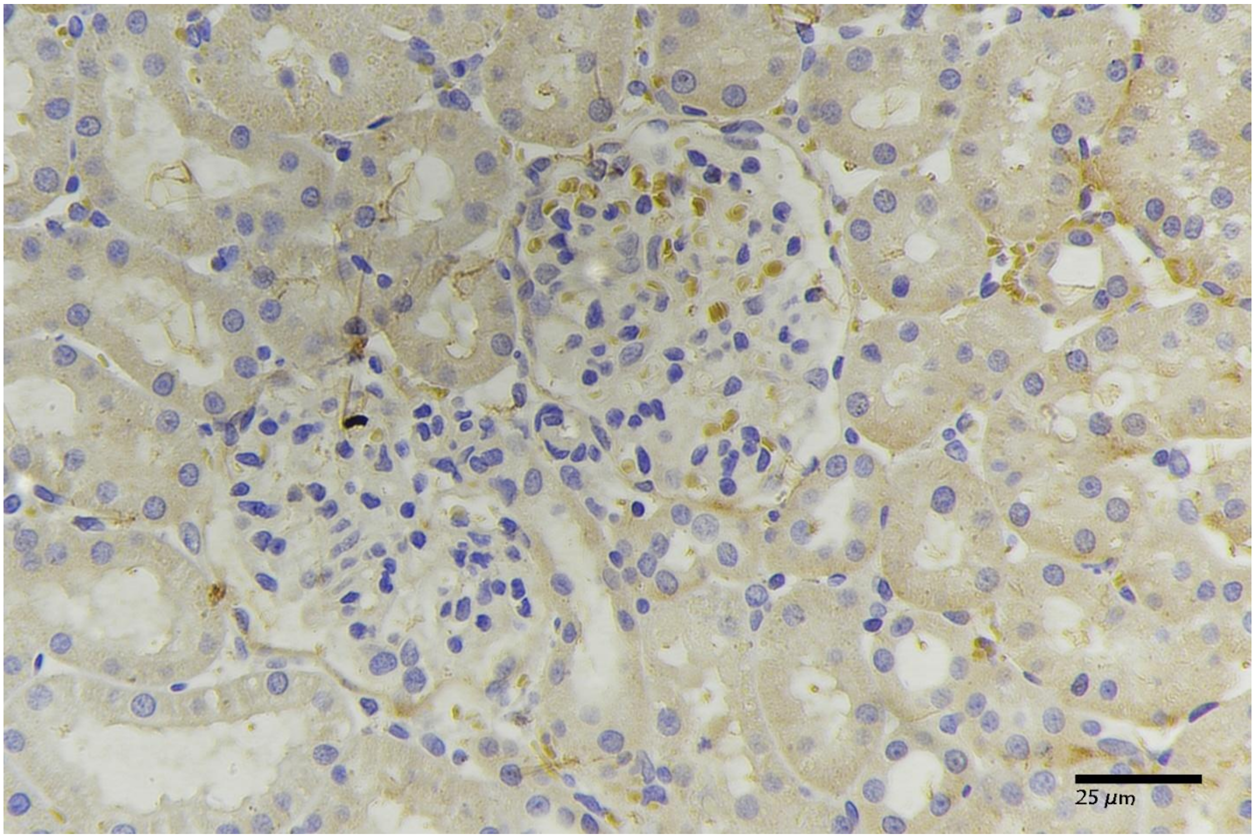
25 μm

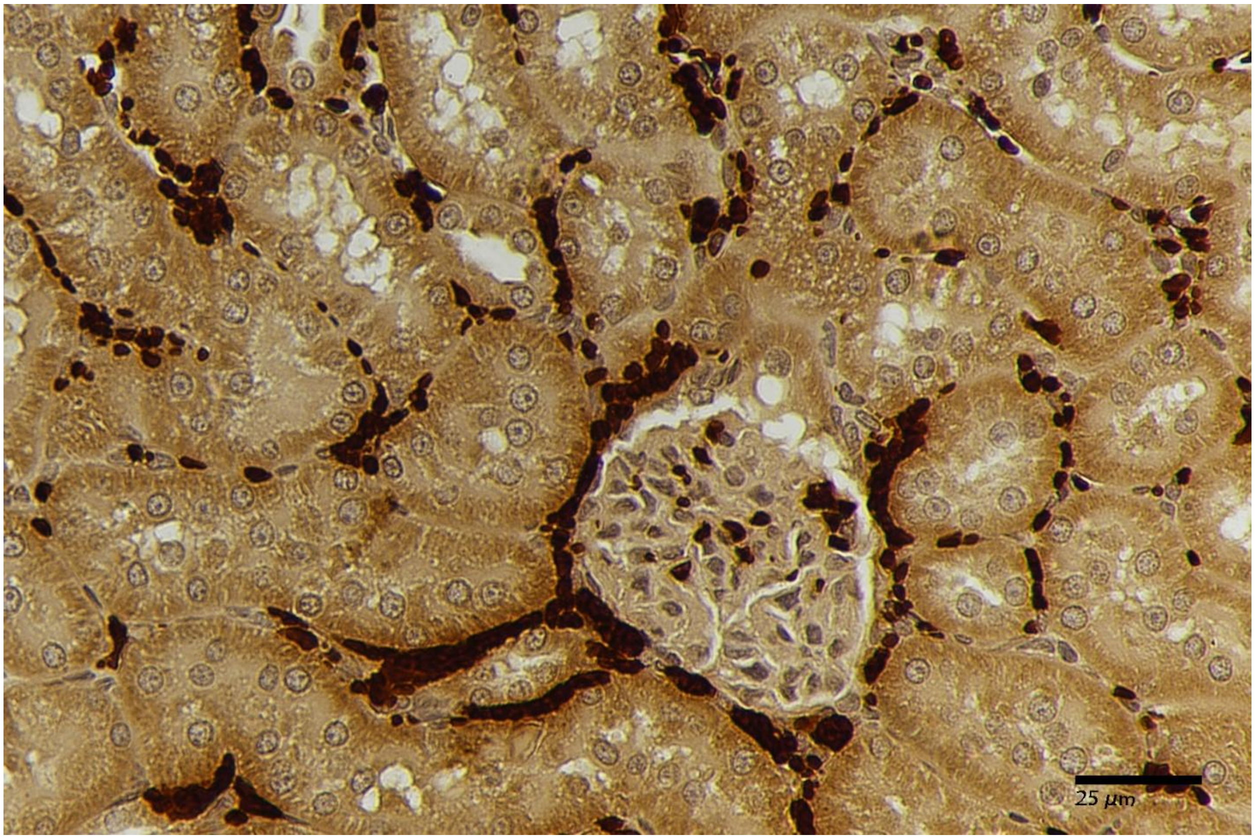
25 μm

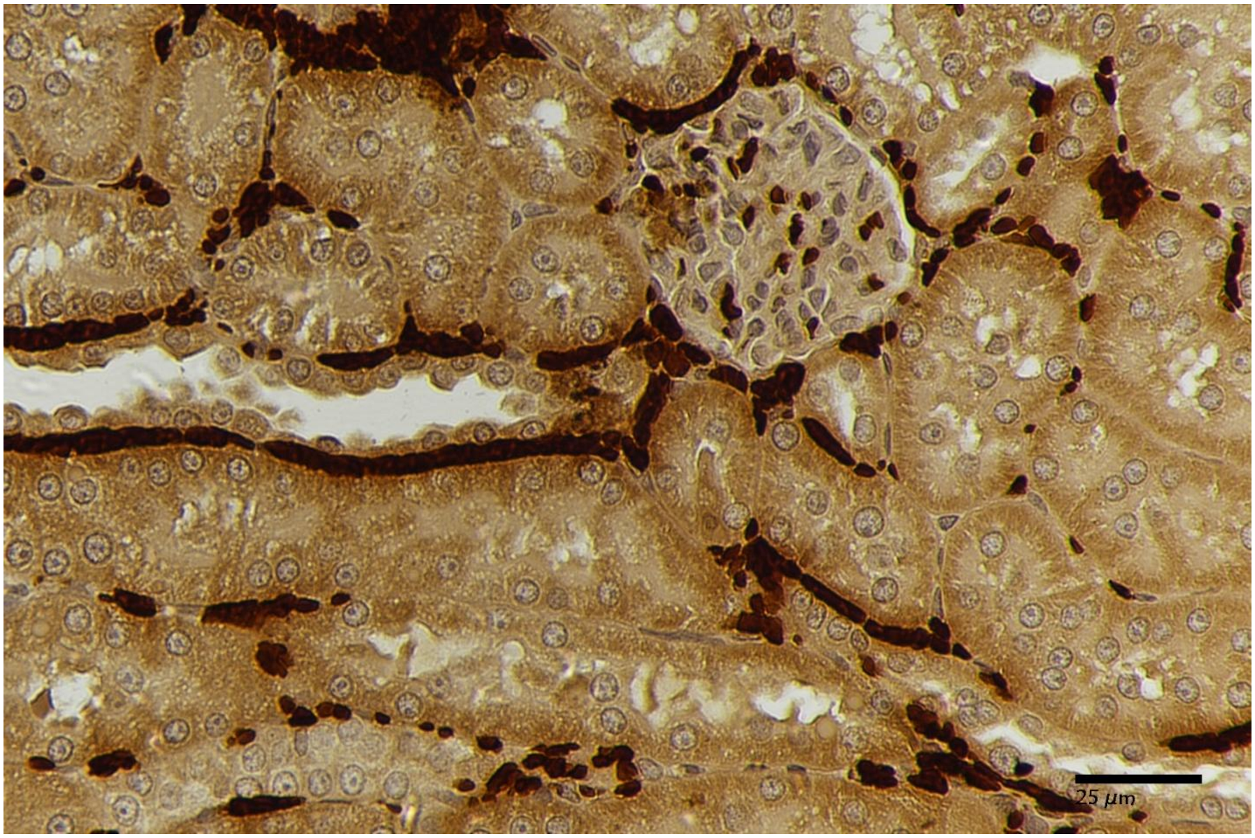
25 μm
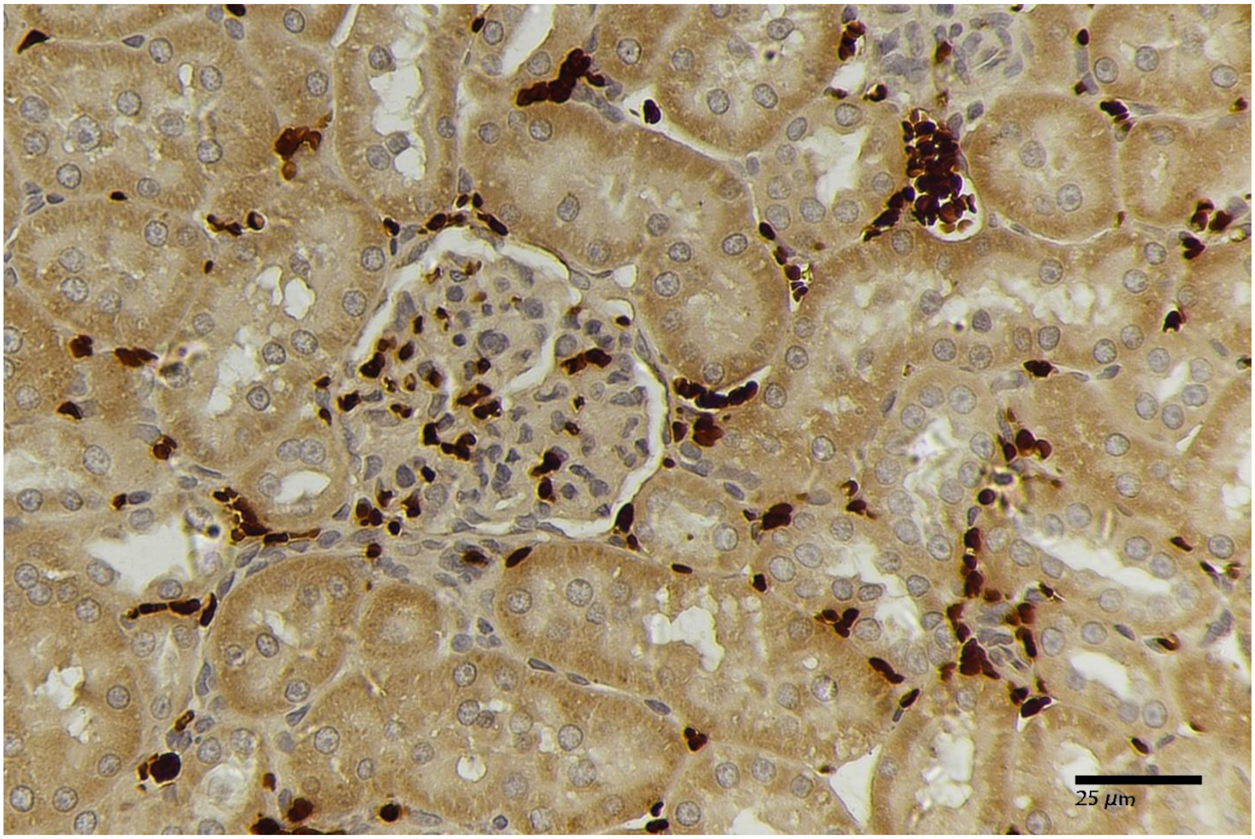
25 μm

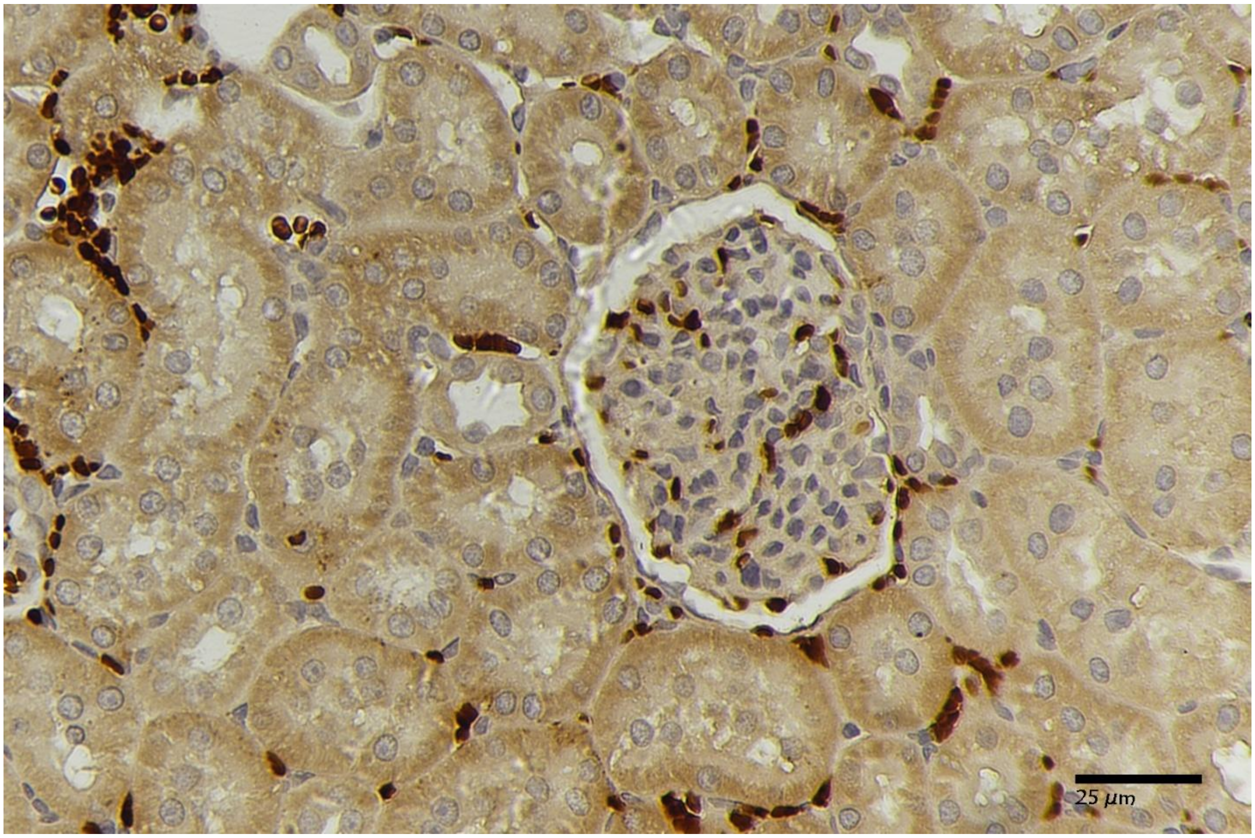
25 μm

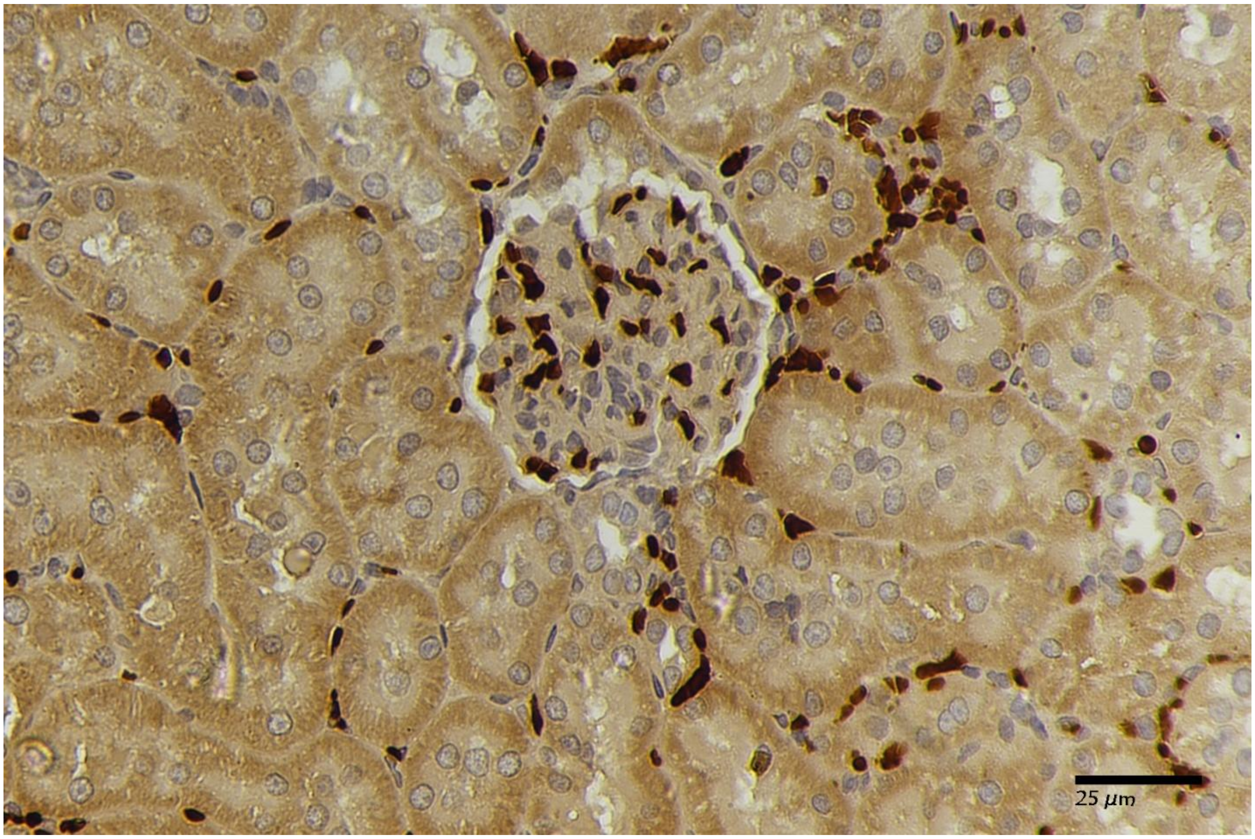
25 μm

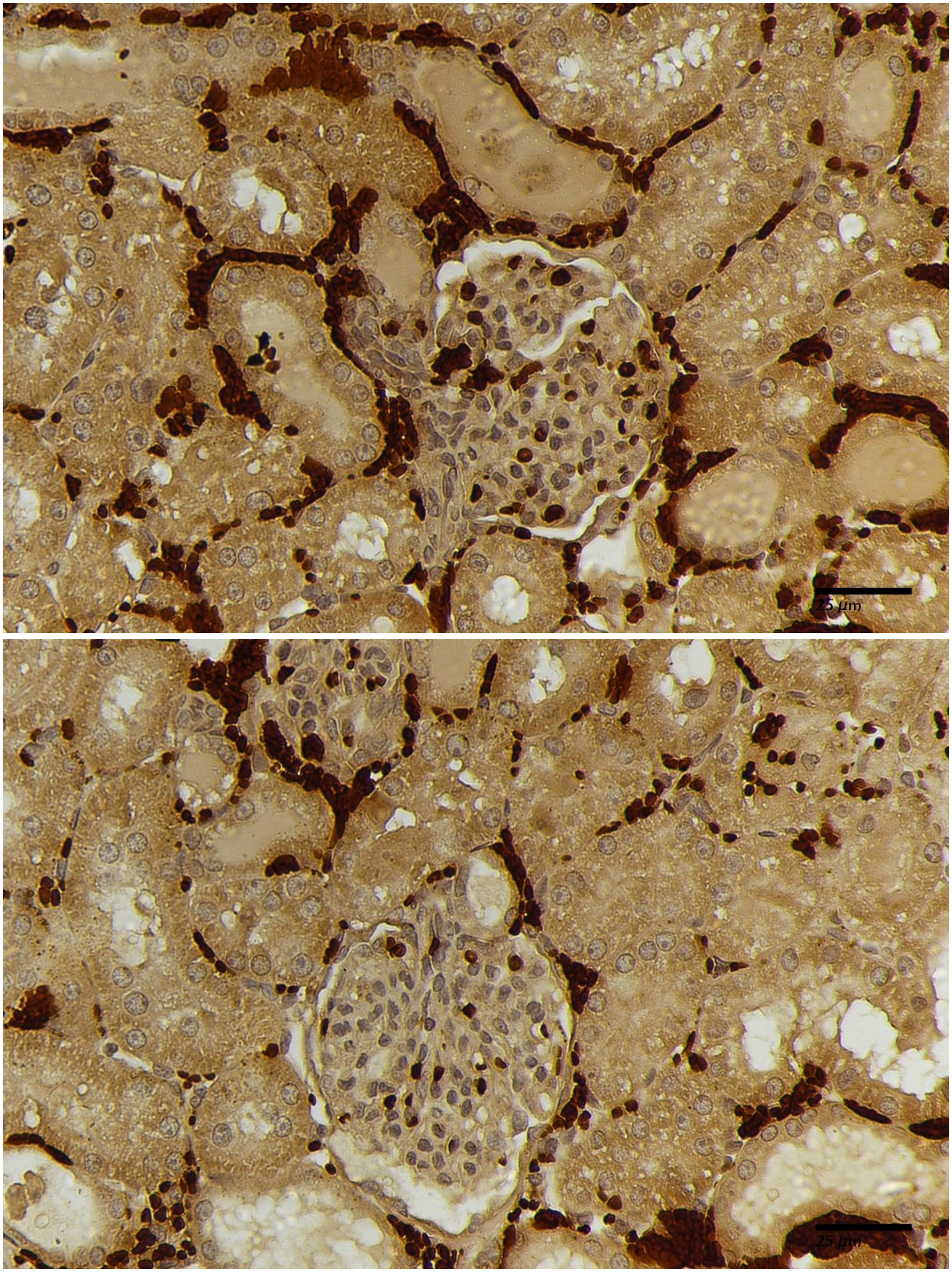
25 µm
25 µm
